# Regiodivergent sulfonylarylation of 1,3-enynes *via* nickel/photoredox dual catalysis†

**DOI:** 10.1039/d1sc04320j

**Published:** 2021-09-20

**Authors:** Ya Chen, Kun Zhu, Qingqin Huang, Yixin Lu

**Affiliations:** Department of Chemistry, National University of Singapore 3 Science Drive 3 Singapore 117543 Singapore chmlyx@nus.edu.sg; Joint School of National University of Singapore and Tianjin University, International Campus of Tianjin University Binhai New City Fuzhou Fujian 350207 China

## Abstract

Catalytic difunctionalization of 1,3-enynes represents an efficient and versatile approach to rapidly assemble multifunctional propargylic compounds, allenes and 1,3-dienes. Controlling selectivity in such addition reactions has been a long-standing challenging task due to multiple reactive centers resulting from the conjugated structure of 1,3-enynes. Herein, we present a straightforward method for regiodivergent sulfonylarylation of 1,3-enynes *via* dual nickel and photoredox catalysis. Hinging on the nature of 1,3-enynes, diverse reaction pathways are feasible: synthesis of α-allenyl sulfones *via* 1,4-sulfonylarylation, or preparation of (*E*)-1,3-dienyl sulfones with high chemo-, regio- and stereoselectivity through 3,4-sulfonylarylation. Notably, this is the first example that nickel and photoredox catalysis are merged to achieve efficient and versatile difunctionalization of 1,3-enynes.

## Abstract 2

A mild reaction protocol for regiodivergent sulfonylarylation of 1,3-enynes *via* dual nickel and photoredox catalysis has been developed, which led to efficient synthesis of α-allenyl sulfones or 1,3-dienyl sulfones.

## Graphical abstract

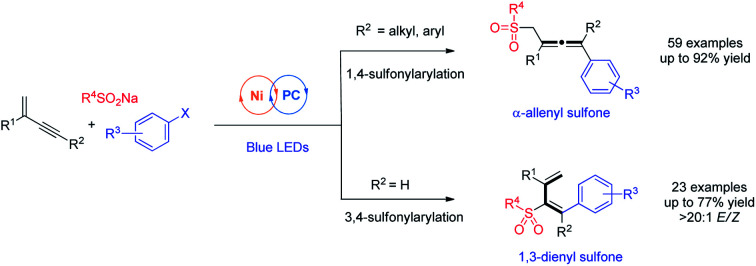

## Introduction

Owing to their ambident reactivity and ready availability, 1,3-enynes have been exploited as versatile building blocks to access various highly useful molecular architectures,^1^ including propargylic compounds,^2^ allenes^3^ and dienes.^4^ Transition-metal catalyzed difunctionalization of unactivated 1,3-enynes represents one of the most powerful approaches.^5,6^ In this context, radical 1,4-difunctionalization of 1,3-enynes has drawn much attention recently,^7^ and reports from the groups of Liu,^7*a*^ Bao,^7*b*–*d*^ Liu,^7*e*^ and Wang,^7*h*^ among others, demonstrated practicality and great potential of such synthetic strategies. Despite all the impressive advances that have been made to date, a number of notable limitations exist: (1) most reports are focused on alkyl or perfluoroalkyl radicals, and N-, Si-, P-, and S-radicals are virtually not explored. (2) Reaction conditions are often harsh, and excess oxidants, bases, or high temperatures are often required. (3) Radical 3,4-addition of 1,3-enynes remains elusive.

**Fig. 1:**
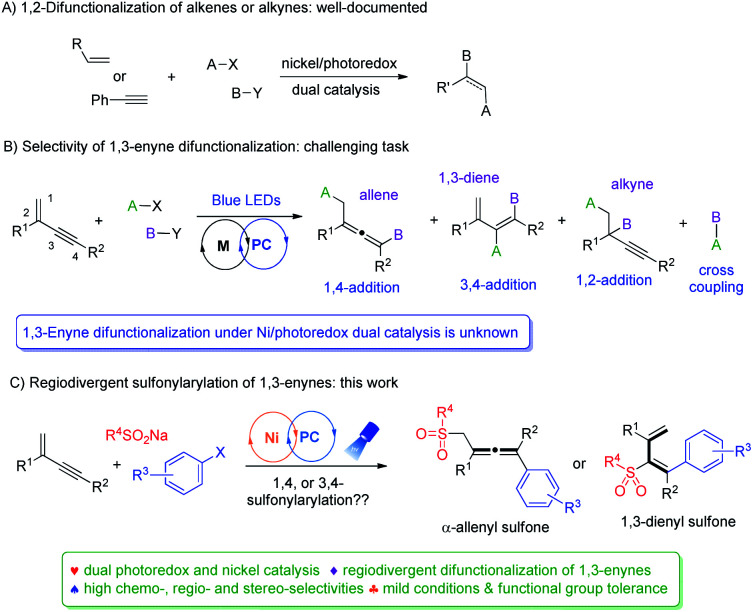
Difunctionalization of unsaturated bonds enabled by photoredox/nickel dual catalysis.

The merger of photoredox and transition metal catalysis has recently become a powerful synthetic strategy for the discovery of novel transformations.^8^ In this regard, dual nickel/photoredox catalysis evolved tremendously, finding remarkable applications in carbon–carbon bond forming reactions under mild conditions.^9^ Notably, a number of investigations are focused on 1,2-difunctionalization of conventional alkenes and alkynes (Fig. 1A).^10,11^ In contrast, difunctionalization of 1,3-enynes *via* Ni/photoredox dual catalysis is unknown (Fig. 1B). 1,3-Enynes contain conjugated carbon–carbon double bonds and triple bonds, thus controlling regioselectivities of such substrates in radical reactions is synthetically very challenging. If the radical addition takes place at the double bond site, functionalized propargylic compounds and allenes may be formed, *via* 1,2-addition and 1,4-addition pathways, respectively. Yet, another possibility is that the radical may add to the carbon–carbon triple bond, forming 1,3-dienes *via* a 3,4-addition pathway. Moreover, the presence of the carbon–carbon double bond in 3,4-difunctionalization products would naturally mean the existence of *E*/*Z* isomers, making this difficult synthetic task even more intimidating. Apparently, unproductive substrate cross-coupling poses another potential problem. Very recently, Glorius *et al.* reported radical carbonyl propargylation between 1,3-enynes and aldehydes by synergistic chromium and photoredox catalysis.^12^ Later, we developed the copper/photoredox dual-catalyzed decarboxylative 1,4-carbocyanation of 1,3-enynes, providing access to tetra-substituted allenes.^13^ Encouraged by these results, we hypothesized that by merging visible light photoredox and nickel catalysis, riding on the powerful single electron transfer of various nickel species, and with careful selection of radical precursors and 1,3-enyne substrates, regiodivergent difunctionalization of 1,3-enynes may be feasible.

Sulfone-containing molecules possess unique electronic properties and structural features, and consequently, they have played prominent roles in medicinal chemistry, materials science, and organic synthesis.^14,15^ Conventionally, sulfones are prepared through oxidation of sulfides, aromatic sulfonylation, and alkylation/arylation of sulfinates.^16^ These methods however do suffer from some serious drawbacks, *e.g.* the necessity of using foul-smelling thiols and employment of strong oxidants, and the requirement of harsh acidic conditions and high reaction temperatures. In this context, the addition of the sulfonyl radical to unsaturated bonds represents an attractive green approach to the efficient synthesis of various sulfone molecules. Allenic and 1,3-dienyl sulfones have shown to be versatile in conjugate addition, as well as in cyclization and cycloaddition reactions,^17–19^ yet, efficient synthesis of these molecules is under-developed. We therefore targeted their efficient preparation by employing readily available and cheap sulfonating agents, ideally *via* divergent synthetic pathways utilizing the same types of starting materials. Herein, we disclose divergent creation of α-allenyl sulfones and 1,3-dienyl sulfones by merging photoredox and nickel catalysis. Hinging on the alkyne terminal substituent, either 1,4-difunctionalization or 3,4-difunctionalization of 1,3-enynes took place smoothly under mild conditions, forming α-allenyl sulfones or 1,3-dienyl sulfones, respectively, and all the reactions proceeded with high chemo-, regio- and stereoselectivity (Fig. 1C).

## Results and discussion

We started our investigation by evaluating the reaction of 1,3-enyne **1a**, 4-bromobenzaldehyde **2a** and sodium 4-methylbenzenesulfinate **3a** in the presence of NiCl_2_·glyme, the dtbbpy ligand and Ru(bpy)_3_Cl_2_·6H_2_O under blue-light irradiation (Table 1). To our delight, regiospecific 1,4-addition was observed, and no 1,2- or 3,4-addition product could be detected. The molar ratio of reactants was found to be crucial for achieving high chemoselectivity. The employment of excess 1,3-enyne **1a** significantly favored the 1,4-sulfonylarylation product **4a**, and inhibited the undesired coupling between bromide **2a** and sulfinate **3a** (see ESI, Table S1†). It is noteworthy that the solvent also markedly influenced the reactivity and DMSO was the optimal solvent. Several common photocatalysts were subsequently examined, and inexpensive organic photosensitizer 4CzIPN (1,2,3,5-tetrakis(carbazol-9-yl)-4,6-dicyanobenzene) was found to be the best (Table 1, entries 1–4). Screening of nickel precatalysts and ligands was followed, and NiCl_2_·glyme (5 mol%) and diOMebpy (7 mol%) were identified to be the best combination, leading to the formation of sulfonylated allene product **4a** in 72% isolated yield in a regiospecific manner (entries 5–10, for a complete screening, see ESI, Table S2†). We also performed a number of control experiments, showing that the photocatalyst, nickel precatalyst, ligand, light, and inert nitrogen were all essential, without which the reaction would not take place (entries 11–15).

**Table 1:** Optimization of the 1,4-sulfonylarylation reaction conditions^a^

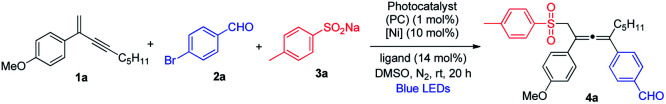				
Entry	PC	[Ni]	Ligand	Yield^b^ (%)
1	Ru(bpy)_3_Cl_2_·6H_2_O	NiCl_2_·glyme	dtbbpy	55
2	Ru(bpy)_3_(PF_6_)_2_	NiCl_2_·glyme	dtbbpy	45
3	[Ir(dFCF_3_ppy)_2_(dtbbpy)]PF_6_	NiCl_2_·glyme	dtbbpy	62
4	4CzIPN	NiCl_2_·glyme	dtbbpy	64
5	4CzIPN	NiCl_2_	dtbbpy	35
6	4CzIPN	NiCl_2_·6H_2_O	dtbbpy	11
7	4CzIPN	NiCl_2_·glyme	diOMebpy	73
8	4CzIPN	NiCl_2_·glyme	bpy	62
9	4CzIPN	NiCl_2_·glyme	Phen	0
10^c^	4CzIPN	NiCl_2_·glyme	diOMebpy	72
11	—	NiCl_2_·glyme	diOMebpy	0
12	4CzIPN	—	diOMebpy	0
13	4CzIPN	NiCl_2_·glyme	—	0
14^d^	4CzIPN	NiCl_2_·glyme	diOMebpy	0
15^e^	4CzIPN	NiCl_2_·glyme	diOMebpy	0

a Reaction conditions: **1a** (0.2 mmol), **2a** (0.1 mmol) and **3a** (0.12 mmol) in DMSO (1.0 mL), photocatalyst (1 mol%), nickel-precatalyst (10 mol%), ligand (14 mol%), at room temperature, 30 W blue LEDs, 20 h.

b Isolated yield.

c NiCl_2_·glyme (5 mol%), diOMebpy (7 mol%).

d No light.

e Reaction was performed in air.

**Scheme 1:**
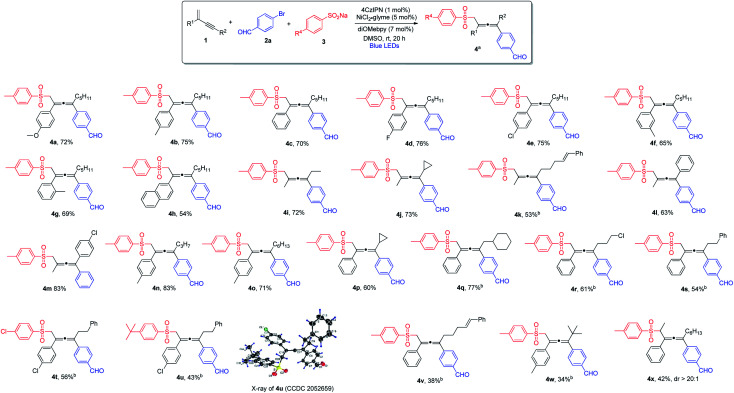
Variation of 1,3-enynes. ^a^Conditions: **1** (0.4 mmol), **2a** (0.2 mmol) and **3** (0.24 mmol) in DMSO (2.0 mL), 4CzIPN (1 mol%), NiCl_2_·glyme (5 mol%), diOMebpy (7 mol%), at room temperature, 30 W blue LEDs, 20 h. Isolated yield. ^b^NiCl_2_·glyme (10 mol%), diOMebpy (14 mol%).

With the optimized reaction conditions in hand, we investigated the substrate scope for the 1,4-sulfonylarylation of 1,3-enynes, first focusing on the variation of 1,3-enyne structures, and the results are summarized in Scheme 1. For the alkene C2-aryl substituent, aryls containing both electron-donating and electron-withdrawing groups were well tolerated, and the corresponding tetra-substituted allenes were obtained in good yields (**4a–4e**). The reaction was applicable to aryls with different substitution patterns (**4f** and **4g**). 1,3-Enyne **1h** with a 2-naphthyl substituent was also found to be a suitable substrate, forming the corresponding allene in a satisfactory yield (**4h**). The C2-substituents for 1,3-enynes were not limited to aryls, 1,3-enynes bearing a 2-methyl group could also be employed, and the allene products were obtained in moderate to high yields (**4i–4m**). The alkynyl moiety in the 1,3-enyne structures could also be varied; the C4-substituent could be linear alkyl chains of different lengths (**4n** and **4o**), or branched alkyl groups (**4p** and **4q**), and consistently good yields were obtained. Furthermore, when 1,3-enyne substrates with structurally diverse C4-linear alkyl chains, *i.e.* bearing a chloride, a phenyl group, and a remote alkene function, were employed, the desired allene products were formed in acceptable yields (**4r–4v**). Interestingly, when sterically highly demanding 1,3-enyne **1w** bearing a C4-*tert*-butyl substituent was utilized, we were able to isolate the very congested tetra-substituted allene **4w**, albeit in low yield. To our surprise, when 1,3-enyne containing an internal alkene was subjected to the reaction, the allene product **4x** was obtained with excellent diastereoselectivity (dr > 20 : 1).

**Scheme 2:**
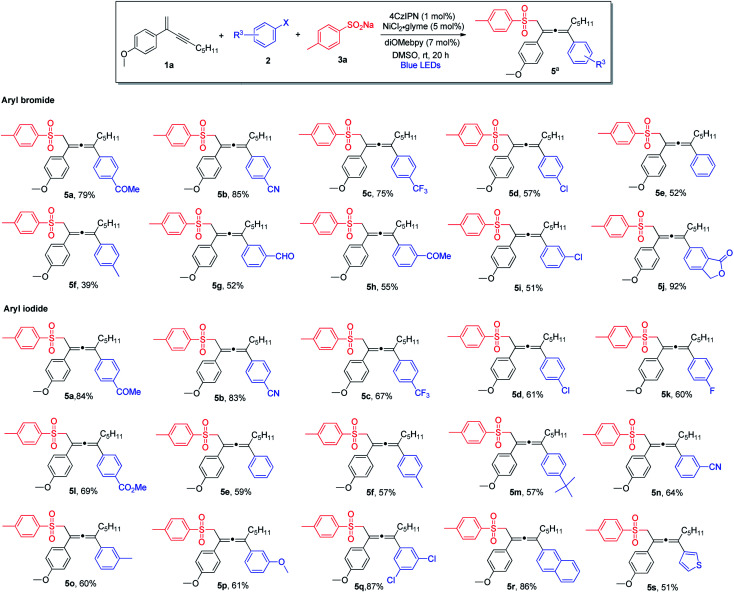
Variation of aryl halides. ^a^Conditions: **1a** (0.4 mmol), **2** (0.2 mmol) and **3a** (0.24 mmol) in DMSO (2.0 mL), 4CzIPN (1 mol%), NiCl_2_·glyme (5 mol%), diOMebpy (7 mol%), at room temperature, 30 W blue LEDs, 20 h. Isolated yield.

Next, we explored the scope of aryl halides (Scheme 2). In general, aryl bromides and aryl iodides bearing electron-withdrawing substituents were found to be the most suitable substrates, whereas aryl halides with electron-donating substituents tended to deliver the products in lower chemical yields. Notably, this mild synthetic protocol was compatible with a wide range of functional groups and structural moieties, including ketone (**5a** and **5h**), nitrile (**5b** and **5n**), trifluoromethyl (**5c**), aldehyde (**5g**) and ester (**5l**). Moreover, the allene products containing chlorine or fluorine on the aromatic ring were obtained with equal efficiency (**5d**, **5i** and **5k**), and such halogen atoms could be engaged for further structural elaborations when necessary. Disubstituted aryl halides and bicyclic substrates including phthalide- and naphthyl-derived halides proved to be good substrates, and the corresponding products were obtained in excellent yields (**5q**, **5j** and **5r**). In addition, thiophene-derived iodide worked well in this three-component cross-coupling reaction (**5s**). However, *ortho*-substituted aryl halides were found to be unsuitable substrates.

**Scheme 3:**
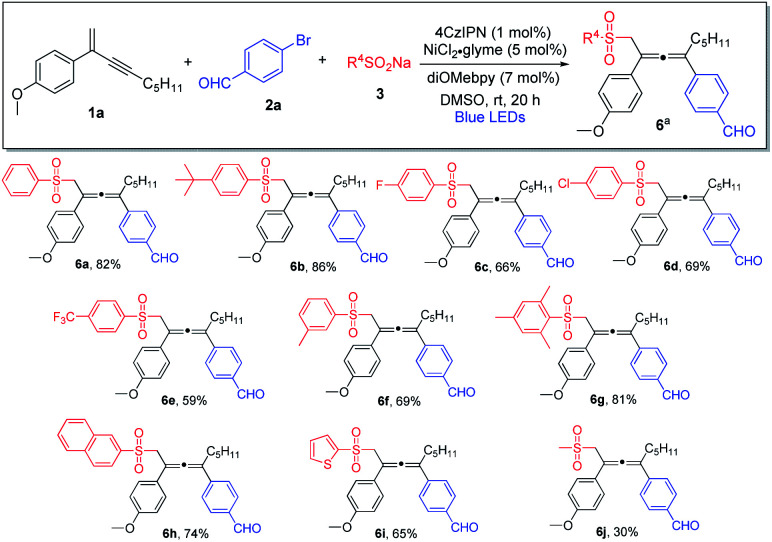
Variation of sulfinates. ^a^Conditions: **1a** (0.4 mmol), **2a** (0.2 mmol) and **3** (0.24 mmol) in DMSO (2.0 mL), 4CzIPN (1 mol%), NiCl_2_·glyme (5 mol%), diOMebpy (7 mol%), at room temperature, 30 W blue LEDs, 20 h. Isolated yield.

We subsequently investigated the generality of the radical precursors, and accordingly, a series of sodium aryl sulfinates were examined (Scheme 3). Employment of benzenesulfinate and sodium sulfinate containing *t*-butyl-substituted phenyl led to the formation of the allene products in high yields (**6a** and **6b**). Aryl sulfinates containing fluorine, chlorine, trifluoromethyl, and methyl groups were well tolerated, and the corresponding products were obtained in decent yields (**6c–6f**). Interestingly, sterically hindered sulfinate was shown to be an excellent substrate as well, and the cross-coupling product **6g** was formed in 81% yield. Moreover, the reaction was found to be compatible with sodium naphthyl and thienyl sulfinates (**6h** and **6i**). It is noteworthy that sodium alkyl sulfinate could also be employed, albeit having lower reactivity, and consequently a lower chemical yield was obtained (**6j**).

In order to develop regiodivergent sulfonylation of 1,3-enynes, we hypothesized that small structural variation of 1,3-enynes may provide a straightforward solution: the presence of an alkyl or aryl substituent *versus* a hydrogen atom may affect the stability of radical species formed during the reaction, which may also lead to certain elementary organometallic steps, *e.g.* migratory insertion, which are more favorable, thus leading to different regiodivergent pathways. To test the above hypothesis, we chose terminal 1,3-enyne **1a′** and examined its reaction under the optimal reaction conditions. Gratifyingly, 2-sulfonyl 1,3-diene **7a** was obtained in 70% yield as a single isomer, which was formed *via* the 3,4-addition pathway (see ESI, Table S4†). We suspected that the *E*/*Z* isomerization may take place *via* the energy transfer process when photocatalysts possessing different triplet state energies are employed. Indeed, when [Ir(dFCF_3_ppy)_2_(dtbbpy)]PF_6_ (*E*_T_ = 59.4 kcal mol^−1^)^11*b*^ or Ir(dFppy)_3_ (*E*_T_ = 60.1 kcal mol^−1^)^20^ was utilized, the *E*/*Z* ratio of product **7a** decreased drastically (Table S4†). Similarly, control experiments confirmed that the photocatalyst, nickel catalyst, ligand, and light are indispensable for the observed 3,4-sulfonylarylation of 1,3-enynes (see ESI, Table S5†).

**Scheme 4:**
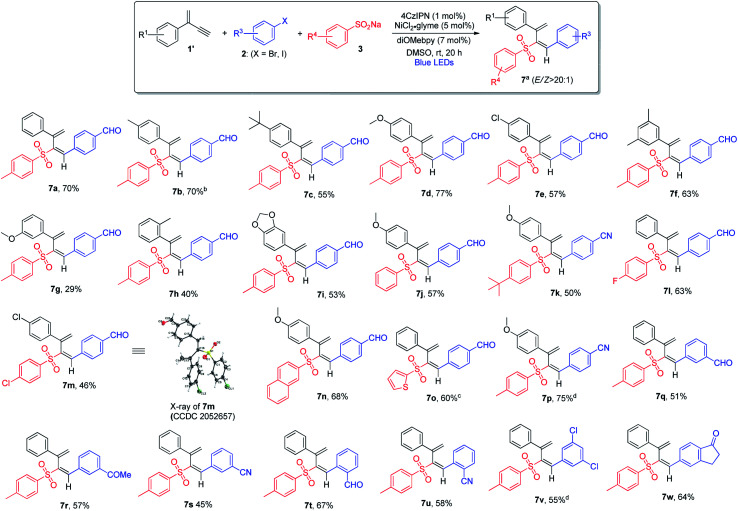
Variation of 3,4-addition of 1,3-enynes. ^a^Conditions: **1′** (0.4 mmol), aryl bromide **2** (0.2 mmol) and **3** (0.24 mmol) in DMSO (2.0 mL), 4CzIPN (1 mol%), NiCl_2_·glyme (5 mol%), diOMebpy (7 mol%), at room temperature, 30 W blue LEDs, 20 h. Isolated yield. *Z*/*E* selectivity was determined by ^1^H NMR analysis. ^b^The *E*/*Z* ratio of product **7b** is 14 : 1. ^c^The *E*/*Z* ratio of product **7o** is 11 : 1. ^d^Aryl iodide **2** (0.2 mmol) was utilized.

The scope of 3,4-sulfonylarylation of 1,3-enynes for the assembly of (*E*)-1,3-dienyl sulfones was also found to be broad, which is summarized in Scheme 4. The addition of aryl sulfinates **3** and aryl halides **2** across the triple bond of 1,3-enyne **1′** took place in an *anti*-fashion to furnish the corresponding 1,3-diene products **7** in moderate to good yields. For terminal 1,3-enynes, aryls bearing both electron-withdrawing and electron-donating groups were found to be suitable, and excellent regio- and stereo-selectivities were attainable (**7a–7e**). The substitution pattern of aromatic rings was also examined, and 1,3-enynes containing either *ortho*- or *meta*-substituted aryl rings appeared to be less ideal substrates (**7f–7h**). When dioxole-derived 1,3-enyne was employed, the cross-coupling product was obtained in a moderate yield (**7i**). With regard to the sulfinate substrate, sodium aryl sulfinates bearing electron-rich or electron-deficient aryl groups were equally good, and the products were obtained in pure isomeric forms (**7j–7m**). Moreover, sodium naphthyl and thienyl sulfinates were both amenable to the reaction (**7n** and **7o**). When different aryl bromides and aryl iodides bearing electron-withdrawing groups were utilized, regardless of the substitution pattern, the 1,3-diene products were obtained in satisfactory yields (**7p–7u**). However, electron-rich aryl halides failed to form the desired products in reasonable yields. In contrast to the inability of applying *ortho*-substituted aryl halides to the formation of allene products *via* 1,2-addition, the 3,4-sulfonylarylation of 1,3-enynes was applicable to *ortho*-substituted aryl halides (**7t** and **7u**). When aryl halide **2v** bearing both iodine and chlorine atoms was subjected to this cross-coupling reaction, chemoselectivity was observed and the reaction took place solely at the iodide site (**7v**). The employment of the bicyclic substrate was also well tolerated, and the product was obtained in a good yield (**7w**).

**Scheme 5:**
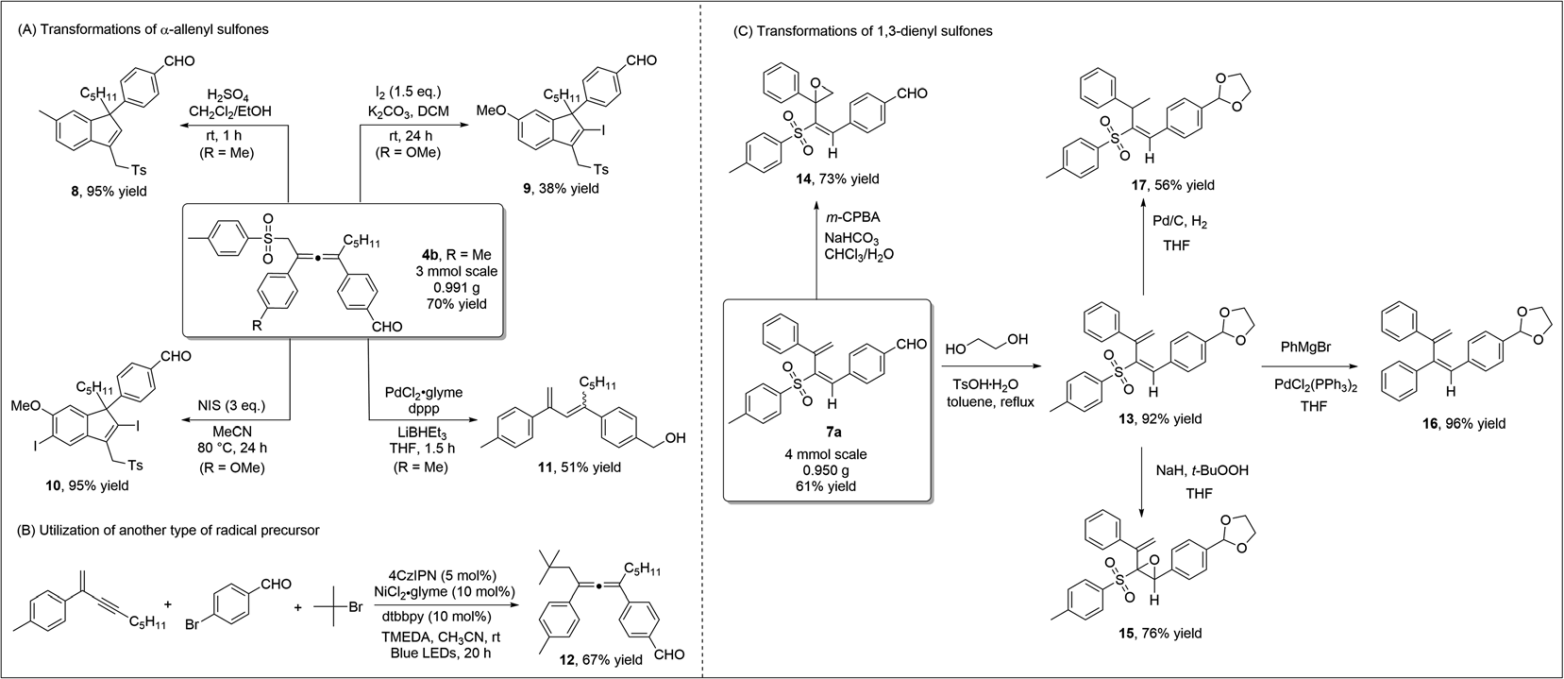
Scale-up reactions and synthetic applications.

To highlight the practicability and robustness of our methodology, we carried out gram-scale synthesis of diverse products (Scheme 5). Both allene product **4b***via* 1,4-sulfonylarylation of 1,3-enyne, and 1,3-dienyl sulfone **7a** through 3,4-sulfonylarylation of 1,3-enyne, were obtained in good yields at the gram-scale. When allene **4b** was treated under acidic conditions, 3-sulfonylmethyl 1*H*-indene **8**, which contains the synthetically valuable allylic sulfone moiety,^21^ was readily prepared in an excellent yield. Iodine, or *N*-iodosuccinimide (NIS)-mediated cyclization of allene **4a** occurred smoothly to deliver different indenyl iodide products **9** or **10**. Under palladium catalysis, desulfonylation of allene sulfone **4a** and subsequent isomerization led to the formation of 1,3-diene **11** (Scheme 5A). Our new strategy may potentially be extended to include the utilization of other radical precursors. Using TMEDA as a sacrificial electron donor, the employment of *tert*-butyl bromide led to the formation of the corresponding allene **12** in a good yield (Scheme 5B). We also performed a number of transformations on the 1,3-dienyl sulfone product **7a** (Scheme 5C). We first carried out regioselective epoxidation of **7a**, as epoxy sulfones are known to be valuable in organic synthesis/medicinal chemistry.^22^ Treating **7a** with *m*-CPBA led to the formation of unsaturated β,γ-epoxy sulfone **14** in a good yield, while α,β-epoxy sulfone **15** was obtained when *t*-BuOONa was used as a nucleophilic oxidant. When dienyl sulfone **13** reacted with the Grignard reagent under palladium catalysis, the cross-coupling reaction took place and diene **16** was obtained in 96% yield. Moreover, the two double bonds in 1,3-dienyl sulfones were easily differentiated, and under standard Pd/C conditions, regioselective hydrogenation of **13** formed vinyl sulfone **17** in a moderate yield.

**Fig. 2:**
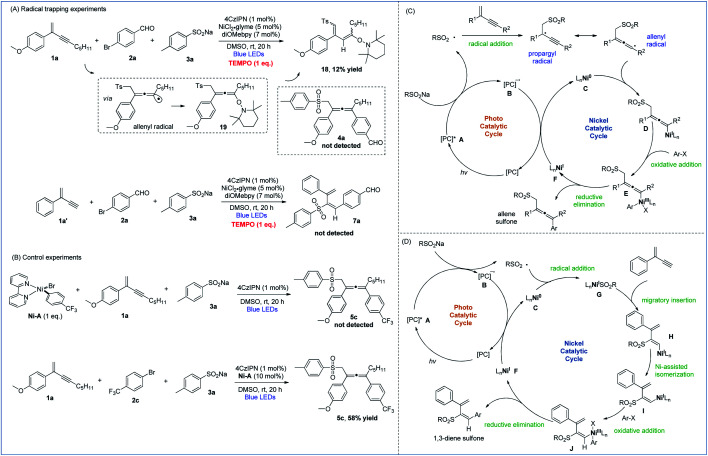
Mechanistic studies. (A) Radical trapping experiments. (B) Control experiments. (C) Proposed mechanism of 1,4-sulfonylarylation. (D) Proposed mechanism of 3,4-sulfonylarylation.

To gain insights into the mechanistic pathways leading to 1,4- and 3,4-sulfonylarylation of 1,3-enynes, we performed a number of experiments. Stern–Volmer quenching experiments of all three starting materials were conducted, which revealed that the excited state of 4CzIPN was only quenched by sodium 4-methylbenzenesulfinate, suggesting the involvement of sulfonyl radical species in the reaction processes (Fig. S1–S3 in the ESI†). The radical trapping experiments were also carried out. With the addition of one molar equivalence of radical scavenger TEMPO, the cross-coupling reaction of enyne **1a**, bromide **2a**, and sulfinate **3a** was inhibited, 1,3-diene **18** isomerized from TEMPO-captured allene **19** was isolated in 12% yield, and the allene product **4a** was not detected. Similarly, the addition of TEMPO to a mixture of 1,3-enyne **1a′**, bromide **2a**, and sulfinate **3a** under standard reaction conditions completely inhibited the radical reaction pathways, and 1,3-dienyl sulfone **7a** was not detected (Fig. 2A). These experiments suggested that both 1,4- and 3,4-difunctionalization reactions of 1,3-enynes most likely proceed *via* radical reaction pathways. Moreover, the isolation of 1,3-diene **18** provided solid evidence to support the existence of allenyl radicals in the 1,4-sulfonylarylation process. The following experiments were performed to understand the role of active nickel species involved in the reaction. When a mixture of 1,3-enyne **1a** and sodium 4-methylbenzenesulfinate **3a** was exposed to a stoichiometric amount of the **Ni-A** complex, the corresponding allene product **5c** was not formed. However, treatment of 1,3-enyne **1a**, bromide **2c**, and sulfinate **3a** in the presence of 10 mol% **Ni-A** led to the formation of desired product **5c** in 58% yield (Fig. 2B). The above results imply that the aryl halide-derived Ar–Ni(ii) complex may not be the reactive species in the catalytic cycle.

On the basis of the above mechanistic investigations, as well as related literature reports,^10*b*,*i*^ mechanisms of regiodivergent 1,4- and 3,4-sulfonylarylation of 1,3-enynes are proposed. In the photocatalytic cycle, excitation of photocatalyst (PC) 4CzIPN (*E*_1/2_ [*PC/PC^−^ = + 1.35 V *versus* SCE in CH_3_CN])^23^ leads to its active state **A**, which then oxidizes sodium sulfinate (TsNa, *E*_1/2_ = + 0.45 V *versus* SCE in CH_3_CN)^24^ to generate the sulfonyl radical along with the reduced form **B** of the PC. When 1,3-enynes with a terminal substituent (R^2^) are used, the addition of the sulfonyl radical to the double bond of 1,3-enyne creates a propargyl radical, the resonance form of which is a relatively well-stabilized allenyl radical species. The interception of the allenyl radical by Ni^0^ species **C** then forms Ni^I^ intermediate **D**. Subsequently, oxidative addition of aryl halide to **D** furnishes Ni^III^ species **E**, which undergoes reductive elimination to yield the 1,4-sulfonylarylated allene product and Ni^I^ species **F**. Finally, single-electron-transfer (SET) with PC reduced form **B** then returns the Ni^I^ species to the Ni^0^ catalyst and regenerates ground-state 4CzIPN (Fig. 2C). On the other hand, when terminal 1,3-enynes are used (R^2^ = H), compared with pathways of generating propargyl/allenyl radical species, the trapping of the sulfonyl radical by Ni^0^ specie **C** becomes more favorable. The sulfonyl Ni^I^ species **G** undergoes 1,2-migratory insertion into the triple bond of 1,3-enyne to generate intermediate **H**, which transforms to the intermediate **I***via* Ni-assisted *syn*/*anti* isomerization. The subsequent oxidative addition of aryl halide produces Ni^III^ species **J**, which readily reductively eliminates to deliver the 3,4-sulfonylarylated 1,3-diene product, and the resulting Ni^I^ returns to the Ni^0^ catalyst in a similar fashion as described in the 1,4-addition pathway (Fig. 2D).

## Conclusions

In conclusion, the merger of nickel and photoredox catalysis enables the successful development of a practical catalytic strategy for regiodivergent sulfonylarylation of 1,3-enynes. By utilizing 1,3-enynes with subtle structural variation, efficient synthesis of α-allenyl sulfones or 1,3-dienyl sulfones was accomplished, *via* 1,4-sulfonylarylation or 3,4-sulfonylarylation of 1,3-enynes, respectively. The reactions proceeded with excellent chemo-, regio- and stereo-selectivities, mild conditions, broad substrate scopes and wide functional group tolerance. Moreover, the protocols described herein could be readily scaled-up, and the products were shown to be versatile in organic synthesis. Preliminary mechanistic investigations suggest the involvement of radical species in the difunctionalization of 1,3-enynes. We anticipate that our approach will stimulate more interest in multi-functionalization of 1,3-enynes and related compounds *via* photoredox and metal dual catalysis pathways.

## Data availability

All experimental procedures, characterization, copies of NMR spectra for all new compounds related to this article can be found in the ESI.†

## Author contributions

Y. C. and K. Z. designed and carried out the experiments, and prepared the ESI† Q. H. participated in the synthesis of partial substrates. Y. C. and Y. L. initiated the project and wrote the paper. Y. L supervised the project overall. All authors discussed the results and commented on the manuscript.

## Conflicts of interest

There are no conflicts to declare.

## Supplementary Material

SC-012-D1SC04320J-s001

SC-012-D1SC04320J-s002
