# Supplementary material for: Regiodivergent sulfonylarylation of 1,3-enynes via nickel/photoredox dual catalysis
Source: Chem Sci. 2021 Sep 20;12(40):13564–71. doi: 10.1039/d1sc04320j (PMC8528021; doi:10.1039/d1sc04320j)
Supplement: SC-012-D1SC04320J-s001 [file SC-012-D1SC04320J-s001.pdf]

Electronic Supplementary Material (ESI) for Chemical Science.
This journal is © The Royal Society of Chemistry 2021

Supporting Information for

Regiodivergent Sulfonylarylation of 1,3-Enynes via Nickel/Photoredox Dual Catalysis

Ya Chen,[1] Kun Zhu,[1,2] Qingqin Huang[1,2] and Yixin Lu*,[1,2]

[1] Department of Chemistry, National University of Singapore, 3 Science Drive 3, Singapore, 117543

[2] Joint School of National University of Singapore and Tianjin University, International Campus of Tianjin University, Binhai New City, Fuzhou, Fujian, PR China, 350207

E-mail: chmlyx@nus.edu.sg

Contents

1. General information --S2
2. Optimization of reaction conditions ---S3
3. General procedure for the synthesis of 1,3-enynes -----------------------------------S7
4. General procedure for 1,4-sulfonylarylation of 1,3-enynes------------------------S14
5. General procedure for 3,4-sulfonylarylation of 1,3-enynes------------------------S37
6. Scale-up syntheses and further transformations----------------------------------S47
7. Mechanistic experiments ---S57
8. X-ray crystallography data --S64
9. References --S68
10. Copy of NMR spectra--S69

1. General information

Unless otherwise noted, all experiments were carried out under an atmosphere of nitrogen and anhydrous conditions. ^{1}H NMR and ^{13}C NMR spectra were recorded on Bruker AMX 400 Spectrometer (^{1}H 400 MHz and ^{13}C 100 MHz, respectively) and Bruker AMX 500 Spectrometer (^{1}H 500 MHz and ^{13}C 125 MHz, respectively). Chemical shifts (δ) were given in ppm and were referenced to residual solvent or TMS peaks. All high resolution mass spectra were obtained on a Bruker micrOTOFQ II (ESI). All the solvents were purified according to the standard procedures. Substrates of sodium sulfinates were synthesized from the corresponding sulfonyl chlorides according to the previous report.[1] All other chemicals which are commercially available were employed without further purification.

2. Optimization of reaction conditions

2.1 1,4-Sulfonylarylation of 1,3-enynes

Table S1. Screening of the ratios of the starting materials and solvents [a]

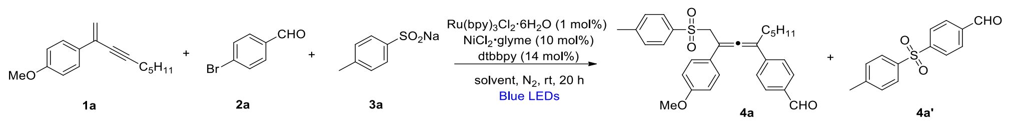

Entry	**1a** (equiv)	**2a** (equiv)	**3a** (equiv)	Solvent	**4a**: **4a'**[b]	Yield of **4a** (%)[c]
1	1.0	1.0	1.2	DMSO	9: 1	37
2	1.0	2.0	1.2	DMSO	1.4: 1	43
3	1.5	1.0	1.2	DMSO	18: 1	42
**4**	**2.0**	**1.0**	**1.2**	**DMSO**	**>20: 1**	**55**
5	2.0	1.0	2.0	DMSO	5: 1	40
6	2.0	1.0	1.2	DMA	>20: 1	10
7	2.0	1.0	1.2	DMF	>20: 1	39
8	2.0	1.0	1.2	NMP	---	0
9	2.0	1.0	1.2	MeCN	---	0
10	2.0	1.0	1.2	MeOH	---	0
11	2.0	1.0	1.2	THF	---	0

[a] Reaction conditions: $Ru(bpy)_3Cl_2 \cdot 6H_2O$ (1 mol%), $NiCl_2$·glyme (10 mol%), dtbbpy (14 mol%), solvent (0.1 M), at room temperature, 30 W blue LEDs, 20 h. [b] Determined by ^1H NMR analysis of crude product. [c] Isolated yield.

Table S2. Screening of photocatalysts, nickel sources and ligands [a]

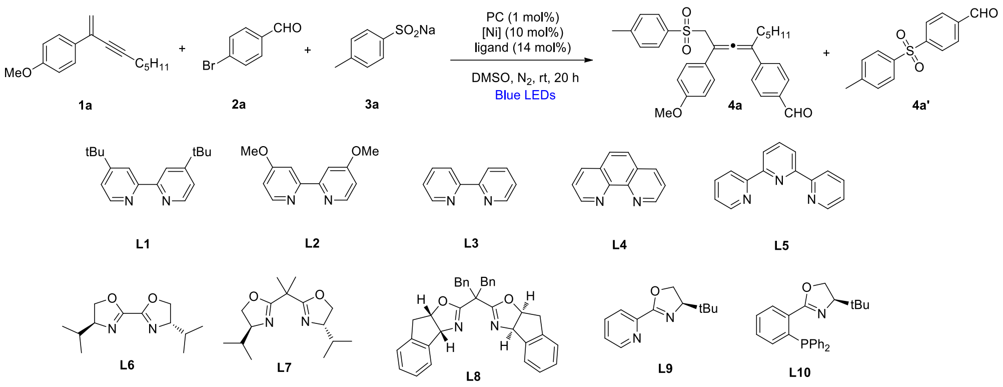

Entry	PC	[Ni]	Ligand	**4a**: **4a'**[b]	Yield of **4a** (%)[c]
1	$Ru(bpy)_3Cl_2·6H_2O$	$NiCl_2$·glyme	**L1**	>20: 1	55
2	$Ru(bpy)_3(PF_6)_2$	$NiCl_2$·glyme	**L1**	>20: 1	45
3	$[Ir(dFCF_3ppy)_2(dtbbpy)]PF_6$	$NiCl_2$·glyme	**L1**	>20: 1	62
4	4CzIPN	$NiCl_2$·glyme	**L1**	>20: 1	64
5	4CzIPN	$NiCl_2$	**L1**	>20: 1	35
6	4CzIPN	$NiCl_2·6H_2O$	**L1**	>20: 1	11
7	4CzIPN	$NiCl_2$·glyme	**L2**	>20: 1	73
8	4CzIPN	$NiCl_2$·glyme	**L3**	>20: 1	62
9	4CzIPN	$NiCl_2$·glyme	**L4**	---	0
10	4CzIPN	$NiCl_2$·glyme	**L5**	---	0
11	4CzIPN	$NiCl_2$·glyme	**L6**	---	0
12	4CzIPN	$NiCl_2$·glyme	**L7**	7: 1	30
13	4CzIPN	$NiCl_2$·glyme	**L8**	---	trace
14	4CzIPN	$NiCl_2$·glyme	**L9**	5: 1	14
15	4CzIPN	$NiCl_2$·glyme	**L10**	1: 1	12
**16[d]**	**4CzIPN**	**$NiCl_2$·glyme**	**L2**	**>20: 1**	**72**

[a] Reaction conditions: **1a** (0.2 mmol), **2a** (0.1 mmol) and **3a** (0.12 mmol) in 1.0 mL DMSO, PC (1 mol%), [Ni] (10 mol%), ligand (14 mol%), at room temperature, 30 W blue LEDs, 20 h. [b] Determined by ^1H NMR analysis of crude product. [c] Isolated yield. [d] 5 mol% $NiCl_2$·glyme, 7 mol% diOMebpy.

Table S3. Other screening and control experiments [a]

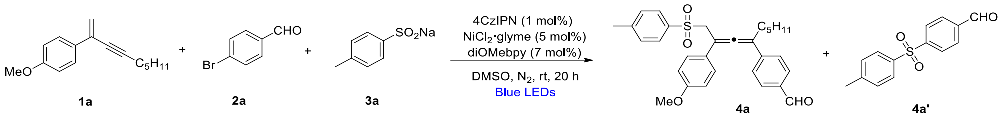

Entry	Deviations from the reaction conditions	**4a**: **4a'**[b]	Yield of **4a** (%)[c]
1	None	>20: 1	72
2	No photocatalyst	---	0
3	No light	---	0
4	No [Ni]	---	0
5	No ligand	---	0
6	In air	---	0
7	Addition of 10 uL H_2O	>20: 1	54

[a] Reaction conditions: **1a** (0.2 mmol), **2a** (0.1 mmol) and **3a** (0.12 mmol) in 1.0 mL DMSO, 4CzIPN (1 mol%), $NiCl_2$·glyme (5 mol%), diOMebpy (7 mol%), at room temperature, 30 W blue LEDs, 20 h. [b] Determined by ^1H NMR analysis of crude product. [c] Isolated yield.

2.2 3,4-Sulfonylarylation of 1,3-enynes

Table S4. Screening of the reaction conditions [a]

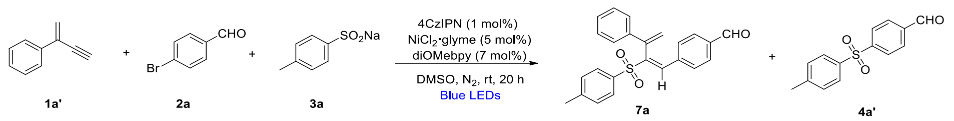

Entry	Ratio (**1a'**/**2a**/**3a**)	Ligand	PC (EnT energy (kcal/mol))	**7a**: **4a'**[b]	Yield of **7a**(%)[c]	*E*/*Z* of **7a**(%)[b]
1	1:1:1.2	**L2**	4CzIPN	5: 1	49	>20: 1
**2**	**2:1:1.2**	**L2**	**4CzIPN**	**5: 1**	**70**	**>20: 1**
3	3:1:1.2	**L2**	4CzIPN	5: 1	18	>20: 1
4	2:1:1.2	**L1**	4CzIPN	10: 1	47	>20: 1
5	2:1:1.2	**L4**	4CzIPN	5: 1	50	>20: 1
6	2:1:1.2	**L5**	4CzIPN	---	0	---
7	2:1:1.2	**L2**	$Ru(bpy)_3Cl_2·6H_2O$ (46.5)	5: 1	62	>20: 1
8	2:1:1.2	**L2**	$Ru(bpy)_3(PF_6)_2$ (46.8)	7: 1	61	>20: 1
9	2:1:1.2	**L2**	$Ir(ppy)_3$ (55.2)	3: 1	50	>20: 1
10	2:1:1.2	**L2**	$[Ir(dFCF_3ppy)_2(dtbbpy)]PF_6$ (59.4)	5: 1	56	7: 1
11	2:1:1.2	**L2**	$Ir(dFppy)_3$ (60.1)	4: 1	52	1.5: 1

[a] Reaction conditions: **1a'** (0.2 mmol), **2a** (0.1 mmol) and **3a** (0.12 mmol) in 1.0 mL DMSO, 4CzIPN (1 mol%), $NiCl_2$·glyme (5 mol%), diOMebpy (7 mol%), at room temperature, 30 W blue LEDs, 20 h. [b] Determined by ^1H NMR analysis of crude product. [c] Isolated yield.

Table S5. Control experiments [a]

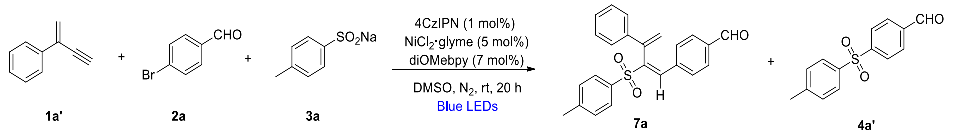

Entry	Deviations from the reaction	**7a**: **4a'**[b]	Yield of **7a** (%)[c]	*E*/*Z* of **7a** (%)[b]
1	None	5: 1	70	>20: 1
2	No photocatalyst	---	0	
3	No light	---	0	
4	No [Ni]	---	0	
5	No ligand	---	0	
6	In air	---	0	

[a] Reaction conditions: **1a'** (0.2 mmol), **2a** (0.1 mmol) and **3a** (0.12 mmol) in 1.0 mL DMSO, 4CzIPN (1 mol%), $NiCl_2$·glyme (5 mol%), diOMebpy (7 mol%), at room temperature, 30 W blue LEDs, 20 h. [b] Determined by ^{1}H NMR analysis of crude product. [c] Isolated yield.

3. General procedure for the synthesis of 1,3-enynes[2]

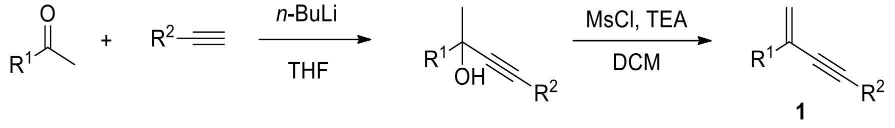

Procedure A: Under nitrogen atmosphere, *n*-BuLi (2.0 M in hexane, 5 mmol, 2.5 mL) was added dropwise to a solution of alkyne (5 mmol) in anhydrous THF (20 mL) at -78 °C. After addition, the resulting solution was stirred at room temperature for one hour. Then, cooled to -78 °C again, ketone (5 mmol) in THF (10 mL) was added dropwise. The reaction mixture was allowed to warm to room temperature and was monitored by TLC for completion. Once completion the reaction was quenched with saturated aqueous NH_4Cl and extracted with EtOAc three times. The combined organic layer was dried over Na_2SO_4 and concentrated under reduced pressure to afford the crude propargyl alcohol.

The resulting propargyl alcohol was dissolved in DCM (30 mL), and the mixture was cooled to 0 °C. TEA (25 mmol, 5 equiv) was added to this solution and methylsulfonyl chloride (12.5 mmol, 2.5 equiv) sequentially. After one hour the reaction was monitored by TLC for completion. Once completion the reaction was quenched with saturated aqueous NH_4Cl. The aqueous layer was extracted with DCM and the combined organic layers were washed with brine, dried over Na_2SO_4, filtered, and concentrated under reduced pressure. The crude material was purified by flash chromatography to yield the 1,3-enyne **1**.

The analytical data of the new products are summarized below.

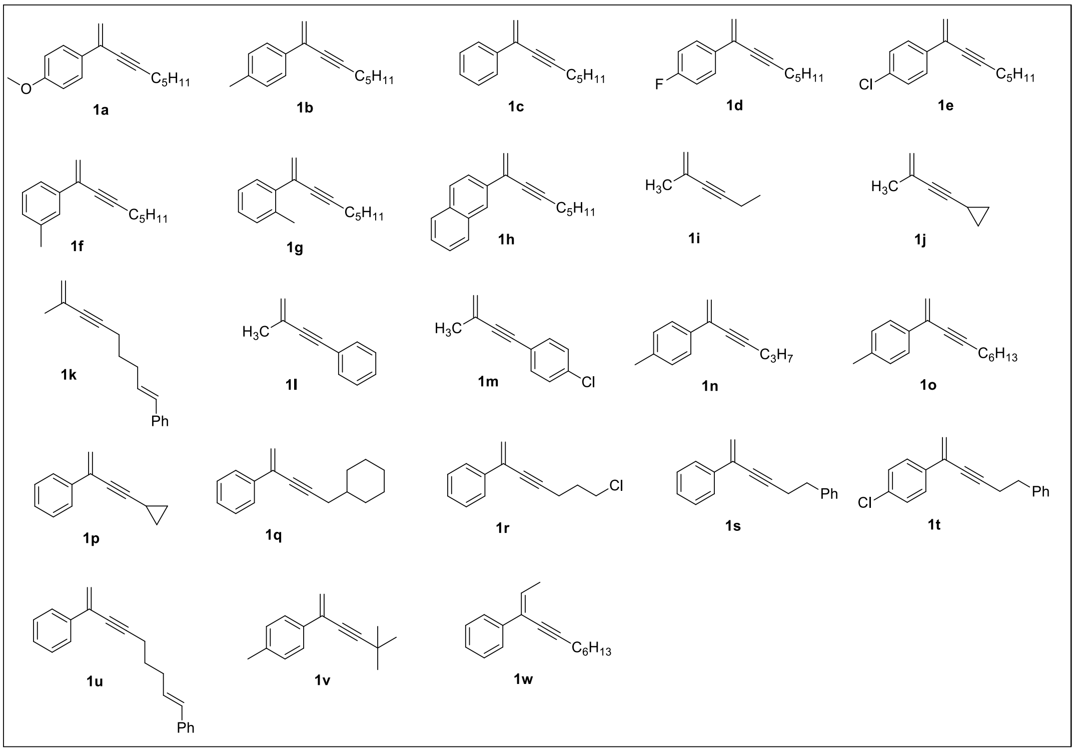

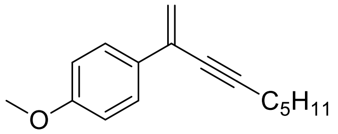

1a

1-methoxy-4-(non-1-en-3-yn-2-yl)benzene (1a): Pale yellow oil, isolated yield 50%. ^{1}H NMR (400 MHz, $CDCl_3$) δ 7.52 (d, *J* = 9.2 Hz, 2H), 6.79 (d, *J* = 8.8 Hz, 2H), 5.65 (d, *J* = 1.2 Hz, 1H), 5.40 (d, *J* = 1.2 Hz, 1H), 3.73 (s, 3H), 2.32 (t, *J* = 7.0 Hz, 2H), 1.56 – 1.49 (m, 2H), 1.40 – 1.25 (m, 4H), 0.85 (t, *J* = 7.2 Hz, 3H). ^{13}C NMR (100 MHz, $CDCl_3$) δ 159.6, 130.5, 130.3, 127.3, 117.4, 113.6, 91.8, 79.9, 55.3, 31.6, 28.5, 22.2, 19.4, 14.0. MS (ESI): 229.2 $[M+H]^+$.

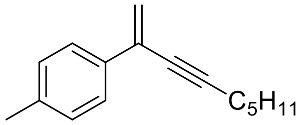

1b

1-methyl-4-(non-1-en-3-yn-2-yl)benzene (1b): Pale yellow oil, isolated yield 50%. ^{1}H NMR (400 MHz, $CDCl_3$) δ 7.56 (d, *J* = 8.0 Hz, 2H), 7.17-7.15 (m, 2H), 5.81 (d, *J* = 1.2 Hz, 1H), 5.54 (d, *J* = 1.2 Hz, 1H), 2.42 (t, *J* = 7.2 Hz, 2H), 2.37 (s, 3H), 1.65 – 1.56 (m, 2H), 1.49 – 1.34 (m, 4H), 0.94 (t, *J* = 7.2 Hz, 3H). ^{13}C NMR (100 MHz, $CDCl_3$) δ 137.9, 135.0, 130.8, 128.9, 125.9, 118.4, 91.8, 79.9, 31.2, 28.5, 22.2, 21.1, 19.4, 14.0. MS (ESI): 213.2 $[M+H]^+$.

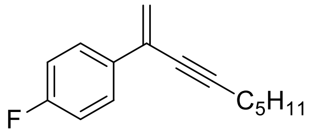

1d

1-fluoro-4-(non-1-en-3-yn-2-yl)benzene (1d): Pale yellow oil, isolated yield 59%. ^{1}H NMR (400 MHz, $CDCl_3$) δ 7.64 – 7.61 (m, 2H), 7.05 – 7.00 (m, 2H), 5.77 (d, J = 1.2 Hz, 1H), 5.56 – 5.55 (m, 1H), 2.41 (t, J = 7.0 Hz, 2H), 1.64 – 1.58 (m, 2H), 1.46 – 1.33 (m, 4H), 0.93 (t, J = 7.0 Hz, 3H). ^{13}C NMR (100 MHz, $CDCl_3$) δ 162.2 (d, J = 246.0 Hz), 133.9 (d, J = 3.0 Hz), 129.9, 127.8 (d, J = 8.0 Hz), 119.0 (d, J = 1.0 Hz), 115.1 (d, J = 22.0 Hz), 92.3, 79.6, 31.2, 28.4, 22.2, 19.4, 14.0. MS (ESI): 217.1 $[M+H]^+$.

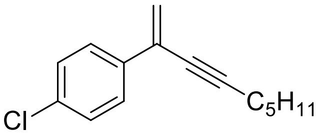

1e

1-chloro-4-(non-1-en-3-yn-2-yl)benzene (1e): Pale yellow oil, isolated yield 68%. ^{1}H NMR (400 MHz, $CDCl_3$) δ 7.54 (d, J = 8.4 Hz, 2H), 7.27 (d, J = 8.4 Hz, 2H), 5.77 (d, J = 0.8 Hz, 1H), 5.55 (d, J = 0.8 Hz, 1H), 2.37 (t, J = 7.2 Hz, 2H), 1.59 – 1.52 (m, 2H), 1.43 – 1.29 (m, 4H), 0.89 (t, J = 7.2 Hz, 3H). ^{13}C NMR (100 MHz, $CDCl_3$) δ 136.3, 133.9, 129.9, 128.4, 127.4, 119.0, 92.5, 79.3, 31.2, 28.4, 22.2, 19.3, 14.0. MS (ESI): 233.1 $[M+H]^+$.

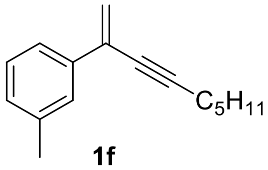

1f

1-methyl-3-(non-1-en-3-yn-2-yl)benzene (1f): Pale yellow oil, isolated yield 55%. ^{1}H NMR (500 MHz, $CDCl_3$) δ 7.51 – 7.48 (m, 2H), 7.28 – 7.24 (m, 1H), 7.15 – 7.13 (m, 1H), 5.85 (d, J = 1.5 Hz, 1H), 5.59 (d, J = 1.0 Hz, 1H), 2.44 (t, J = 7.0 Hz, 2H), 2.40 (s, 3H), 1.68 – 1.62 (m, 2H), 1.51 – 1.45 (m, 2H), 1.42 – 1.37 (m, 2H), 0.96 (t, J = 7.3 Hz, 3H). ^{13}C NMR (125 MHz, $CDCl_3$) δ 137.8, 131.1, 128.8, 128.1, 126.8, 123.2, 119.2, 91.9, 79.9, 31.2, 28.4, 22.2, 21.4, 19.4, 14.0. MS (ESI): 213.2 $[M+H]^+$.

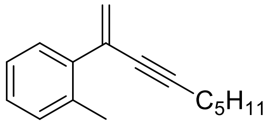

1g

1-methyl-2-(non-1-en-3-yn-2-yl)benzene (1g): Pale yellow oil, isolated yield 46%. ^{1}H NMR (400 MHz, $CDCl_3$) δ 7.29-7.26 (m, 1H), 7.23-7.17 (m, 3H), 5.70 (s, 1H), 5.40 (s, 1H),

2.46-2.45 (m, 3H), 2.37-2.32 (m, 2H), 1.59 – 1.53 (m, 2H), 1.42 – 1.31 (m, 4H), 0.95 – 0.90 (m, 3H). ^{13}C NMR (100 MHz, $CDCl_3$) δ 140.0, 135.4, 132.2, 130.26, 128.6, 127.6, 125.8, 123.8, 91.9, 80.6, 31.1, 28.3, 22.2, 20.2, 19.4, 14.0. MS (ESI): 213.2 $[M+H]^+$.

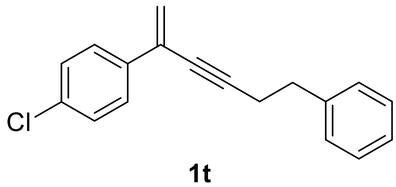

1t

1-chloro-4-(6-phenylhex-1-en-3-yn-2-yl)benzene (1t): Pale yellow oil, isolated yield 51%. ^{1}H NMR (400 MHz, $CDCl_3$) δ 7.36 (d, *J* = 8.8 Hz, 2H), 7.28 – 7.21 (m, 2H), 7.21 – 7.08 (m, 5H), 5.72 (d, *J* = 1.2 Hz, 1H), 5.48 (d, *J* = 0.8 Hz, 1H), 2.84 (t, *J* = 7.6 Hz, 2H), 2.64 (t, *J* = 7.6 Hz, 2H). ^{13}C NMR (100 MHz, $CDCl_3$) δ 140.5, 136.0, 133.9, 129.7, 128.5, 128.4, 128.3, 127.4, 126.4, 119.8, 91.4, 80.1, 34.9, 21.5. MS (ESI): 267.1 $[M+H]^+$.

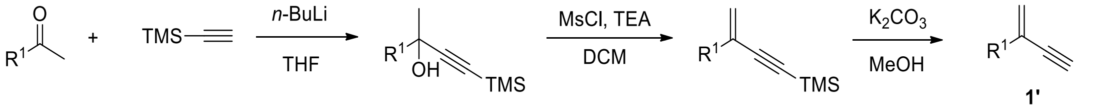

Procedure B: Under nitrogen atmosphere, *n*-BuLi (2.0 M in hexane, 5 mmol, 2.5 mL) was added dropwise to a solution of trimethylsilylacetylene (5 mmol) in anhydrous THF (20 mL) at -78 °C. After addition, the resulting solution was stirred at room temperature for one hour. Then, cooled to -78 °C again, ketone (5 mmol) in THF (10 mL) was added dropwise. The reaction mixture was allowed to warm to room temperature and was monitored by TLC for completion. Once completion the reaction was quenched with saturated aqueous NH_4Cl and extracted with EtOAc three times. The combined organic layer was dried over Na_2SO_4 and concentrated under reduced pressure to afford the crude propargyl alcohol.

The resulting propargyl alcohol was dissolved in DCM (30 mL), and the mixture was cooled to 0 °C. TEA (25 mmol, 5 equiv) was added to this solution and methylsulfonyl chloride (12.5 mmol, 2.5 equiv) sequentially. After one hour the reaction was monitored by TLC for completion. Once completion the reaction was quenched with saturated aqueous NH_4Cl. The aqueous layer was extracted with DCM and the combined organic layers were washed with brine, dried over Na_2SO_4, filtered,

and concentrated under reduced pressure.

The crude material was directly treated with anhydrous K_2CO_3 (15 mmol, 3 equiv) in MeOH (20 mL) and stirred at room temperature for two hours. Then, MeOH was removed and water was added to the remaining residue. The aqueous layer was extracted with EtOAc and the combined organic layers were washed with brine, dried over Na_2SO_4, filtered, and concentrated under reduced pressure. The crude residue was purified by flash chromatography to yield the 1,3-enyne **1'**.

The analytical data of the new products are summarized below.

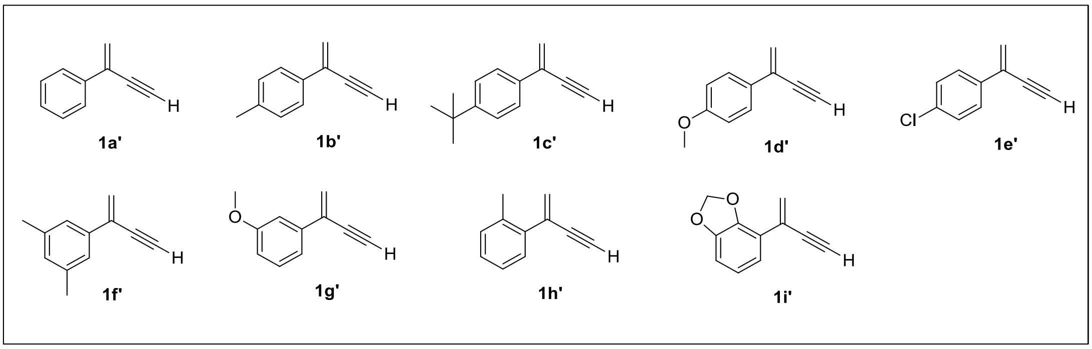

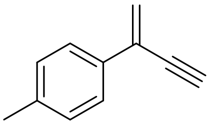

1b'

1-(but-1-en-3-yn-2-yl)-4-methylbenzene (1b'): Pale yellow oil, isolated yield 59%. ^{1}H NMR (400 MHz, $CDCl_3$) δ 7.57 (d, *J* = 8.0 Hz, 2H), 7.21 – 7.14 (m, 2H), 5.96 (s, 1H), 5.72 (s, 1H), 3.11 (t, *J* = 0.6 Hz, 1H), 2.37 (s, 3H). ^{13}C NMR (100 MHz, $CDCl_3$) δ 138.4, 133.9, 129.5, 129.1, 125.9, 121.3, 82.9, 78.4, 21.2. MS (ESI): 143.1 $[M+H]^+$.

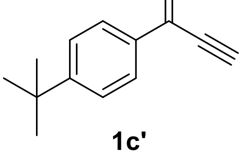

1c'

1-(but-1-en-3-yn-2-yl)-4-(tert-butyl)benzene (1c'): Pale yellow oil, isolated yield 57%. ^{1}H NMR (400 MHz, $CDCl_3$) δ 7.62 (d, *J* = 8.8 Hz, 2H), 7.41 (d, *J* = 8.8 Hz, 2H), 5.99 – 5.96 (m, 1H), 5.75 – 5.71 (m, 1H), 3.11 (t, *J* = 0.6 Hz, 1H), 1.35 (s, 9H). ^{13}C NMR (100 MHz, $CDCl_3$) δ 151.6, 133.9, 129.5, 125.7, 125.3, 121.4, 82.9, 78.4, 34.6, 31.3. MS (ESI): 185.1 $[M+H]^+$.

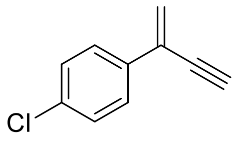

1e'

1-(but-1-en-3-yn-2-yl)-4-chlorobenzene (1e'): Pale yellow oil, isolated yield 55%. ^{1}H NMR (400 MHz, $CDCl_3$) δ 7.59 (d, *J* = 8.8 Hz, 2H), 7.33 (d, *J* = 8.8 Hz, 2H), 5.97 (s, 1H), 5.77 (s, 1H), 3.13 (t, *J* = 0.6 Hz, 1H). ^{13}C NMR (100 MHz, $CDCl_3$) δ 135.1, 134.4, 128.7, 128.5, 127.3, 122.5, 82.3, 79.1. MS (ESI): 163.0 $[M+H]^+$.

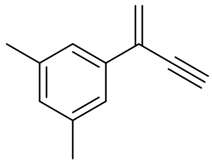

1f'

1-(but-1-en-3-yn-2-yl)-3,5-dimethylbenzene (1f'): Pale yellow oil, isolated yield 54%. ^{1}H NMR (400 MHz, $CDCl_3$) δ 7.34 – 7.30 (m, 2H), 7.02 – 6.98 (m, 1H), 6.00 (s, 1H), 5.76 (s, 1H), 3.14 (t, *J* = 0.4 Hz, 1H), 2.38 (d, *J* = 0.8 Hz, 6H). ^{13}C NMR (100 MHz, $CDCl_3$) δ 137.9, 136.6, 130.1, 129.9, 123.8, 122.0, 83.0, 78.4, 21.3. MS (ESI): 157.1 $[M+H]^+$.

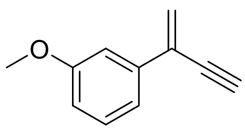

1g'

1-(but-1-en-3-yn-2-yl)-3-methoxybenzene (1g'): Pale yellow oil, isolated yield 58%. ^{1}H NMR (400 MHz, $CDCl_3$) δ 7.37 – 7.17 (m, 3H), 6.93 – 6.86 (m, 1H), 6.02 (s, 1H), 5.79 (s, 1H), 3.86 (s, 3H), 3.14 (t, *J* = 0.6 Hz, 1H). ^{13}C NMR (100 MHz, $CDCl_3$) δ 159.6, 138.1, 129.6, 129.4, 122.5, 118.4, 113.9, 111.9, 82.7, 78.6, 55.3. MS (ESI): 159.1 $[M+H]^+$.

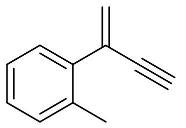

1h'

1-(but-1-en-3-yn-2-yl)-2-methylbenzene (1h'): Pale yellow oil, isolated yield 54%. ^{1}H NMR (400 MHz, $CDCl_3$) δ 7.25 – 6.92 (m, 4H), 5.75 (dd, *J* = 1.6, 0.8 Hz, 1H), 5.42 (dd, *J* = 1.6, 0.8 Hz, 1H), 2.93 (t, *J* = 0.6 Hz, 1H), 2.31 (s, 3H). ^{13}C NMR (100 MHz, $CDCl_3$) δ 138.7, 135.5, 130.9, 130.4, 128.7, 128.0, 126.9, 125.9, 83.4, 78.5, 20.1. MS (ESI): 143.1 $[M+H]^+$.

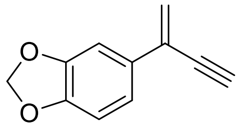

1i'

5-(but-1-en-3-yn-2-yl)benzo[d][1,3]dioxole (1i'): Pale yellow oil, isolated yield 53%. ^{1}H NMR (400 MHz, $CDCl_3$) δ 7.20 (dd, *J* = 8.2, 1.8 Hz, 1H), 7.14 (dd, *J* = 1.8, 0.6 Hz, 1H), 6.80 (dd, *J* = 8.4, 0.4 Hz, 1H), 5.97 (s, 2H), 5.85 (s, 1H), 5.67 (s, 1H), 3.11 (s, 1H).

^{13}C NMR (100 MHz, $CDCl_3$) δ 147.9, 147.8, 131.0, 129.1, 120.7, 120.3, 108.0, 106.1, 101.2, 82.8, 78.5. MS (ESI): 173.1 $[M+H]^+$.

4. General procedure for 1,4-sulfonylarylation of 1,3-enynes

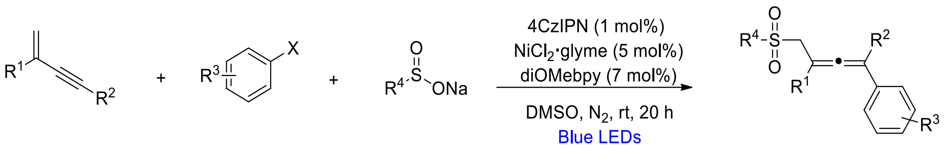

General procedure : A dry reaction tube equipped with a Teflon-coated magnetic stir bar was charged with 4CzIPN (1.6 mg, 0.002 mmol, 1 mol%), $NiCl_2$·glyme (2.2 mg, 0.01 mmol, 5 mol%), diOMebpy (3.0 mg, 0.014 mmol, 7 mol%), aryl halide (0.2 mmol, 1 equiv., if solid), sodium sulfinate (0.24 mmol, 1.2 equiv.) and 1,3-enyne (0.4 mmol, 2 equiv., if solid). It was capped with a rubber septum, evacuated and backfilled with argon. Then, degassed anhydrous DMSO (2.0 mL), aryl halide (0.2 mmol, 1 equiv., if liquid) and 1,3-enyne (0.4 mmol, 2 equiv., if liquid) were added via syringe. The reaction mixture was stirred at room temperature under 30 W blue LEDs irradiation for 20 hours. After the reaction completion, the reaction mixture was diluted with EtOAc, then washed with brine, dried over Na_2SO_4, filtered, and concentrated under reduced pressure. The residue was purified by column chromatography on silica gel (hexane/EtOAc) to afford the corresponding products.

The analytical data of the products are summarized below. **4m** is known compound in the references.[3]

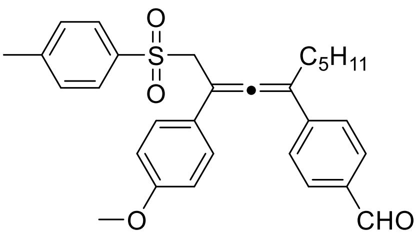

4a

4-(2-(4-methoxyphenyl)-1-tosylnona-2,3-dien-4-yl)benzaldehyde (4a): Pale yellow oil, 70.3 mg, isolated yield 72%. ^{1}H NMR (400 MHz, $CDCl_3$) δ 9.98 (s, 1H), 7.79 (d, *J* = 8.8 Hz, 2H), 7.69 (d, *J* = 8.0 Hz, 2H), 7.47 (d, *J* = 8.4 Hz, 2H), 7.26 (d, *J* = 8.8 Hz, 2H), 7.18 (d, *J* = 8.0 Hz, 2H), 6.81 (d, *J* = 8.8 Hz, 2H), 4.36 – 4.26 (m, 2H), 3.79 (s, 3H), 2.49 – 2.39 (m, 2H), 2.36 (s, 3H), 1.53 – 1.44 (m, 2H), 1.35 – 1.26 (m, 4H), 0.85 (t, *J* = 6.8 Hz, 3H). ^{13}C NMR (100 MHz, $CDCl_3$) δ 210.3, 191.7, 159.2, 144.6, 142.1, 136.1, 135.2, 129.9, 129.6, 128.4, 127.4, 126.9, 125.8, 114.1, 109.8, 99.3, 58.2,

55.3, 31.7, 30.3, 27.6, 22.4, 21.6, 14.0. HRMS (ESI): m/z calcd. for $C_{33}H_{33}O_4S^+$([M+H]$^+$) = 489.2094, found = 489.2095.

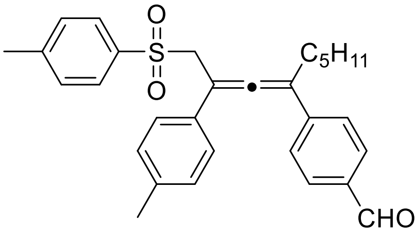

4b

4-(2-(*p*-tolyl)-1-tosylnona-2,3-dien-4-yl)benzaldehyde (4b): Light yellow solid, 70.8 mg, isolated yield 75%. ^{1}H NMR (400 MHz, $CDCl_3$) δ 9.98 (s, 1H), 7.79 (d, *J* = 8.4 Hz, 2H), 7.69 (d, *J* = 8.4 Hz, 2H), 7.48 (d, *J* = 8.4 Hz, 2H), 7.22 (d, *J* = 8.4 Hz, 2H), 7.17 (d, *J* = 8.4 Hz, 2H), 7.08 (d, *J* = 8.0 Hz, 2H), 4.40 – 4.25 (m, 2H), 2.52 – 2.38 (m, 2H), 2.35 (s, 3H), 2.32 (s, 3H), 1.56 – 1.43 (m, 2H), 1.35 – 1.26 (m, 4H), 0.85 (t, *J* = 7.2 Hz, 3H). ^{13}C NMR (100 MHz, $CDCl_3$) δ 210.5, 191.6, 144.6, 142.0, 137.6, 136.1, 135.2, 130.7, 129.9, 129.6, 129.3, 128.4, 126.9, 126.1, 109.9, 99.6, 58.1, 31.7, 30.2, 27.5, 22.4, 21.6, 21.1, 14.0. HRMS (ESI): m/z calcd. for $C_{30}H_{32}O_3SNa^+$([M+Na]$^+$) = 495.1964, found = 495.1962.

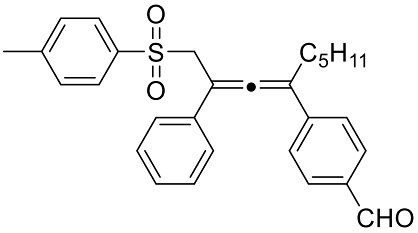

4c

4-(2-phenyl-1-tosylnona-2,3-dien-4-yl)benzaldehyde (4c): Pale yellow oil, 64.1 mg, isolated yield 70%. ^{1}H NMR (400 MHz, $CDCl_3$) δ 9.91 (s, 1H), 7.74 (d, *J* = 8.8 Hz, 2H), 7.63 (d, *J* = 8.4 Hz, 2H), 7.44 (d, *J* = 8.4 Hz, 2H), 7.29 – 7.24 (m, 2H), 7.23 – 7.13 (m, 3H), 7.10 (d, *J* = 7.6 Hz, 2H), 4.34 – 4.23 (m, 2H), 2.48 – 2.33 (m, 2H), 2.28 (s, 3H), 1.52 – 1.37 (m, 2H), 1.30 – 1.20 (m, 4H), 0.78 (t, *J* = 7.8 Hz, 3H). ^{13}C NMR (100 MHz, $CDCl_3$) δ 210.8, 191.6, 144.6, 141.7, 136.0, 135.2, 133.7, 129.9, 129.6, 128.6, 128.3, 127.5, 126.9, 126.1, 110.0, 99.6, 58.0, 31.7, 30.2, 27.5, 22.4, 21.5, 14.0. HRMS (ESI): m/z calcd. for $C_{29}H_{31}O_3S^+$([M+H]$^+$) = 459.1988, found = 459.1987.

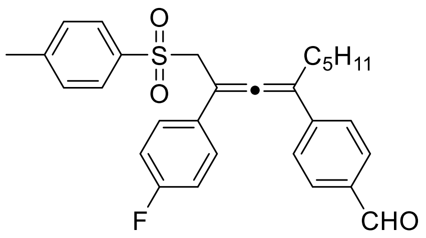

4d

4-(2-(4-fluorophenyl)-1-tosylnona-2,3-dien-4-yl)benzaldehyde (4d): Pale yellow oil, 72.0 mg, isolated yield 76%. ^{1}H NMR (400 MHz, $CDCl_3$) δ 9.91 (s, 1H), 7.73 (d, *J* = 8.4 Hz, 2H), 7.62 (d, *J* = 8.4 Hz, 2H), 7.41 (d, *J* = 8.0 Hz, 2H), 7.22 (dd, *J* = 8.8, 5.2 Hz, 2H), 7.12 (d, *J* = 7.6 Hz, 2H), 6.88 (t, *J* = 8.8 Hz, 2H), 4.29 – 4.18 (m, 2H), 2.47 – 2.33 (m, 2H), 2.29 (s, 3H), 1.49 – 1.36 (m, 2H), 1.27 – 1.19 (m, 4H), 0.78 (t, *J* = 6.8 Hz, 3H). ^{13}C NMR (100 MHz, $CDCl_3$) δ 210.6 (d, *J* = 2.0 Hz), 191.6, 162.2 (d, *J* = 247.0 Hz), 144.8, 141.6, 136.0, 135.3, 129.9, 129.8 (d, *J* = 3.0 Hz), 129.7, 128.4, 127.9 (d, *J* = 8.0 Hz), 127.0, 115.6 (d, *J* = 21.0 Hz), 110.1, 98.9, 58.3, 31.6, 30.2, 27.5, 22.4, 21.6, 14.0. HRMS (ESI): m/z calcd. for $C_{29}H_{30}FO_3S^+$([M+H]$^+$) = 477.1894, found = 477.1896.

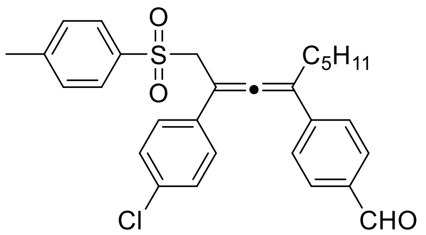

4e

4-(2-(4-chlorophenyl)-1-tosylnona-2,3-dien-4-yl)benzaldehyde (4e): Pale yellow oil, 73.8 mg, isolated yield 75%. ^{1}H NMR (400 MHz, $CDCl_3$) δ 9.92 (s, 1H), 7.74 (d, *J* = 8.4 Hz, 2H), 7.62 (d, *J* = 8.0 Hz, 2H), 7.41 (d, *J* = 8.4 Hz, 2H), 7.21 – 7.08 (m, 6H), 4.30 – 4.16 (m, 2H), 2.47 – 2.32 (m, 2H), 2.30 (s, 3H), 1.48 – 1.35 (m, 2H), 1.27 – 1.21 (m, 4H), 0.68 (t, *J* = 6.8 Hz, 3H). ^{13}C NMR (100 MHz, $CDCl_3$) δ 210.8, 191.6, 144.9, 141.4, 136.0, 135.4, 133.50, 132.3, 130.0, 129.7, 128.8, 128.4, 127.5, 127.0, 110.4, 99.0, 58.1, 31.7, 30.2, 27.5, 22.4, 21.6, 14.0. HRMS (ESI): m/z calcd. for $C_{29}H_{29}ClO_3SNa^+$([M+Na]$^+$) = 515.1418, found = 515.1414.

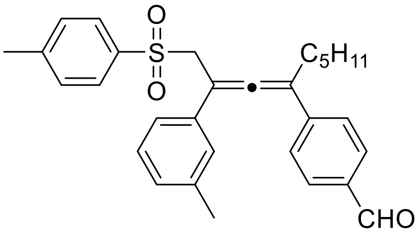

4f

4-(2-(*m*-tolyl)-1-tosylnona-2,3-dien-4-yl)benzaldehyde (4f): Pale yellow oil, 61.4 mg, isolated yield 65%. ^{1}H NMR (400 MHz, $CDCl_3$) δ 9.99 (s, 1H), 7.81 (d, *J* = 8.4 Hz, 2H), 7.69 (d, *J* = 8.4 Hz, 2H), 7.52 (d, *J* = 8.4 Hz, 2H), 7.19 – 7.10 (m, 4H), 7.05 – 6.99 (m, 2H), 4.40 – 4.27 (m, 2H), 2.55 – 2.39 (m, 2H), 2.35 (s, 3H), 2.26 (s, 3H), 1.58 – 1.47 (m, 2H), 1.39 – 1.24 (m, 4H), 0.86 (t, *J* = 7.2 Hz, 3H). ^{13}C NMR (100 MHz, $CDCl_3$) δ 210.8, 191.6, 144.5, 141.9, 138.1, 136.1, 135.2, 133.6, 129.9, 129.5, 128.5, 128.4, 128.4, 127.0, 126.7, 123.4, 109.8, 99.7, 58.1, 31.7, 30.2, 27.5, 22.4, 21.5, 21.4, 14.0. HRMS (ESI): m/z calcd. for $C_{30}H_{33}O_3S^+$($[M+H]^+$) = 473.2145, found = 473.2141.

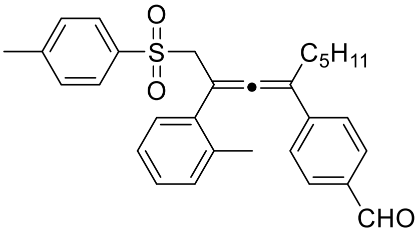

4g

4-(2-(*o*-tolyl)-1-tosylnona-2,3-dien-4-yl)benzaldehyde (4g): Pale yellow oil, 65.4 mg, isolated yield 69%. ^{1}H NMR (400 MHz, $CDCl_3$) δ 10.02 (s, 1H), 7.86 (d, *J* = 8.8 Hz, 2H), 7.67 (d, *J* = 8.4 Hz, 2H), 7.63 (d, *J* = 8.4 Hz, 2H), 7.20 (d, *J* = 8.0 Hz, 2H), 7.17 – 7.05 (m, 4H), 4.36 – 4.22 (m, 2H), 2.51 – 2.43 (m, 2H), 2.40 (s, 3H), 2.26 (s, 3H), 1.59 – 1.47 (m, 2H), 1.39 – 1.26 (m, 4H), 0.88 (t, *J* = 6.8 Hz, 3H). ^{13}C NMR (100 MHz, $CDCl_3$) δ 209.1, 191.7, 144.5, 142.1, 136.4, 135.6, 135.1, 134.6, 130.6, 129.8, 129.6, 128.4, 128.0, 127.6, 127.2, 125.9, 107.3, 98.0, 60.8, 31.6, 30.2, 27.5, 22.4, 21.5, 20.7, 14.0. HRMS (ESI): m/z calcd. for $C_{30}H_{33}O_3S^+$($[M+H]^+$) = 473.2145, found = 473.2144.

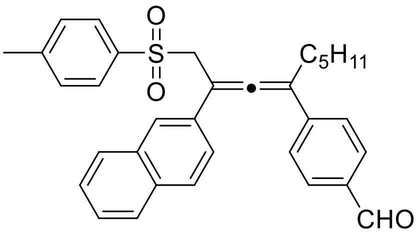

4h

4-(2-(naphthalen-2-yl)-1-tosylnona-2,3-dien-4-yl)benzaldehyde (4h): Pale yellow oil, 54.9 mg, isolated yield 54%. ^{1}H NMR (400 MHz, $CDCl_3$) δ 10.00 (s, 1H), 7.83 (d, *J* = 8.8 Hz, 2H), 7.78 – 7.73 (m, 1H), 7.72 – 7.65 (m, 4H), 7.60 (d, *J* = 1.6 Hz,

1H), 7.56 (d, *J* = 8.0 Hz, 2H), 7.48 – 7.38 (m, 3H), 7.08 (d, *J* = 8.0 Hz, 2H), 4.53 – 4.39 (m, 2H), 2.59 – 2.43 (m, 2H), 2.20 (s, 3H), 1.59 – 1.50 (m, 2H), 1.42 – 1.24 (m, 4H), 0.85 (t, *J* = 6.8 Hz, 3H). ^{13}C NMR (100 MHz, $CDCl_3$) δ 211.4, 191.6, 144.8, 141.8, 136.1, 135.3, 133.3, 132.6, 130.9, 130.0, 129.6, 128.4, 128.3, 128.1, 127.5, 127.0, 126.3, 126.2, 124.0, 124.4, 110.3, 100.0, 58.2, 31.7, 30.3, 27.6, 22.4, 21.4, 14.0. HRMS (ESI): m/z calcd. for $C_{33}H_{33}O_3S^+$([M+H]$^+$) = 509.2145, found = 509.2141.

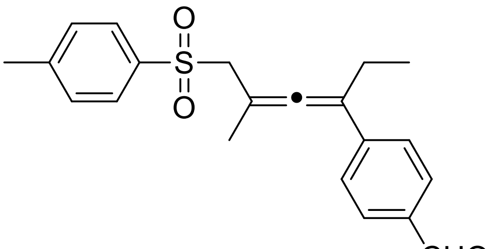

4i

4-(5-methyl-6-tosylhexa-3,4-dien-3-yl)benzaldehyde (4i): Pale yellow oil, 50.7 mg, isolated yield 72%. ^{1}H NMR (400 MHz, $CDCl_3$) δ 9.96 (s, 1H), 7.78 (d, *J* = 8.4 Hz, 2H), 7.74 (d, *J* = 8.4 Hz, 2H), 7.32 (d, *J* = 8.4 Hz, 2H), 7.28 (d, *J* = 8.0, 2H), 3.91 – 3.81 (m, 2H), 2.40 (s, 3H), 2.32 (q, *J* = 7.2 Hz, 2H), 1.99 (s, 3H), 0.99 (t, *J* = 7.2 Hz, 3H). ^{13}C NMR (100 MHz, $CDCl_3$) δ 207.4, 191.7, 144.8, 142.8, 136.0, 134.8, 129.8, 129.7, 128.2, 126.7, 107.8, 93.9, 61.4, 23.1, 21.6, 18.9, 12.4. HRMS (ESI): m/z calcd. for $C_{21}H_{23}O_3S^+$([M+H]$^+$) = 355.1362, found = 355.1364.

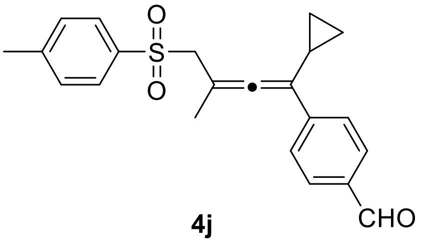

4j

4-(1-cyclopropyl-3-methyl-4-tosylbuta-1,2-dien-1-yl)benzaldehyde (4j): Pale yellow oil, 53.4 mg, isolated yield 73%. ^{1}H NMR (400 MHz, $CDCl_3$) δ 9.97 (s, 1H), 7.79 – 7.72 (m, 4H), 7.49 (d, *J* = 8.4 Hz, 2H), 7.28 (d, *J* = 8.0 Hz, 2H), 3.88 – 3.77 (m, 2H), 2.40 (s, 3H), 1.95 (s, 3H), 1.51 – 1.43 (m, 1H), 0.91 – 0.76 (m, 2H), 0.47 – 0.36 (m, 2H). ^{13}C NMR (100 MHz, $CDCl_3$) δ 206.8, 191.7, 144.8, 143.2, 136.0, 134.9, 129.9, 129.6, 128.1, 127.0, 109.5, 94.4, 61.2, 21.6, 18.9, 11.0, 7.3, 7.0. HRMS (ESI): m/z calcd. for $C_{22}H_{23}O_3S^+$([M+H]$^+$) = 367.1362, found = 367.1360.

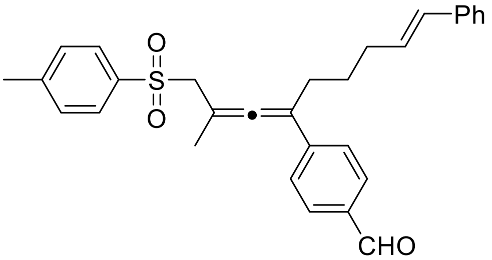

4k

(*E*)-4-(2-methyl-9-phenyl-1-tosylnona-2,3,8-trien-4-yl)benzaldehyde (4k): Pale yellow oil, 49.8 mg, isolated yield 53%. ^{1}H NMR (400 MHz, $CDCl_3$) δ 9.90 (s, 1H), 7.71 – 7.67 (m, 4H), 7.28 – 7.23 (m, 5H), 7.23 – 7.20 (m, 2H), 7.18 – 7.12 (m, 2H), 6.34 – 6.29 (m, 1H), 6.15 – 6.08 (m, 1H), 3.83 – 3.75 (m, 2H), 2.33 (s, 3H), 2.29 – 2.22 (m, 2H), 2.19 – 2.14 (m, 2H), 1.93 (s, 3H), 1.52 – 1.51 (m, 2H). ^{13}C NMR (100 MHz, $CDCl_3$) δ 207.6, 191.7, 144.8, 142.7, 137.5, 136.0, 134.9, 130.5, 129.9, 129.8, 129.7, 128.5, 128.2, 127.0, 126.8, 125.9, 105.9, 93.3, 61.3, 32.5, 29.4, 27.4, 21.6, 18.9. HRMS (ESI): m/z calcd. for $C_{30}H_{30}O_3SNa^+$($[M+Na]^+$) = 493.1808, found = 493.1804.

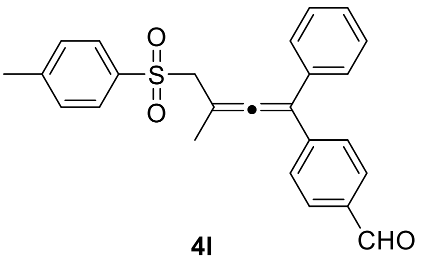

4l

4-(3-methyl-1-phenyl-4-tosylbuta-1,2-dien-1-yl)benzaldehyde (4l): Pale yellow oil, 50.7 mg, isolated yield 63%. ^{1}H NMR (500 MHz, $CDCl_3$) δ 10.00 (s, 1H), 7.78 (d, J = 6.8 Hz, 2H), 7.69 (d, J = 6.8 Hz, 2H), 7.36 – 7.27 (m, 5H), 7.16 – 7.09 (m, 4H), 3.98 – 3.90 (m, 2H), 2.33 (s, 3H), 2.07 (s, 3H). ^{13}C NMR (125 MHz, $CDCl_3$) δ 208.3, 191.6, 144.6, 142.8, 135.4, 135.3, 135.2, 129.7, 129.6, 129.0, 128.6, 128.5, 128.1, 127.8, 109.7, 93.7, 61.1, 21.6, 19.1. HRMS (ESI): m/z calcd. for $C_{25}H_{23}O_3S^+$($[M+H]^+$) = 403.1362, found = 403.1363.

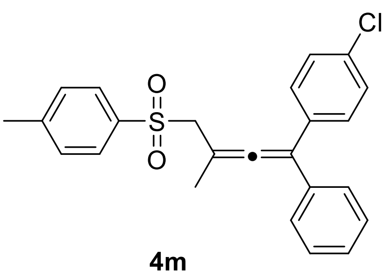

4m

1-chloro-4-(3-methyl-1-phenyl-4-tosylbuta-1,2-dien-1-yl)benzene (4m): Pale yellow oil, 67.7 mg, isolated yield 83%. ^{1}H NMR (400 MHz, $CDCl_3$) δ 7.67 (d, J = 8.0 Hz, 2H), 7.32 – 7.26 (m, 3H), 7.23 (d, J = 8.4 Hz, 2H), 7.14 – 7.07 (m, 4H), 7.04 (d, J = 8.8 Hz, 2H), 3.98 – 3.86 (m, 2H), 2.35 (s, 3H), 2.05 (s, 3H). ^{13}C NMR (100 MHz, $CDCl_3$) δ 207.4, 144.6, 135.8, 135.4, 134.7, 133.2, 129.9, 129.7, 128.5, 128.4, 128.4, 128.1, 127.7,

109.3, 93.2, 61.4, 21.6, 19.2. HRMS (ESI): m/z calcd. for $C_{24}H_{21}ClO_2SNa^+$([M+Na]$^+$) = 431.0843, found = 431.0841.

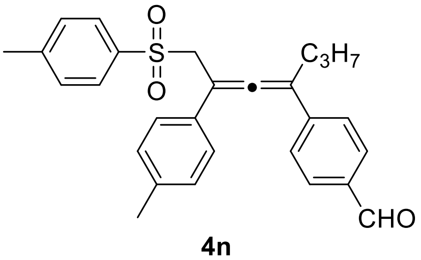

4n

4-(2-(*p*-tolyl)-1-tosylhepta-2,3-dien-4-yl)benzaldehyde (4n): Pale yellow oil, 73.7 mg, isolated yield 83%. ^{1}H NMR (400 MHz, $CDCl_3$) δ 9.98 (s, 1H), 7.79 (d, *J* = 8.4 Hz, 2H), 7.69 (d, *J* = 8.4 Hz, 2H), 7.48 (d, *J* = 8.4 Hz, 2H), 7.23 (d, *J* = 8.4 Hz, 2H), 7.18 (d, *J* = 7.6 Hz, 2H), 7.08 (d, *J* = 8.0 Hz, 2H), 4.42 – 4.28 (m, 2H), 2.50 – 2.38 (m, 2H), 2.35 (s, 3H), 2.32 (s, 3H), 1.60 – 1.43 (m, 2H), 0.95 (t, *J* = 7.6 Hz, 3H). ^{13}C NMR (100 MHz, $CDCl_3$) δ 210.6, 191.6, 144.6, 141.9, 137.6, 136.1, 135.2, 130.6, 129.9, 129.6, 129.3, 128.4, 126.9, 126.1, 109.7, 99.6, 58.0, 32.3, 21.6, 21.1, 21.1, 14.0. HRMS (ESI): m/z calcd. for $C_{28}H_{28}O_3SNa^+$([M+Na]$^+$) = 467.1651, found = 467.1648.

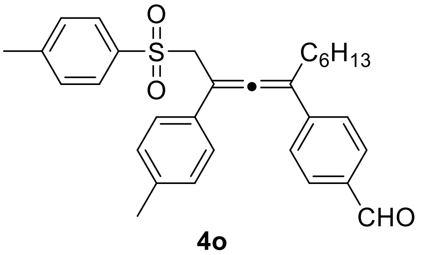

4o

4-(2-(*p*-tolyl)-1-tosyldeca-2,3-dien-4-yl)benzaldehyde (4o): Pale yellow oil, 69.0 mg, isolated yield 71%. ^{1}H NMR (400 MHz, $CDCl_3$) δ 9.98 (s, 1H), 7.79 (d, *J* = 8.4 Hz, 2H), 7.69 (d, *J* = 8.4 Hz, 2H), 7.48 (d, *J* = 8.0 Hz, 2H), 7.22 (d, *J* = 8.4 Hz, 2H), 7.17 (d, *J* = 7.6 Hz, 2H), 7.08 (d, *J* = 8.0 Hz, 2H), 4.39 – 4.26 (m, 2H), 2.54 – 2.39 (m, 2H), 2.35 (s, 3H), 2.32 (s, 3H), 1.55 – 1.43 (m, 2H), 1.38 – 1.19 (m, 6H), 0.85 (t, *J* = 7.2 Hz, 3H). ^{13}C NMR (100 MHz, $CDCl_3$) δ 210.5, 191.6, 144.6, 142.0, 137.6, 136.1, 135.2, 130.7, 129.9, 129.6, 129.3, 128.4, 126.9, 126.1, 109.9, 99.6, 58.1, 31.6, 30.3, 29.2, 27.8, 22.6, 21.6, 21.1, 14.0. HRMS (ESI): m/z calcd. for $C_{31}H_{34}O_3SNa^+$([M+Na]$^+$) = 509.2121, found = 509.2119.

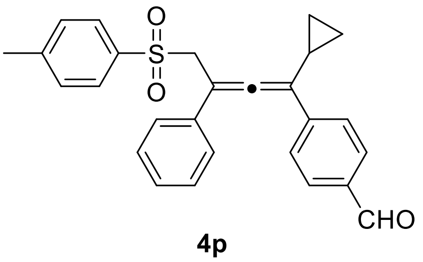

4p

4-(1-cyclopropyl-3-phenyl-4-tosylbuta-1,2-dien-1-yl)benzaldehyde (4p): Pale yellow oil, 51.4 mg, isolated yield 60%. ^{1}H NMR (400 MHz, $CDCl_3$) δ 9.98 (s, 1H), 7.80 (d, *J* = 8.4 Hz, 2H), 7.71 – 7.61 (m, 4H), 7.27 – 7.17 (m, 5H), 7.14 (d, *J* = 8.0 Hz, 2H), 4.38 – 4.22 (m, 2H), 2.32 (s, 3H), 1.67 – 1.61 (m, 1H), 0.96 – 0.85 (m, 2H), 0.66 – 0.58 (m, 1H), 0.57 – 0.50 (m, 1H). ^{13}C NMR (100 MHz, $CDCl_3$) δ 210.2, 191.7, 144.7, 142.3, 136.1, 135.4, 133.5, 129.8, 129.7, 128.7, 128.3, 127.7, 127.2, 126.1, 113.3, 100.9, 58.0, 21.6, 11.4, 7.3. HRMS (ESI): m/z calcd. for $C_{27}H_{25}O_3S^+$($[M+H]^+$) = 429.1519, found = 429.1520.

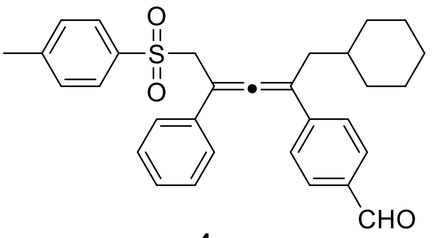

4q

4-(1-cyclohexyl-4-phenyl-5-tosylpenta-2,3-dien-2-yl)benzaldehyde (4q): Pale yellow oil, 74.5 mg, isolated yield 77%. ^{1}H NMR (400 MHz, $CDCl_3$) δ 9.90 (s, 1H), 7.73 (d, *J* = 8.4 Hz, 2H), 7.60 (d, *J* = 8.4 Hz, 2H), 7.42 (d, *J* = 8.4 Hz, 2H), 7.26 – 7.21 (m, 2H), 7.21 – 7.11 (m, 3H), 7.09 (d, *J* = 8.4 Hz, 2H), 4.33 – 4.18 (m, 2H), 2.34 – 2.13 (m, 5H), 1.69 – 1.49 (m, 5H), 1.42 – 1.30 (m, 1H), 1.13 – 0.98 (m, 3H), 0.89 – 0.73 (m, 2H). ^{13}C NMR (100 MHz, $CDCl_3$) δ 211.3, 191.6, 144.6, 141.8, 135.9, 135.2, 133.7, 129.9, 129.6, 128.5, 128.4, 127.5, 127.1, 126.2, 108.1, 98.7, 58.2, 38.1, 36.1, 33.3, 33.2, 26.2, 26.0, 21.5. HRMS (ESI): m/z calcd. for $C_{31}H_{33}O_3S^+$($[M+H]^+$) = 485.2145, found = 485.2141.

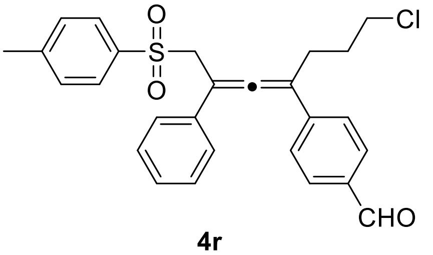

4r

4-(7-chloro-2-phenyl-1-tosylhepta-2,3-dien-4-yl)benzaldehyde (4r): Pale yellow oil, 56.6 mg, isolated yield 61%. ^{1}H NMR (400 MHz, $CDCl_3$) δ 9.92 (s, 1H), 7.75 (d, *J* = 8.4 Hz, 2H), 7.64 (d, *J* = 8.4 Hz, 2H), 7.47 (d, *J* = 8.4 Hz, 2H), 7.25 – 7.14 (m, 5H), 7.12 (d, *J* = 8.0 Hz, 2H), 4.26 (s, 2H), 3.52 (t, *J* = 6.0 Hz, 2H), 2.71 – 2.56 (m,

2H), 2.29 (s, 3H), 2.05 – 1.87 (m, 2H). ^{13}C NMR (100 MHz, $CDCl_3$) δ 210.5, 191.6, 144.8, 141.2, 136.1, 135.4, 133.5, 130.0, 129.7, 128.7, 128.3, 127.8, 126.9, 126.1, 108.9, 100.3, 57.9, 44.5, 30.4, 27.5, 21.6. HRMS (ESI): m/z calcd. for $C_{27}H_{26}ClO_3S^+$([M+H]$^+$) = 465.1286, found = 465.1287.

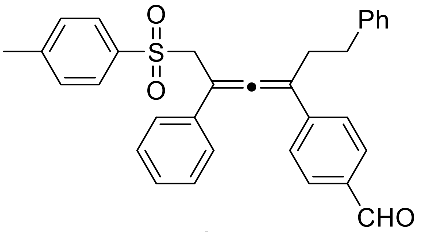

4s

4-(1,5-diphenyl-6-tosylhexa-3,4-dien-3-yl)benzaldehyde (4s): Pale yellow oil, 53.1 mg, isolated yield 54%. ^{1}H NMR (400 MHz, $CDCl_3$) δ 9.92 (s, 1H), 7.75 (d, J = 8.4 Hz, 2H), 7.58 (d, J = 8.0 Hz, 2H), 7.45 (d, J = 8.4 Hz, 2H), 7.22 – 7.04 (m, 12H), 4.12 (d, J = 14.4 Hz, 1H), 3.86 (d, J = 14.4 Hz, 1H), 2.77 (s, 4H), 2.23 (s, 3H). ^{13}C NMR (100 MHz, $CDCl_3$) δ 211.0, 191.6, 144.7, 141.4, 140.9, 136.1, 135.3, 133.5, 130.0, 129.6, 128.6, 128.5, 128.4, 128.3, 127.6, 127.0, 126.2, 126.2, 108.9, 100.1, 57.6, 33.6, 31.7, 21.5. HRMS (ESI): m/z calcd. for $C_{32}H_{29}O_3S^+$([M+H]$^+$) = 493.1832, found = 493.1835.

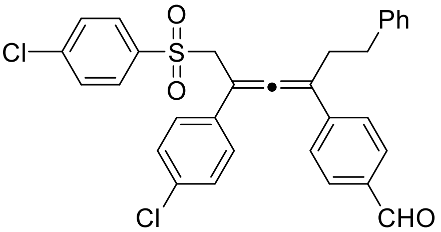

4t

4-(5-(4-chlorophenyl)-6-((4-chlorophenyl)sulfonyl)-1-phenylhexa-3,4-dien-3-yl)benzaldehyde (4t): Pale yellow oil, 61.2 mg, isolated yield 56%. ^{1}H NMR (400 MHz, $CDCl_3$) δ 9.76 (s, 1H), 7.59 (d, J = 8.4 Hz, 2H), 7.41 (d, J = 8.4 Hz, 2H), 7.20 (d, J = 8.4 Hz, 2H), 7.07 (d, J = 8.8 Hz, 2H), 7.04 – 6.92 (m, 5H), 6.85 (d, J = 8.4 Hz, 2H), 6.79 (d, J = 8.8 Hz, 2H), 3.90 (d, J = 14.8 Hz, 1H), 3.59 (d, J = 14.8 Hz, 1H), 2.68 – 2.52 (m, 4H). ^{13}C NMR (100 MHz, $CDCl_3$) δ 210.9, 191.5, 140.9, 140.6, 140.5, 137.2, 135.6, 133.7, 131.7, 130.1, 129.7, 129.3, 128.8, 128.6, 128.4, 127.4, 127.0, 126.3, 109.3, 98.8, 57.6, 33.3, 31.7. HRMS (ESI): m/z calcd. for $C_{31}H_{25}Cl_2O_3S^+$([M+H]$^+$) = 547.0896, found = 547.0894.

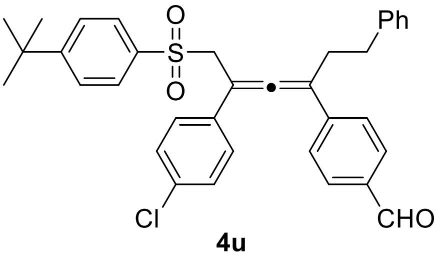

4u

4-(6-((4-(tert-butyl)phenyl)sulfonyl)-5-(4-chlorophenyl)-1-phenylhexa-3,4-dien-3-yl)benzaldehyde (4u): Pale yellow solid, 48.8 mg, isolated yield 43%. ^{1}H NMR (400 MHz, $CDCl_3$) δ 9.93 (s, 1H), 7.80 (d, *J* = 8.4 Hz, 2H), 7.60 (d, *J* = 8.8 Hz, 2H), 7.54 (d, *J* = 8.4 Hz, 2H), 7.30 (d, *J* = 8.8 Hz, 2H), 7.20 – 7.17 (m, 2H), 7.12 (d, *J* = 7.2 Hz, 1H), 7.08 – 7.04 (m, 2H), 7.00 (d, *J* = 8.8 Hz, 2H), 6.85 (d, *J* = 8.8 Hz, 2H), 4.05 (d, *J* = 14.4 Hz, 1H), 3.83 (d, *J* = 14.4 Hz, 1H), 2.88 – 2.77 (m, 4H), 1.20 (s, 9H). ^{13}C NMR (100 MHz, $CDCl_3$) δ 211.2, 191.6, 157.9, 141.1, 140.7, 135.9, 135.5, 133.2, 132.0, 130.1, 128.6, 128.5, 128.5, 128.2, 127.3, 127.1, 126.2, 126.0, 109.1, 99.1, 57.6, 35.2, 33.5, 31.7, 31.0. HRMS (ESI): m/z calcd. for $C_{35}H_{34}ClO_3S^+$($[M+H]^+$) = 569.1912, found = 569.1915.

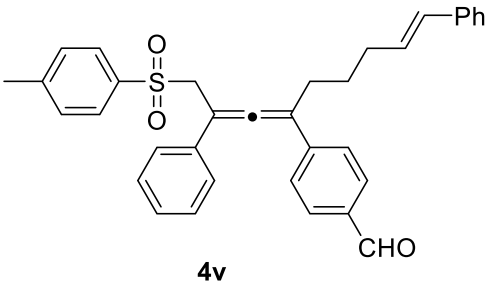

4v

(*E*)-4-(2,9-diphenyl-1-tosylnona-2,3,8-trien-4-yl)benzaldehyde (4v): Pale yellow oil, 40.4 mg, isolated yield 38%. ^{1}H NMR (400 MHz, $CDCl_3$) δ 9.99 (s, 1H), 7.81 (d, *J* = 8.4 Hz, 2H), 7.69 (d, *J* = 8.4 Hz, 2H), 7.52 (d, *J* = 8.4 Hz, 2H), 7.36 – 7.27 (m, 7H), 7.24 – 7.18 (m, 3H), 7.15 (d, *J* = 8.0 Hz, 2H), 6.33 (d, *J* = 15.6 Hz, 1H), 6.17 (dt, *J* = 15.6, 7.2 Hz, 1H), 4.42 – 4.28 (m, 2H), 2.61 – 2.51 (m, 2H), 2.34 (s, 3H), 2.31 – 2.23 (m, 2H), 1.78 – 1.66 (m, 2H). ^{13}C NMR (125 MHz, $CDCl_3$) δ 210.8, 191.7, 144.7, 141.6, 137.5, 136.1, 135.3, 133.7, 130.6, 130.0, 129.8, 129.7, 128.7, 128.5, 128.4, 127.7, 127.0, 127.0, 126.2, 125.9, 109.9, 99.9, 58.0, 32.7, 29.7, 27.4, 21.6. HRMS (ESI): m/z calcd. for $C_{35}H_{32}NaO_3S^+$($[M+Na]^+$) = 555.1964, found = 555.1962.

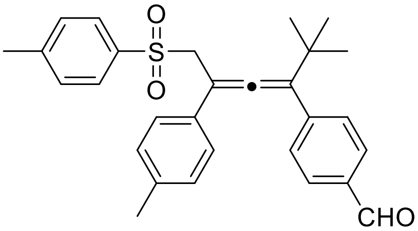

4w

4-(2,2-dimethyl-5-(*p*-tolyl)-6-tosylhexa-3,4-dien-3-yl)benzaldehyde (4w): Pale yellow oil, 31.1 mg, isolated yield 34%. ^{1}H NMR (500 MHz, $CDCl_3$) δ 10.02 (s, 1H), 7.80 (d, *J* = 6.8 Hz, 2H), 7.50 (d, *J* = 6.8 Hz, 2H), 7.36 (d, *J* = 6.4 Hz, 2H), 7.15 (d, *J* = 6.4 Hz, 2H), 7.05 (d, *J* = 6.0 Hz, 2H), 7.01 (d, *J* = 6.4 Hz, 2H), 4.27 (d, *J* = 11.6 Hz, 1H), 4.20 (d, *J* = 11.2 Hz, 1H), 2.31 (s, 3H), 2.28 (s, 3H), 1.17 (s, 9H). ^{13}C NMR (125 MHz, $CDCl_3$) δ 205.4, 191.8, 144.4, 142.9, 137.1, 135.7, 135.1, 131.4, 129.9, 129.4, 129.3, 129.2, 128.4, 125.7, 119.1, 97.5, 58.8, 36.1, 29.6, 21.6, 21.0. HRMS (ESI): m/z calcd. for $C_{29}H_{31}O_3S^+$($[M+H]^+$) = 459.1988, found = 459.1987.

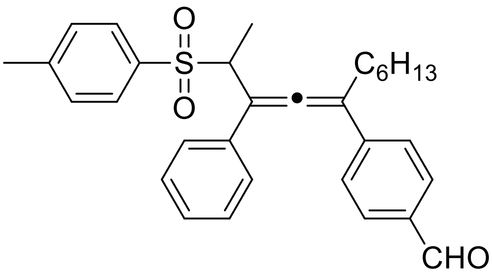

4x

4-(3-(*p*-tolyl)-2-tosylundeca-3,4-dien-5-yl)benzaldehyde (4x): Pale yellow oil, 40.6 mg, isolated yield 42%. ^{1}H NMR (400 MHz, $CDCl_3$) δ 9.88 (s, 1H), 7.72 (d, *J* = 8.5 Hz, 2H), 7.64 (d, *J* = 8.4 Hz, 2H), 7.41 (d, *J* = 8.4 Hz, 2H), 7.24 – 7.09 (m, 7H), 4.32 (q, *J* = 7.2 Hz, 1H), 2.54 – 2.45 (m, 2H), 2.31 (s, 3H), 1.67 – 1.51 (m, 2H), 1.46 (d, *J* = 7.2 Hz, 3H), 1.40 – 1.30 (m, 2H), 1.30 – 1.21 (m, 4H), 0.81 (t, *J* = 6.8 Hz, 3H). ^{13}C NMR (100 MHz, $CDCl_3$) δ 210.2, 191.6, 144.6, 141.7, 135.2, 134.9, 134.8, 130.0, 129.5, 129.2, 128.5, 127.3, 126.5, 126.4, 111.9, 105.9, 60.9, 31.6, 30.1, 29.4, 28.2, 22.7, 21.5, 14.7, 14.1. HRMS (ESI): m/z calcd. for $C_{31}H_{34}O_3SNa^+$($[M+Na]^+$) = 509.2121, found = 509.2118.

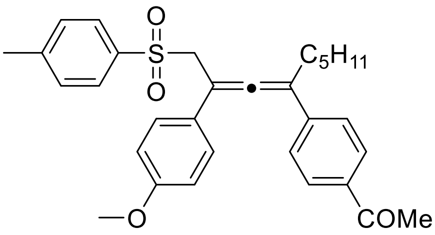

5a

1-(4-(2-(4-methoxyphenyl)-1-tosylnona-2,3-dien-4-yl)phenyl)ethan-1-one (5a): Pale yellow oil, for the reaction of 4'-iodoacetophenone (84.3 mg, isolated yield 84%), for the reaction of 4'-bromoacetophenone (79.3 mg, isolated yield 79%). ^{1}H NMR (500 MHz, $CDCl_3$) δ 7.89 (d, *J* = 6.8 Hz, 2H), 7.71 (d, *J* = 6.4 Hz, 2H), 7.42

(d, J = 6.8 Hz, 2H), 7.28 (d, J = 7.2 Hz, 2H), 7.20 (d, J = 6.4 Hz, 2H), 6.82 (d, J = 6.6 Hz, 2H), 4.39 – 4.30 (m, 2H), 3.80 (s, 3H), 2.60 (s, 3H), 2.47 – 2.36 (m, 2H), 2.37 (s, 3H), 1.54 – 1.45 (m, 2H), 1.35 – 1.27 (m, 4H), 0.87 (t, J = 7.0 Hz, 3H). ^{13}C NMR (125 MHz, $CDCl_3$) δ 210.1, 197.6, 159.2, 144.6, 140.6, 136.1, 135.8, 129.7, 128.6, 128.4, 127.5, 126.5, 126.0, 114.1, 109.8, 99.2, 58.3, 55.3, 31.7, 30.3, 27.6, 26.6, 22.5, 21.6, 14.1. HRMS (ESI): m/z calcd. for $C_{31}H_{34}O_4SNa^+$($[M+Na]^+$) = 525.2070, found = 525.2072.

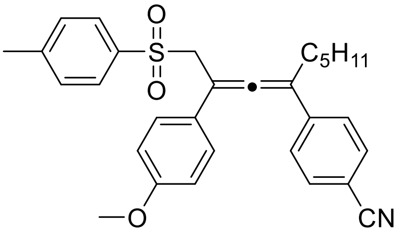

5b

4-(2-(4-methoxyphenyl)-1-tosylnona-2,3-dien-4-yl)benzonitrile (5b): Pale yellow oil, for the reaction of 4-iodobenzonitrile (80.5 mg, isolated yield 83%), for the reaction of 4-bromobenzonitrile (82.5 mg, isolated yield 85%). ^{1}H NMR (400 MHz, $CDCl_3$) δ 7.69 (d, J = 8.4 Hz, 2H), 7.56 (d, J = 8.8 Hz, 2H), 7.45 (d, J = 8.8 Hz, 2H), 7.23 (d, J = 8.8 Hz, 2H), 7.19 (d, J = 8.0 Hz, 2H), 6.80 (d, J = 8.8 Hz, 2H), 4.36 – 4.24 (m, 2H), 3.78 (s, 3H), 2.49 – 2.34 (m, 5H), 1.54 – 1.43 (m, 2H), 1.33 – 1.23 (m, 4H), 0.85 (t, J = 6.8 Hz, 3H). ^{13}C NMR (100 MHz, $CDCl_3$) δ 210.1, 159.3, 144.6, 140.6, 136.2, 132.2, 129.6, 128.3, 127.4, 127.0, 125.6, 118.9, 114.1, 110.6, 109.4, 99.6, 58.2, 55.3, 31.6, 30.1, 27.4, 22.4, 21.6, 14.0. HRMS (ESI): m/z calcd. for $C_{30}H_{32}NO_3S^+$($[M+H]^+$) = 486.2097, found = 486.2095.

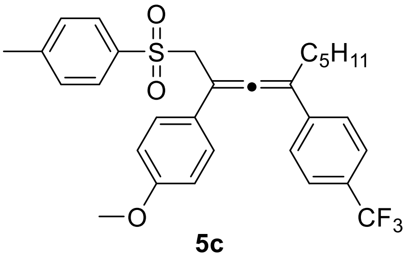

5c

1-methoxy-4-(1-tosyl-4-(4-(trifluoromethyl)phenyl)nona-2,3-dien-2-yl)benzene (5c): Pale yellow oil, for the reaction of 1-iodo-4-(trifluoromethyl)benzene (70.7 mg, isolated yield 67%), for the reaction of 1-bromo-4-(trifluoromethyl)benzene (79.2 mg, isolated yield 75%). ^{1}H NMR (400 MHz, $CDCl_3$) δ 7.60 (d, J = 8.4 Hz, 2H), 7.44 (d, J = 8.4 Hz, 2H), 7.32 (d, J = 8.4 Hz, 2H), 7.19 (d, J = 8.8 Hz, 2H), 7.08 (d, J = 8.0 Hz, 2H), 6.73 (d, J = 8.8 Hz, 2H), 4.30 – 4.17 (m, 2H),

3.71 (s, 3H), 2.41 – 2.29 (m, 2H), 2.27 (s, 3H), 1.44 – 1.35 (m, 2H), 1.24 – 1.17 (m, 4H), 0.77 (t, *J* = 6.8 Hz, 3H). ^{13}C NMR (125 MHz, $CDCl_3$) δ 209.5, 159.2, 144.6, 139.3, 136.0, 129.6, 129.4 (q, *J* = 32.5 Hz), 128.4, 127.4, 127.3 (q, *J* = 348.8 Hz), 126.6, 125.9, 125.3 (q, *J* = 2.5 Hz), 114.1, 109.4, 99.2, 58.3, 55.3, 31.7, 30.3, 27.5, 22.4, 21.5, 14.0. HRMS (ESI): m/z calcd. for $C_{30}H_{31}F_3O_3SNa^+$($[M+Na]^+$) = 551.1838, found = 551.1839.

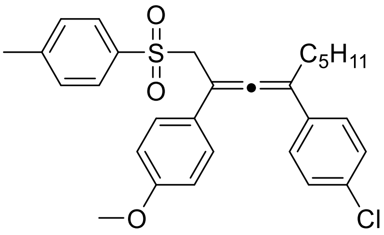

5d

1-chloro-4-(2-(4-methoxyphenyl)-1-tosylnona-2,3-dien-4-yl)benzene (5d): Pale yellow oil, for the reaction of 1-chloro-4-iodobenzene (60.3 mg, isolated yield 61%), for the reaction of 1-bromo-4-chlorobenzene (56.3 mg, isolated yield 57%). ^{1}H NMR (400 MHz, $CDCl_3$) δ 7.59 (d, *J* = 8.0 Hz, 2H), 7.19 (d, *J* = 8.8 Hz, 2H), 7.17 – 7.10 (m, 4H), 7.08 (d, *J* = 8.0 Hz, 2H), 6.73 (d, *J* = 8.8 Hz, 2H), 4.30 – 4.17 (m, 2H), 3.71 (s, 3H), 2.36 – 2.19 (m, 5H), 1.42 – 1.32 (m, 2H), 1.24 – 1.16 (m, 4H), 0.77 (t, *J* = 7.2 Hz, 3H). ^{13}C NMR (100 MHz, $CDCl_3$) δ 209.0, 159.1, 144.5, 136.0, 133.9, 133.0, 129.5, 128.5, 128.3, 127.7, 127.3, 126.2, 114.0, 109.4, 98.8, 58.4, 55.3, 31.7, 30.4, 27.5, 22.4, 21.5, 14.0. HRMS (ESI): m/z calcd. for $C_{29}H_{31}ClO_3SNa^+$($[M+Na]^+$) = 517.1575, found = 517.1574.

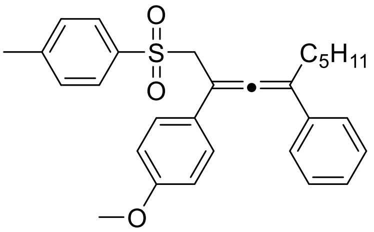

5e

1-methoxy-4-(4-phenyl-1-tosylnona-2,3-dien-2-yl)benzene (5e): Pale yellow oil, for the reaction of iodobenzene (54.3 mg, isolated yield 59%), for the reaction of bromobenzene (47.9 mg, isolated yield 52%). ^{1}H NMR (400 MHz, $CDCl_3$) δ 7.60 (d, *J* = 8.0 Hz, 2H), 7.22 (d, *J* = 9.2 Hz, 2H), 7.20 –7.17 (m, 4H), 7.17 – 7.12 (m, 1H), 7.08 (d, *J* = 8.0 Hz, 2H), 6.73 (d, *J* = 8.8 Hz, 2H), 4.24 (s, 2H), 3.71 (s, 3H), 2.40 – 2.18 (m, 5H), 1.43 – 1.32 (m, 2H), 1.27 – 1.15 (m, 4H), 0.78 (t, *J* = 7.2 Hz, 3H); ^{13}C NMR (125 MHz, $CDCl_3$) δ 209.1, 158.9, 144.3, 136.0, 135.4, 129.5, 128.4, 128.4, 127.4, 127.3, 126.6, 126.4, 113.9, 110.1, 98.4, 58.5, 55.3, 31.7, 30.4, 27.6, 22.4, 21.6, 14.0. HRMS (ESI):

m/z calcd. for $C_{29}H_{33}O_3S^+$([M+H]$^+$) = 461.2145, found = 461.2147.

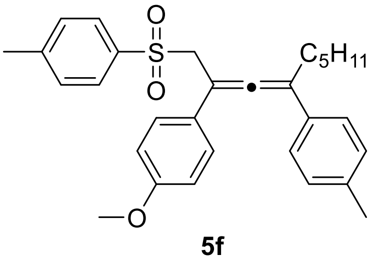

5f

1-methoxy-4-(4-(*p*-tolyl)-1-tosylnona-2,3-dien-2-yl)benzene (5f): Pale yellow oil, for the reaction of 1-iodo-4-methylbenzene (54.0 mg, isolated yield 57%), for the reaction of 1-bromo-4-methylbenzene (36.9 mg, isolated yield 39%). ^{1}H NMR (500 MHz, $CDCl_3$) δ 7.69 (d, *J* = 6.8 Hz, 2H), 7.30 (d, *J* = 6.4 Hz, 2H), 7.16 (d, *J* = 6.4 Hz, 4H), 7.09 (d, *J* = 6.4 Hz, 2H), 6.81 (d, *J* = 6.8 Hz, 2H), 4.32 (s, 2H), 3.79 (s, 3H), 2.41 – 2.25 (m, 8H), 1.48 – 1.42 (m, 2H), 1.31 – 1.24 (m, 4H), 0.85 (t, *J* = 6.6 Hz, 3H). ^{13}C NMR (125 MHz, $CDCl_3$) δ 209.0, 158.9, 144.3, 137.1, 136.0, 132.3, 129.5, 129.1, 128.4, 127.3, 126.7, 126.3, 113.9, 110.0, 98.2, 58.6, 55.3, 31.8, 30.4, 27.6, 22.4, 21.5, 21.1, 14.0. HRMS (ESI): m/z calcd. for $C_{30}H_{34}O_3SNa^+$([M+Na]$^+$) = 497.2121, found = 497.2115.

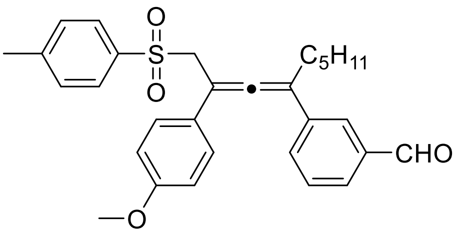

5g

3-(2-(4-methoxyphenyl)-1-tosylnona-2,3-dien-4-yl)benzaldehyde (5g): Pale yellow oil, 50.6 mg, isolated yield 52%. ^{1}H NMR (400 MHz, $CDCl_3$) δ 10.00 (s, 1H), 7.86 (t, *J* = 1.2 Hz, 1H), 7.77 – 7.73 (m, 1H), 7.69 (d, *J* = 8.4 Hz, 2H), 7.65 – 7.60 (m, 1H), 7.45 (t, *J* = 7.6 Hz, 1H), 7.26 (d, *J* = 8.8 Hz, 2H), 7.16 (d, *J* = 8.0 Hz, 2H), 6.80 (d, *J* = 8.8 Hz, 2H), 4.40 – 4.24 (m, 2H), 3.78 (s, 3H), 2.55 – 2.37 (m, 2H), 2.34 (s, 3H), 1.54 – 1.43 (m, 2H), 1.35 – 1.25 (m, 4H), 0.85 (t, *J* = 6.8 Hz, 3H). ^{13}C NMR (100 MHz, $CDCl_3$) δ 209.2, 192.3, 159.1, 144.5, 136.8, 136.7, 136.2, 132.7, 129.6, 129.2, 128.6, 128.3, 127.4, 127.3, 126.1, 114.1, 109.5, 99.2, 58.4, 55.3, 31.7, 30.3, 27.5, 22.4, 21.5, 14.0. HRMS (ESI): m/z calcd. for $C_{30}H_{33}O_4S^+$([M+H]$^+$) = 489.2094, found = 489.2095.

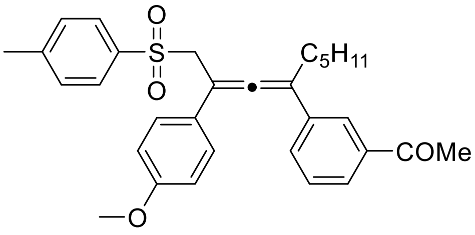

5h

1-(3-(2-(4-methoxyphenyl)-1-tosylnona-2,3-dien-4-yl)phenyl)ethan-1-one (5h): Pale yellow oil, 55.2 mg, isolated yield 55%. ^{1}H NMR (400 MHz, $CDCl_3$) δ 8.04 (t, *J* = 1.2 Hz, 1H), 7.84 – 7.81 (m, 1H), 7.70 (d, *J* = 8.4 Hz, 2H), 7.57 – 7.53 (m, 1H), 7.39 (t, *J* = 8.0 Hz, 1H), 7.25 (d, *J* = 9.2 Hz, 2H), 7.17 (d, *J* = 7.6 Hz, 2H), 6.79 (d, *J* = 9.2 Hz, 2H), 4.36 – 4.25 (m, 2H), 3.78 (s, 3H), 2.61 (s, 3H), 2.51 – 2.38 (m, 2H), 2.35 (s, 3H), 1.53 – 1.43 (m, 2H), 1.35 – 1.27 (m, 4H), 0.85 (t, *J* = 6.8 Hz, 3H). ^{13}C NMR (100 MHz, $CDCl_3$) δ 209.2, 198.3, 159.1, 144.5, 137.5, 136.3, 136.2, 131.3, 129.6, 128.7, 128.4, 127.4, 127.2, 126.4, 126.2, 114.0, 109.8, 99.0, 58.5, 55.3, 31.7, 30.4, 27.5, 26.8, 22.4, 21.6, 14.0. HRMS (ESI): m/z calcd. for $C_{31}H_{34}O_4SNa^+$([M+Na]$^+$) = 525.2070, found = 525.2071.

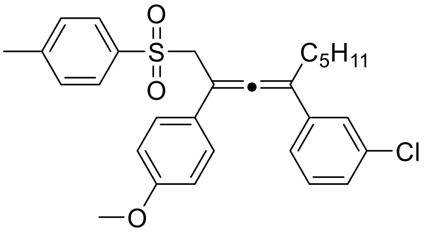

5i

1-chloro-3-(2-(4-methoxyphenyl)-1-tosylnona-2,3-dien-4-yl)benzene (5i): Pale yellow oil, 50.4 mg, isolated yield 51%. ^{1}H NMR (400 MHz, $CDCl_3$) δ 7.60 (d, *J* = 8.0 Hz, 2H), 7.22 – 7.16 (m, 3H), 7.15 – 7.10 (m, 3H), 7.08 (d, *J* = 8.0 Hz, 2H), 6.73 (d, *J* = 8.8 Hz, 2H), 4.28 – 4.19 (m, 2H), 3.71 (s, 3H), 2.35 – 2.19 (m, 5H), 1.43 – 1.32 (m, 2H), 1.25 – 1.16 (m, 4H), 0.78 (t, *J* = 6.8 Hz, 3H). ^{13}C NMR (100 MHz, $CDCl_3$) δ 209.0, 159.1, 144.4, 137.6, 136.0, 135.4, 129.6, 129.5, 128.3, 127.4, 127.3, 126.4, 126.2, 124.7, 114.0, 109.4, 99.0, 58.3, 55.3, 31.7, 30.4, 27.5, 22.4, 21.6, 14.0. HRMS (ESI): m/z calcd. for $C_{29}H_{31}ClO_3SNa^+$([M+Na]$^+$) = 517.1575, found = 517.1572.

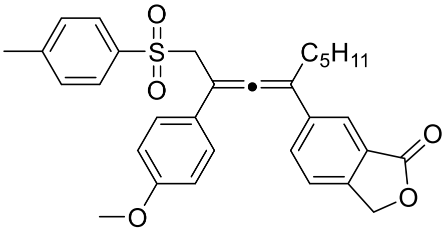

5j

5-(2-(4-methoxyphenyl)-1-tosylnona-2,3-dien-4-yl)-2,3-dihydro-1H-inden-1-one (5j): Pale yellow oil, 94.6 mg, isolated yield 92%. ^{1}H NMR (400 MHz, $CDCl_3$) δ 7.82 (d, *J* = 8.0 Hz, 1H), 7.70 (d, *J* = 8.4 Hz, 2H), 7.55 (s, 1H), 7.50 (dd, *J* = 8.0,

1.2 Hz, 1H), 7.26 – 7.22 (d, J = 8.8, 2H), 7.19 (d, J = 8.0 Hz, 2H), 6.80 (d, J = 8.8 Hz, 2H), 5.28 (s, 2H), 4.35 – 4.26 (m, 2H), 3.78 (s, 3H), 2.54 – 2.40 (m, 2H), 2.36 (s, 3H), 1.54 – 1.46 (m, 2H), 1.37 – 1.27 (m, 4H), 0.85 (t, J = 6.8 Hz, 3H). ^{13}C NMR (100 MHz, $CDCl_3$) δ 210.2, 170.8, 159.3, 147.1, 144.6, 142.2, 136.2, 129.6, 128.3, 127.4, 127.3, 125.6, 125.6, 124.4, 119.9, 114.1, 109.8, 99.3, 69.6, 58.2, 55.3, 31.6, 30.4, 27.5, 22.4, 21.5, 14.0. HRMS (ESI): m/z calcd. for $C_{31}H_{32}O_5SNa^+$($[M+Na]^+$) = 539.1863, found = 539.1862.

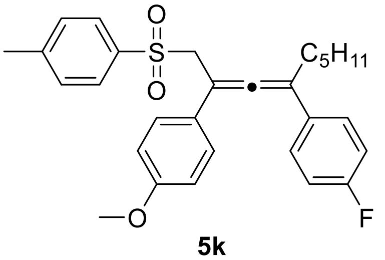

5k

1-fluoro-4-(2-(4-methoxyphenyl)-1-tosylnona-2,3-dien-4-yl)benzene (5k): Pale yellow oil, 57.4 mg, isolated yield 60%. ^{1}H NMR (400 MHz, $CDCl_3$) δ 7.65 (d, J = 8.4 Hz, 2H), 7.27 – 7.20 (m, 4H), 7.14 (d, J = 8.0 Hz, 2H), 6.94 (t, J = 8.4 Hz, 2H), 6.78 (d, J = 8.8 Hz, 2H), 4.34 – 4.23 (m, 2H), 3.76 (s, 3H), 2.40 – 2.26 (m, 5H), 1.48 – 1.38 (m, 2H), 1.29 – 1.22 (m, 4H), 0.83 (t, J = 7.2 Hz, 3H). ^{13}C NMR (100 MHz, $CDCl_3$) δ 208.8 (d, J = 2.0 Hz), 162.1 (d, J = 245.0 Hz), 159.0, 144.4, 136.1, 131.4, 129.5, 128.4, 128.0 (d, J = 8.0 Hz), 127.3, 126.5, 115.3 (d, J = 21.0 Hz), 114.0, 109.4, 98.6, 58.5, 55.3, 31.7, 30.6, 27.5, 22.4, 21.5, 14.0. HRMS (ESI): m/z calcd. for $C_{29}H_{31}FO_3SNa^+$($[M+Na]^+$) = 501.1870, found = 501.1868.

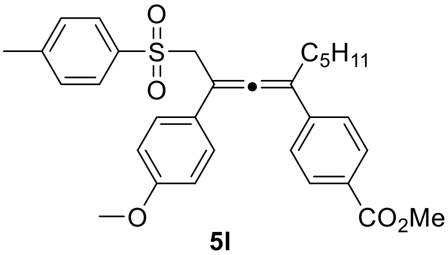

5l

methyl 4-(2-(4-methoxyphenyl)-1-tosylnona-2,3-dien-4-yl)benzoate (5l): Pale yellow oil, 71.5 mg, isolated yield 69%. ^{1}H NMR (400 MHz, $CDCl_3$) δ 7.85 (d, J = 8.4 Hz, 2H), 7.59 (d, J = 8.0 Hz, 2H), 7.24 (d, J = 8.4 Hz, 2H), 7.19 (d, J = 9.2 Hz, 2H), 7.08 (d, J = 8.0 Hz, 2H), 6.73 (d, J = 8.8 Hz, 2H), 4.30 – 4.19 (m, 2H), 3.83 (s, 3H), 3.70 (s, 3H), 2.39 – 2.24 (m, 5H), 1.44 – 1.34 (m, 2H), 1.25 – 1.16 (m, 4H), 0.76 (t, J = 6.8 Hz, 3H). ^{13}C NMR (100 MHz, $CDCl_3$) δ 209.8, 166.8, 159.1, 144.5, 140.3, 136.0, 129.7, 129.6, 128.7, 128.4, 127.4, 126.2, 126.0, 114.0, 109.7, 99.1, 58.3,

55.2, 52.0, 31.6, 30.3, 27.5, 22.4, 21.5, 14.0. HRMS (ESI): m/z calcd. for $C_{31}H_{35}O_5S^+$([M+H]$^+$) = 519.2200, found = 519.2198.

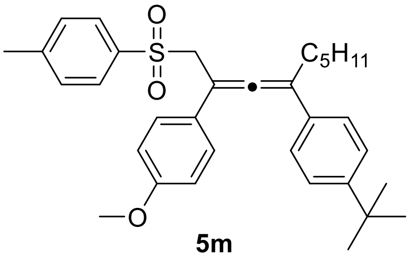

5m

1-(*tert*-butyl)-4-(2-(4-methoxyphenyl)-1-tosylnona-2,3-dien-4-yl)benzene (5m): Pale yellow oil, 58.8 mg, isolated yield 57%. ^{1}H NMR (400 MHz, $CDCl_3$) δ 7.61 (d, *J* = 8.4 Hz, 2H), 7.24 – 7.20 (m, 4H), 7.14 (d, *J* = 8.4 Hz, 2H), 7.09 (d, *J* = 8.0 Hz, 2H), 6.71 (d, *J* = 8.8 Hz, 2H), 4.23 (s, 2H), 3.70 (s, 3H), 2.29 (s, 3H), 2.28-2.15 (m, 2H), 1.39 – 1.36 (m, 2H), 1.23 (s, 9H), 1.21 – 1.16 (m, 4H), 0.77 (t, *J* = 6.8 Hz, 3H). ^{13}C NMR (100 MHz, $CDCl_3$) δ 209.2, 158.9, 150.3, 144.3, 136.1, 132.2, 129.5, 128.5, 127.4, 126.7, 126.1, 125.4, 113.9, 109.8, 98.4, 58.6, 55.2, 34.5, 31.8, 31.3, 30.2, 27.7, 22.4, 21.6, 14.0. HRMS (ESI): m/z calcd. for $C_{33}H_{41}O_3S^+$([M+H]$^+$) = 517.2771, found = 517.2765.

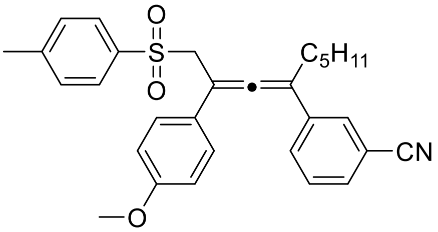

5n

3-(2-(4-methoxyphenyl)-1-tosylnona-2,3-dien-4-yl)benzonitrile (5n): Pale yellow oil, 62.1 mg, isolated yield 64%. ^{1}H NMR (400 MHz, $CDCl_3$) δ 7.69 (d, *J* = 8.0 Hz, 2H), 7.63 – 7.59 (m, 1H), 7.57 – 7.56 (m, 1H), 7.52 – 7.48 (m, 1H), 7.41 – 7.37 (m, 1H), 7.23 (d, *J* = 8.8 Hz, 2H), 7.18 (d, *J* = 8.0 Hz, 2H), 6.80 (d, *J* = 8.8 Hz, 2H), 4.35 – 4.26 (m, 2H), 3.78 (s, 3H), 2.47 – 2.28 (m, 5H), 1.53 – 1.43 (m, 2H), 1.35 – 1.23 (m, 4H), 0.85 (t, *J* = 6.8 Hz, 3H). ^{13}C NMR (100 MHz, $CDCl_3$) δ 209.2, 159.2, 144.6, 137.1, 136.1, 131.0, 130.6, 129.6, 129.6, 129.2, 128.3, 127.3, 125.7, 118.8, 114.1, 112.6, 108.9, 99.6, 58.1, 55.3, 31.6, 30.2, 27.4, 22.4, 21.5, 14.0. HRMS (ESI): m/z calcd. for $C_{30}H_{31}NO_3SNa^+$([M+Na]$^+$) = 508.1917, found = 508.1916.

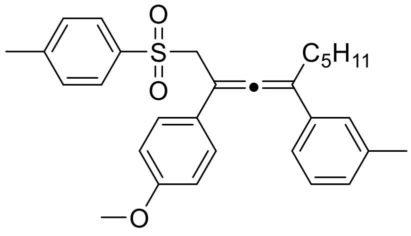

5o

1-(2-(4-methoxyphenyl)-1-tosylnona-2,3-dien-4-yl)-3-methylbenzene (5o): Pale yellow oil, 56.9 mg, isolated yield 60%. ^{1}H NMR (400 MHz, $CDCl_3$) δ 7.68 (d, *J* = 8.4 Hz, 2H), 7.30 (d, *J* = 8.8 Hz, 2H), 7.19 – 7.12 (m, 4H), 7.10 – 7.02 (m, 2H), 6.81 (d, *J* = 8.8 Hz, 2H), 4.36 – 4.28 (m, 2H), 3.79 (s, 3H), 2.41 – 2.27 (m, 8H), 1.51 – 1.41 (m, 2H), 1.33 – 1.25 (m, 4H), 0.86 (t, *J* = 6.8 Hz, 3H). ^{13}C NMR (100 MHz, $CDCl_3$) δ 209.1, 158.9, 144.3, 138.0, 136.1, 135.4, 129.5, 128.4, 128.3, 128.1, 127.3, 127.1, 126.7, 123.6, 113.9, 110.2, 98.2, 58.6, 55.3, 31.8, 30.4, 27.7, 22.4, 21.5, 21.5, 14.0. HRMS (ESI): m/z calcd. for $C_{30}H_{34}O_3SNa^+$($[M+Na]^+$) = 497.2121, found = 497.2119.

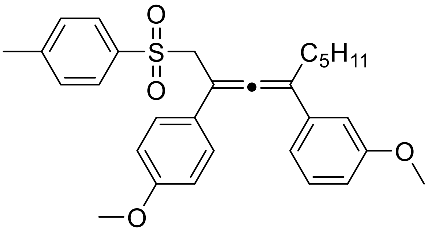

5p

1-methoxy-3-(2-(4-methoxyphenyl)-1-tosylnona-2,3-dien-4-yl)benzene (5p): Pale yellow oil, 59.8 mg, isolated yield 61%. ^{1}H NMR (400 MHz, $CDCl_3$) δ 7.60 (d, *J* = 8.4 Hz, 2H), 7.19 (d, *J* = 8.8 Hz, 2H), 7.11 (t, *J* = 8.0 Hz, 1H), 7.07 (d, *J* = 8.0 Hz, 2H), 6.89 (s, 1H), 6.81 (d, *J* = 8.0 Hz, 1H), 6.71 (d, *J* = 8.8 Hz, 3H), 4.27 – 4.17 (m, 2H), 3.71 (s, 3H), 3.69 (s, 3H), 2.35 – 2.18 (m, 5H), 1.43 – 1.32 (m, 2H), 1.23 – 1.16 (m, 4H), 0.76 (t, *J* = 6.8 Hz, 3H). ^{13}C NMR (100 MHz, $CDCl_3$) δ 209.2, 159.7, 158.9, 144.3, 137.0, 136.2, 129.5, 129.3, 128.4, 127.3, 126.6, 118.8, 113.9, 112.8, 112.3, 110.1, 98.4, 58.6, 55.3, 55.2, 31.7, 30.4, 27.6, 22.4, 21.5, 14.0. HRMS (ESI): m/z calcd. for $C_{30}H_{34}O_4SNa^+$($[M+Na]^+$) = 513.2070, found = 513.2069.

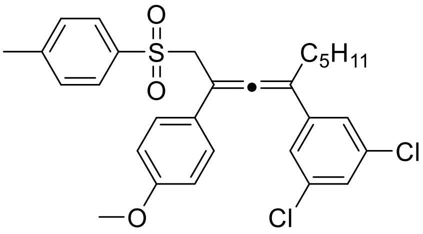

5q

1,3-dichloro-5-(2-(4-methoxyphenyl)-1-tosylnona-2,3-dien-4-yl)benzene (5q): Pale yellow oil, 91.9 mg, isolated yield 87%. ^{1}H NMR (400 MHz, $CDCl_3$) δ 7.68 (d, *J* = 8.4 Hz, 2H), 7.24 (d, *J* = 9.2 Hz, 2H), 7.22 – 7.20 (m, 1H), 7.19 – 7.16 (m, 3H), 7.16 (s, 1H), 6.81 (d, *J* = 8.8 Hz, 2H), 4.35 – 4.26 (m, 2H), 3.79 (s, 3H), 2.41

– 2.27 (m, 5H), 1.51 – 1.40 (m, 2H), 1.32 – 1.24 (m, 4H), 0.85 (t, *J* = 7.2 Hz, 3H). ^{13}C NMR (100 MHz, $CDCl_3$) δ 209.0, 159.3, 144.5, 139.1, 135.9, 135.0, 129.6, 128.3, 127.4, 127.2, 125.7, 124.8, 114.1, 108.8, 99.5, 58.1, 55.3, 31.6, 30.4, 27.4, 22.4, 21.5, 14.0. HRMS (ESI): m/z calcd. for $C_{29}H_{30}Cl_2O_3SNa^+$([M+Na]$^+$) = 551.1182, found = 551.1185.

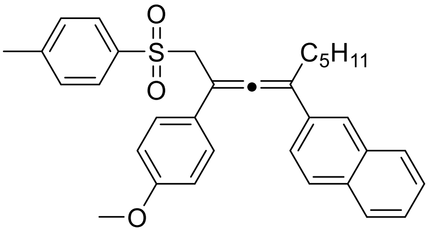

5r

2-(2-(4-methoxyphenyl)-1-tosylnona-2,3-dien-4-yl)naphthalene (5r): Pale yellow oil, 87.7 mg, isolated yield 86%. ^{1}H NMR (400 MHz, $CDCl_3$) δ 7.83 – 7.77 (m, 2H), 7.73 – 7.67 (m, 4H), 7.50 – 7.43 (m, 2H), 7.39 (dd, *J* = 8.4, 1.2 Hz, 1H), 7.35 (d, *J* = 9.2 Hz, 2H), 7.08 (d, *J* = 8.0 Hz, 2H), 6.83 (d, *J* = 8.8 Hz, 2H), 4.42 – 4.32 (m, 2H), 3.80 (s, 3H), 2.59 – 2.39 (m, 2H), 2.22 (s, 3H), 1.58 – 1.47 (m, 2H), 1.38 – 1.28 (m, 4H), 0.87 (t, *J* = 6.8 Hz, 3H). ^{13}C NMR (100 MHz, $CDCl_3$) δ 209.7, 159.0, 144.3, 136.0, 133.5, 132.9, 132.7, 129.5, 128.4, 128.1, 127.9, 127.5, 127.4, 126.5, 126.1, 125.9, 125.4, 124.4, 114.0, 110.5, 98.7, 58.5, 55.3, 31.8, 30.4, 27.7, 22.4, 21.4, 14.0. HRMS (ESI): m/z calcd. for $C_{33}H_{34}O_3SNa^+$([M+Na]$^+$) = 533.2121, found = 533.2125.

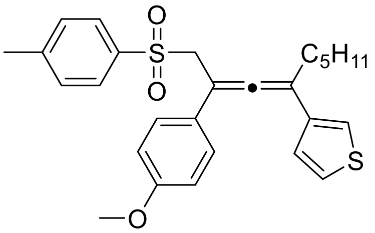

5s

3-(2-(4-methoxyphenyl)-1-tosylnona-2,3-dien-4-yl)thiophene (5s): Pale yellow oil, 47.5 mg, isolated yield 51%. ^{1}H NMR (400 MHz, DMSO) δ 7.70 (d, *J* = 8.4 Hz, 2H), 7.48 – 7.44 (m, 1H), 7.43 – 7.37 (m, 1H), 7.34 (d, *J* = 8.0 Hz, 2H), 7.25 (d, *J* = 8.8 Hz, 2H), 6.93 (dd, *J* = 4.8, 0.8 Hz, 1H), 6.83 (d, *J* = 8.8 Hz, 2H), 4.67 – 4.65 (m, 2H), 3.72 (s, 3H), 2.36 (s, 3H), 2.34 – 2.18 (m, 2H), 1.44 – 1.30 (m, 2H), 1.26 – 1.15 (m, 4H), 0.78 (t, *J* = 6.8 Hz, 3H). ^{13}C NMR (100 MHz, DMSO) δ 208.6, 158.5, 144.2, 136.7, 136.3, 129.6, 127.9, 127.5, 126.8, 126.7, 126.1, 120.9, 113.8, 105.2, 98.2, 56.8, 55.1, 31.1, 30.3, 27.0, 21.9, 21.1, 13.9. HRMS (ESI): m/z calcd. for $C_{27}H_{31}O_3S_2^+$([M+H]$^+$) = 467.1709, found = 467.1704.

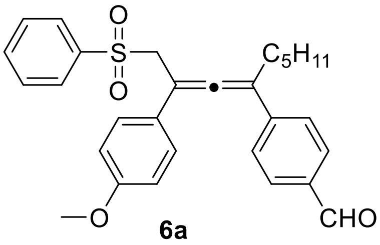

4-(2-(4-methoxyphenyl)-1-(phenylsulfonyl)nona-2,3-dien-4-yl)benzaldehyde (6a): Pale yellow oil, 77.7 mg, isolated yield 82%. ^{1}H NMR (400 MHz, $CDCl_3$) δ 9.91 (s, 1H), 7.79 – 7.67 (m, 4H), 7.48 – 7.43 (m, 1H), 7.41 (d, *J* = 8.4 Hz, 2H), 7.33 (t, *J* = 8.0 Hz, 2H), 7.18 (d, *J* = 8.8 Hz, 2H), 6.73 (d, *J* = 8.8 Hz, 2H), 4.33 – 4.20 (m, 2H), 3.71 (s, 3H), 2.45 – 2.28 (m, 2H), 1.48 – 1.36 (m, 2H), 1.28 – 1.20 (m, 4H), 0.78 (t, *J* = 7.2 Hz, 3H). ^{13}C NMR (100 MHz, $CDCl_3$) δ 210.4, 191.7, 159.2, 142.0, 139.1, 135.2, 133.6, 129.9, 129.0, 128.4, 127.4, 126.9, 125.7, 114.1, 109.9, 99.2, 58.3, 55.3, 31.7, 30.3, 27.5, 22.4, 14.0. HRMS (ESI): m/z calcd. for $C_{29}H_{31}O_4S^+$($[M+H]^+$) = 475.1938, found = 475.1937.

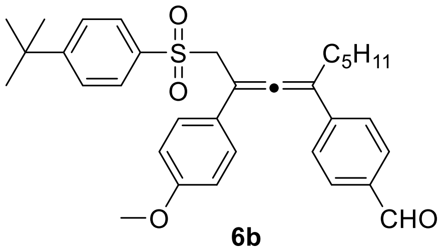

4-(1-((4-(tert-butyl)phenyl)sulfonyl)-2-(4-methoxyphenyl)nona-2,3-dien-4-yl)benzaldehyde (6b): Pale yellow oil, 91.2 mg, isolated yield 86%. ^{1}H NMR (400 MHz, $CDCl_3$) δ 9.98 (s, 1H), 7.83 (d, *J* = 8.4 Hz, 2H), 7.74 (d, *J* = 8.4 Hz, 2H), 7.59 (d, *J* = 8.4 Hz, 2H), 7.39 (d, *J* = 8.8 Hz, 2H), 7.18 (d, *J* = 8.8 Hz, 2H), 6.75 (d, *J* = 8.8 Hz, 2H), 4.31 (s, 2H), 3.76 (s, 3H), 2.57 – 2.37 (m, 2H), 1.63 – 1.46 (m, 2H), 1.39 – 1.30 (m, 4H), 1.28 (s, 9H), 0.85 (t, *J* = 6.8 Hz, 3H). ^{13}C NMR (100 MHz, $CDCl_3$) δ 210.5, 191.7, 159.0, 157.6, 142.0, 136.1, 135.2, 130.0, 128.3, 127.3, 127.0, 126.0, 125.9, 114.0, 109.8, 99.2, 58.2, 55.2, 35.1, 31.7, 31.0, 30.2, 27.5, 22.2, 14.0. HRMS (ESI): m/z calcd. for $C_{33}H_{38}O_4SNa^+$($[M+Na]^+$) = 553.2383, found = 553.2382.

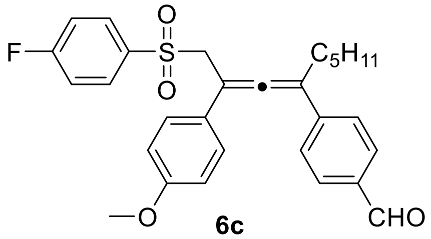

6c

4-(1-((4-fluorophenyl)sulfonyl)-2-(4-methoxyphenyl)nona-2,3-dien-4-yl)benzaldehyde (6c): Pale yellow oil, 64.9 mg, isolated yield 66%. ^{1}H NMR (400 MHz, $CDCl_3$) δ 9.98 (s, 1H), 7.84 – 7.77 (m, 4H), 7.48 (d, J = 8.0 Hz, 2H), 7.23 (d, J = 8.8 Hz, 2H), 7.03 (t, J = 8.4 Hz, 2H), 6.81 (d, J = 8.8 Hz, 2H), 4.39 – 4.29 (m, 2H), 3.79 (s, 3H), 2.53 – 2.39 (m, 2H), 1.55 – 1.44 (m, 2H), 1.35 – 1.27 (m, 4H), 0.85 (t, J = 6.8 Hz, 3H). ^{13}C NMR (100 MHz, $CDCl_3$) δ 210.3, 191.6, 165.7 (d, J = 255.0 Hz), 159.3, 141.9, 135.3, 135.0 (d, J = 3.0 Hz), 131.2 (d, J = 10.0 Hz), 129.9, 127.4, 126.9, 125.5, 116.2 (d, J = 22.0 Hz), 114.2, 110.0, 99.1, 58.4, 55.3, 31.6, 30.4, 27.5, 22.4, 14.0. HRMS (ESI): m/z calcd. for $C_{29}H_{29}FO_4SNa^+$($[M+Na]^+$) = 515.1663, found = 515.1656.

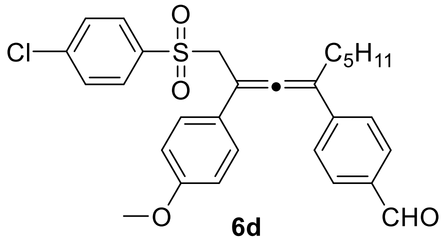

6d

4-(1-((4-chlorophenyl)sulfonyl)-2-(4-methoxyphenyl)nona-2,3-dien-4-yl)benzaldehyde (6d): Pale yellow oil, 70.1 mg, isolated yield 69%. ^{1}H NMR (400 MHz, $CDCl_3$) δ 9.99 (s, 1H), 7.81 (d, J = 8.4 Hz, 2H), 7.71 (d, J = 8.4 Hz, 2H), 7.44 (d, J = 8.4 Hz, 2H), 7.31 (d, J = 8.4 Hz, 2H), 7.23 (d, J = 9.2 Hz, 2H), 6.81 (d, J = 9.2 Hz, 2H), 4.40 – 4.29 (m, 2H), 3.80 (s, 3H), 2.52 – 2.37 (m, 2H), 1.54 – 1.44 (m, 2H), 1.36 – 1.27 (m, 4H), 0.85 (t, J = 7.2 Hz, 3H). ^{13}C NMR (100 MHz, $CDCl_3$) δ 210.2, 191.6, 159.4, 141.9, 140.4, 137.3, 135.3, 130.0, 129.9, 129.3, 127.4, 126.9, 125.4, 114.2, 110.1, 99.1, 58.4, 55.3, 31.6, 30.4, 27.6, 22.4, 14.0. HRMS (ESI): m/z calcd. for $C_{29}H_{30}ClO_4S^+$($[M+H]^+$) = 509.1548, found = 509.1546.

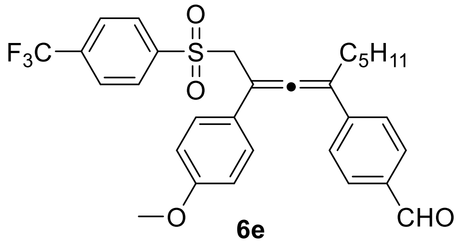

6e

4-(2-(4-methoxyphenyl)-1-((4-(trifluoromethyl)phenyl)sulfonyl)nona-2,3-dien-4-yl)benzaldehyde (6e): Pale yellow oil, 64.0 mg, isolated yield 59%. ^{1}H NMR (400 MHz,

$CDCl_3$) δ 9.98 (s, 1H), 7.92 (d, *J* = 8.0 Hz, 2H), 7.81 (d, *J* = 8.4 Hz, 2H), 7.60 (d, *J* = 8.0 Hz, 2H), 7.50 (d, *J* = 8.8 Hz, 2H), 7.18 (d, *J* = 8.8 Hz, 2H), 6.77 (d, *J* = 8.8 Hz, 2H), 4.43 – 4.33 (m, 2H), 3.77 (s, 3H), 2.53 – 2.36 (m, 2H), 1.58 – 1.44 (m, 2H), 1.35 – 1.26 (m, 4H), 0.85 (t, *J* = 7.2 Hz, 3H). ^{13}C NMR (100 MHz, $CDCl_3$) δ 210.2, 191.6, 159.3, 142.5, 141.7, 135.3, 135.2 (q, *J* = 34.0 Hz), 129.9, 129.0, 127.3, 126.9, 126.0 (q, *J* = 3.0 Hz), 125.3, 123.0 (q, *J* = 272.0 Hz), 114.2, 110.2, 98.8, 58.3, 55.2, 31.6, 30.3, 27.5, 22.4, 13.9. HRMS (ESI): m/z calcd. for $C_{30}H_{29}F_3O_4SNa^+$($[M+Na]^+$) = 565.1631, found = 565.1627.

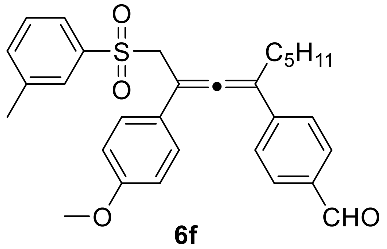

6f

4-(2-(4-methoxyphenyl)-1-(m-tolylsulfonyl)nona-2,3-dien-4-yl)benzaldehyde (6f): Pale yellow oil, 67.3 mg, isolated yield 69%. ^{1}H NMR (400 MHz, $CDCl_3$) δ 9.91 (s, 1H), 7.73 (d, *J* = 8.4 Hz, 2H), 7.57 (d, *J* = 6.8 Hz, 1H), 7.53 (s, 1H), 7.42 (d, *J* = 8.4 Hz, 2H), 7.27 – 7.20 (m, 2H), 7.17 (d, *J* = 9.2 Hz, 2H), 6.73 (d, *J* = 8.8 Hz, 2H), 4.31 – 4.20 (m, 2H), 3.71 (s, 3H), 2.45 – 2.30 (m, 2H), 2.24 (s, 3H), 1.49 – 1.35 (m, 2H), 1.29 – 1.20 (m, 4H), 0.78 (t, *J* = 6.8 Hz, 3H). ^{13}C NMR (100 MHz, $CDCl_3$) δ 210.4, 191.7, 159.2, 142.0, 139.3, 139.0, 135.2, 134.4, 129.9, 128.9, 128.7, 127.4, 126.9, 125.8, 125.4, 114.1, 109.8, 99.3, 58.2, 55.3, 31.7, 30.3, 27.5, 22.4, 21.2, 14.0. HRMS (ESI): m/z calcd. for $C_{30}H_{33}O_4S^+$($[M+H]^+$) = 489.2094, found = 489.2092.

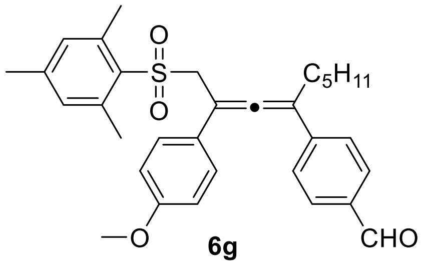

6g

4-(1-(mesitylsulfonyl)-2-(4-methoxyphenyl)nona-2,3-dien-4-yl)benzaldehyde (6g): Pale yellow oil, 83.6 mg, isolated yield 81%. ^{1}H NMR (400 MHz, $CDCl_3$) δ 9.97 (s, 1H), 7.78 (d, *J* = 8.4 Hz, 2H), 7.47 (d, *J* = 8.4 Hz, 2H), 7.32 (d, *J* = 8.8 Hz, 2H), 6.82 (s, 3H), 6.80 (s, 1H), 4.39 – 4.29 (m, 2H), 3.78 (s, 3H), 2.56 (s, 6H), 2.48 – 2.34 (m, 2H), 2.22 (s, 3H), 1.53 – 1.44 (m, 2H), 1.33 – 1.25 (m, 4H), 0.85 (t, *J* = 6.8 Hz, 3H). ^{13}C NMR (100 MHz, $CDCl_3$) δ 210.5, 191.6,

159.2, 143.2, 142.0, 140.3, 135.1, 133.2, 132.1, 129.9, 127.4, 126.9, 125.7, 114.0, 109.5, 99.5, 58.2, 55.2, 31.6, 30.2, 27.6, 22.9, 22.3, 20.9, 14.0. HRMS (ESI): m/z calcd. for $C_{32}H_{36}O_4SNa^+$($[M+Na]^+$) = 539.2227, found = 539.2220.

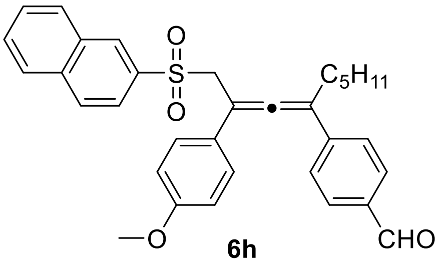

6h

4-(2-(4-methoxyphenyl)-1-(naphthalen-2-ylsulfonyl)nona-2,3-dien-4-yl)benzaldehyde (6h): Pale yellow oil, 77.6 mg, isolated yield 74%. ^{1}H NMR (400 MHz, $CDCl_3$) δ 9.77 (s, 1H), 8.27 (d, *J* = 0.8 Hz, 1H), 7.77 – 7.70 (m, 3H), 7.67 (dd, *J* = 8.8, 1.2 Hz, 1H), 7.58 – 7.53 (m, 1H), 7.52 – 7.47 (m, 1H), 7.45 (d, *J* = 8.4 Hz, 2H), 7.23 – 7.17 (m, 4H), 6.69 (d, *J* = 8.8 Hz, 2H), 4.44 – 4.31 (m, 2H), 3.66 (s, 3H), 2.33 – 2.16 (m, 2H), 1.37 – 1.27 (m, 2H), 1.17 – 1.07 (m, 4H), 0.74 (t, *J* = 6.8 Hz, 3H). ^{13}C NMR (100 MHz, $CDCl_3$) δ 210.1, 191.6, 159.2, 141.8, 135.6, 135.2, 135.0, 132.0, 130.4, 129.7, 129.4, 129.3, 129.2, 127.9, 127.5, 127.4, 126.6, 125.6, 122.8, 114.1, 109.9, 99.4, 58.0, 55.2, 31.6, 30.3, 27.5, 22.3, 14.0. HRMS (ESI): m/z calcd. for $C_{33}H_{33}O_4S^+$($[M+H]^+$) = 525.2094, found = 525.2091.

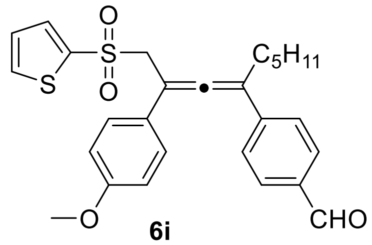

6i

4-(2-(4-methoxyphenyl)-1-(thiophen-2-ylsulfonyl)nona-2,3-dien-4-yl)benzaldehyde (6i): Pale yellow oil, 62.4 mg, isolated yield 65%. ^{1}H NMR (400 MHz, $CDCl_3$) δ 9.91 (s, 1H), 7.75 (d, *J* = 8.4 Hz, 2H), 7.53 – 7.49 (m, 2H), 7.47 (d, *J* = 8.4 Hz, 2H), 7.18 (d, *J* = 8.8 Hz, 2H), 6.90 (dd, *J* = 4.8, 4.0 Hz, 1H), 6.74 (d, *J* = 8.8 Hz, 2H), 4.42 – 4.29 (m, 2H), 3.71 (s, 3H), 2.51 – 2.38 (m, 2H), 1.52 – 1.39 (m, 2H), 1.32 – 1.20 (m, 4H), 0.79 (t, *J* = 7.2 Hz, 3H). ^{13}C NMR (100 MHz, $CDCl_3$) δ 210.6, 191.7, 159.2, 142.0, 140.0, 135.2, 134.7, 134.3, 130.0, 127.7, 127.3, 127.0, 125.7, 114.1, 110.1, 99.2, 59.6, 55.3, 31.7, 30.4, 27.6, 22.4, 14.0. HRMS (ESI): m/z calcd. for $C_{27}H_{28}O_4S_2Na^+$($[M+Na]^+$) = 503.1321, found = 503.1322.

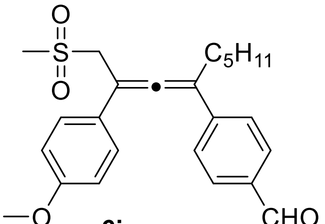

6j

4-(2-(4-methoxyphenyl)-1-(methylsulfonyl)nona-2,3-dien-4-yl)benzaldehyde (6j): Pale yellow oil, 24.7 mg, isolated yield 30%. ^{1}H NMR (400 MHz, $CDCl_3$) δ 9.98 (s, 1H), 7.85 (d, *J* = 8.4 Hz, 2H), 7.63 (d, *J* = 8.4 Hz, 2H), 7.41 (d, *J* = 8.8 Hz, 2H), 6.91 (d, *J* = 9.2 Hz, 2H), 4.27 – 4.14 (m, 2H), 3.81 (s, 3H), 2.79 (s, 3H), 2.67 – 2.60 (m, 2H), 1.69 – 1.54 (m, 2H), 1.42 – 1.27 (m, 4H), 0.86 (t, *J* = 6.8 Hz, 3H). ^{13}C NMR (100 MHz, $CDCl_3$) δ 210.0, 191.6, 159.6, 141.7, 135.4, 130.1, 127.5, 126.9, 125.8, 114.5, 110.6, 99.4, 56.9, 55.4, 40.8, 31.7, 30.4, 27.6, 22.4, 14.0. HRMS (ESI): m/z calcd. for $C_{24}H_{29}O_4S^+$ ($[M+H]^+$) = 413.1781, found = 413.1783.

5. General procedure for 3,4-sulfonylarylation of 1,3-enynes

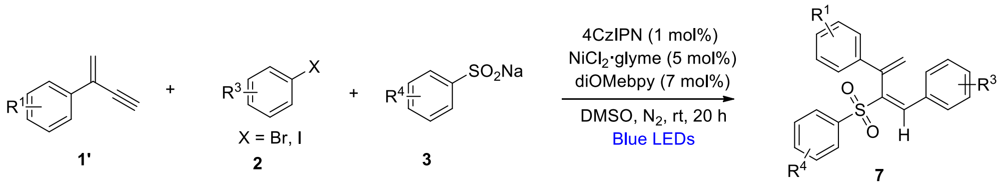

General procedure : A dry reaction tube equipped with a Teflon-coated magnetic stir bar was charged with 4CzIPN (1.6 mg, 0.002 mmol, 1 mol%), $NiCl_2$·glyme (2.2 mg, 0.01 mmol, 5 mol%), diOMebpy (3.0 mg, 0.014 mmol, 7 mol%), aryl halide (0.2 mmol, 1 equiv., if solid), sodium sulfinate (0.24 mmol, 1.2 equiv.) and 1,3-enyne (0.4 mmol, 2 equiv., if solid). It was capped with a rubber septum, evacuated and backfilled with argon. Then, degassed anhydrous DMSO (2.0 mL), aryl halide (0.2 mmol, 1 equiv., if liquid) and 1,3-enyne (0.4 mmol, 2 equiv., if liquid) were added via syringe. The reaction mixture was stirred at room temperature under 30 W blue LEDs irradiation for 20 hours. After the reaction completion, the reaction mixture was diluted with EtOAc, then washed with brine, dried over Na_2SO_4, filtered, and concentrated under reduced pressure. The residue was purified by column chromatography on silica gel (hexane/EtOAc) to afford the corresponding 1,3-diene products.

The analytical data of the products are summarized below.

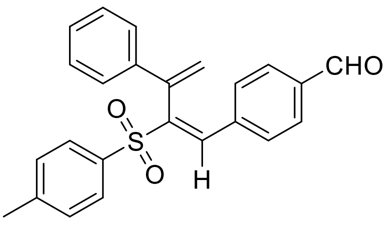

7a

(*E*)-4-(3-phenyl-2-tosylbuta-1,3-dien-1-yl)benzaldehyde (7a): Light yellow solid, 54.3 mg, isolated yield 70%. ^{1}H NMR (400 MHz, $CDCl_3$) δ 9.94 (s, 1H), 8.03 (s, 1H), 7.74 (d, J = 8.4 Hz, 2H), 7.70 – 7.66 (m, 4H), 7.22 – 7.14 (m, 7H), 5.91 (s, 1H), 5.18 (s, 1H), 2.36 (s, 3H). ^{13}C NMR (100 MHz, $CDCl_3$) δ 191.4, 144.5, 144.3, 139.2, 138.4, 136.7, 136.7, 136.3, 135.1, 130.7, 129.7, 129.4, 129.1, 128.4, 128.4, 126.1, 120.9, 21.6. HRMS (ESI): m/z calcd. for $C_{24}H_{21}O_3S^+$([M+H]$^+$) = 389.1206, found = 389.1205.

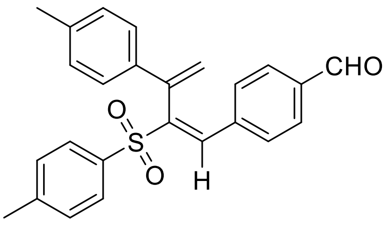

7b

4-(3-(*p*-tolyl)-2-tosylbuta-1,3-dien-1-yl)benzaldehyde (7b): Pale yellow oil, 56.3 mg, isolated yield 70%, *E/Z* = 14:1. ^{1}H NMR (400 MHz, $CDCl_3$) δ 9.93 (s, 1H), 8.01 (s, 1H), 7.76 – 7.64 (m, 6H), 7.19 (d, J = 8.0 Hz, 2H), 7.13 (d, J = 8.2 Hz, 2H), 6.96 (d, J = 8.0 Hz, 2H), 5.85 (s, 1H), 5.56 (s, 0.07H of Z isomer), 5.40 (s, 0.07H of Z isomer), 5.06 (s, 1H), 2.37 (s, 3H), 2.26 (s, 3H). ^{13}C NMR (100 MHz, $CDCl_3$) δ 191.4, 144.4, 139.1, 138.5, 138.4, 136.7, 136.6, 135.1, 133.4, 130.7, 129.6, 129.4, 129.2, 129.1, 126.0, 119.8, 21.6, 21.1. HRMS (ESI): m/z calcd. for $C_{25}H_{23}O_3S^+$([M+H]$^+$) = 403.1362, found = 403.1360.

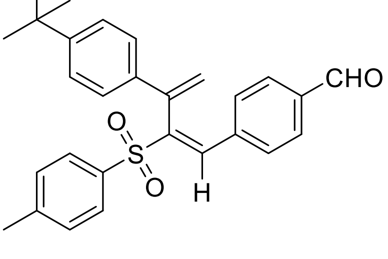

7c

(*E*)-4-(3-(4-(tert-butyl)phenyl)-2-tosylbuta-1,3-dien-1-yl)benzaldehyde (7c): Pale yellow oil, 48.8 mg, isolated yield 55%. ^{1}H NMR (400 MHz, $CDCl_3$) δ 9.94 (s, 1H), 8.02 (s, 1H), 7.78 – 7.70 (m, 4H), 7.62 (d, J = 8.0 Hz, 2H), 7.14 (s, 6H), 5.87 (s, 1H), 5.11 (s, 1H), 2.34 (s, 3H), 1.24 (s, 9H). ^{13}C NMR (100 MHz, $CDCl_3$) δ 191.3, 151.5, 144.6, 144.2, 139.0, 138.5, 136.7, 136.5, 135.3, 133.2, 130.7,

129.7, 129.3, 129.1, 125.8, 125.3, 120.1, 34.4, 31.1, 21.5. HRMS (ESI): m/z calcd. for $C_{28}H_{29}O_3S^+$([M+H]$^+$) = 445.1832, found = 445.1836.

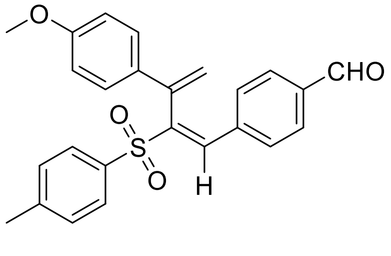

7d

(*E*)-4-(3-(4-methoxyphenyl)-2-tosylbuta-1,3-dien-1-yl)benzaldehyde (7d): Pale yellow oil, 64.4 mg, isolated yield 77%. ^{1}H NMR (400 MHz, $CDCl_3$) δ 9.94 (s, 1H), 8.00 (s, 1H), 7.74 (d, *J* = 8.4 Hz, 2H), 7.72 – 7.63 (m, 4H), 7.19 (t, *J* = 8.2 Hz, 4H), 6.68 (d, *J* = 8.8 Hz, 2H), 5.77 (s, 1H), 4.99 (s, 1H), 3.74 (s, 3H), 2.37 (s, 3H). ^{13}C NMR (100 MHz, $CDCl_3$) δ 191.4, 159.8, 144.5, 144.4, 138.6, 138.5, 136.7, 136.5, 135.2, 130.7, 129.6, 129.4, 129.1, 128.8, 127.4, 118.6, 113.8, 55.2, 21.6. HRMS (ESI): m/z calcd. for $C_{25}H_{23}O_4S^+$([M+H]$^+$) = 419.1312, found = 419.1313.

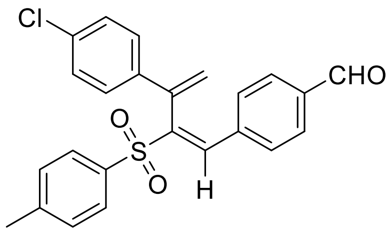

7e

(*E*)-4-(3-(4-chlorophenyl)-2-tosylbuta-1,3-dien-1-yl)benzaldehyde (7e): Pale yellow solid, 48.1 mg, isolated yield 57%. ^{1}H NMR (400 MHz, $CDCl_3$) δ 9.95 (s, 1H), 8.02 (s, 1H), 7.75 (d, *J* = 8.4 Hz, 2H), 7.66 (d, *J* = 8.4 Hz, 4H), 7.20 (d, *J* = 7.6 Hz, 2H), 7.16 – 7.09 (m, 4H), 5.90 (s, 1H), 5.18 (s, 1H), 2.39 (s, 3H). ^{13}C NMR (100 MHz, $CDCl_3$) δ 191.3, 144.8, 143.8, 138.2, 138.2, 137.0, 136.9, 135.0, 134.8, 134.4, 130.6, 129.7, 129.5, 129.1, 128.7, 127.4, 121.4, 21.6. HRMS (ESI): m/z calcd. for $C_{24}H_{20}ClO_3S^+$([M+H]$^+$) = 423.0816, found = 423.0817.

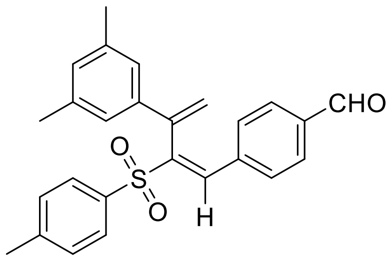

7f

(*E*)-4-(3-(3,5-dimethylphenyl)-2-tosylbuta-1,3-dien-1-yl)benzaldehyde (7f): Pale yellow oil, 52.4 mg, isolated yield 63%. ^{1}H NMR (400 MHz, $CDCl_3$) δ 9.94 (s, 1H), 8.01 (s, 1H), 7.77 – 7.64 (m, 6H), 7.16 (d, *J* = 7.6 Hz, 2H), 6.81 – 6.77 (m, 3H), 5.88 (d, *J* = 0.8 Hz, 1H), 5.15 (d, *J* = 0.8 Hz, 1H), 2.36 (s, 3H), 2.15

(d, J = 0.8 Hz, 6H). ^{13}C NMR (100 MHz, $CDCl_3$) δ 191.4, 144.7, 144.3, 139.3, 138.5, 137.8, 136.7, 136.7, 136.1, 135.5, 130.7, 130.1, 129.6, 129.3, 129.0, 124.0, 120.6, 21.5, 21.1. HRMS (ESI): m/z calcd. for $C_{26}H_{25}O_3S^+$([M+H]$^+$) = 417.1519, found = 417.1520.

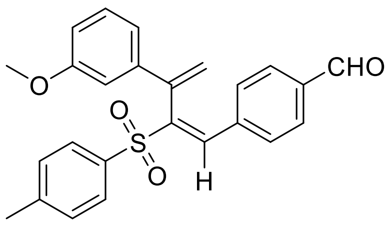

7g

(*E*)-4-(3-(3-methoxyphenyl)-2-tosylbuta-1,3-dien-1-yl)benzaldehyde (7g): Pale yellow oil, 24.2 mg, isolated yield 29%. ^{1}H NMR (400 MHz, $CDCl_3$) δ 9.94 (s, 1H), 8.02 (s, 1H), 7.75 (d, J = 8.4 Hz, 2H), 7.72 – 7.63 (m, 4H), 7.18 (d, J = 8.0 Hz, 2H), 7.07 (t, J = 8.4 Hz, 1H), 6.85 – 6.80 (m, 1H), 6.75 – 6.68 (m, 2H), 5.91 (s, 1H), 5.19 (s, 1H), 3.68 (s, 3H), 2.37 (s, 3H). ^{13}C NMR (100 MHz, $CDCl_3$) δ 191.4, 159.6, 144.5, 144.3, 139.1, 138.5, 137.7, 136.8, 135.2, 130.7, 129.7, 129.5, 129.4, 129.1, 121.3, 118.8, 113.8, 112.0, 55.1, 21.6. HRMS (ESI): m/z calcd. for $C_{25}H_{23}O_4S^+$([M+H]$^+$) = 419.1312, found = 419.1311.

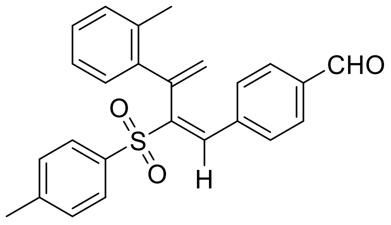

7h

(*E*)-4-(3-(*o*-tolyl)-2-tosylbuta-1,3-dien-1-yl)benzaldehyde (7h): Pale yellow oil, 32.2 mg, isolated yield 40%. ^{1}H NMR (400 MHz, $CDCl_3$) δ 9.98 (s, 1H), 7.97 (s, 1H), 7.86 – 7.76 (m, 4H), 7.50 (d, J = 8.0 Hz, 2H), 7.14 – 7.02 (m, 4H), 6.89 – 6.80 (m, 2H), 5.64 (d, J = 1.0 Hz, 1H), 5.56 (d, J = 1.0 Hz, 1H), 2.35 (s, 3H), 2.34 (s, 3H). ^{13}C NMR (100 MHz, $CDCl_3$) δ 191.3, 146.5, 144.1, 138.9, 138.3, 137.0, 136.8, 136.6, 136.1, 136.0, 131.3, 130.5, 129.6, 129.3, 128.6, 128.4, 127.8, 126.3, 125.6, 21.5, 21.0. HRMS (ESI): m/z calcd. for $C_{25}H_{23}O_3S^+$([M+H]$^+$) = 403.1362, found = 403.1360.

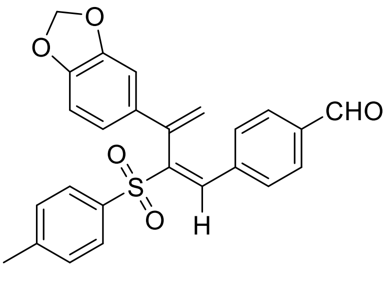

7i

(*E*)-4-(3-(benzo[*d*][1,3]dioxol-5-yl)-2-tosylbuta-1,3-dien-1-yl)benzaldehyde (7i): Pale yellow oil, 45.8 mg, isolated yield 53%. ^{1}H NMR (400 MHz, $CDCl_3$) δ 9.95 (s, 1H), 7.99 (s, 1H), 7.80 – 7.73 (m, 2H), 7.72 – 7.60 (m, 4H), 7.22 (d, *J* = 8.0 Hz, 2H), 6.77 – 6.68 (m, 2H), 6.57 (d, *J* = 8.0 Hz, 1H), 5.90 (s, 2H), 5.75 (s, 1H), 4.99 (s, 1H), 2.38 (s, 3H). ^{13}C NMR (100 MHz, $CDCl_3$) δ 191.3, 147.9, 144.5, 144.4, 138.7, 138.3, 136.8, 136.7, 135.1, 130.7, 130.5, 129.7, 129.4, 129.1, 120.6, 119.2, 108.1, 106.2, 101.2, 21.6. HRMS (ESI): m/z calcd. for $C_{25}H_{21}O_5S^+$([M+H]$^+$) = 433.1104, found = 433.1108.

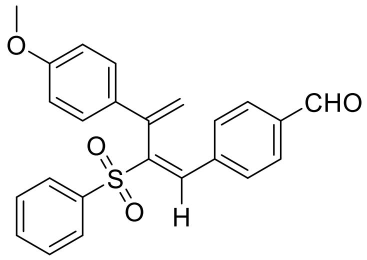

7j

(*E*)-4-(3-(4-methoxyphenyl)-2-(phenylsulfonyl)buta-1,3-dien-1-yl)benzaldehyde (7j): Pale yellow oil, 46.1 mg, isolated yield 57%. ^{1}H NMR (400 MHz, $CDCl_3$) δ 9.94 (s, 1H), 8.03 (s, 1H), 7.81 (dd, *J* = 8.4, 1.2 Hz, 2H), 7.76 – 7.69 (m, 4H), 7.55 – 7.50 (m, 1H), 7.41 (t, *J* = 7.6Hz, 2H), 7.17 (d, *J* = 8.8 Hz, 2H), 6.68 (d, *J* = 8.8 Hz, 2H), 5.79 (s, 1H), 5.00 (s, 1H), 3.73 (s, 3H). ^{13}C NMR (100 MHz, $CDCl_3$) δ 191.4, 159.8, 144.2, 138.5, 138.4, 138.2, 137.0, 136.8, 133.4, 130.7, 129.7, 129.1, 128.8, 128.6, 127.4, 118.8, 113.9, 55.2. HRMS (ESI): m/z calcd. for $C_{24}H_{20}O_4SNa^+$([M+Na]$^+$) = 427.0975, found = 427.0972.

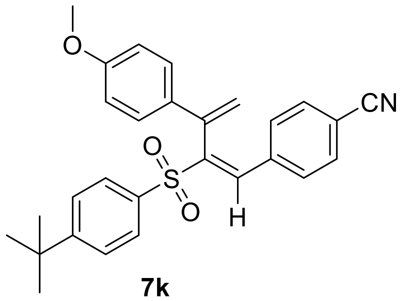

7k

(*E*)-4-(2-((4-(tert-butyl)phenyl)sulfonyl)-3-(4-methoxyphenyl)buta-1,3-dien-1-yl)benzaldehyde (7k): Pale yellow oil, 46.0 mg, isolated yield 50%. ^{1}H NMR (400 MHz, $CDCl_3$) δ 7.89 (s, 1H), 7.60 (d, *J* = 8.6 Hz, 2H), 7.57 (d, *J* = 8.4 Hz, 2H), 7.45 (d, *J* = 8.4 Hz, 2H), 7.29 (d, *J* = 8.6 Hz, 2H), 7.00 (d, *J* = 8.8 Hz, 2H), 6.55 (d, *J* = 8.8 Hz, 2H), 5.75 (s, 1H), 5.04 (s, 1H), 3.65 (s, 3H), 1.20 (s, 9H). ^{13}C

NMR (100 MHz, $CDCl_3$) δ 159.7, 157.5, 145.1, 138.3, 137.2, 135.5, 134.9, 132.2, 130.5, 129.0, 128.5, 127.3, 125.8, 118.8, 118.2, 113.8, 113.3, 55.1, 35.1, 31.0. HRMS (ESI): m/z calcd. for $C_{28}H_{28}NO_3S^+$($[M+H]^+$) = 458.1784, found = 458.1786.

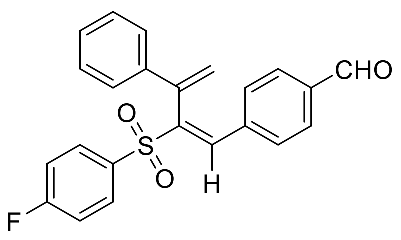

7l

(*E*)-4-(2-((4-fluorophenyl)sulfonyl)-3-phenylbuta-1,3-dien-1-yl)benzaldehyde (7l): Pale yellow oil, 49.3 mg, isolated yield 63%. ^{1}H NMR (400 MHz, $CDCl_3$) δ 9.88 (s, 1H), 7.98 (s, 1H), 7.77 – 7.58 (m, 6H), 7.17 – 7.03 (m, 5H), 6.97 (t, J = 8.6 Hz, 2H), 5.89 (s, 1H), 5.18 (s, 1H). ^{13}C NMR (100 MHz, $CDCl_3$) δ 191.3, 165.6 (d, J = 255.0 Hz), 143.8, 139.1, 138.1, 137.2, 136.9, 136.0, 134.2 (d, J = 3.0 Hz), 131.9 (d, J = 10.0 Hz), 130.7, 129.7, 128.6, 128.6, 126.0, 121.3, 116.1 (d, J = 22.0 Hz). HRMS (ESI): m/z calcd. for $C_{23}H_{17}FO_3SNa^+$($[M+Na]^+$) = 415.0775, found = 415.0773.

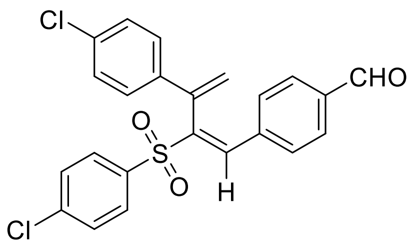

7m

(*E*)-4-(3-(4-chlorophenyl)-2-((4-chlorophenyl)sulfonyl)buta-1,3-dien-1-yl)benzaldehyde (7m): Pale yellow solid, 40.7 mg, isolated yield 46%. ^{1}H NMR (400 MHz, CDCl3) δ 9.96 (s, 1H), 8.04 (s, 1H), 7.77 (d, J = 8.4 Hz, 2H), 7.72 (d, J = 8.8 Hz, 2H), 7.68 (d, J = 8.4 Hz, 2H), 7.40 (d, J = 8.8 Hz, 2H), 7.16 (s, 4H), 5.93 (s, 1H), 5.18 (s, 1H). ^{13}C NMR (100 MHz, $CDCl_3$) δ 191.2, 143.1, 140.5, 138.1, 137.9, 137.8, 137.1, 136.5, 134.8, 134.5, 130.7, 130.5, 129.8, 129.2, 128.8, 127.3, 121.7. HRMS (ESI): m/z calcd. for $C_{23}H_{16}Cl_2O_3SNa^+$($[M+Na]^+$) = 465.0089, found = 465.0093.

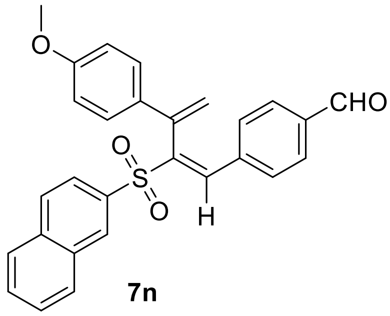

7n

(*E*)-4-(3-(4-methoxyphenyl)-2-(naphthalen-2-ylsulfonyl)buta-1,3-dien-1-yl)benzaldehyde (7n): Pale yellow oil, 61.7 mg, isolated yield 68%. ^{1}H NMR (400 MHz, $CDCl_3$) δ 9.95 (s, 1H), 8.31 (d, *J* = 2.0 Hz, 1H), 8.09 (s, 1H), 7.85 (d, *J* = 8.4 Hz, 3H), 7.79 – 7.71 (m, 5H), 7.66 – 7.54 (m, 2H), 7.15 (d, *J* = 9.2 Hz, 2H), 6.55 (d, *J* = 8.8 Hz, 2H), 5.75 (s, 1H), 4.98 (s, 1H), 3.61 (s, 3H). ^{13}C NMR (100 MHz, $CDCl_3$) δ 191.4, 159.7, 144.4, 138.7, 138.4, 137.1, 136.9, 135.1, 135.1, 131.9, 131.2, 130.7, 129.7, 129.4, 129.2, 129.1, 128.6, 127.8, 127.4, 127.4, 123.5, 118.8, 113.8, 55.1. HRMS (ESI): m/z calcd. for $C_{28}H_{23}O_4S^+$([M+H]$^+$) = 455.1312, found = 455.1311.

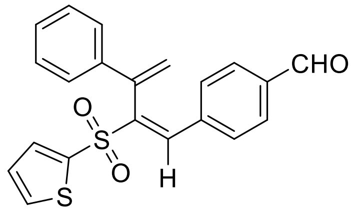

7o

4-(3-phenyl-2-(thiophen-2-ylsulfonyl)buta-1,3-dien-1-yl)benzaldehyde (7o): Pale yellow oil, 45.6 mg, isolated yield 60%, *E/Z* = 11:1. ^{1}H NMR (400 MHz, CDCl3) δ 9.94 (s, 1H), 8.05 (s, 1H), 7.77 – 7.67 (m, 4H), 7.60 (dd, *J* = 5.0, 1.3 Hz, 1H), 7.53 (dd, *J* = 3.8, 1.3 Hz, 1H), 7.25 – 7.14 (m, 5H), 6.96 (dd, *J* = 4.9, 3.8 Hz, 1H), 6.03 (s, 1H), 5.71 (s, 0.09H of Z isomer), 5.57 (s, 0.09H of Z isomer), 5.39 (s, 1H). ^{13}C NMR (100 MHz, $CDCl_3$) δ 191.3, 144.2, 139.2, 138.9, 138.3, 136.9, 136.8, 136.3, 135.6, 134.6, 130.8, 129.7, 128.6, 127.7, 125.9, 121.3. HRMS (ESI): m/z calcd. for $C_{21}H_{16}O_3S_2Na^+$([M+Na]$^+$) = 403.0433, found = 403.0427.

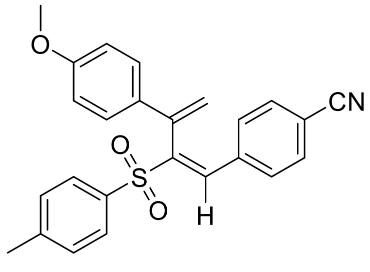

7p

(*E*)-4-(3-(4-methoxyphenyl)-2-tosylbuta-1,3-dien-1-yl)benzonitrile (7p): Pale yellow oil, 62.3 mg, isolated yield 75%. ^{1}H NMR (400 MHz, $CDCl_3$) δ 7.95 (s, 1H), 7.66 (d, *J* = 8.0 Hz, 2H), 7.62 (d, *J* = 8.0 Hz, 2H), 7.51 (d, *J* = 8.4 Hz, 2H), 7.20 (d, *J* = 8.0 Hz, 2H), 7.15 (d, *J* = 8.8 Hz, 2H), 6.68 (d, *J* = 8.8 Hz, 2H), 5.77 (s, 1H), 4.97 (s, 1H), 3.74 (s, 3H), 2.37 (s, 3H). ^{13}C NMR (100 MHz, $CDCl_3$) δ 159.9, 145.0,

144.6, 138.3, 137.1, 135.7, 134.9, 132.1, 130.5, 129.4, 129.1, 128.5, 127.4, 118.6, 118.2, 113.9, 113.2, 55.2, 21.5. HRMS (ESI): m/z calcd. for $C_{25}H_{22}NO_3S^+$([M+H]$^+$) = 416.1315, found = 416.1311.

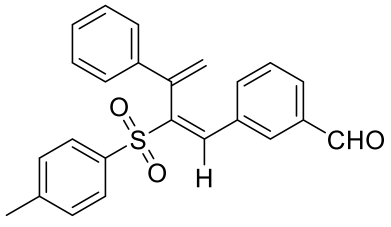

7q

(*E*)-3-(3-phenyl-2-tosylbuta-1,3-dien-1-yl)benzaldehyde (7q): Pale yellow oil, 39.6 mg, isolated yield 51%. ^{1}H NMR (400 MHz, $CDCl_3$) δ 9.87 (s, 1H), 8.04 (s, 1H), 8.00 (t, *J* = 1.6 Hz, 1H), 7.77 (dd, *J* = 7.6, 2.0 Hz, 2H), 7.69 (d, *J* = 8.4 Hz, 2H), 7.40 (d, *J* = 8.0 Hz, 1H), 7.25 – 7.07 (m, 7H), 5.93 (s, 1H), 5.21 (s, 1H), 2.37 (s, 3H). ^{13}C NMR (100 MHz, $CDCl_3$) δ 191.4, 144.5, 143.1, 139.3, 136.7, 136.5, 136.4, 135.5, 135.3, 133.7, 131.7, 130.6, 129.5, 129.3, 129.1, 128.4, 128.3, 126.2, 121.0, 21.5. HRMS (ESI): m/z calcd. for $C_{24}H_{20}O_3SNa^+$([M+Na]$^+$) = 411.1025, found = 411.1027.

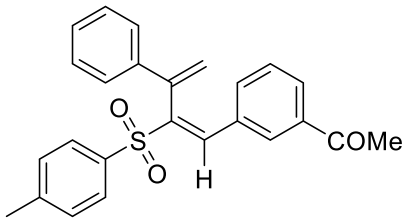

7r

(*E*)-1-(3-(3-phenyl-2-tosylbuta-1,3-dien-1-yl)phenyl)ethan-1-one (7r): Pale yellow oil, 45.6 mg, isolated yield 57%. ^{1}H NMR (400 MHz, $CDCl_3$) δ 7.92 – 7.91 (m, 1H), 7.86 (s, 1H), 7.70 – 7.65 (m, 1H), 7.54 – 7.50 (m, 3H), 7.16 (t, *J* = 7.8 Hz, 1H), 7.10 – 7.06 (m, 2H), 7.04 – 6.95 (m, 5H), 5.76 (s, 1H), 4.98 (s, 1H), 2.23 (s, 3H), 2.19 (s, 3H). ^{13}C NMR (100 MHz, $CDCl_3$) δ 197.3, 144.4, 142.4, 139.4, 137.3, 137.3, 136.3, 135.4, 134.4, 133.2, 130.4, 129.5, 129.4, 129.1, 128.9, 128.5, 128.4, 126.1, 120.6, 26.4, 21.6. HRMS (ESI): m/z calcd. for $C_{25}H_{22}O_3SNa^+$([M+Na]$^+$) = 425.1182, found = 425.1183.

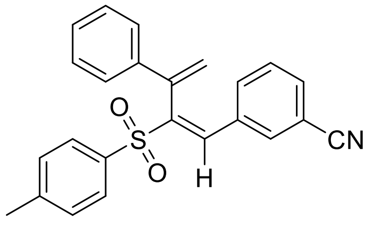

7s

(*E*)-3-(3-phenyl-2-tosylbuta-1,3-dien-1-yl)benzonitrile (7s): Pale yellow oil, 34.7 mg, isolated yield 45%. ^{1}H NMR (400 MHz, $CDCl_3$) δ 7.95 (s, 1H), 7.79 (s, 1H), 7.72 (d, *J* = 8.0 Hz, 1H), 7.67 (d, *J* = 8.3

Hz, 2H), 7.53 (dt, J = 7.8, 1.3 Hz, 1H), 7.34 (t, J = 7.9 Hz, 1H), 7.22 – 7.09 (m, 7H), 5.92 (s, 1H), 5.21 (s, 1H), 2.37 (s, 3H). ^{13}C NMR (100 MHz, CDCl$_3$) δ 144.6, 144.1, 139.0, 136.2, 135.5, 135.0, 134.1, 134.0, 133.3, 133.1, 129.5, 129.4, 129.2, 128.5, 128.5, 126.1, 121.2, 118.0, 113.0, 21.6. HRMS (ESI): m/z calcd. for $C_{24}H_{20}NO_2S^+$([M+H]$^+$) = 386.1209, found = 386.1210.

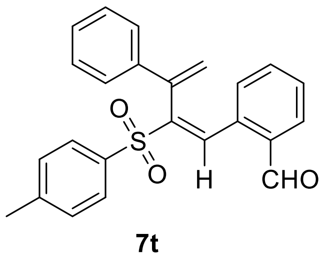

7t

(*E*)-2-(3-phenyl-2-tosylbuta-1,3-dien-1-yl)benzaldehyde (7t): Pale yellow oil, 52.0 mg, isolated yield 67%. ^{1}H NMR (400 MHz, CDCl$_3$) δ 9.80 (s, 1H), 7.97 – 7.93 (m, 2H), 7.70 (dd, J = 7.4, 1.4 Hz, 2H), 7.62 (d, J = 8.0 Hz, 2H), 7.32 (t, J = 7.6 Hz, 1H), 7.18 – 7.02 (m, 7H), 5.86 (s, 1H), 5.14 (s, 1H), 2.30 (s, 3H). ^{13}C NMR (100 MHz, CDCl$_3$) δ 191.4, 144.5, 143.1, 139.3, 136.7, 136.5, 136.4, 135.5, 135.3, 133.7, 131.7, 130.6, 129.5, 129.3, 129.1, 128.4, 128.3, 126.2, 121.0, 21.5. HRMS (ESI): m/z calcd. for $C_{24}H_{20}O_3SNa^+$([M+Na]$^+$) = 411.1025, found = 411.1022.

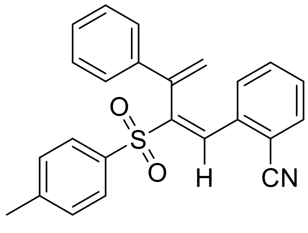

7u

(*E*)-2-(3-phenyl-2-tosylbuta-1,3-dien-1-yl)benzonitrile (7u): Pale yellow oil, 44.7 mg, isolated yield 58%. ^{1}H NMR (400 MHz, CDCl$_3$) δ 8.26 (s, 1H), 7.85 – 7.70 (m, 2H), 7.62 – 7.50 (m, 2H), 7.36 – 7.27 (m, 2H), 7.24 (d, J = 8.0 Hz, 2H), 7.17 – 7.03 (m, 5H), 5.85 (s, 1H), 5.32 (s, 1H), 2.38 (s, 3H). ^{13}C NMR (100 MHz, CDCl$_3$) δ 146.5, 144.7, 138.7, 136.9, 136.4, 135.0, 134.4, 132.9, 132.3, 129.6, 129.6, 129.2, 128.9, 128.4, 128.2, 126.1, 121.5, 116.9, 113.4, 21.5. HRMS (ESI): m/z calcd. for $C_{24}H_{19}NO_2SNa^+$([M+Na]$^+$) = 408.1029, found = 408.1028.

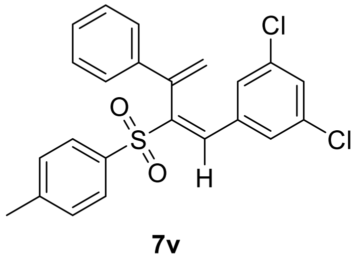

7v

(*E*)-1,3-dichloro-5-(3-phenyl-2-tosylbuta-1,3-dien-1-yl)benzene (7v): Pale yellow oil, 47.1 mg, isolated yield 55%. ^{1}H NMR (400 MHz, CDCl$_3$) δ 7.84 (s, 1H), 7.68 (d, J = 8.4 Hz, 2H), 7.37 (d, J = 2.0 Hz, 2H), 7.25 – 7.08 (m,

8H), 5.90 (s, 1H), 5.23 (s, 1H), 2.37 (s, 3H). ^{13}C NMR (100 MHz, $CDCl_3$) δ 144.6, 144.2, 139.0, 136.4, 135.5, 135.2, 135.0, 129.7, 129.5, 129.2, 128.4, 128.4, 128.2, 126.2, 121.3, 21.6. HRMS (ESI): m/z calcd. for $C_{23}H_{18}Cl_2O_2SNa^+$([M+Na]$^+$) = 451.0297, found = 451.0294.

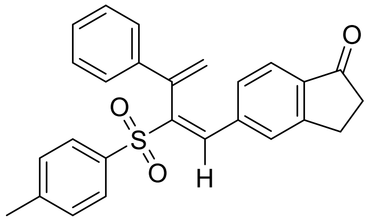

7w

(*E*)-5-(3-phenyl-2-tosylbuta-1,3-dien-1-yl)-2,3-dihydro-1H-inden-1-one (7w): Pale yellow oil, 53.0 mg, isolated yield 64%. ^{1}H NMR (400 MHz, $CDCl_3$) δ 8.04 (s, 1H), 7.67 (d, *J* = 8.4 Hz, 2H), 7.61 (s, 1H), 7.59 – 7.51 (m, 2H), 7.22 – 7.11 (m, 7H), 5.90 (s, 1H), 5.21 (s, 1H), 3.04 – 2.99 (m, 2H), 2.66 – 2.60 (m, 2H), 2.36 (s, 3H). ^{13}C NMR (100 MHz, $CDCl_3$) δ 206.1, 154.9, 144.5, 143.9, 139.3, 138.7, 137.8, 137.2, 136.5, 135.2, 129.4, 129.1, 128.4, 128.4, 128.3, 126.2, 123.6, 121.1, 36.3, 25.6, 21.5. HRMS (ESI): m/z calcd. for $C_{26}H_{23}O_3S^+$([M+H]$^+$) = 415.1362, found = 415.1361.

6. Scale-up syntheses and further transformations

6.1 Scale-up reactions

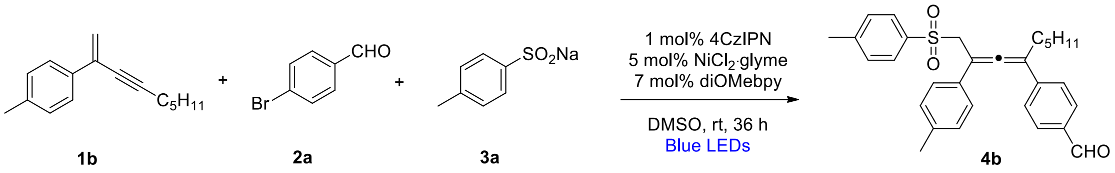

A dry reaction tube equipped with a Teflon-coated magnetic stir bar was charged with 4CzIPN (24 mg, 0.03 mmol, 1 mol%), $NiCl_2$·glyme (33 mg, 0.15 mmol, 5 mol%), diOMebpy (45 mg, 0.21 mmol, 7 mol%), 4-bromobenzaldehyde **2a** (555 mg, 3.0 mmol, 1 equiv.), sodium *p*-methylbenzene sulfinate **3a** (641 mg, 3.6 mmol, 1.2 equiv.) and 1,3-enyne **1b** (1.272 g, 6.0 mmol, 2 equiv.). It was capped with a rubber septum, evacuated and backfilled with argon. Then, degassed DMSO (30 mL) was added via syringe. The reaction mixture was stirred at room temperature under 30 W blue LEDs irradiation for 36 hours. After the reaction completion, the reaction mixture was diluted with EtOAc, then washed with brine, dried over Na_2SO_4, filtered, and concentrated under reduced pressure. The residue was purified by column chromatography on silica gel (hexane/EtOAc) to afford the corresponding product **4b** (0.991 g, 70% yield) as light yellow solid.

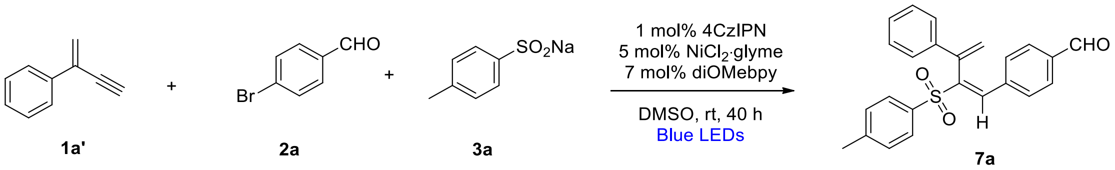

A dry reaction tube equipped with a Teflon-coated magnetic stir bar was charged with 4CzIPN (32 mg, 0.04 mmol, 1 mol%), $NiCl_2$·glyme (44 mg, 0.2 mmol, 5 mol%), diOMebpy (60 mg, 0.28 mmol, 7 mol%), 4-bromobenzaldehyde **2a** (712 mg, 4.0 mmol, 1 equiv.) and sodium *p*-methylbenzene sulfinate **3a** (854 mg, 4.8 mmol, 1.2 equiv.). It was capped with a rubber septum, evacuated and backfilled with argon. Then, degassed

DMSO (40 mL) and 1,3-enyne **1a'** (1.024 g, 8 mmol, 2 equiv.) were added via syringe. The reaction mixture was stirred at room temperature under 30 W blue LEDs irradiation for 40 hours. After the reaction completion, the reaction mixture was diluted with EtOAc, then washed with brine, dried over Na_2SO_4, filtered, and concentrated under reduced pressure. The residue was purified by column chromatography on silica gel (hexane/EtOAc) to afford the corresponding product **7a** (0.950 g, 61% yield) as light yellow solid.

6.2 Further transformations

Further transformations of α-allenyl sulfones:

(a)

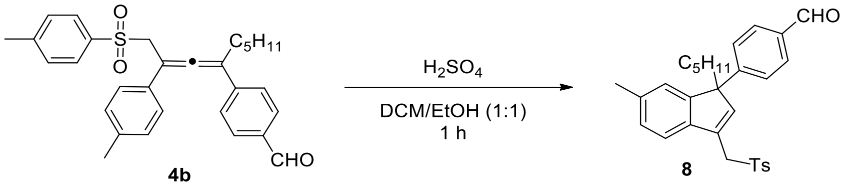

Procedure [2a]: 4-(2-(*p*-tolyl)-1-tosylnona-2,3-dien-4-yl)benzaldehyde **4b** (42.2 mg, 0.1 mmol, 1 equiv.) was dissolved in 1 mL DCM/EtOH (1:1), then 1 mL conc. H_2SO_4 was added dropwise to the solution. The reaction mixture was stirred at room temperature for 1h. After quenching with 10 mL H_2O, the mixture was extracted with ethyl acetate 3 times. The combined organic layers were washed with brine, dried over Na_2SO_4 and concentrated under vacuum. The residue was purified by column chromatography to afford desired product **8** in 95% yield.

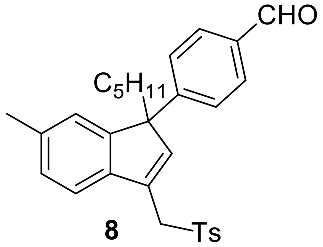

8

4-(6-methyl-1-pentyl-3-(tosylmethyl)-1*H*-inden-1-yl)benzaldehyde (**8**): Pale yellow oil, 44.8 mg, isolated yield 95%. ^{1}H NMR (400 MHz, $CDCl_3$) δ 9.88 (s, 1H), 7.66 (d, *J* = 8.4 Hz, 2H), 7.56 (d, *J* = 8.4 Hz, 2H), 7.24 (d, *J* = 8.4 Hz, 2H), 7.11 – 7.04 (m, 3H), 6.96 (d, *J* = 7.6 Hz, 1H), 6.91 (s, 1H), 6.17 (s, 1H), 4.35 – 4.24 (m, 2H), 2.29 (s, 3H), 2.26 (s, 3H), 2.21 – 2.08 (m, 1H), 1.84 – 1.72 (m, 1H), 1.22 – 1.00 (m, 6H), 0.74 (t, *J* = 6.8 Hz, 3H). ^{13}C NMR (100

MHz, $CDCl_3$) δ 191.7, 150.7, 149.4, 145.3, 144.7, 138.9, 136.2, 135.2, 134.9, 130.9, 129.8, 129.5, 128.5, 127.9, 127.1, 123.9, 120.2, 60.3, 55.7, 37.0, 32.3, 24.8, 22.4, 21.6, 14.0. HRMS (ESI): m/z calcd. for $C_{30}H_{32}O_3SNa^+$($[M+Na]^+$) = 495.1964, found = 495.1957.

(b)

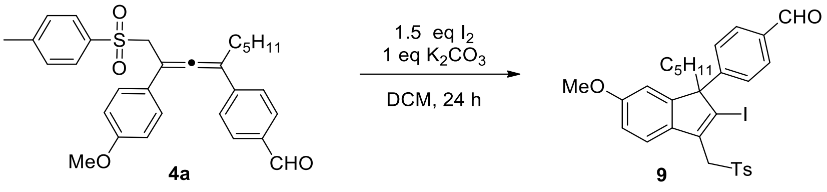

Procedure [2a]: To a dry sealed tube charged with a magnetic stir bar was added K_2CO_3 (13.8 mg, 0.1 mmol, 1 equiv), 4-(2-(4-methoxyphenyl)-1-tosylnona-2,3-dien-4-yl)benzaldehyde **4a** (49 mg, 0.1 mmol, 1 equiv), I_2 (38 mg, 0.15 mmol, 1.5 equiv), and 1mL DCM under inert atmosphere. The reaction mixture was stirred at room temperature for 24 h. After quenching with saturated sodium thiosulfate solution, the mixture was extracted with ethyl acetate 3 times. The combined organic layers were washed with brine, dried over Na_2SO_4 and concentrated under vacuum. The residue was purified by column chromatography to afford desired product **9** in 38% yield.

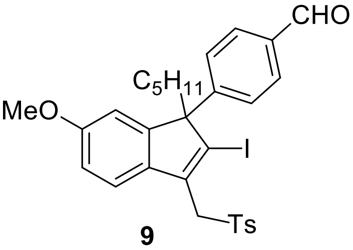

9

4-(2-iodo-6-methoxy-1-pentyl-3-(tosylmethyl)-1H-inden-1-yl)benzaldehyde (**9**): Pale yellow oil, 23.3 mg, isolated yield 38%. ^{1}H NMR (400 MHz, $CDCl_3$) δ 9.89 (s, 1H), 7.73 –7.61 (m, 4H), 7.39 (d, *J* = 8.4 Hz, 1H), 7.14 (d, *J* = 8.0 Hz, 2H), 7.06 (d, *J* = 8.4 Hz, 2H), 6.73 (dd, *J* = 8.4, 2.4 Hz, 1H), 6.51 (d, *J* = 2.0 Hz, 1H), 4.42 (s, 2H), 3.68 (s, 3H), 2.32 (s, 3H), 2.11 – 2.03 (m, 2H), 1.18 –1.04 (m, 6H), 0.75 (t, *J* = 7.2 Hz, 3H). ^{13}C NMR (125 MHz, $CDCl_3$) δ 191.7, 156.0, 152.3, 148.5, 145.1, 136.5, 135.7, 135.2, 134.9, 129.9, 129.7, 128.9, 127.5, 121.6, 118.9, 112.1, 110.2, 64.5, 58.7, 55.5, 34.8, 31.9, 22.3, 22.2, 21.7, 14.0. HRMS (ESI): m/z calcd. for $C_{30}H_{31}IO_4SNa^+$($[M+Na]^+$) = 637.0880, found = 637.0875.

(c)

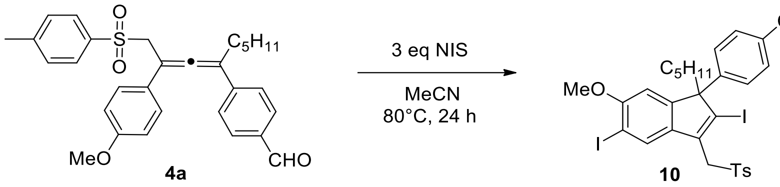

Procedure[2a]: To a 25 ml pressure tube charged with a magnetic stir bar was added NIS (67.5 mg, 0.3 mmol, 3 equiv), 1 mL CH_3CN, and 4-(2-(4-methoxyphenyl)-1-tosylnona-2,3-dien-4-yl)benzaldehyde **4a** (49 mg, 0.1 mmol, 1 equiv) under inert atmosphere. The reaction mixture was stirred at 80 °C for 24 hours. After completion of the reaction, the mixture was concentrated under vacuum. The residue was purified by flash column chromatography to afford desired product **10** in 95% yield.

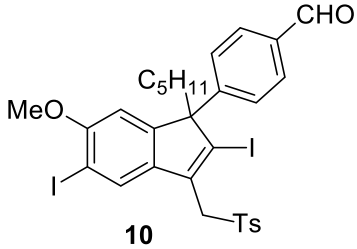

10

4-(2,5-diiodo-6-methoxy-1-pentyl-3-(tosylmethyl)-1H-inden-1-yl)benzaldehyde (**10**): Pale yellow oil, 70.3 mg, isolated yield 95%. ^{1}H NMR (400 MHz, $CDCl_3$) δ 9.91 (s, 1H), 7.71 – 7.62 (m, 4H), 7.36 (s, 1H), 7.18 (d, *J* = 8.0 Hz, 2H), 7.10 (d, *J* = 8.4 Hz, 2H), 6.40 (s, 1H), 4.37 (s, 2H), 3.68 (s, 3H), 2.36 (s, 3H), 2.21 – 2.04 (m, 2H), 1.22 – 1.07 (m, 6H), 0.77 (t, *J* = 6.8 Hz, 3H). ^{13}C NMR (100 MHz, $CDCl_3$) δ 191.6, 157.0, 152.8, 147.8, 145.5, 136.4, 135.9, 135.5, 135.4, 130.8, 130.0, 129.9, 128.8, 127.5, 120.6, 106.3, 84.6, 64.8, 58.4, 56.6, 34.8, 31.9, 22.3, 22.2, 21.9, 14.0. HRMS (ESI): m/z calcd. for $C_{30}H_{30}I_2O_4SNa^+$ ($[M+Na]^+$) = 762.9846, found = 762.9840.

(d)

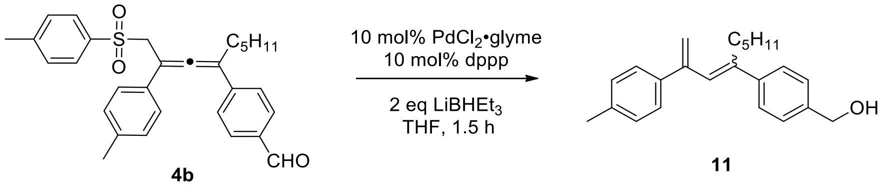

Procedure[4]: To a flame-dried 15 mL vial equipped with a stir bar was added $PdCl_2$ • glyme (5.2 mg, 0.02 mmol, 10 mol%) and 1,2-bis(diphenylphosphino)propane (8.0 mg, 0.02 mmol, 10 mol%) under argon, 0.5 mL DCM was added and the reaction mixture was stirred for 10 mins. The solvent was removed by sparging with argon, then a solution of 4-(2-(*p*-tolyl)-1-tosylnona-2,3-dien-4-yl)benzaldehyde **4b** (94.4 mg, 0.2 mmol, 1 equiv.) in 2 mL THF was added. Then 2 mL Super-Hydride® solution (1 M, 2 equiv.) was added dropwise, and the reaction was stirred for 1.5 h. After quenching by NaOH (2.5%), the mixture was extracted with DCM for three times. The combined organic layers were dried over Na_2SO_4 and concentrated under vacuum. The residue was purified by column chromatography to afford desired product **11** in 51% yield.

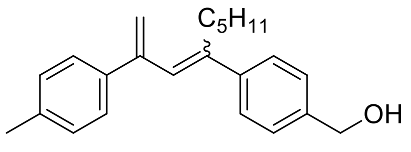

11

(4-(2-(*p*-tolyl)nona-1,3-dien-4-yl)phenyl)methanol (**11**): Pale yellow oil, 32.6 mg, isolated yield 51%. ^{1}H NMR (400 MHz, $CDCl_3$) δ 7.22 (d, *J* = 8.0 Hz, 2H), 7.13 – 7.04 (m, 4H), 7.01 (d, *J* = 8.0 Hz, 2H), 6.11 (d, *J* = 1.2 Hz, 1H), 5.16 (d, *J* = 1.6 Hz, 1H), 4.69 (t, *J* = 1.6 Hz, 1H), 4.54 (s, 2H), 2.41 (t, *J* = 6.4 Hz, 2H), 2.25 (s, 3H), 1.38 – 1.29 (m, 2H), 1.28 – 1.19 (m, 4H), 0.81 (t, *J* = 7.2 Hz, 3H). ^{13}C NMR (100 MHz, $CDCl_3$) δ 144.5, 144.5, 140.5, 139.0, 138.0, 137.1, 128.8, 128.4, 126.7, 126.6, 126.3, 115.2, 77.3, 77.0, 76.7, 65.3, 39.5, 31.4, 27.7, 22.4, 21.1, 14.1. HRMS (ESI): m/z calcd. for $C_{23}H_{28}ONa^+$($[M+Na]^+$) = 343.2032, found = 343.2031.

Utilization of tert-butyl bromide as radical precursor:

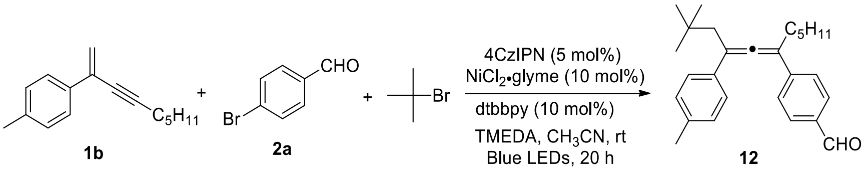

Procedure: A dry reaction tube equipped with a Teflon-coated magnetic stir bar was charged with 4CzIPN (3.9 mg, 0.005 mmol, 5 mol%), $NiCl_2$·glyme (2.2 mg, 0.01

mmol, 10 mol%), dtbbpy (2.7 mg, 0.01 mmol, 10 mol%), **2a** (0.2 mmol, 36 mg, 2 equiv.) and 1,3-enyne **1b** (0.1 mmol, 22 mg, 1 equiv.). It was capped with a rubber septum, evacuated and backfilled with argon. Then, degassed anhydrous CH_3CN (1.5 mL), tert-butyl bromide (0.25 mmol, 28 ul, 2.5 equiv.) and TMEDA (0.3 mmol, 45 ul, 3 equiv.) were added via syringe. The reaction mixture was stirred at room temperature under 30 W blue LEDs irradiation for 20 hours. After the reaction completion, the reaction mixture was filtered and concentrated under reduced pressure. The residue was purified by column chromatography on silica gel (hexane/EtOAc) to afford the product **12** in 67% yield.

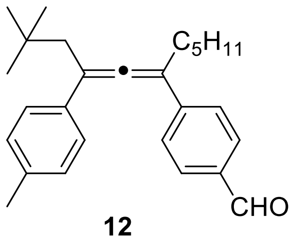

12

4-(2,2-dimethyl-4-(*p*-tolyl)undeca-4,5-dien-6-yl)benzaldehyde (12): Pale yellow liquld, 25.0 mg, isolated yield 67%. ^{1}H NMR (400 MHz, CD_3CN) δ 9.94 (s, 1H), 7.83 (d, J = 8.8 Hz, 2H), 7.61 (d, J = 8.4 Hz, 2H), 7.37 (d, J = 8.4 Hz, 2H), 7.14 (d, J = 8.0 Hz, 2H), 2.60 – 2.55 (m, 2H), 2.52 (s, 2H), 2.30 (s, 3H), 1.61 – 1.53 (m, 2H), 1.37 – 1.26 (m, 4H), 0.91 (s, 9H), 0.83 (t, J = 7.0 Hz, 3H). ^{13}C NMR (100 MHz, CD_3CN) δ 209.5, 192.9, 144.7, 137.8, 135.9, 130.7, 130.1, 127.5, 127.3, 108.2, 107.4, 44.5, 32.7, 32.4, 31.0, 30.3, 28.6, 23.2, 21.0, 14.3. HRMS (ESI): m/z calcd. for $C_{27}H_{34}ONa^+$($[M+Na]^+$) = 397.2507, found = 397.2506.

Further transformations of 1,3-dienyl sulfones:

(a)

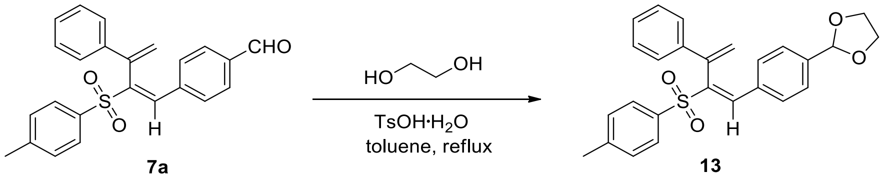

Procedure: In a round bottom flask were introduced substrate **7a** (388 mg, 1.0 mmol, 1 equiv.), toluene (20 mL), *p*-toluenesulfonic acid monohydrate (19 mg, 0.1 mmol, 10 mol%) and ethylene glycol (0.11 mL, 2.0 mmol, 2 equiv.). The reaction mixture was refluxed overnight. After cooling to room temperature, the mixture was washed with

brine, dried over Na_2SO_4 and concentrated under vacuum. The product **13** was obtained in 92% yield.

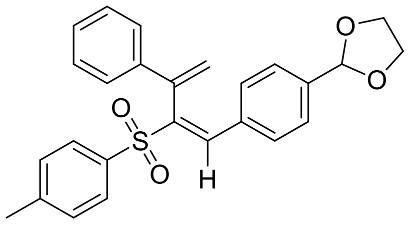

13

(*E*)-2-(4-(3-phenyl-2-tosylbuta-1,3-dien-1-yl)phenyl)-1,3-dioxolane (13): Pale yellow solid, 396.0 mg, isolated yield 92%. ^{1}H NMR (400 MHz, $CDCl_3$) δ 7.99 (s, 1H), 7.64 (d, J = 8.0 Hz, 2H), 7.58 (d, J = 8.4 Hz, 2H), 7.36 (d, J = 8.4 Hz, 2H), 7.26 – 7.24 (m, 2H), 7.17 – 7.14 (m, 5H), 5.88 (d, J = 0.8 Hz, 1H), 5.74 (s, 1H), 5.09 (d, J = 0.8 Hz, 1H), 4.08 – 3.97 (m, 4H), 2.35 (s, 3H). ^{13}C NMR (100 MHz, $CDCl_3$) δ 144.1, 141.5, 140.0, 139.6, 138.0, 136.5, 135.6, 133.5, 130.5, 129.3, 129.0, 128.4, 128.2, 126.7, 126.2, 120.7, 103.0, 65.3, 21.5. HRMS (ESI): m/z calcd. for $C_{26}H_{24}O_4NaS^+$([M+Na]$^+$) = 455.1288, found = 455.1286.

(b)

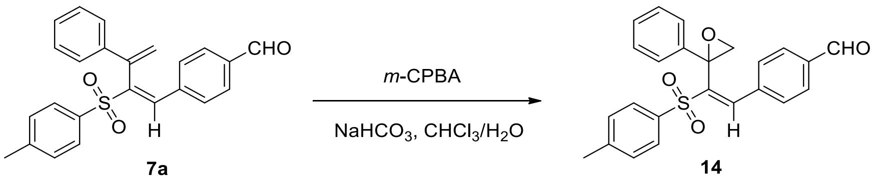

Procedure: To a 10 mL tube were added substrate **7a** (38.8 mg, 0.1 mmol, 1 equiv.), *m*-CPBA (25.9 mg, 0.15 mmol, 1.5 equiv.), $NaHCO_3$ (12.6 mg, 0.15 mmol, 1.5 equiv.) and $CHCl_3/H_2O$ (v/v = 1/1, 1.0 mL). The resulting mixture was stirred at room temperature until **7a** was consumed as indicated by TLC. Then, the reaction was quenched by adding a solution of $Na_2S_2O_3$ in water and the mixture was extracted by DCM for three times. The combined organic layers were washed with brine, dried over Na_2SO_4 and concentrated under vacuum. The residue was purified by column chromatography to afford the corresponding product **14** in 73% yield.

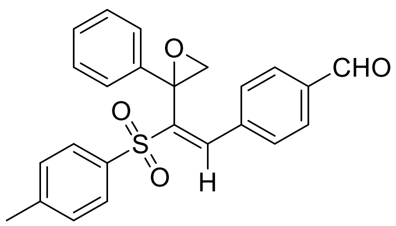

14

(*E*)-4-(2-(2-phenyloxiran-2-yl)-2-tosylvinyl)benzaldehyde (14): Pale yellow oil, 29.5 mg, isolated yield 73%. ^{1}H NMR (400 MHz, $CDCl_3$) δ 9.99 (s, 1H), 8.17 (s, 1H), 7.83 (d, *J* = 8.4 Hz, 2H), 7.76 (d, *J* = 8.4 Hz, 2H), 7.65 (d, *J* = 8.4 Hz, 2H), 7.28 – 7.26 (m, 5H), 7.24 – 7.21 (m, 2H), 3.03 (d, *J* = 5.2 Hz, 1H), 2.84 (d, *J* = 5.6 Hz, 1H), 2.39 (s, 3H).^{13}C NMR (100 MHz, $CDCl_3$) δ 191.2, 144.6, 142.3, 141.4, 137.7, 137.6, 137.1, 136.4, 131.0, 129.8, 129.6, 128.6, 128.4, 125.7, 59.3, 56.0, 21.6. HRMS (ESI): m/z calcd. for $C_{24}H_{20}O_4NaS^+$($[M+Na]^+$) = 427.0975, found = 427.0978.

(c)

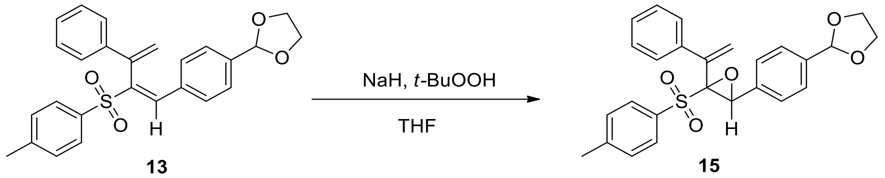

Procedure: A 10 mL tube was charged with anhydrous THF (2.0 mL) and oil free NaH (19.2 mg, 0.8 mmol, 4 equiv., washed with hexane). The mixture was cooled to 0 °C and then *t*-BuOOH (0.16 mL, 0.8 mmol, 4 equiv., 5.0 M in decane) was added. After stirring at room temperature for 30 mins, the resulting solution was cooled to 0 °C and a solution of **13** (86.4 mg, 0.2 mmol, 1 equiv.) in anhydrous THF (2.0 mL) was added dropwise. The reaction mixture was stirred at 0 °C until **13** was consumed as indicated by LCMS. Then, the reaction was quenched by adding a solution of $Na_2S_2O_3$ in water and the mixture was extracted by ethyl acetate. The combined organic layers were washed with brine, dried over Na_2SO_4 and concentrated under vacuum. The residue was purified by column chromatography to afford the corresponding product **15** in 76% yield.

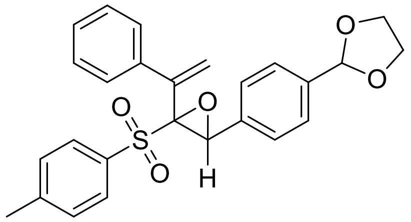

15

2-(4-(3-(1-phenylvinyl)-3-tosyloxiran-2-yl)phenyl)-1,3-dioxolane (15): Colorless oil, 68.4 mg, isolated yield 76%. ^{1}H NMR (400 MHz, $CDCl_3$) δ 7.70 (d, *J* = 8.4 Hz, 2H), 7.35 (d, *J* = 8.0 Hz, 2H), 7.28 (d, *J* = 8.4 Hz, 2H), 7.24 (d, *J* = 8.4 Hz, 2H), 7.15 – 7.12 (m, 5H), 5.72 – 5.71 (m, 2H), 5.17 (s, 1H), 5.14 (s, 1H), 4.07 – 3.97 (m, 4H), 2.43 (s, 3H). ^{13}C NMR (100 MHz, $CDCl_3$) δ 145.3, 138.7, 136.2, 135.5, 132.9, 132.3, 130.0, 129.4, 128.1, 128.0, 127.0, 126.5, 126.3, 123.5, 103.1, 79.9, 65.2, 62.2, 21.7. HRMS (ESI): m/z calcd. for $C_{26}H_{24}O_5NaS^+$($[M+Na]^+$) = 471.1237, found = 471.1235.

(d)

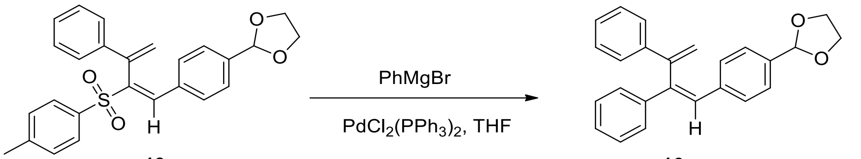

Procedure: Under argon atmosphere, **13** (43.2 mg, 0.1 mmol, 1 equiv.), $PdCl_2(PPh_3)_2$ (7.0 mg, 0.01 mmol, 0.1 equiv.), and anhydrous THF (2.0 mL) were charged in a flask and cooled to 0 ºC. Then, phenyl magnesium bromide (3M in Et_2O, 0.14 mL, 0.4 mmol, 4 equiv.) was added dropwise and stirred at 40 ºC until **13** was consumed as indicated by TLC. Then, the reaction was quenched by water and the mixture was extracted by ethyl acetate. The combined organic layers were washed with brine, dried over Na_2SO_4 and concentrated under vacuum. The residue was purified by column chromatography to afford the corresponding product **16** in 96% yield.

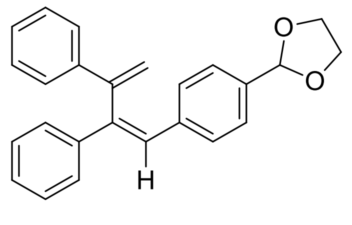

16

(*Z*)-2-(4-(2,3-diphenylbuta-1,3-dien-1-yl)phenyl)-1,3-dioxolane (16): Pale yellow oil, 34.0 mg, isolated yield 96%. ^{1}H NMR (400 MHz, $CDCl_3$) δ 7.47 – 7.44 (m, 4H), 7.42 – 7.40 (m, 2H), 7.26 – 7.14 (m, 8H), 7.00 (s, 1H), 5.82 (d, *J* = 1.6 Hz, 1H), 5.68 (s, 1H), 5.21 (d, *J* = 1.2 Hz, 1H),

4.03 – 4.01 (m, 2H), 3.95 – 3.92 (m, 2H). ^{13}C NMR (100 MHz, $CDCl_3$) δ 146.0, 142.5, 141.2, 138.6, 138.1, 136.5, 129.1, 129.0, 128.6, 128.4, 127.9, 127.6, 126.6, 126.5, 126.2, 116.9, 103.6, 65.3. HRMS (ESI): m/z calcd. for $C_{25}H_{22}O_2Na^+$($[M+Na]^+$) = 377.1512, found = 377.1516.

(e)

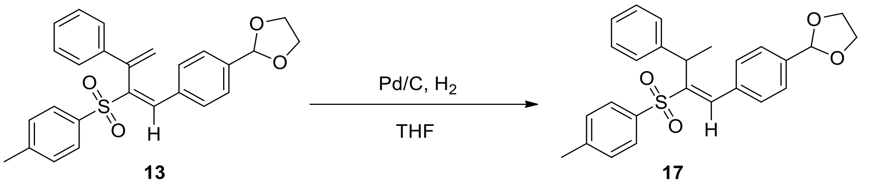

Procedure: A 10 mL tube was charged with **13** (43.2 mg, 0.1 mmol), THF (2.0 mL) and 10 % Pd/C (40 mg). Then the tube was filled with hydrogen gas using a balloon. The mixture was stirred at room temperature until the starting material was consumed as indicated by LCMS. After filtration, concentrated, the residue was purified by column chromatography to afford the corresponding product **17** in 56% yield.

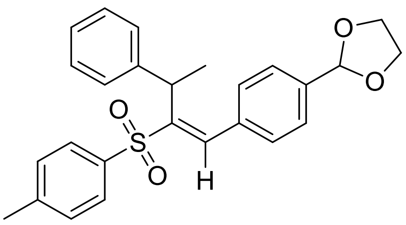

17

(*E*)-2-(4-(3-phenyl-2-tosylbut-1-en-1-yl)phenyl)-1,3-dioxolane (17): Pale yellow solid, 24.5 mg, isolated yield 56%. ^{1}H NMR (500 MHz, $CDCl_3$) δ 8.01 (s, 1H), 7.52 (d, J = 8.5 Hz, 2H), 7.39 (d, J = 8.5 Hz, 2H), 7.19 (d, J = 8.0 Hz, 2H), 7.16 (d, J = 8.0 Hz, 2H), 7.05 – 7.04 (m, 3H), 6.99 – 6.98 (m, 2H), 5.77 (s, 1H), 4.39 (q, J = 7.5 Hz, 1H), 4.12 – 4.09 (m, 2H), 4.04 – 4.01 (m, 2H), 2.38 (s, 3H), 1.55 (d, J = 7.5 Hz, 3H). ^{13}C NMR (125 MHz, $CDCl_3$) δ 147.6, 143.6, 140.6, 139.6, 138.6, 137.7, 134.6, 129.5, 129.0, 128.0, 127.9, 127.5, 126.5, 126.2, 103.1, 65.3, 37.0, 21.5, 18.0. HRMS (ESI): m/z calcd. for $C_{26}H_{26}O_4NaS^+$($[M+Na]^+$) = 457.1444, found = 457.1448.

7. Mechanistic experiments

7.1 Stern-Volmer quenching experiment

Stern-Volmer quenching experiments were carried out using stock solutions of photocatalyst 4CzIPN (1 x 10^{-5} M), sodium *p*-methylbenzenesulfinate **3a** (6 x 10^{-2} M), 4-bromobenzaldehyde **2a** (6 x 10^{-2} M) and 1,3-enyne **1a** (6 x 10^{-2} M) in anhydrous DMSO. In a gas-tight 3 mL quartz cuvette, samples were obtained by mixing a fix volume of the stock solution of photocatalyst 4CzIPN and variable amount of the substrate. Before the measurements, N_2 was bubbled into the solution for 10 mins. Then, samples were irradiated at 400 nm and emission spectras were recorded from wavelength 450 nm to 800 nm for each sample, as shown in the following figures.

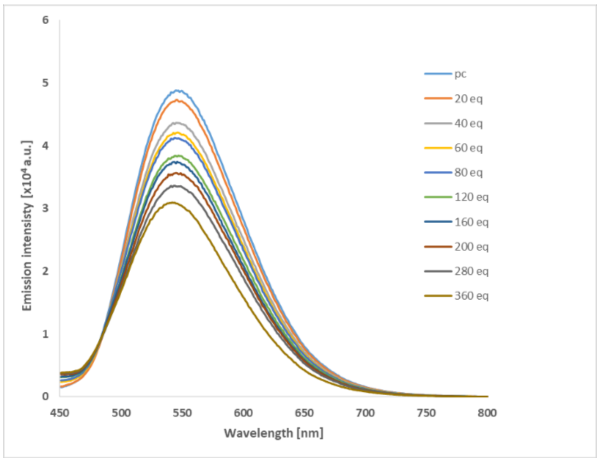

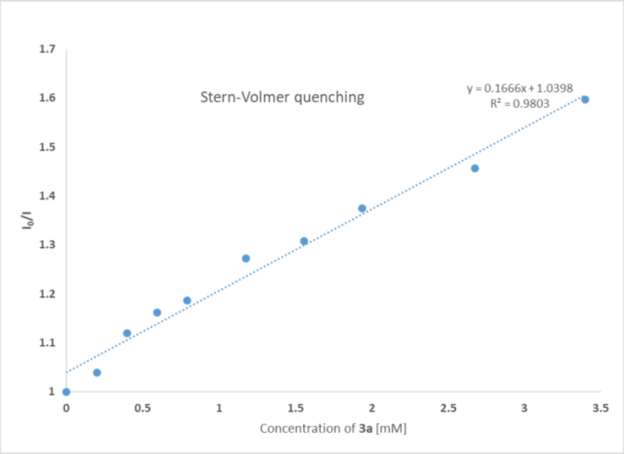

Figure S1: Fluorescence quenching of 4CzIPN with sodium *p*-methylbenzenesulfinate **3a**.

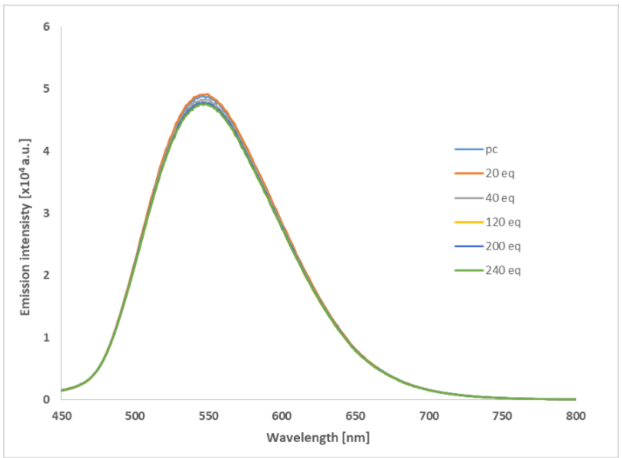

Figure S2: Fluorescence quenching of 4CzIPN with 4-bromobenzaldehyde **2a**.

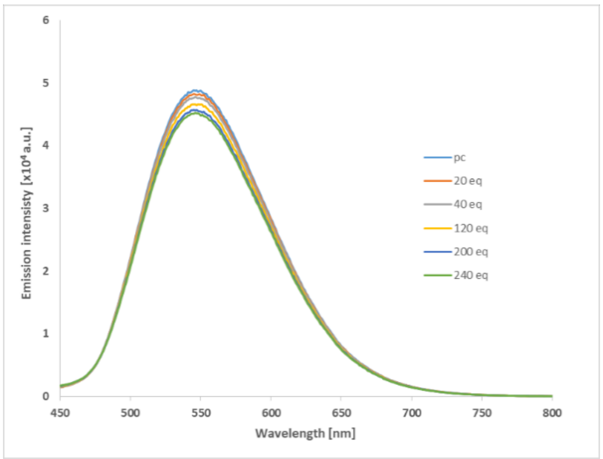

Figure S3: Fluorescence quenching of 4CzIPN with 1,3-enyne **1a**.

These experiments clearly show that only sodium *p*-methylbenzenesulfinate **3a** is an effective quencher of the fluorescence of 4CzIPN.

7.2 Radical trapping experiment

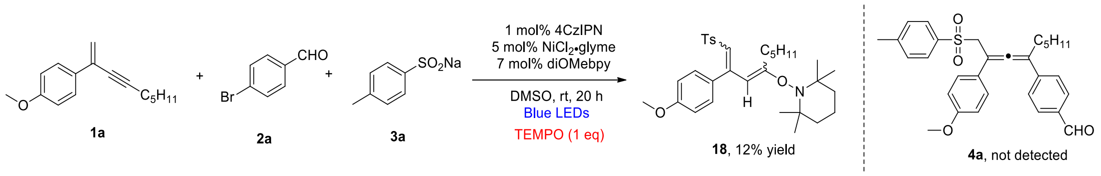

A dry reaction tube equipped with a Teflon-coated magnetic stir bar was charged with

4CzIPN (8 mg, 0.01 mmol, 1 mol%), $NiCl_2$·glyme (11 mg, 0.05 mmol, 5 mol%), diOMebpy (15 mg, 0.07 mmol, 7 mol%), 4-bromobenzaldehyde **2a** (185 mg, 1.0 mmol, 1 equiv.), sodium *p*-methylbenzene sulfinate **3a** (214 mg, 1.2 mmol, 1.2 equiv.), TEMPO (156 mg, 1.0 mmol, 1 equiv.) and 1,3-enyne **1a** (456 mg, 2.0 mmol, 2 equiv.). It was capped with a rubber septum, evacuated and backfilled with argon. Then, degassed DMSO (10 mL) was added via syringe. The reaction mixture was stirred at room temperature under 30 W blue LEDs irradiation for 20 hours. Then the reaction mixture was diluted with EtOAc, then washed with brine, dried over Na_2SO_4, filtered, and concentrated under reduced pressure. Product **4a** was not detected by crude ^{1}H NMR analysis. The residue was purified by column chromatography on silica gel (hexane/EtOAc) to afford the diene product **18** (65.8 mg) in 12% yield.

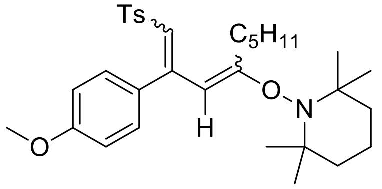

1-((2-(4-methoxyphenyl)-1-tosylnona-1,3-dien-4-yl)oxy)-2,2,6,6-tetramethylpiperidine (18): Colorless oil, 65.8 mg, isolated yield 12%. ^{1}H NMR (400 MHz, $CDCl_3$) δ 7.89 (d, J = 8.4 Hz, 2H), 7.31 (d, J = 8.0 Hz, 2H), 7.20 (d, J = 8.8 Hz, 2H), 6.99 (d, J = 1.2 Hz, 1H), 6.84 (d, J = 8.8 Hz, 2H), 5.96 (d, J = 1.2 Hz, 1H), 3.82 (s, 3H), 2.43 (s, 3H), 1.68 – 1.66 (m, 2H), 1.62 – 1.58 (m, 4H), 1.32 – 1.26 (m, 4H), 1.17 – 1.15 (m, 12H), 1.08 – 1.04 (m, 2H), 0.93 – 0.89 (m, 2H), 0.78 – 0.75 (m, 3H); ^{13}C NMR (100 MHz, $CDCl_3$) δ 170.0, 160.6, 154.1, 143.3, 140.8, 134.4, 129.7, 129.5, 127.1, 121.4, 114.0, 99.6, 60.5, 55.5, 39.7, 32.4, 31.8, 31.5, 26.9, 22.3, 21.7, 20.9, 17.2, 14.0. HRMS (ESI): m/z calcd. for $C_{32}H_{46}NO_4S^+$([M+H]$^+$) = 540.3142, found = 540.3138.

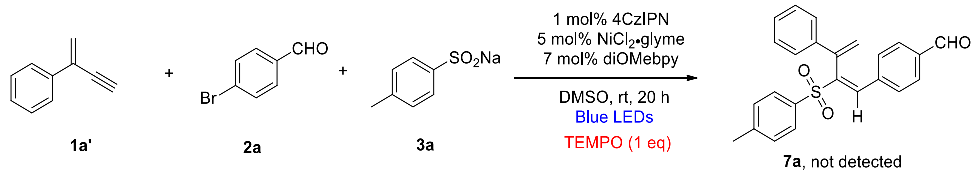

A dry reaction tube equipped with a Teflon-coated magnetic stir bar was charged with 4CzIPN (1.6 mg, 0.002 mmol, 1 mol%), $NiCl_2$·glyme (2.2 mg, 0.01 mmol, 5 mol%), diOMebpy (3.0 mg, 0.014 mmol, 7 mol%), 4-bromobenzaldehyde **2a** (37 mg, 0.2 mmol, 1 equiv.) and sodium *p*-methylbenzene sulfinate **3a** (43 mg, 0.24 mmol, 1.2 equiv.). It was capped with a rubber septum, evacuated and backfilled with argon. Then, degassed DMSO (2.0 mL) and 1,3-enyne **1a'** (74 mg, 0.4 mmol, 2 equiv.) were added via syringe. The reaction mixture was stirred at room temperature under 30 W blue LEDs irradiation for 20 hours. Then the reaction mixture was diluted with EtOAc, then washed with brine, dried over Na_2SO_4, filtered, and concentrated under reduced pressure. Product **7a** was not detected by crude ^{1}H NMR analysis.

7.3 Control experiments

Synthesis of Ni-A[5]

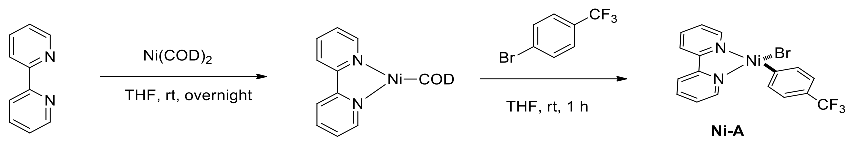

In an argon filled glove box, a 50 mL round bottom flash containing a stirring bar was charged with $Ni(COD)_2$ (276 mg, 1.0 mmol, 1.0 equiv.), 2,2'-dipyridy (156 mg, 1.0 mmol, 1.0 equiv.) and dry THF (10 mL). The dark purple mixture was stirred overnight ar room temperature. Then 4-bromobenzotrifluoride (1.4 mL, 10 mmol, 10 equiv.) was added and stirred for additional 1 h. Dry pentane (20 mL) was added to the orange mixture and filtered. The resulting precipitate was washed with dry pentane (3 x 10 mL) and dried under vacumm to obtain **Ni-A** as an orange solid, which was used without further purification.

Stoichiometric experiment

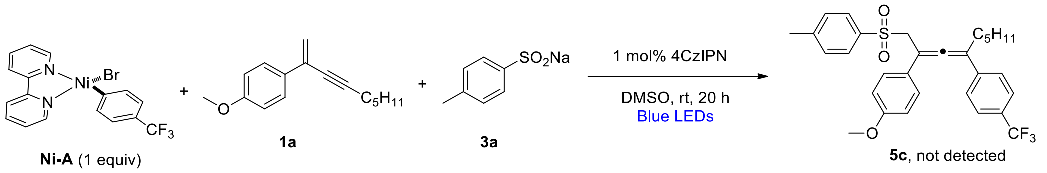

A dry reaction tube equipped with a Teflon-coated magnetic stir bar was charged with 4CzIPN (1.6 mg, 0.002 mmol, 1 mol%), sodium *p*-methylbenzene sulfinate **3a** (43 mg, 0.24 mmol, 1.2 equiv.), 1,3-enyne **1a** (91 mg, 0.4 mmol, 2 equiv.) and **Ni-A** (93.6 mg, 0.2 mmol, 1 equiv.). It was capped with a rubber septum, evacuated and backfilled with argon. Then, degassed DMSO (2.0 mL) was added via syringe. The reaction mixture was stirred at room temperature under 30 W blue LEDs irradiation for 20 hours. Then the reaction mixture was diluted with EtOAc, then washed with brine, dried over Na_2SO_4, filtered, and concentrated under reduced pressure. Product **5c** was not detected by crude ^{1}H NMR analysis.

Catalytic reaction with Ni-A

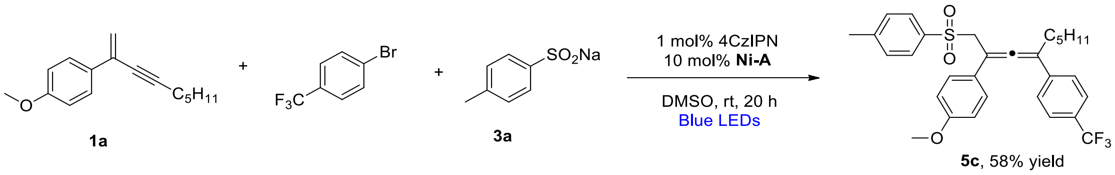

A dry reaction tube equipped with a Teflon-coated magnetic stir bar was charged with 4CzIPN (1.6 mg, 0.002 mmol, 1 mol%), sodium *p*-methylbenzene sulfinate **3a** (43 mg, 0.24 mmol, 1.2 equiv.), 1,3-enyne **1a** (91 mg, 0.4 mmol, 2 equiv.) and **Ni-A** (9.4 mg, 0.02 mmol, 10 mol%). It was capped with a rubber septum, evacuated and backfilled with argon. Then, degassed DMSO (2.0 mL) and 4-bromobenzotrifluoride (28 uL, 0.2 mmol, 1.0 equiv.) were added via syringe. The reaction mixture was stirred at room temperature under 30 W blue LEDs irradiation for 20 hours. Then the reaction mixture was diluted with EtOAc, then washed with brine, dried over Na_2SO_4, filtered, and concentrated under reduced pressure. Product **5c** was obtained in 58% yield.

The regioselectivity of 3,4-sulfonylarylation of 1,3-enynes

For our 3,4-sulfonylarylation of 1,3-enynes, migratory insertion of sulfonyl group took place at the C3 atom of 1,3-enynes, and such regioselectivity is different from Rueping's report.[6] We performed a few more experiments as followings. The 3,4-sulfonylarylation of 1,3-enynes, proceeded smoothly under Rueping's conditions, furnishing the same product (**7a**). Furthermore, Rueping's sulfonylarylation of phenylacetylene under our catalytic conditions yielded the same regioisomer (**7x**). It is therefore clear that the nature of substrates determines the different regioselectivities observed in these two studies.

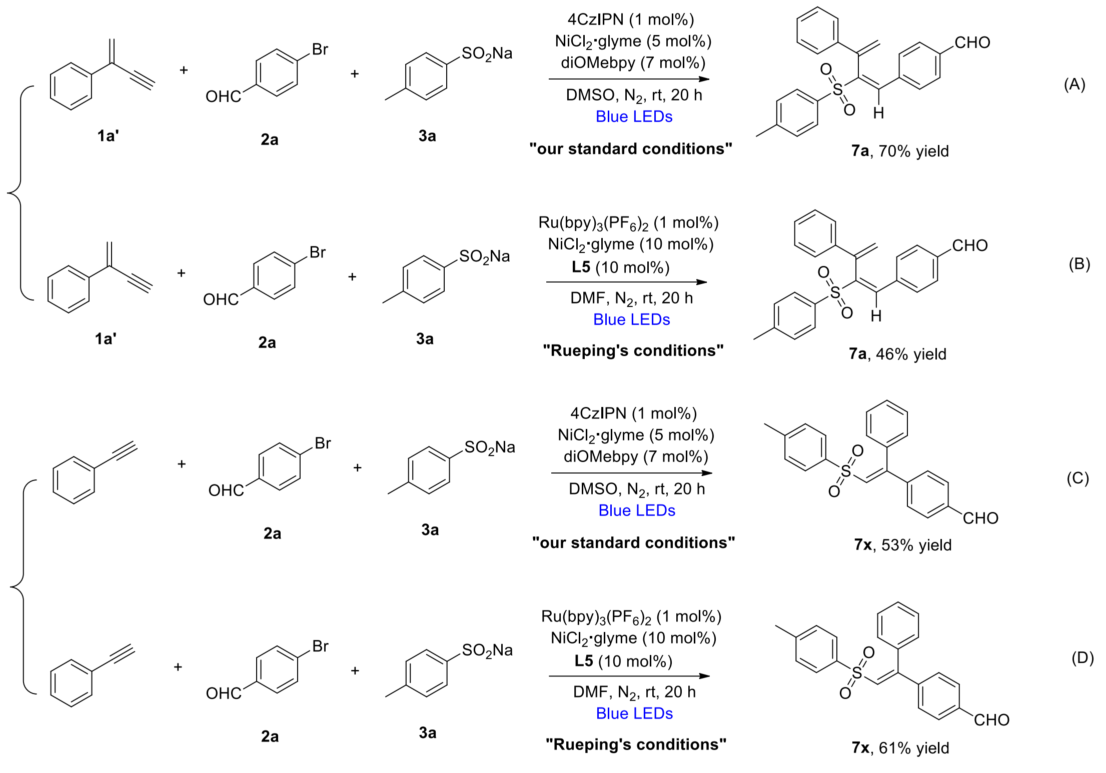

(A) procedure: A dry reaction tube equipped with a Teflon-coated magnetic stir bar was charged with 4CzIPN (1.6 mg, 0.002 mmol, 1 mol%), $NiCl_2$·glyme (2.2 mg, 0.01 mmol, 5 mol%), diOMebpy (3.0 mg, 0.014 mmol, 7 mol%), 4-bromobenzaldehyde **2a** (36.8 mg, 0.2 mmol, 1 equiv.) and sodium *p*-methylbenzene sulfinate **3a** (42.7 mg, 0.24 mmol, 1.2 equiv.). It was capped with a rubber septum, evacuated and backfilled with

argon. Then, degassed anhydrous DMSO (2.0 mL) and 1,3-enyne **1a'** (51.2 mg, 0.4 mmol, 2 equiv.) were added via syringe. The reaction mixture was stirred at room temperature under 30 W blue LEDs irradiation for 20 hours. After the reaction completion, the reaction mixture was diluted with EtOAc, then washed with brine, dried over Na_2SO_4, filtered, and concentrated under reduced pressure. The residue was purified by column chromatography on silica gel (hexane/EtOAc) to afford the corresponding product **7a** (54.3 mg, 70% yield) as light yellow solid.

(B) procedure: A dry reaction tube equipped with a Teflon-coated magnetic stir bar was charged with $Ru(bpy)_3(PF_6)_2$ (1.4 mg, 0.002 mmol, 1 mol%), $NiCl_2$·glyme (4.4 mg, 0.02 mmol, 10 mol%), **L5** (4.7 mg, 0.02 mmol, 10 mol%), 4-bromobenzaldehyde **2a** (73.6 mg, 0.4 mmol, 2 equiv.) and sodium *p*-methylbenzene sulfinate **3a** (35.6 mg, 0.2 mmol, 1 equiv.). It was capped with a rubber septum, evacuated and backfilled with argon. Then, degassed anhydrous DMF (2.0 mL) and 1,3-enyne **1a'** (51.2 mg, 0.4 mmol, 2 equiv.) were added via syringe. The reaction mixture was stirred at room temperature under 30 W blue LEDs irradiation for 20 hours. After the reaction completion, the reaction mixture was diluted with EtOAc, then washed with brine, dried over Na_2SO_4, filtered, and concentrated under reduced pressure. The residue was purified by column chromatography on silica gel (hexane/EtOAc) to afford the corresponding product **7a** (35.7 mg, 46% yield) as light yellow solid.

(C) procedure: A dry reaction tube equipped with a Teflon-coated magnetic stir bar was charged with 4CzIPN (1.6 mg, 0.002 mmol, 1 mol%), $NiCl_2$·glyme (2.2 mg, 0.01 mmol, 5 mol%), diOMebpy (3.0 mg, 0.014 mmol, 7 mol%), 4-bromobenzaldehyde **2a** (36.8 mg, 0.2 mmol, 1 equiv.) and sodium *p*-methylbenzene sulfinate **3a** (42.7 mg, 0.24 mmol, 1.2 equiv.). It was capped with a rubber septum, evacuated and backfilled with argon. Then, degassed anhydrous DMSO (2.0 mL) and phenylacetylene (44 uL, 0.4 mmol, 2 equiv.) were added via syringe. The reaction mixture was stirred at room temperature under 30 W blue LEDs irradiation for 20 hours. After the reaction completion, the reaction mixture was diluted with EtOAc, then washed with brine, dried

over Na_2SO_4, filtered, and concentrated under reduced pressure. The residue was purified by column chromatography on silica gel (hexane/EtOAc) to afford the corresponding product **7x** (38.4 mg, 53% yield) as light yellow solid. ^1H NMR (400 MHz, $CDCl_3$) δ 10.00 (s, 1H), 7.81 (d, J = 8.4 Hz, 2H), 7.49 (d, J = 8.4 Hz, 2H), 7.41 – 7.31 (m, 5H), 7.17 (d, J = 7.6 Hz, 2H), 7.11 (d, J = 7.2 Hz, 2H), 7.05 (s, 1H), 2.39 (s, 3H). Data in accordance with the literature.[6]

(D) procedure: A dry reaction tube equipped with a Teflon-coated magnetic stir bar was charged with $Ru(bpy)_3(PF_6)_2$ (1.4 mg, 0.002 mmol, 1 mol%), $NiCl_2$·glyme (4.4 mg, 0.02 mmol, 10 mol%), **L5** (4.7 mg, 0.02 mmol, 10 mol%), 4-bromobenzaldehyde **2a** (73.6 mg, 0.4 mmol, 2 equiv.) and sodium *p*-methylbenzene sulfinate **3a** (35.6 mg, 0.2 mmol, 1 equiv.). It was capped with a rubber septum, evacuated and backfilled with argon. Then, degassed anhydrous DMF (2.0 mL) and phenylacetylene (44 uL, 0.4 mmol, 2 equiv.) were added via syringe. The reaction mixture was stirred at room temperature under 30 W blue LEDs irradiation for 20 hours. After the reaction completion, the reaction mixture was diluted with EtOAc, then washed with brine, dried over Na_2SO_4, filtered, and concentrated under reduced pressure. The residue was purified by column chromatography on silica gel (hexane/EtOAc) to afford the corresponding product **7x** (44.2 mg, 61% yield) as light yellow solid.

8. X-ray crystallography data

CCDC 2052659 (**4u**) contains the supplementary crystallographic data for this paper. These data can be obtained free of charge from The Cambridge Crystallographic Date Centre via www.ccdc.cam.ac.uk/data_request/cif.

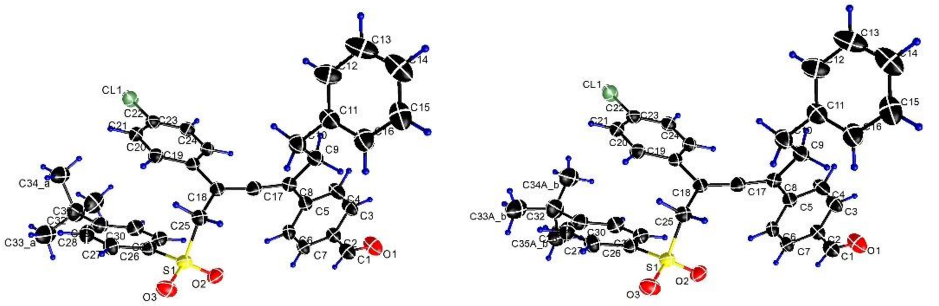

Note: The crystal is Monoclinic, space group P 2(1)/c. The asymmetric unit contains one molecule of the compound C35H33ClO3S. The C(CH3)3 group was disordered into two positions with occupancy ratio = 51:49. Restraints in bond lengths and thermal parameters were applied to the disordered atoms.

Final R values are R1=0.0478 and wR2=0.1279 for 2- theta up to 140º.

Table 1. Crystal data and structure refinement for K482.

Identification code	K482	
Empirical formula	C35 H33 Cl O3 S	
Formula weight	569.12	
Temperature	100(2) K	
Wavelength	1.54178 Å	
Crystal system	Monoclinic	
Space group	$P2_1/c$	
Unit cell dimensions	a = 11.6577(3) Å	a= 90°.
	b = 27.6265(8) Å	b= 113.3930(10)°.
	c = 10.2389(3) Å	g = 90°.
Volume	3026.51(15) Å^3	
Z	4	
Density (calculated)	1.249 Mg/m^3	
Absorption coefficient	2.021 mm^{-1}	
F(000)	1200	
Crystal size	0.627 x 0.323 x 0.266 mm^3	
Theta range for data collection	3.199 to 69.966°.	
Index ranges	-14<=h<=13, -33<=k<=31, -12<=l<=12	
Reflections collected	22521	

Independent reflections	5717 [R(int) = 0.0358]
Completeness to theta = 67.679°	99.8 %
Absorption correction	Semi-empirical from equivalents
Max. and min. transmission	0.7533 and 0.5957
Refinement method	Full-matrix least-squares on F^2
Data / restraints / parameters	5717 / 39 / 395
Goodness-of-fit on F^2	1.067
Final R indices [I>2sigma(I)]	R1 = 0.0478, wR2 = 0.1256
R indices (all data)	R1 = 0.0508, wR2 = 0.1279
Extinction coefficient	n/a
Largest diff. peak and hole	0.701 and -0.377 e.Å^{-3}

CCDC 2052657 (**7m**) contains the supplementary crystallographic data for this paper. These data can be obtained free of charge from The Cambridge Crystallographic Date Centre via www.ccdc.cam.ac.uk/data_request/cif.

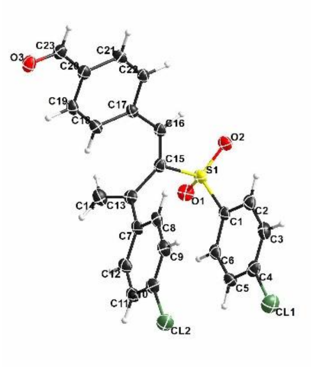

Note: The crystal is triclinic, space group P-1. The asymmetric unit contains one molecule of the compound C23H16Cl2SO3.

The crystal is a non-merohedral twin and twin refinement performed with BASF=0.42289.

Final R values are R1=0.0644 and wR2=0.1611 for 2- theta up to 57°.

Table 1. Sample and crystal data for K433.

Identification code	K433
Chemical formula	$C_{23}H_{16}Cl_2O_3S$

Formula weight	443.32 g/mol	
Temperature	100(2) K	
Wavelength	0.71073 Å	
Crystal size	0.028 x 0.058 x 0.220 mm	
Crystal system	triclinic	
Space group	P -1	
Unit cell dimensions	a = 7.3445(5) Å	α = 97.717(2)°
	b = 7.5430(5) Å	β = 95.887(2)°
	c = 18.6349(12) Å	γ = 105.087(2)°
Volume	977.50(11) $Å^3$	
Z	2	
Density (calculated)	1.506 g/cm^3	
Absorption coefficient	0.462 mm^{-1}	
F(000)	456	

9. References

[1] A. U. Meyer, K. Straková, T. Slanina, B. König, *Chem. Eur. J.* **2016**, *22*, 8694.

[2] a) X. Zhu, W. Deng, M-F. Chiou, C. Ye, W. Jian, Y. Zeng, Y. Jiao, L. Ge, Y. Li, X. Zhang, H. Bao, *J. Am. Chem. Soc.* **2019**, *141*, 548; b) T. R. Pradhan, D. K. Mohapatra, *Adv. Synth. Catal.* **2019**, *361*, 3605.

[3] Y. Song, S. Song, X. Duan, X. Wu, F. Jiang, Y. Zhang, J. Fan, X. Huang, C. Fu, S. Ma, *Chem. Commun.* **2019**, *55*, 11774.

[4] L. Huang, C. Zhu, L. Yi, H. Yue, R. Kancherla, M. Rueping, *Angew. Chem. Int. Ed.* **2020**, *59*, 457.

[5] S-Z. Sun, Y. Duan, R. S. Mega, R. J. Somerville, R. Martin, *Angew. Chem. Int. Ed.* **2020**, *59*, 4370.

[6] C. Zhu, H. Yue, B. Maity, I. Atodiresei, L. Cavallo, M. Rueping, *Nat. Catal.*, **2019**, *2*, 678.

10. Copy of NMR spectra

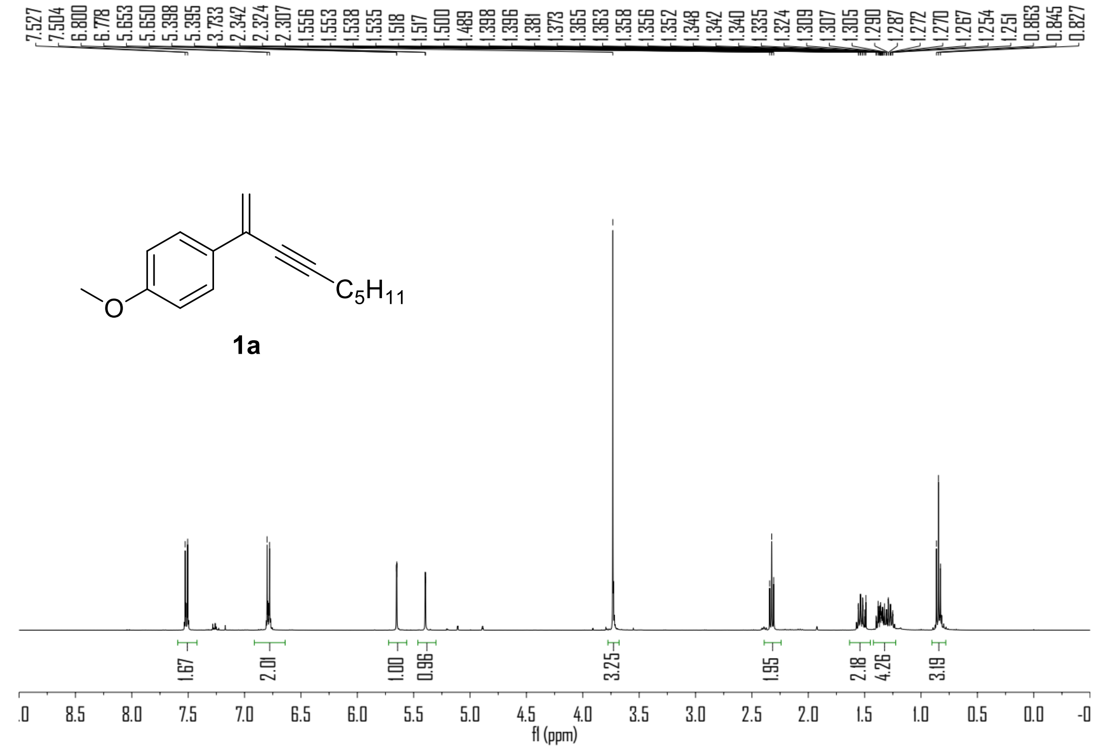

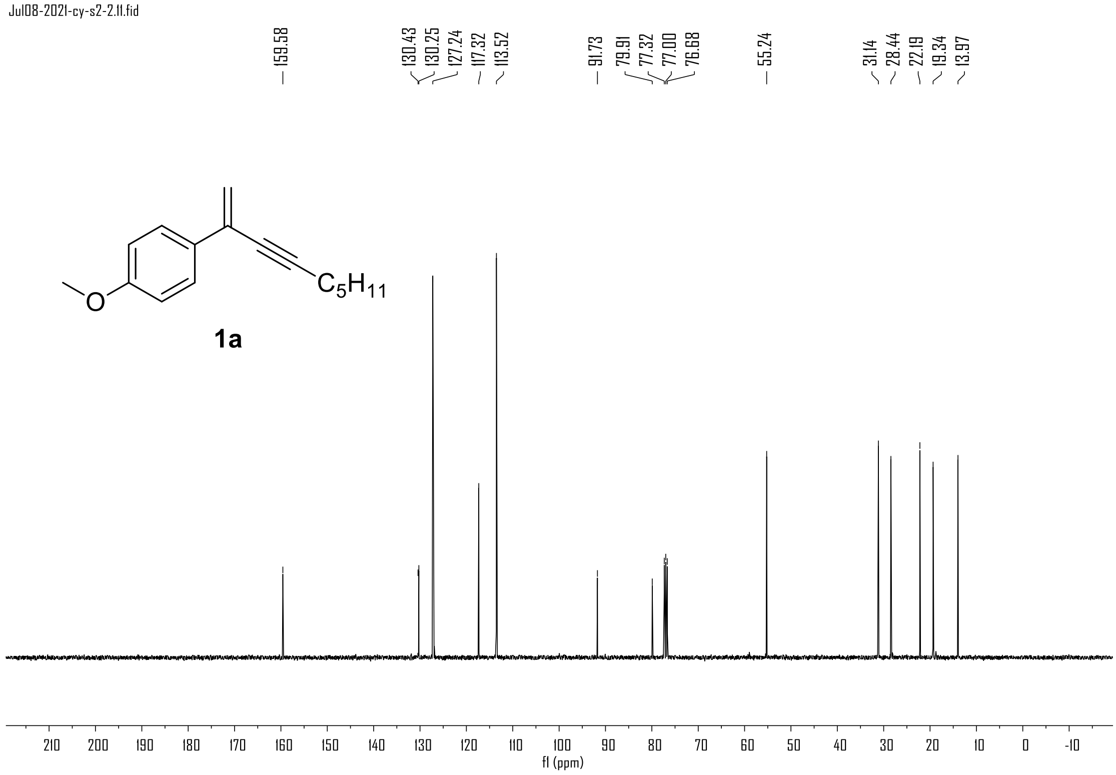

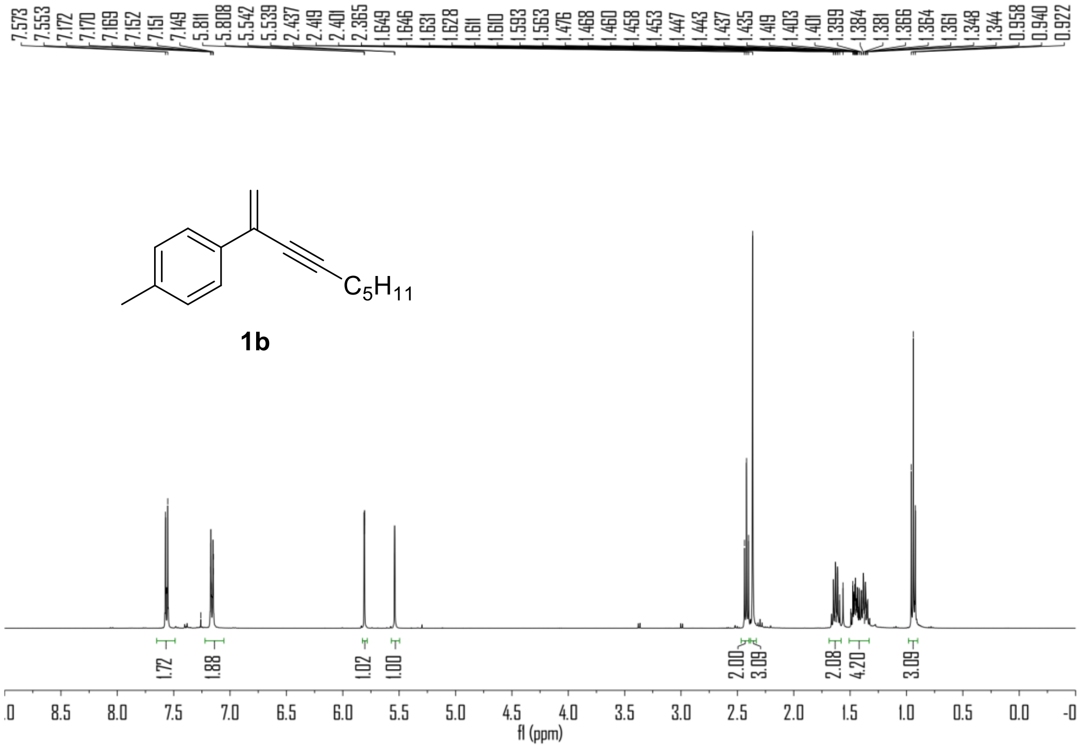
7.573
7.553
7.172
7.170
7.169
7.152
7.151
7.149
5.811
5.808
5.542
5.539
2.437
2.419
2.401
2.365
1.649
1.646
1.631
1.628
1.611
1.610
1.593
1.563
1.476
1.468
1.460
1.458
1.453
1.447
1.443
1.437
1.435
1.419
1.403
1.401
1.399
1.384
1.381
1.366
1.364
1.361
1.348
1.344
0.958
0.940
0.922
C5H11
1b
1.72
1.88
1.02
1.00
2.00
3.09
2.08
4.20
3.09
fl (ppm)

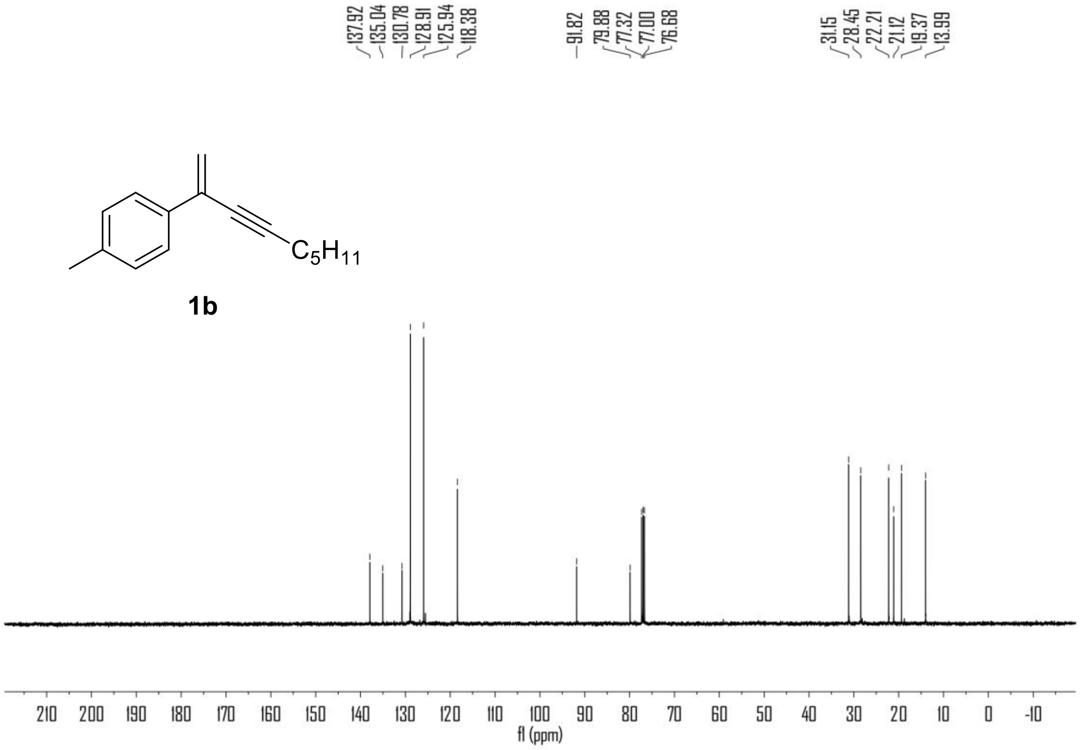
137.92
135.04
130.78
128.91
125.94
118.38
91.82
79.88
77.32
77.00
76.68
31.15
28.45
22.21
21.12
19.37
13.99
C5H11
1b
fl (ppm)

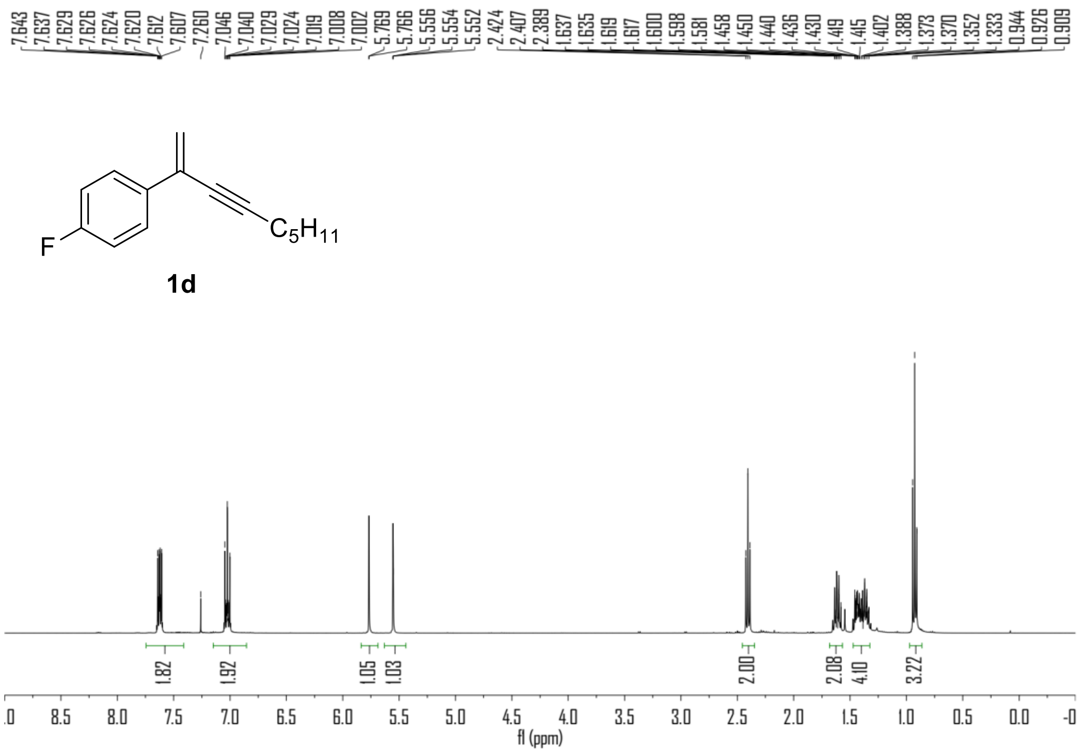

7.643
7.637
7.629
7.626
7.624
7.620
7.612
7.607
7.260
7.046
7.040
7.029
7.024
7.019
7.008
7.002
5.769
5.766
5.556
5.554
5.552
2.424
2.407
2.389
1.637
1.635
1.619
1.617
1.600
1.598
1.581
1.458
1.450
1.440
1.436
1.430
1.419
1.415
1.402
1.388
1.373
1.370
1.352
1.333
0.944
0.926
0.909
F
C5H11
1d
1.82
1.92
1.05
1.03
2.00
2.08
4.10
3.22
fl (ppm)

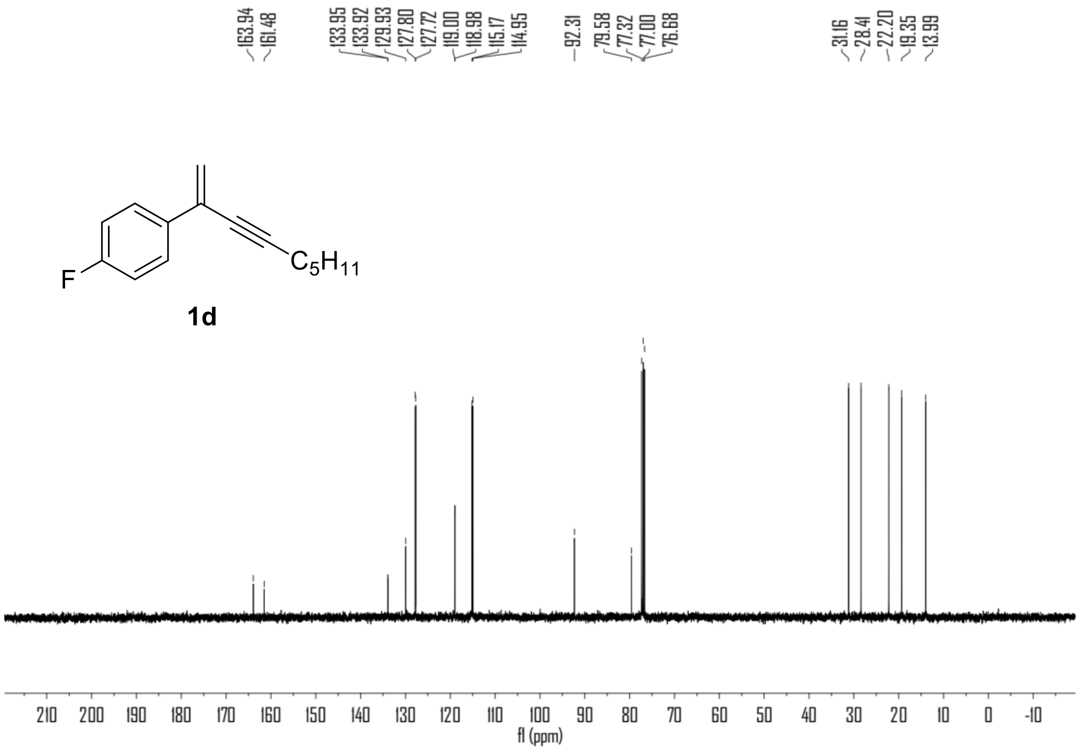

163.94
161.48
133.95
133.92
129.93
127.80
127.72
119.00
118.98
115.17
114.95
92.31
79.58
77.32
77.00
76.68
31.16
28.41
22.20
19.35
13.99
F
C5H11
1d
fl (ppm)

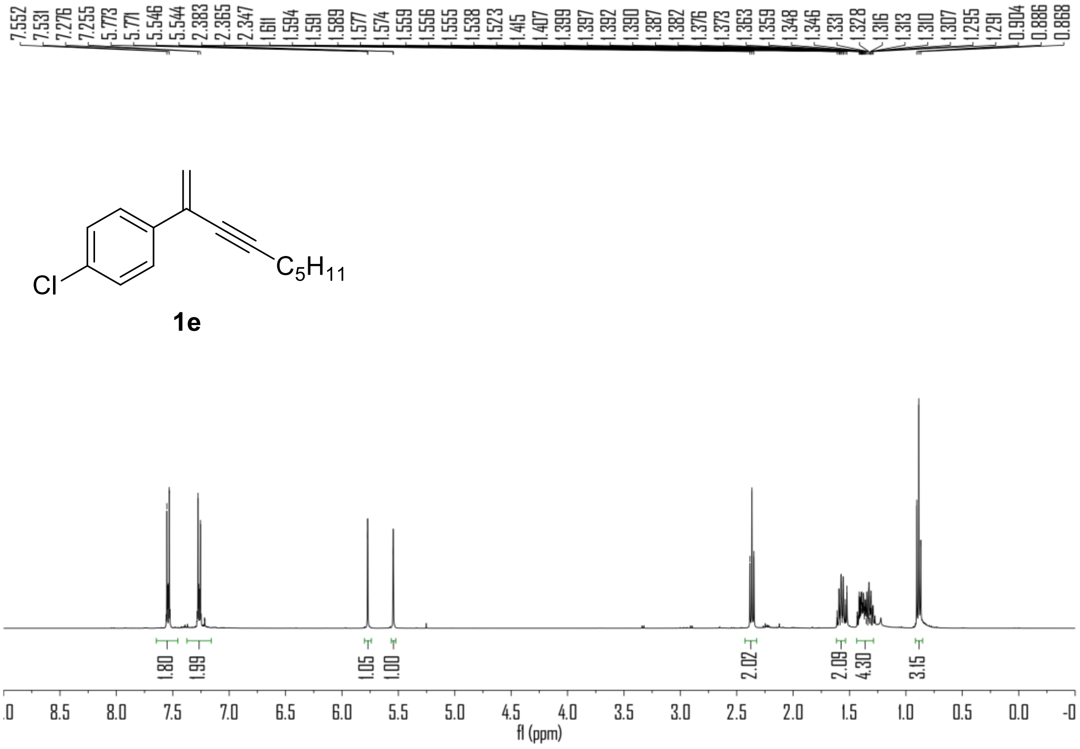
7.552 7.531 7.276 7.255 5.773 5.771 5.546 5.544 2.383 2.365 2.347 1.611 1.594 1.591 1.589 1.577 1.574 1.559 1.556 1.555 1.538 1.523 1.415 1.407 1.399 1.397 1.392 1.390 1.387 1.382 1.376 1.373 1.363 1.359 1.348 1.346 1.331 1.328 1.316 1.313 1.310 1.307 1.295 1.291 0.904 0.886 0.868
Cl
C5H11
1e
1.80 1.99 1.05 1.00 2.02 2.09 4.30 3.15
fl (ppm)

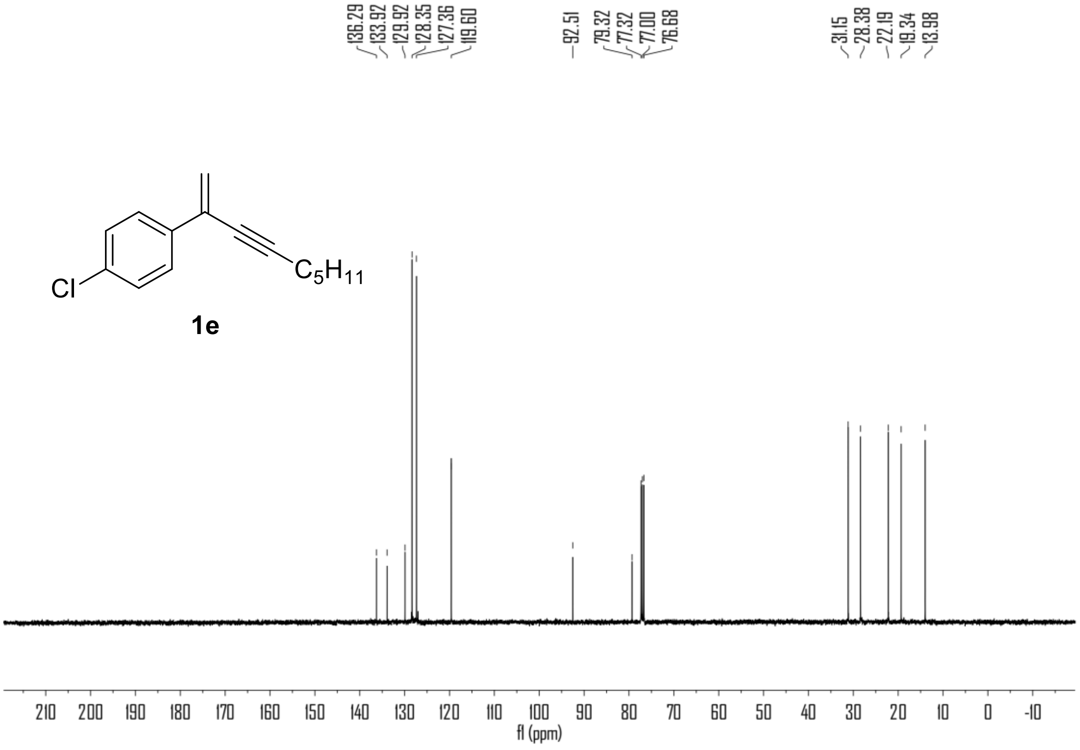
136.29 133.92 129.92 128.35 127.36 119.60 92.51 79.32 77.32 77.00 76.68 31.15 28.38 22.19 19.34 13.98
Cl
C5H11
1e
fl (ppm)

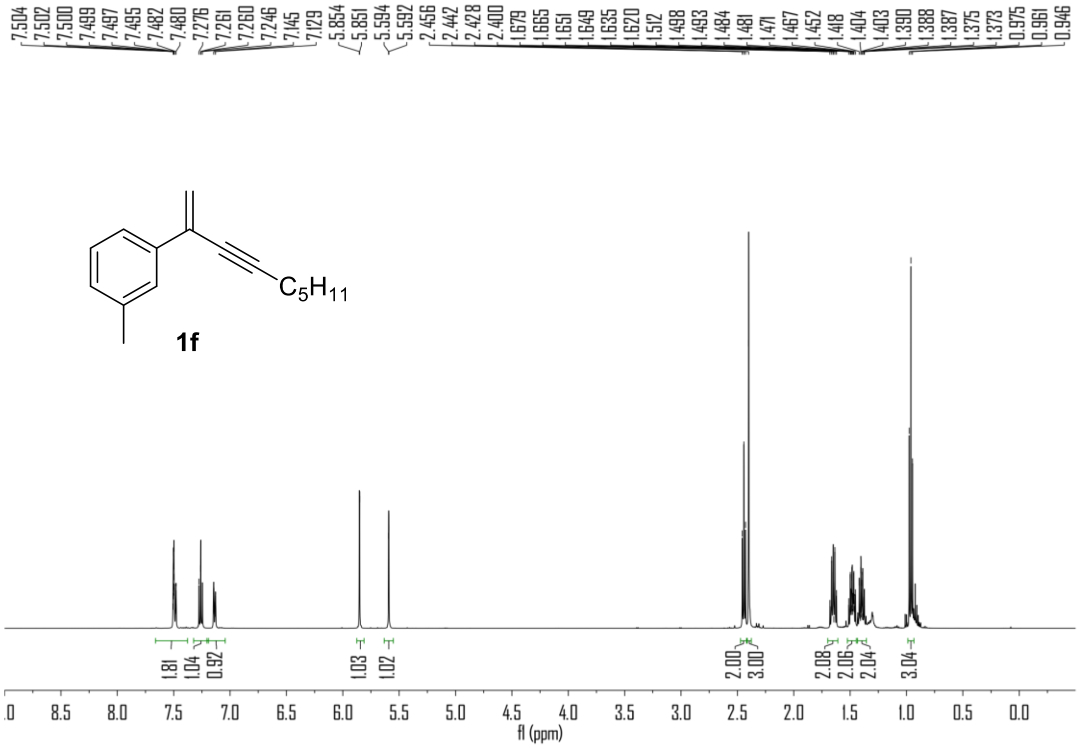
7.504 7.502 7.500 7.499 7.497 7.495 7.482 7.480 7.276 7.261 7.260 7.246 7.145 7.129 5.854 5.851 5.594 5.592 2.456 2.442 2.428 2.400 1.679 1.665 1.651 1.649 1.635 1.620 1.512 1.498 1.493 1.484 1.481 1.471 1.467 1.452 1.418 1.404 1.403 1.390 1.388 1.387 1.375 1.373 0.975 0.961 0.946
C5H11
1f
1.81 1.04 0.92 1.03 1.02 2.00 3.00 2.08 2.06 2.04 3.04
fl (ppm)

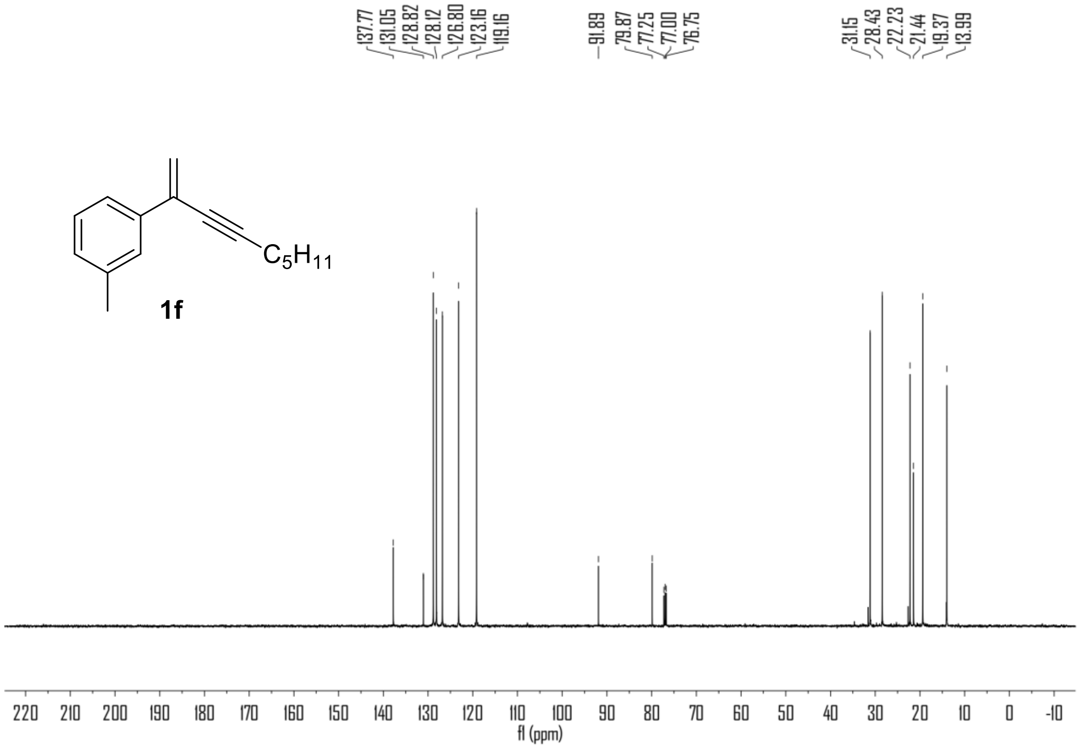
137.77 131.05 128.82 128.12 126.80 123.16 119.16 91.89 79.87 77.25 77.00 76.75 31.15 28.43 22.23 21.44 19.37 13.99
C5H11
1f
fl (ppm)

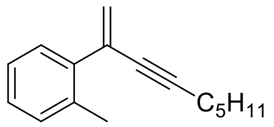

1g

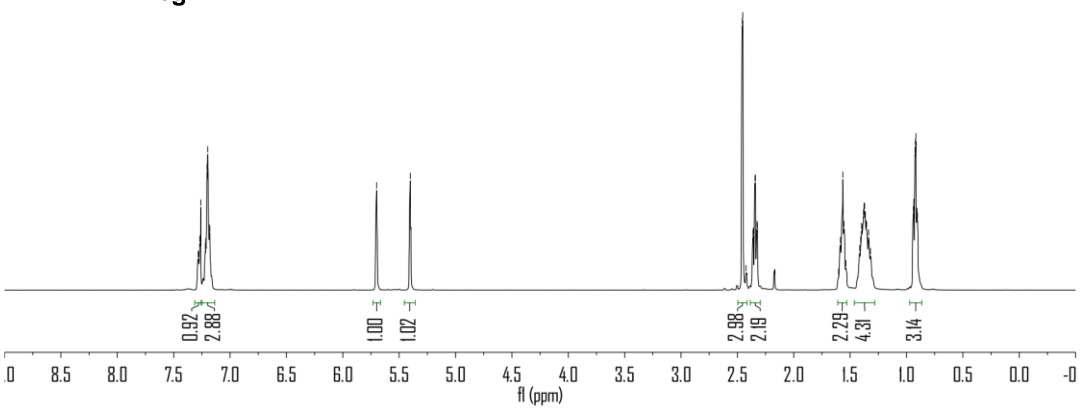
7.283 7.278 7.268 7.263 7.260 7.219 7.215 7.204 7.199 7.196 7.182 7.180 5.700 5.402 2.456 2.452 2.361 2.358 2.343 2.340 2.326 2.322 1.586 1.564 1.555 1.537 1.415 1.406 1.398 1.393 1.381 1.376 1.371 1.365 1.360 1.357 1.354 1.336 1.320 0.939 0.935 0.924 0.921 0.917 0.910 0.906 0.903
0.92 2.88 1.00 1.02 2.98 2.19 2.29 4.31 3.14
fl (ppm)

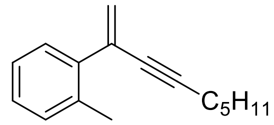

1g

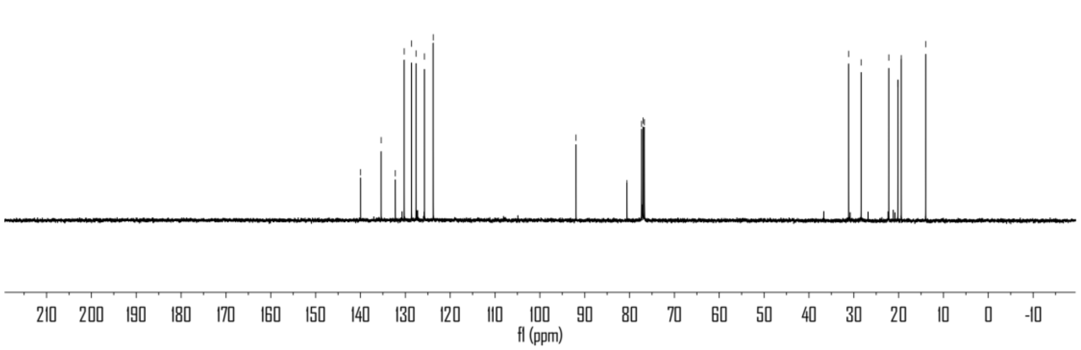
139.97 135.39 132.22 130.26 128.63 127.62 125.75 123.77 91.94 80.59 77.32 77.00 76.68 31.14 28.34 22.18 20.16 19.41 13.95
fl (ppm)

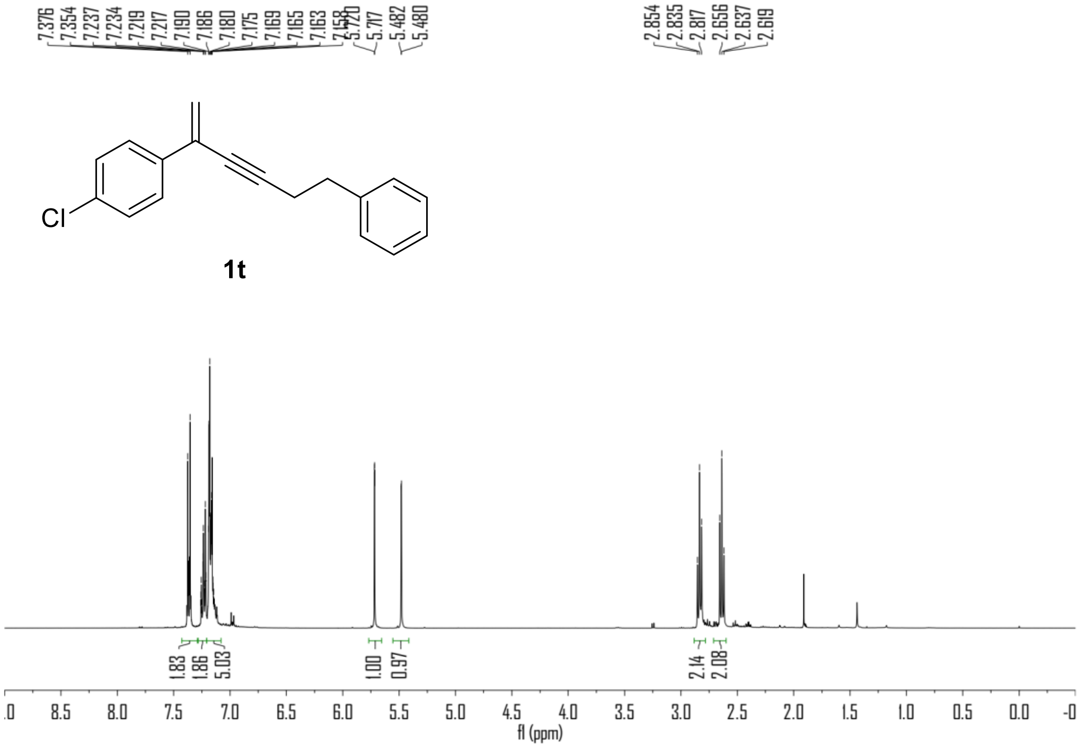
7.376
7.354
7.237
7.234
7.219
7.217
7.190
7.186
7.180
7.175
7.169
7.165
7.163
7.158
5.720
5.717
5.482
5.480
2.854
2.835
2.817
2.656
2.637
2.619
Cl
1t
1.83
1.86
5.03
1.00
0.97
2.14
2.08
fl (ppm)

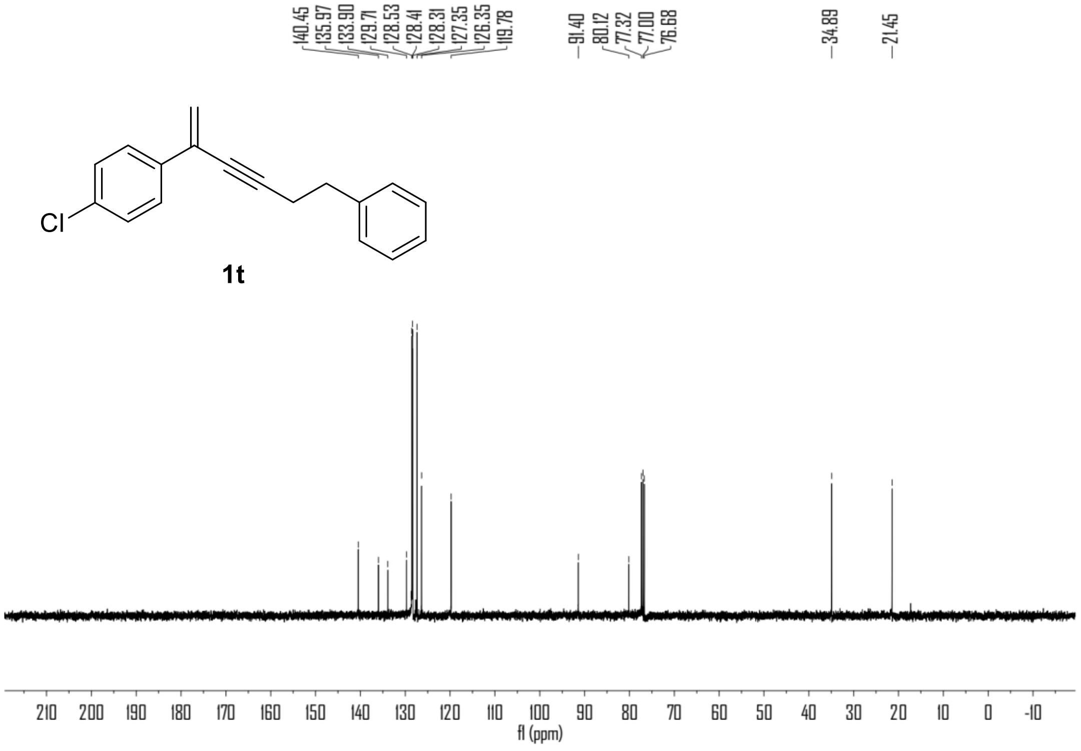
140.45
135.97
133.90
129.71
128.53
128.41
128.31
127.35
126.35
119.78
91.40
80.12
77.32
77.00
76.68
34.89
21.45
Cl
1t
fl (ppm)

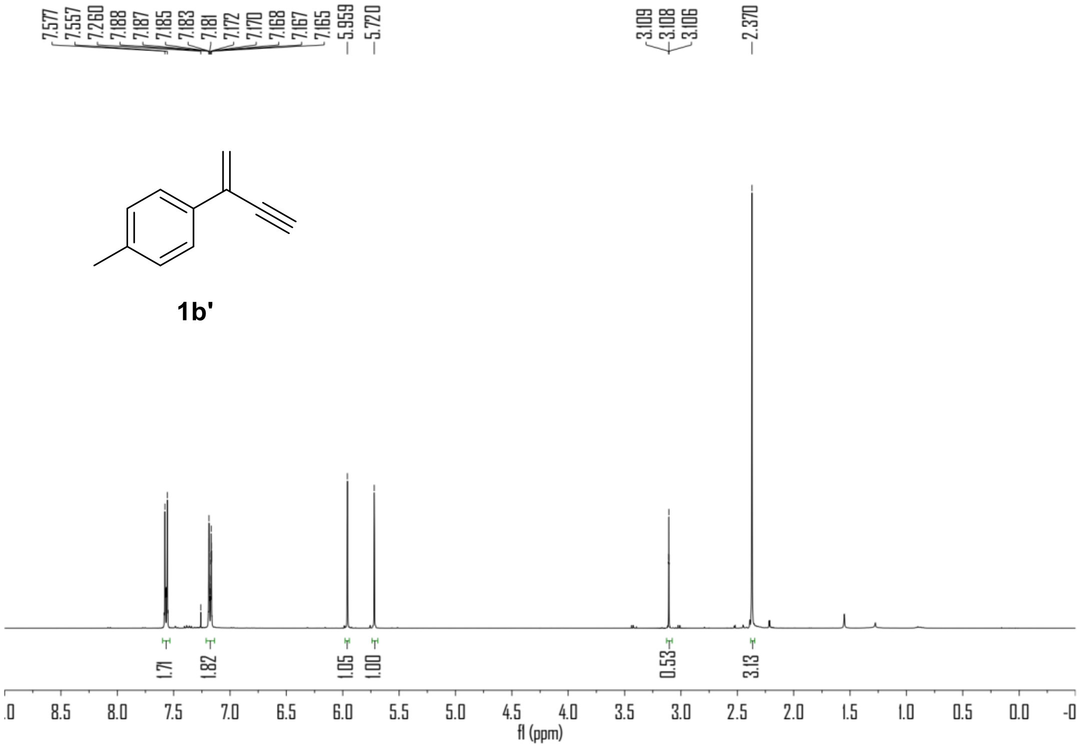
7.577
7.557
7.260
7.188
7.187
7.185
7.183
7.181
7.172
7.170
7.168
7.167
7.165
5.959
5.720
3.109
3.108
3.106
2.370
1b'
1.71
1.82
1.05
1.00
0.53
3.13
fl (ppm)

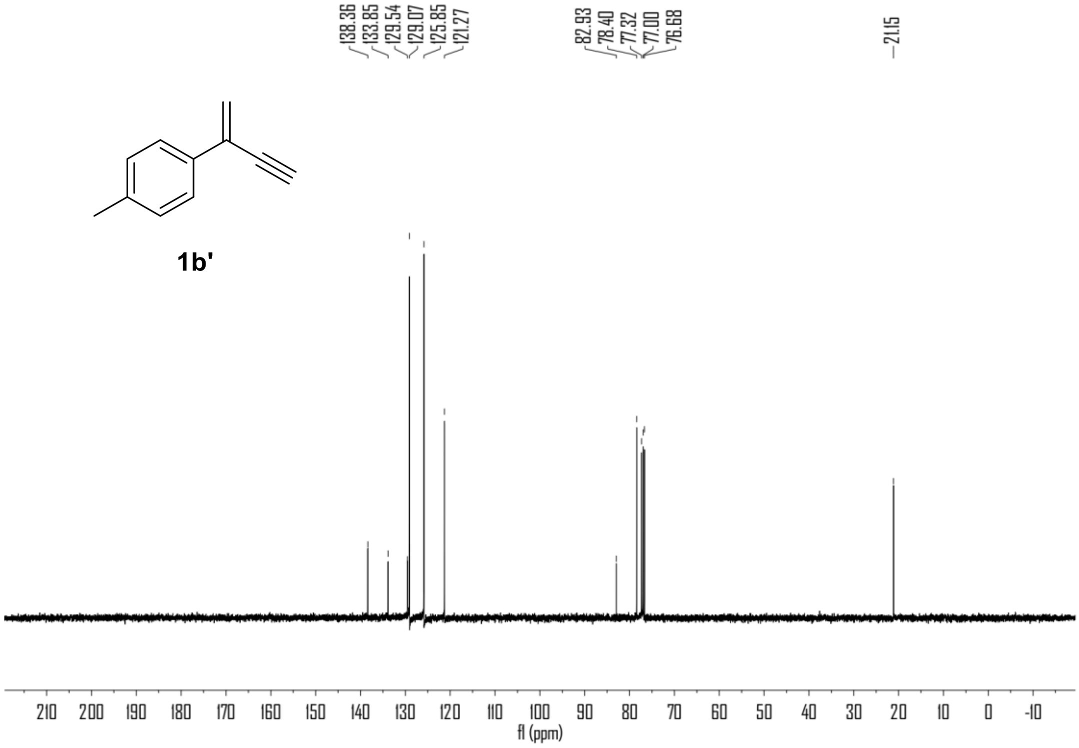
138.36
133.85
129.54
129.07
125.85
121.27
82.93
78.40
77.32
77.00
76.68
21.15
1b'
fl (ppm)

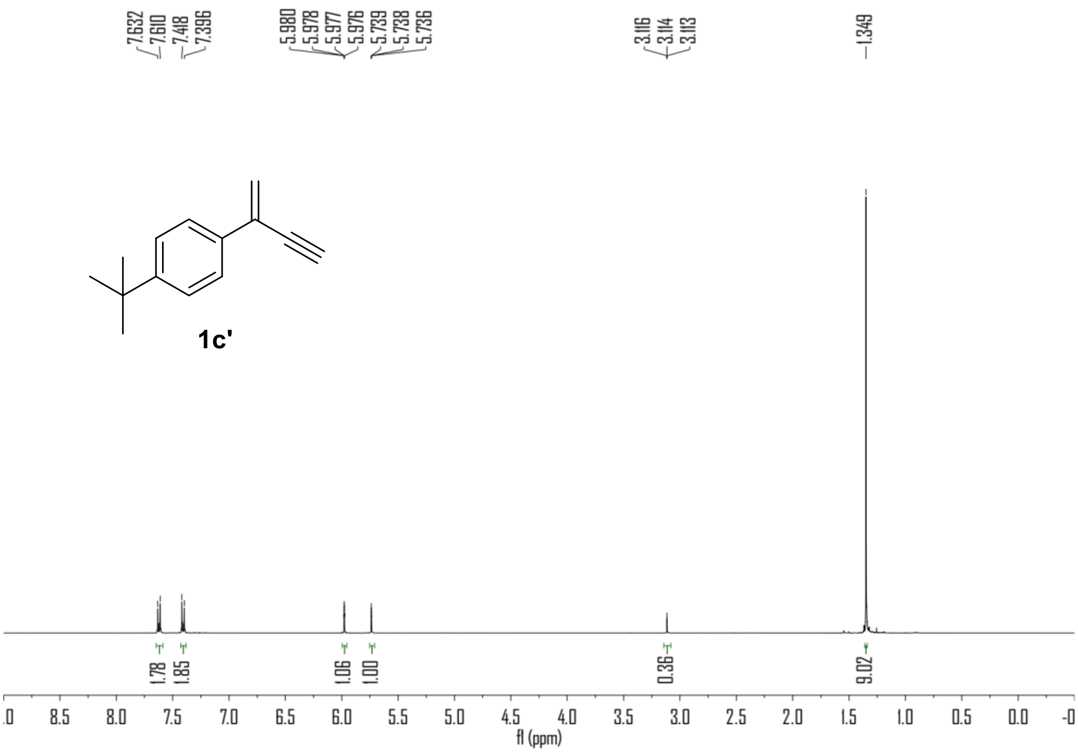
7.632
7.610
7.418
7.396
5.980
5.978
5.977
5.976
5.739
5.738
5.736
3.116
3.114
3.113
1.349
1c'
1.78
1.85
1.06
1.00
0.36
9.02
fl (ppm)

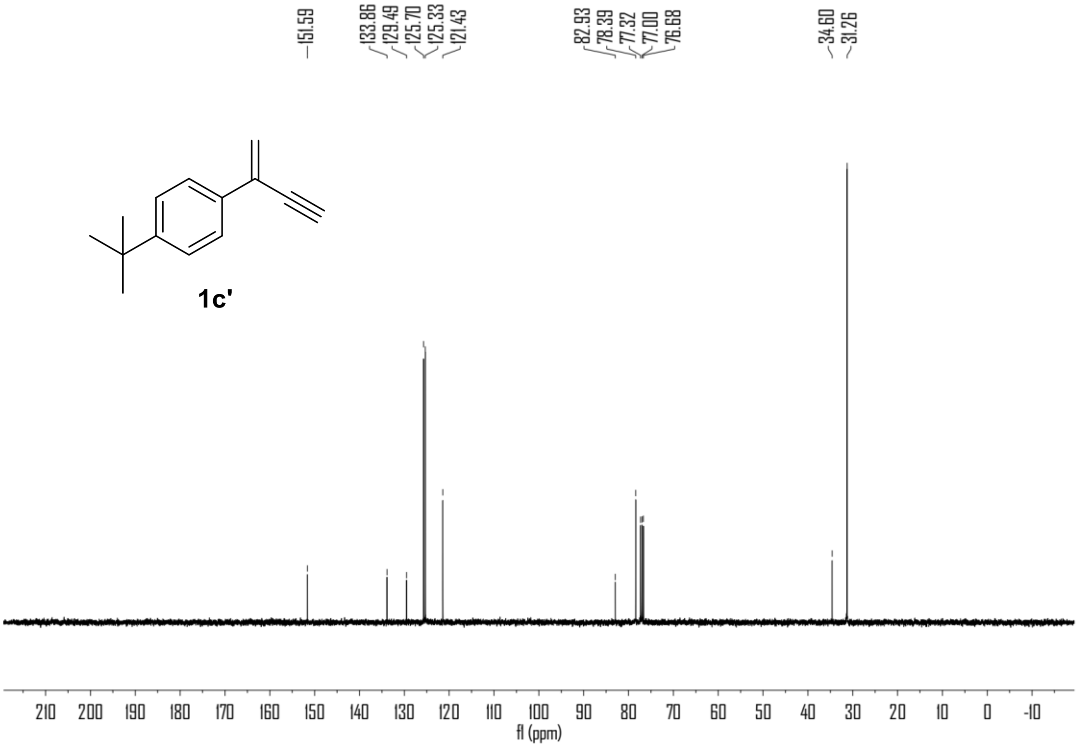
151.59
133.86
129.49
125.70
125.33
121.43
82.93
78.39
77.32
77.00
76.68
34.60
31.26
1c'
fl (ppm)

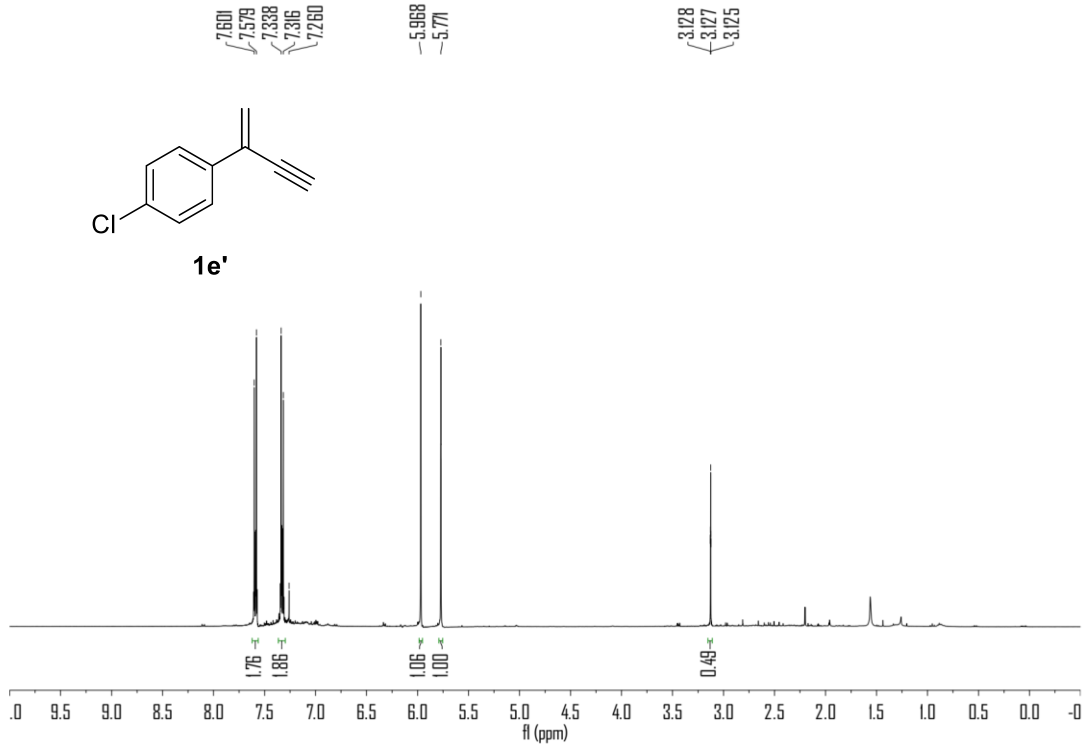
7.601
7.579
7.338
7.316
7.260
5.968
5.771
3.128
3.127
3.125
Cl
1e'
1.76
1.86
1.06
1.00
0.49
fl (ppm)

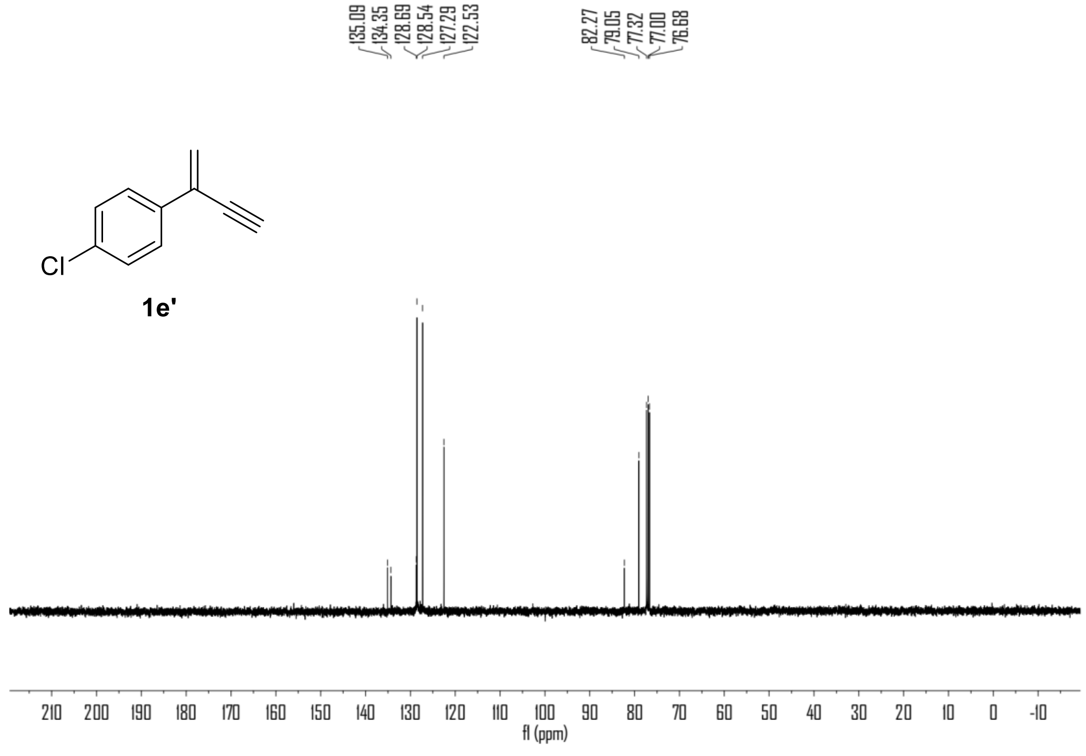
135.09
134.35
128.69
128.54
127.29
122.53
82.27
79.05
77.32
77.00
76.68
Cl
1e'
fl (ppm)

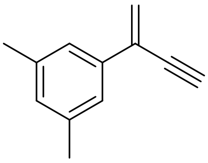

1f'

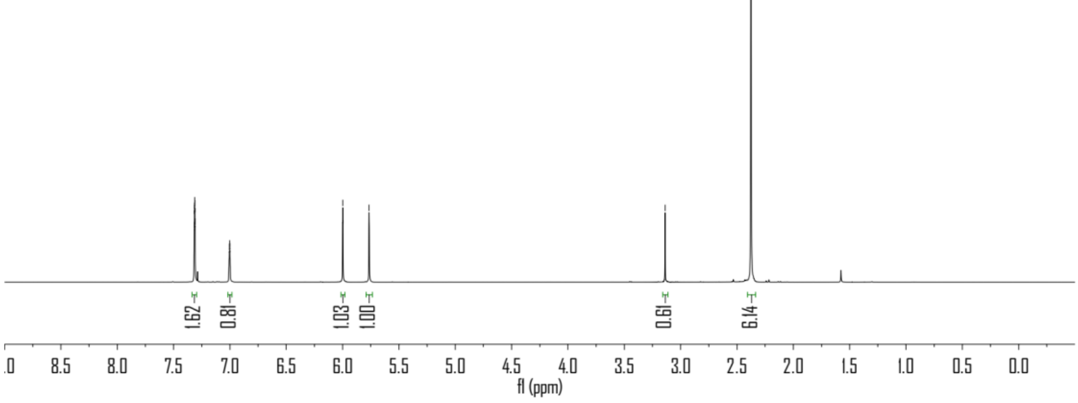
7.316
7.315
7.313
7.311
7.310
7.005
7.003
7.001
5.998
5.765
3.139
3.138
3.137
2.377
2.375
1.62
0.81
1.03
1.00
0.61
6.14
fl (ppm)

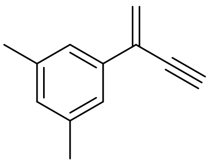

1f'

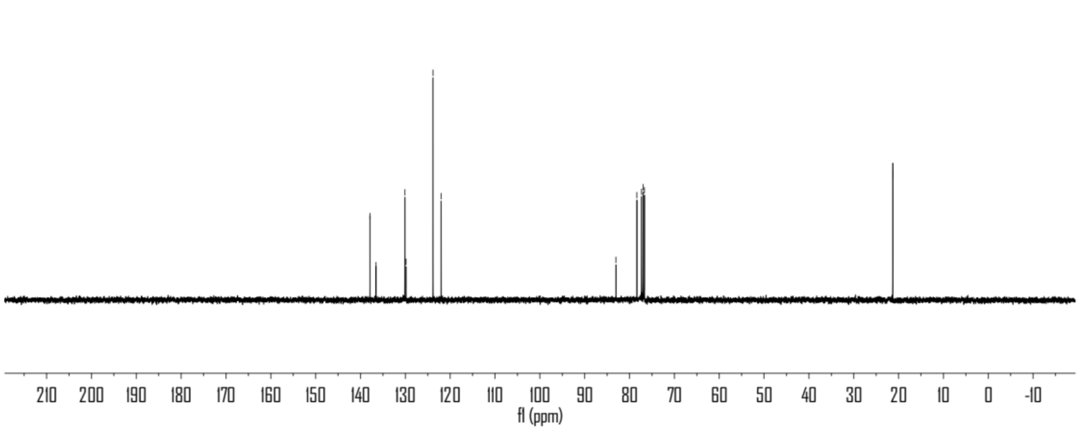
137.90
136.57
130.12
129.85
123.82
121.99
83.03
78.37
77.32
77.00
76.68
21.30
fl (ppm)

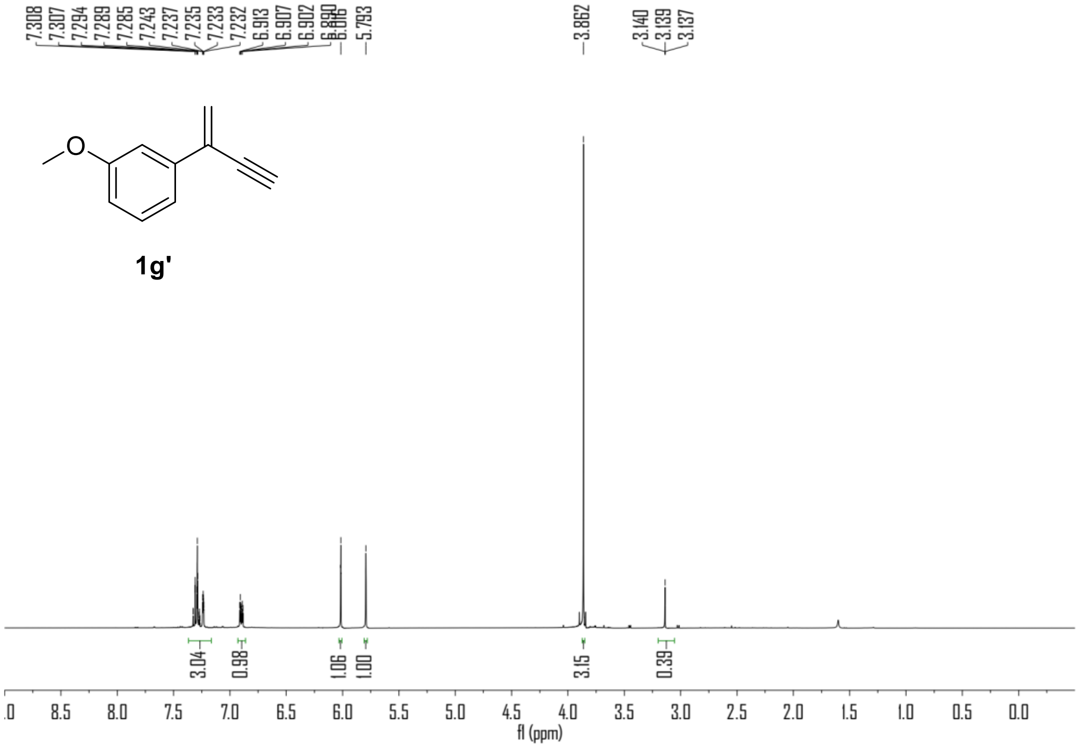
7.308
7.307
7.294
7.289
7.285
7.243
7.237
7.235
7.233
7.232
6.913
6.907
6.902
5.793
3.862
3.140
3.139
3.137
3.04
0.98
1.06
1.00
3.15
0.39
8.5 8.0 7.5 7.0 6.5 6.0 5.5 5.0 4.5 4.0 3.5 3.0 2.5 2.0 1.5 1.0 0.5 0.0
f1 (ppm)

1g'

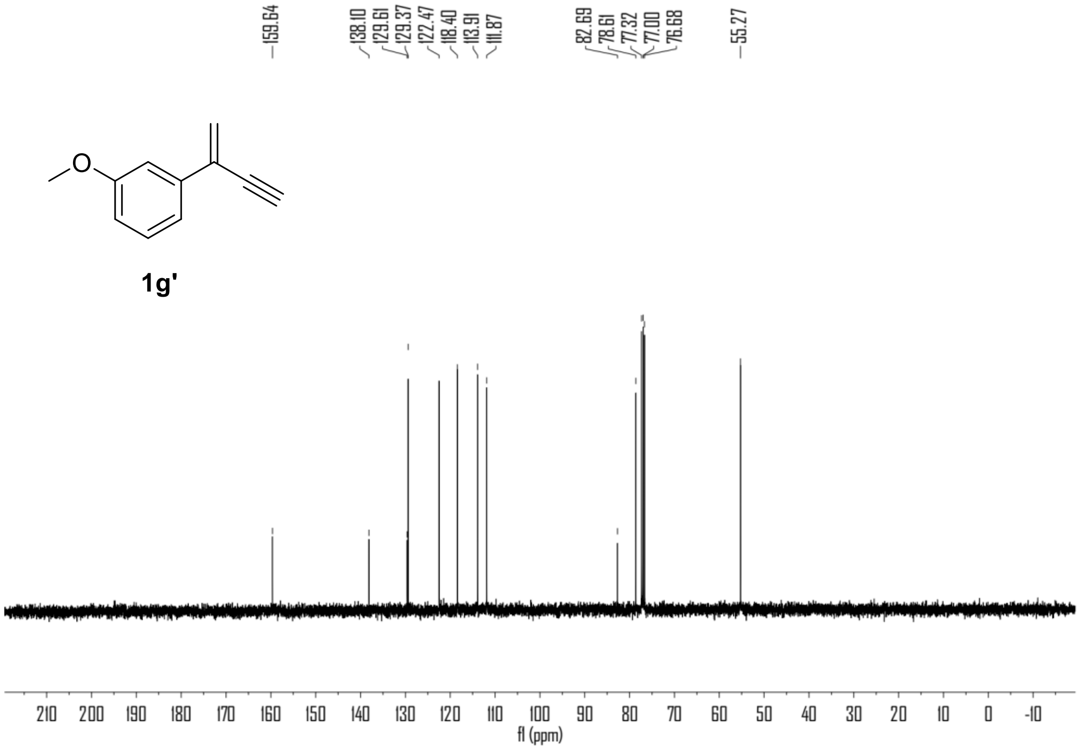
159.64
138.10
129.61
129.37
122.47
118.40
113.91
111.87
82.69
78.61
77.32
77.00
76.68
55.27
210 200 190 180 170 160 150 140 130 120 110 100 90 80 70 60 50 40 30 20 10 0 -10
f1 (ppm)

1g'

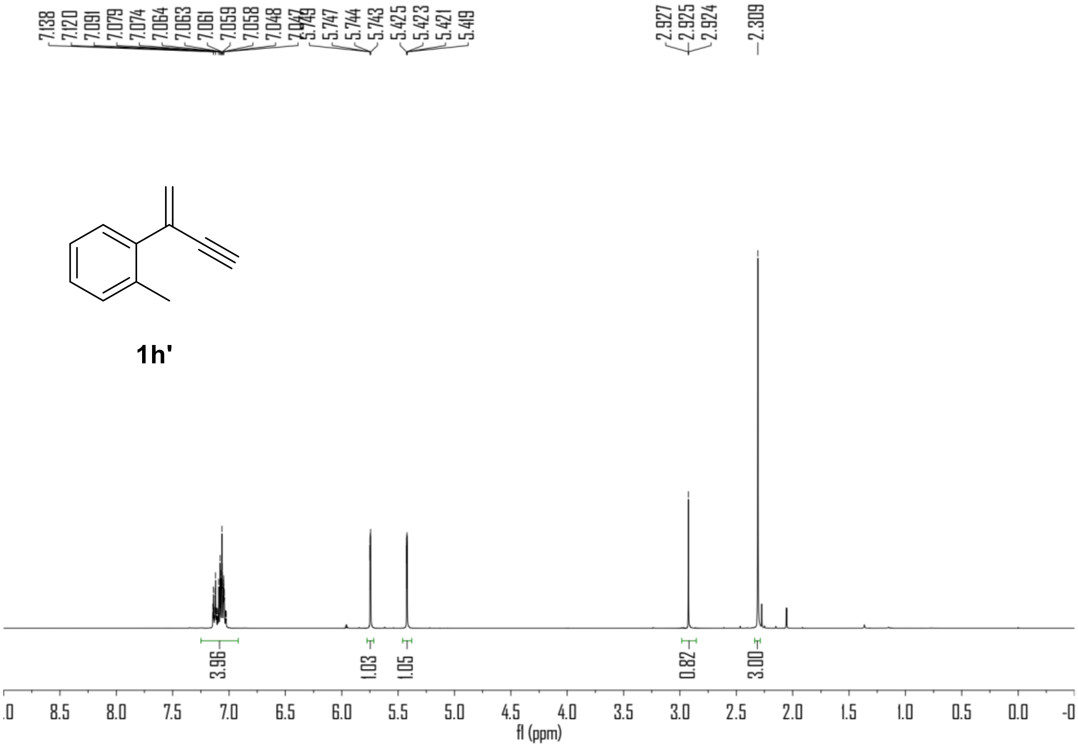

1h'

7.138
7.120
7.091
7.079
7.074
7.064
7.063
7.061
7.059
7.058
7.048
7.047
5.749
5.747
5.744
5.743
5.425
5.423
5.421
5.419
2.927
2.925
2.924
2.309
3.96
1.03
1.05
0.82
3.00
8.5 8.0 7.5 7.0 6.5 6.0 5.5 5.0 4.5 4.0 3.5 3.0 2.5 2.0 1.5 1.0 0.5 0.0
f1 (ppm)

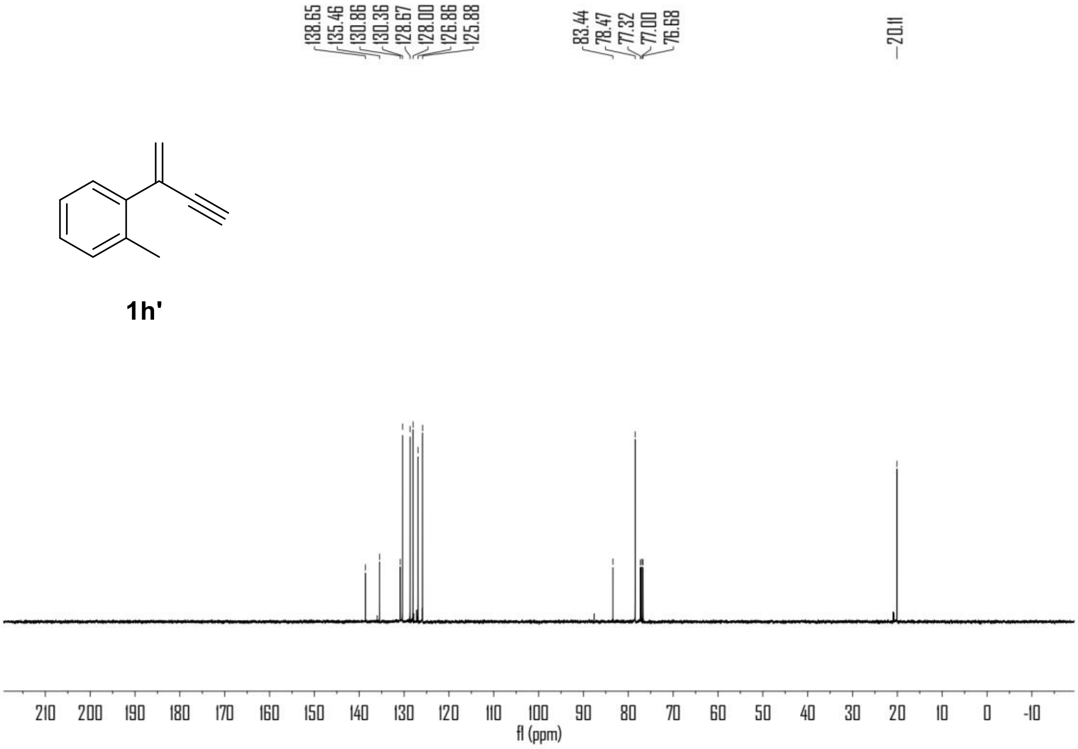

1h'

138.65
135.46
130.86
130.36
128.67
128.00
126.86
125.88
83.44
78.47
77.32
77.00
76.68
20.11
210 200 190 180 170 160 150 140 130 120 110 100 90 80 70 60 50 40 30 20 10 0 -10
f1 (ppm)

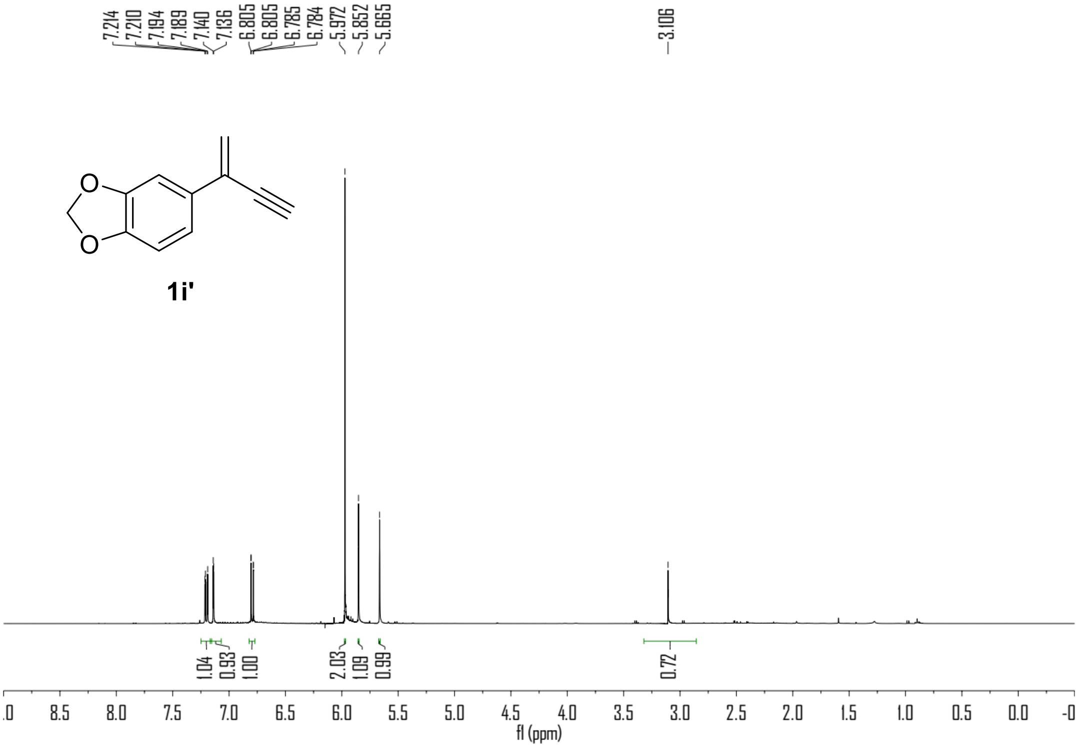
7.214
7.210
7.194
7.189
7.140
7.136
6.805
6.805
6.785
6.784
5.972
5.852
5.665
3.106
1i'
1.04
0.93
1.00
2.03
1.09
0.99
0.72
fl (ppm)

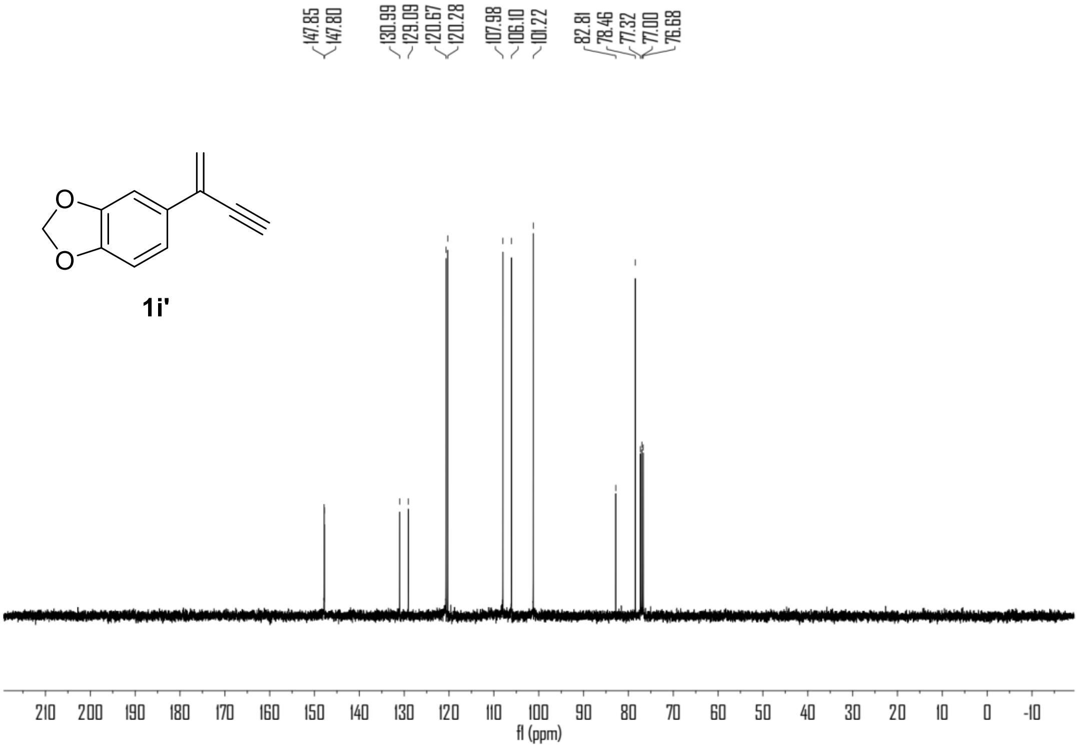
147.85
147.80
130.99
129.09
120.67
120.28
107.98
106.10
101.22
82.81
78.46
77.32
77.00
76.68
1i'
fl (ppm)

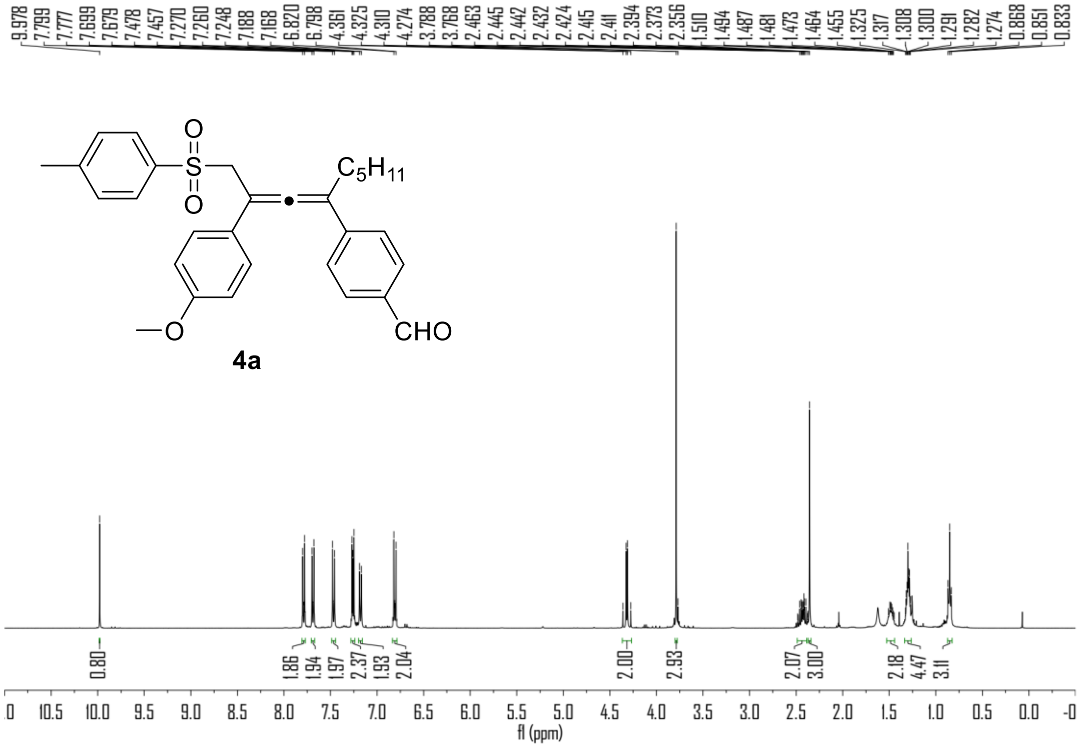

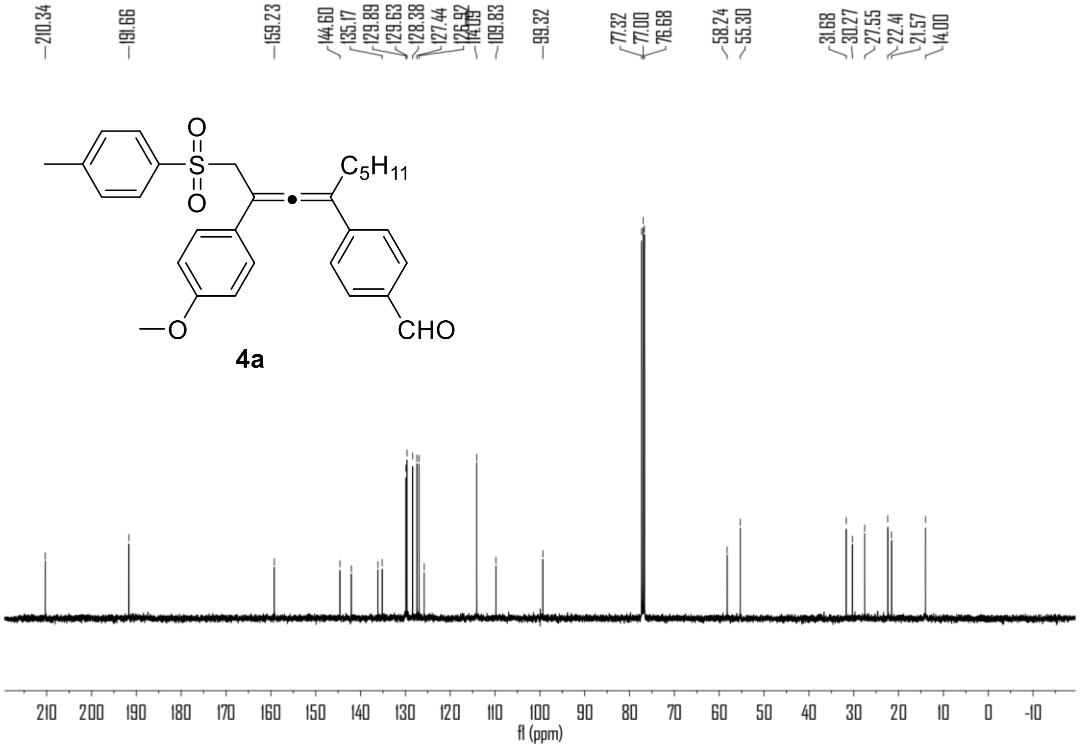

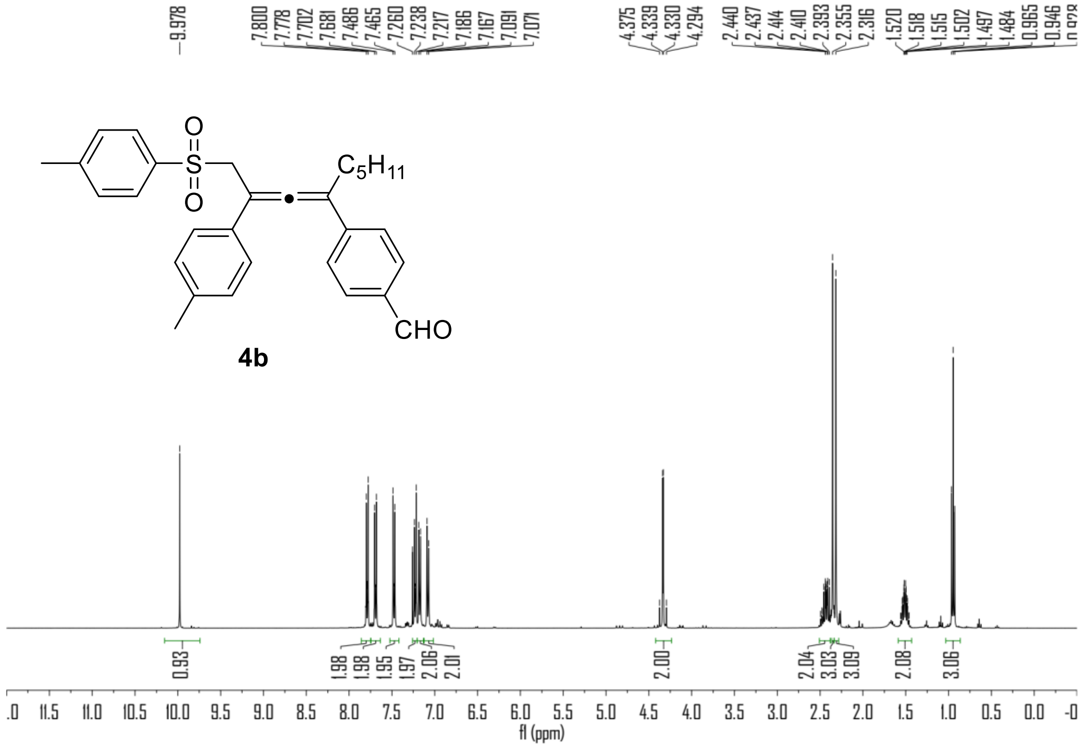

C5H11
CHO
4b
fl (ppm)

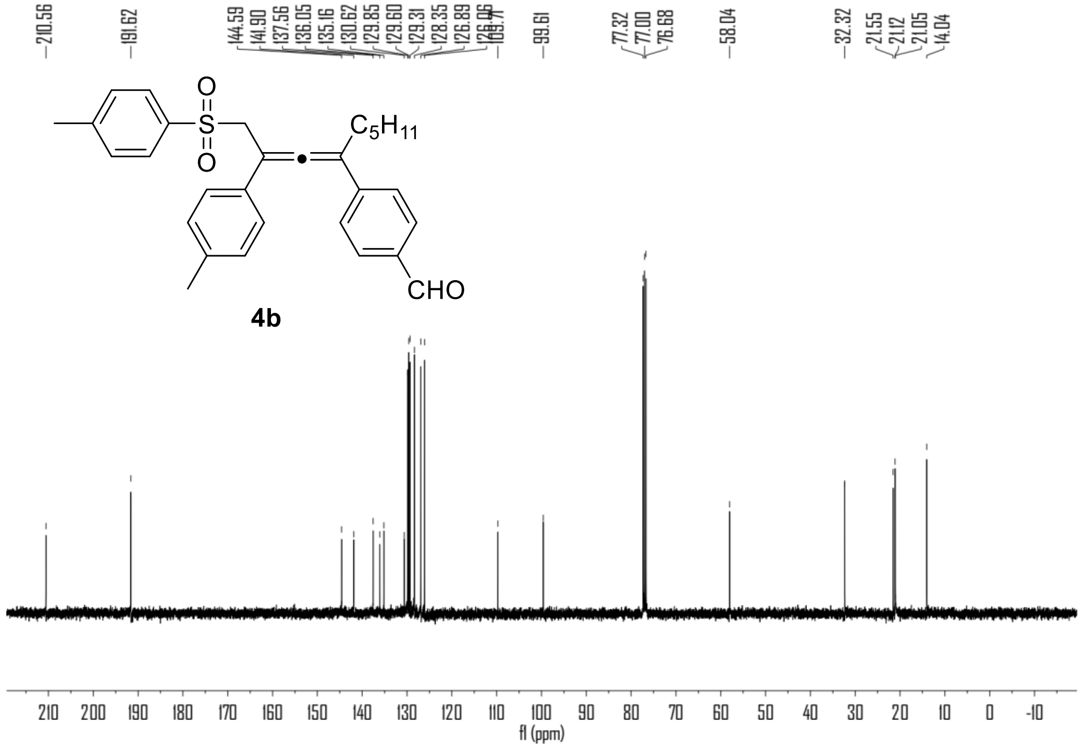

C5H11
CHO
4b
fl (ppm)

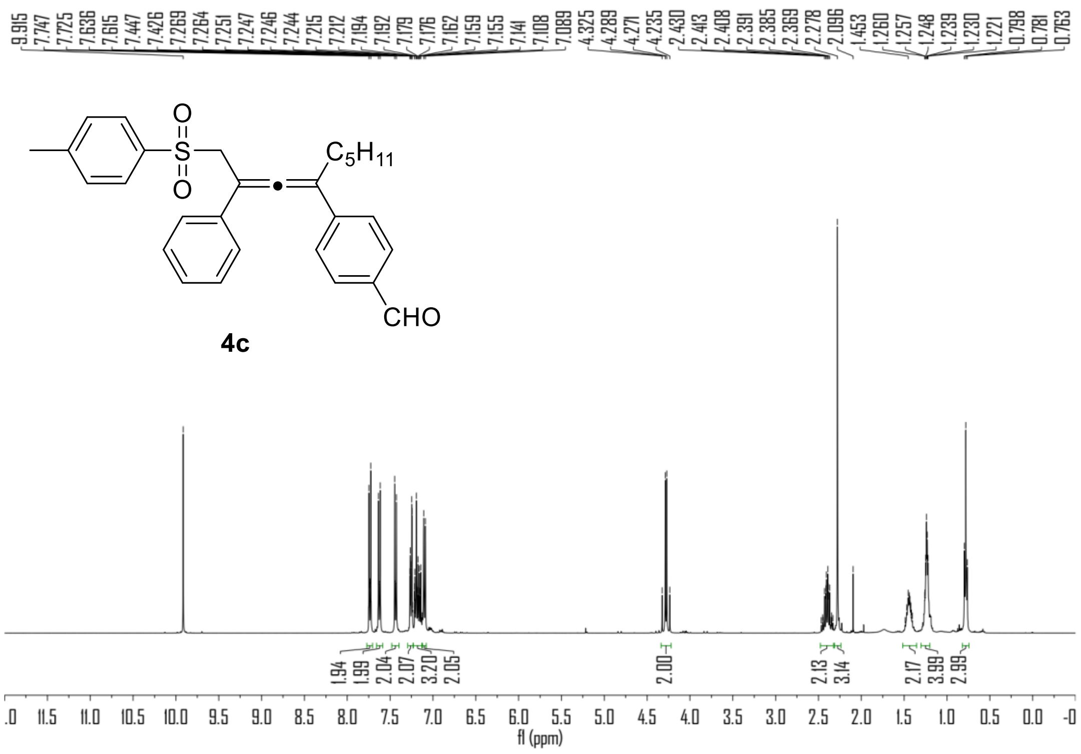

9.915 7.747 7.725 7.636 7.615 7.447 7.426 7.269 7.264 7.251 7.247 7.246 7.244 7.215 7.212 7.194 7.192 7.179 7.176 7.162 7.159 7.155 7.141 7.108 7.089 4.325 4.289 4.271 4.235 2.430 2.413 2.408 2.391 2.385 2.369 2.278 2.096 1.453 1.260 1.257 1.248 1.239 1.230 1.221 0.798 0.781 0.763
O
S
O
C5H11
CHO
4c
1.94 1.99 2.04 2.07 3.20 2.05 2.00 2.13 3.14 2.17 3.99 2.99
fl (ppm)

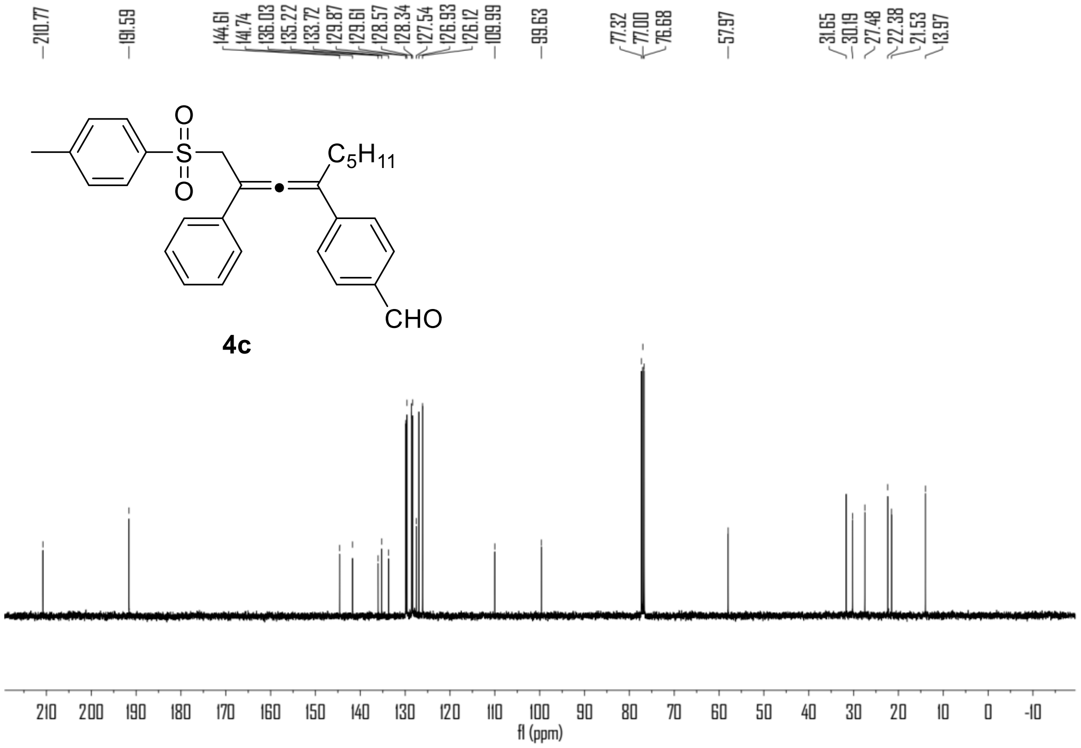

210.77 191.59 144.61 141.74 136.03 135.22 133.72 129.87 129.61 128.57 128.34 127.54 126.93 126.12 109.99 99.63 77.32 77.00 76.68 57.97 31.65 30.19 27.48 22.38 21.53 13.97
O
S
O
C5H11
CHO
4c
fl (ppm)

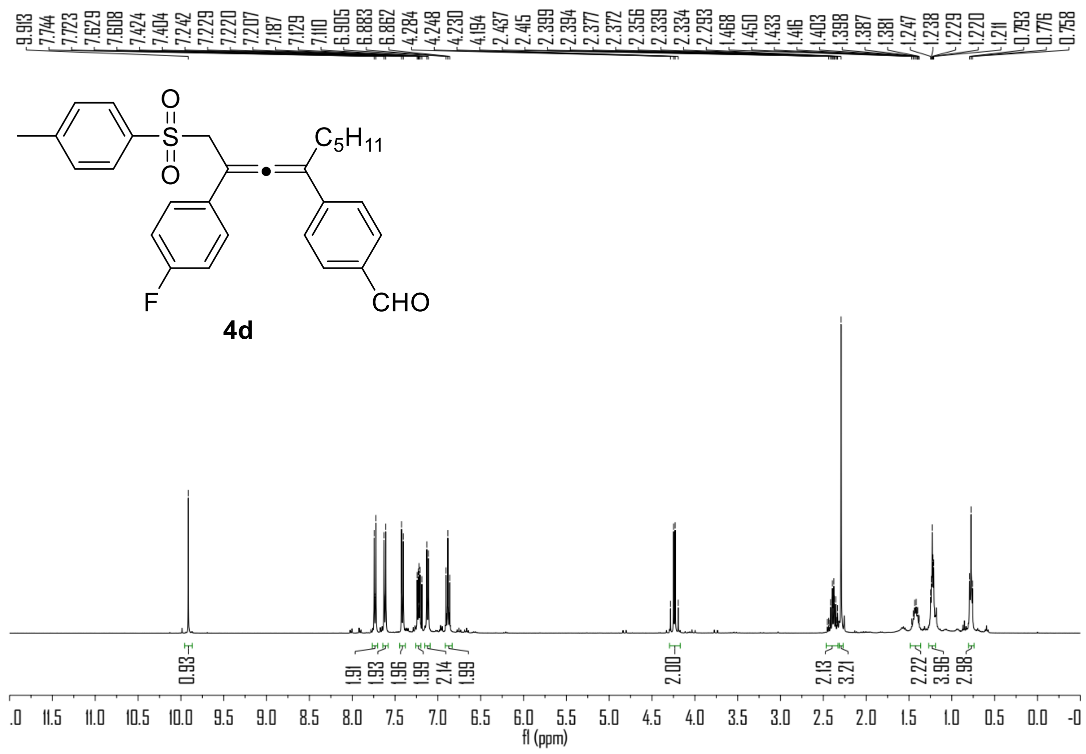
9.913 7.744 7.723 7.629 7.608 7.424 7.404 7.242 7.229 7.220 7.207 7.187 7.129 7.110 6.905 6.883 6.862 4.284 4.248 4.230 4.194 2.437 2.415 2.399 2.394 2.377 2.372 2.356 2.339 2.334 2.293 1.468 1.450 1.433 1.416 1.403 1.398 1.387 1.381 1.247 1.238 1.229 1.220 1.211 0.793 0.776 0.758
C5H11
F
CHO
4d
0.93 1.91 1.93 1.96 1.99 2.14 1.99 2.00 2.13 3.21 2.22 3.96 2.98
fl (ppm)

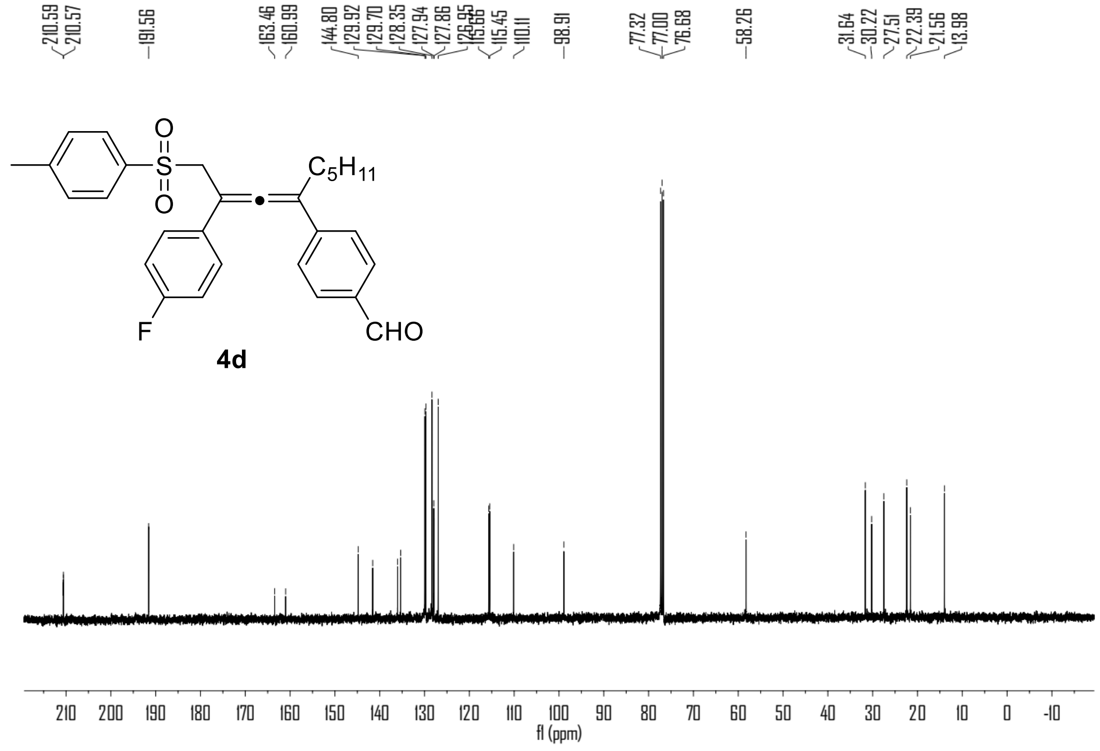
210.59 210.57 191.56 163.46 160.99 144.80 129.92 129.70 128.35 127.94 127.86 115.66 115.45 110.11 98.91 77.32 77.00 76.68 58.26 31.64 30.22 27.51 22.39 21.56 13.98
C5H11
F
CHO
4d
fl (ppm)

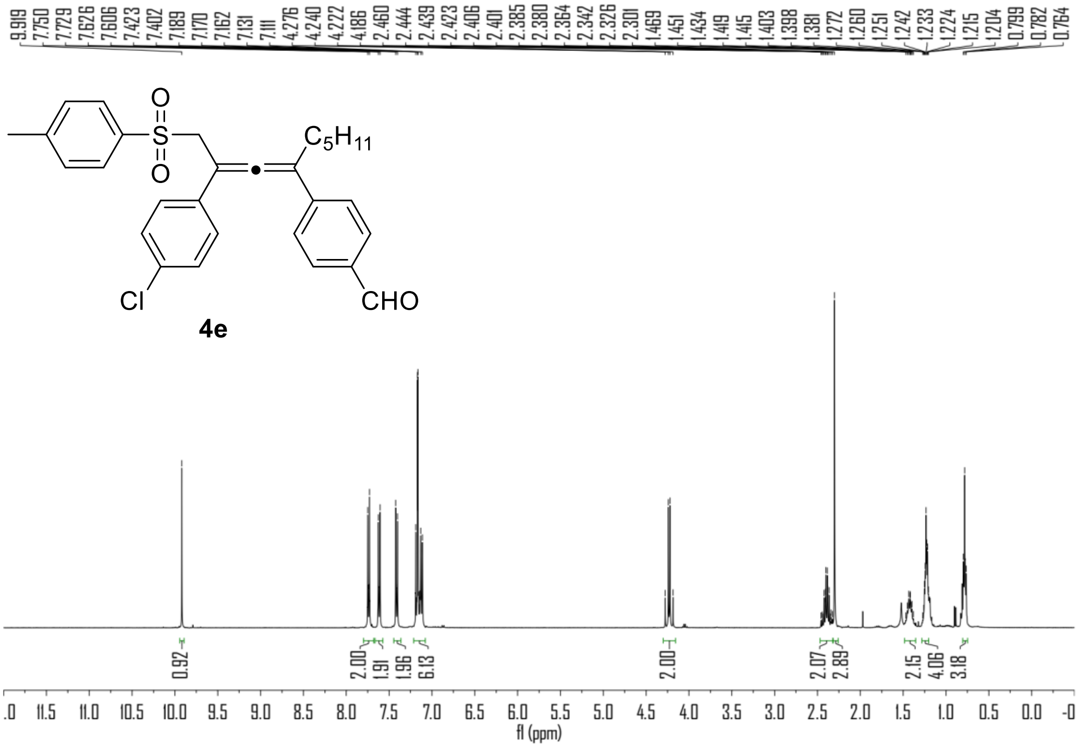
9.919 7.750 7.729 7.626 7.606 7.423 7.402 7.189 7.170 7.162 7.131 7.111 4.276 4.240 4.222 4.186 2.460 2.444 2.439 2.423 2.406 2.401 2.385 2.380 2.364 2.342 2.326 2.301 1.469 1.451 1.434 1.419 1.415 1.403 1.398 1.381 1.272 1.260 1.251 1.242 1.233 1.224 1.215 1.204 0.799 0.782 0.764
O
S
O
C5H11
Cl
CHO
4e
0.92
2.00
1.91
1.96
6.13
2.00
2.07
2.89
2.15
4.06
3.18
11.5 11.0 10.5 10.0 9.5 9.0 8.5 8.0 7.5 7.0 6.5 6.0 5.5 5.0 4.5 4.0 3.5 3.0 2.5 2.0 1.5 1.0 0.5 0.0
f1 (ppm)

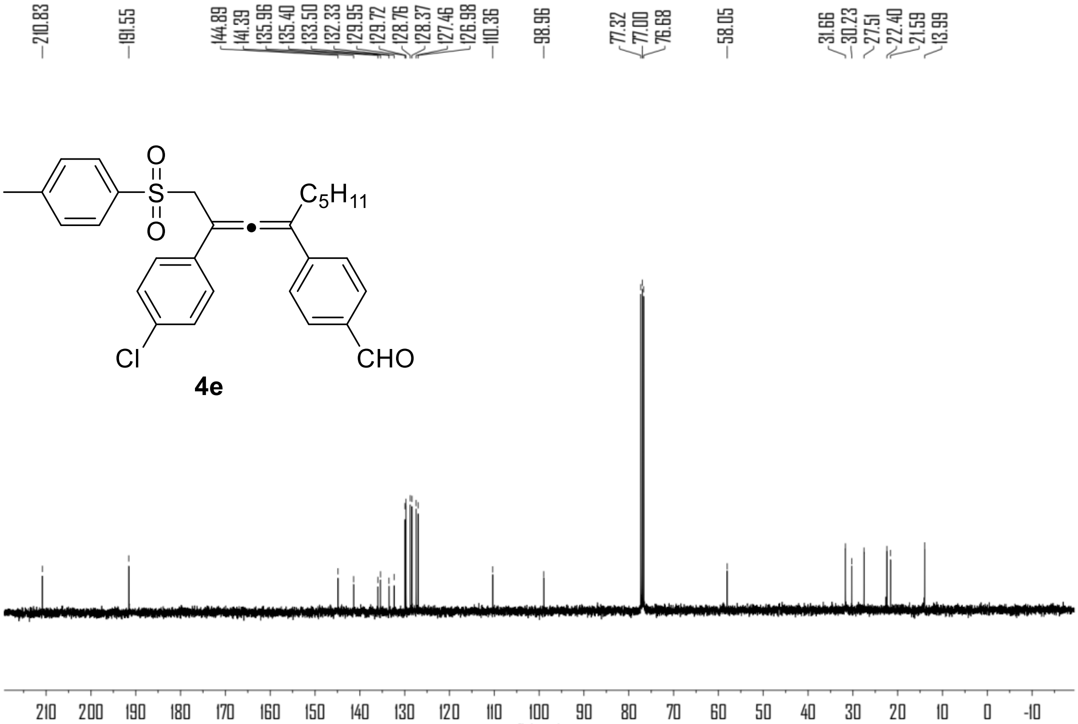
210.83 191.55 144.89 141.39 135.96 135.40 133.50 132.33 129.95 129.72 128.76 128.37 127.46 126.98 110.36 98.96 77.32 77.00 76.68 58.05 31.66 30.23 27.51 22.40 21.59 13.99
O
S
O
C5H11
Cl
CHO
4e
210 200 190 180 170 160 150 140 130 120 110 100 90 80 70 60 50 40 30 20 10 0 -10
f1 (ppm)

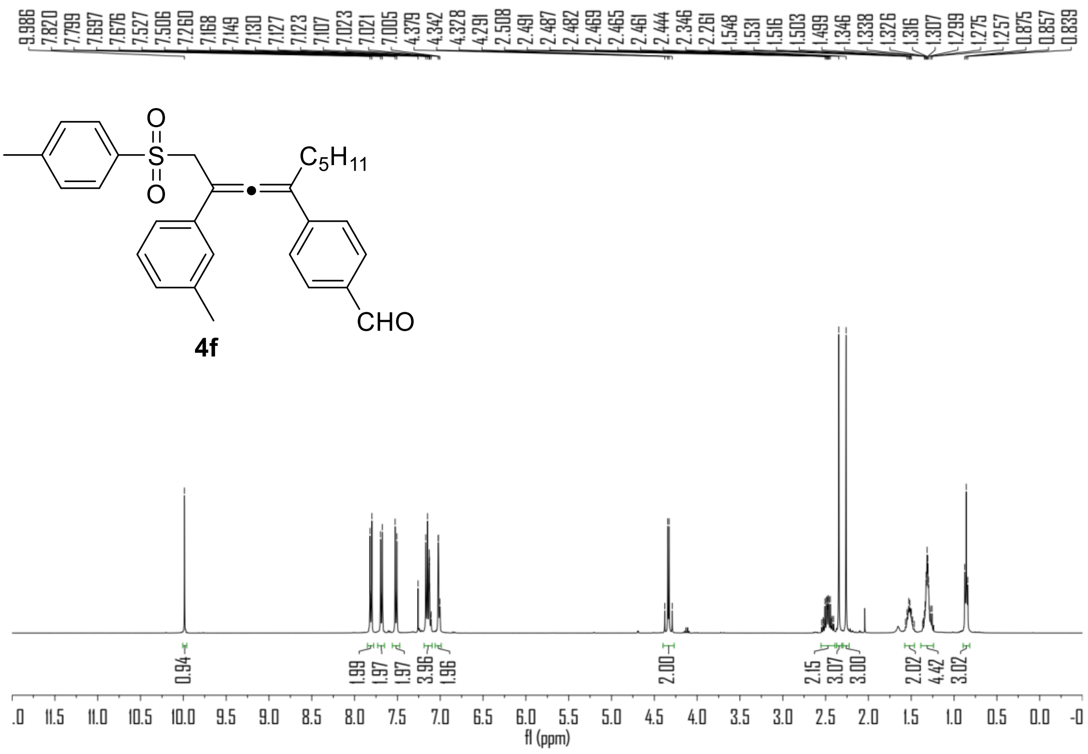
9.986 7.820 7.799 7.697 7.676 7.527 7.506 7.260 7.168 7.149 7.130 7.127 7.123 7.107 7.023 7.021 7.005 4.379 4.342 4.328 4.291 2.508 2.491 2.487 2.482 2.469 2.465 2.461 2.444 2.346 2.261 1.548 1.531 1.516 1.503 1.499 1.346 1.338 1.326 1.316 1.307 1.299 1.275 1.257 0.875 0.857 0.839
C5H11
CHO
4f
0.94
1.99 1.97 1.97 3.96 1.96
2.00
2.15 3.07 3.00
2.02 4.42 3.02
f1 (ppm)

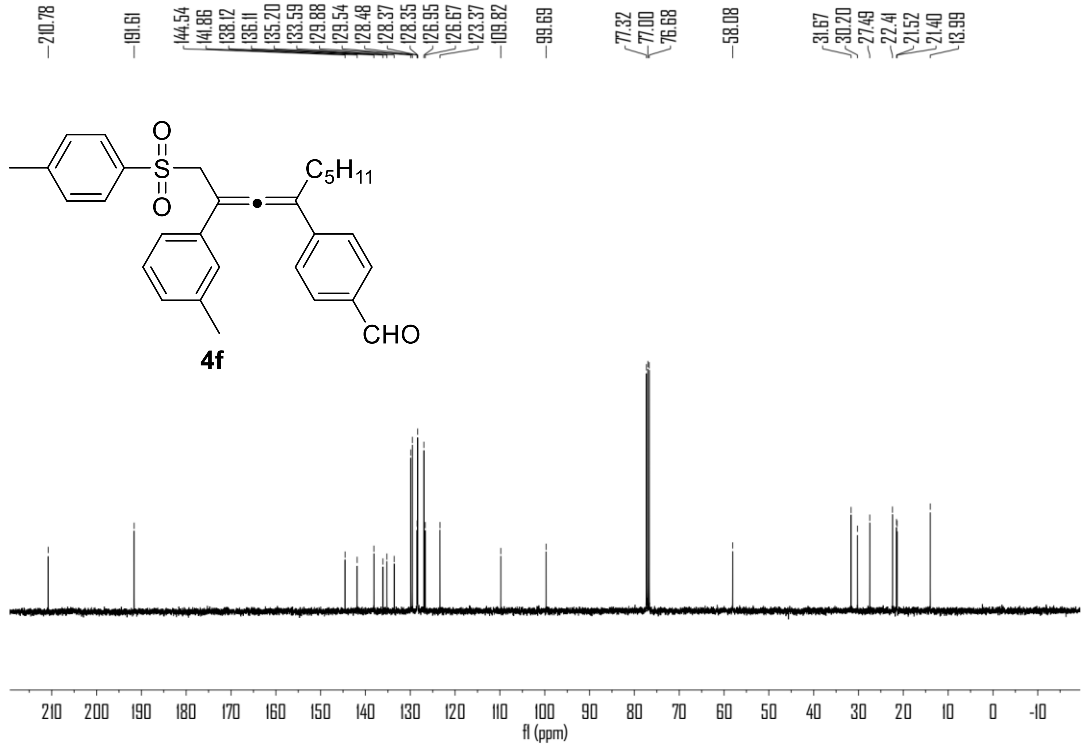
210.78
191.61
144.54 141.86 138.12 136.11 135.20 133.59 129.88 129.54 128.48 128.37 128.35 126.95 126.67 123.37 109.82
99.69
77.32 77.00 76.68
58.08
31.67 30.20 27.49 22.41 21.52 21.40 13.99
C5H11
CHO
4f
f1 (ppm)

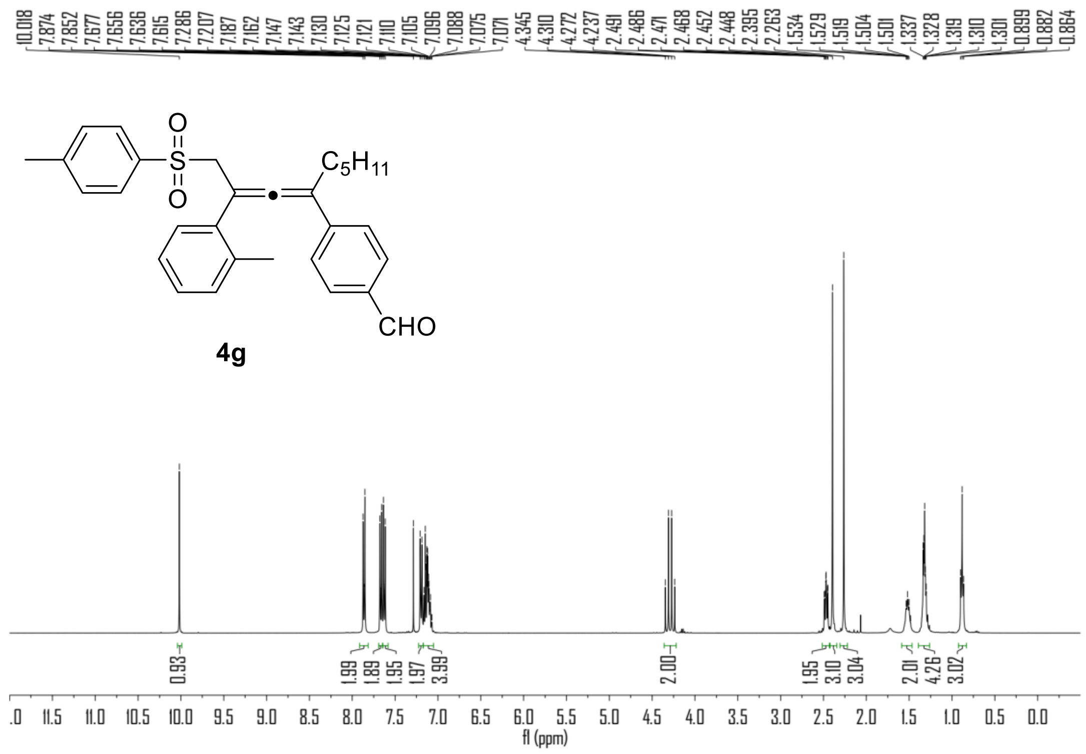

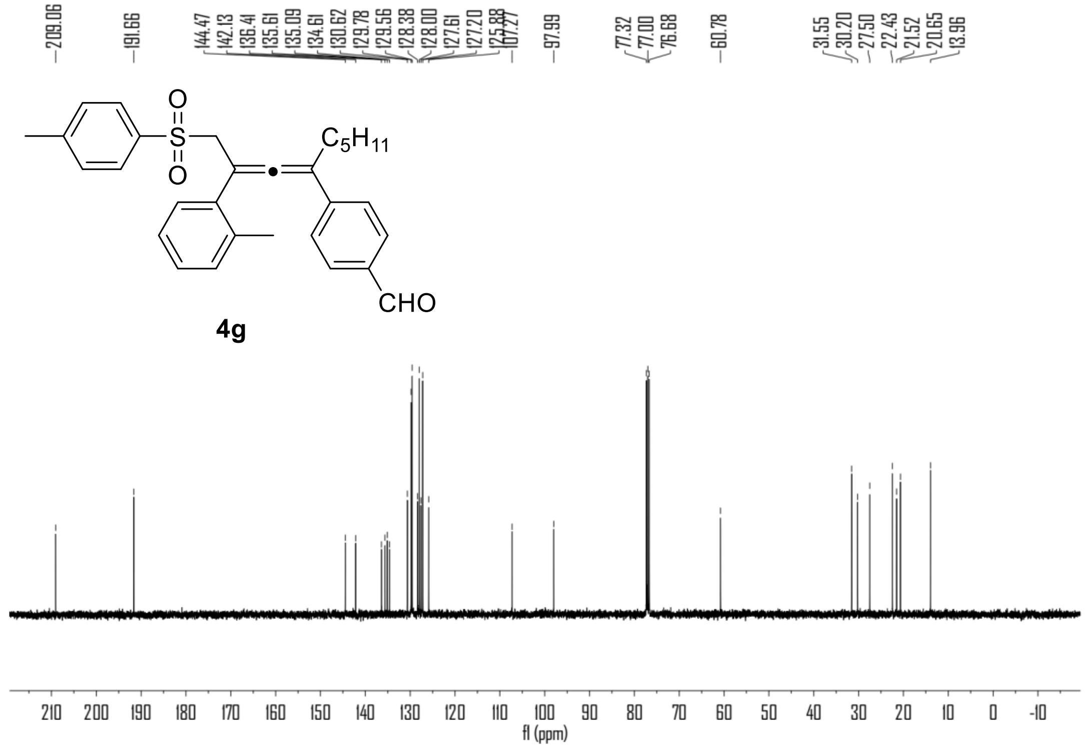

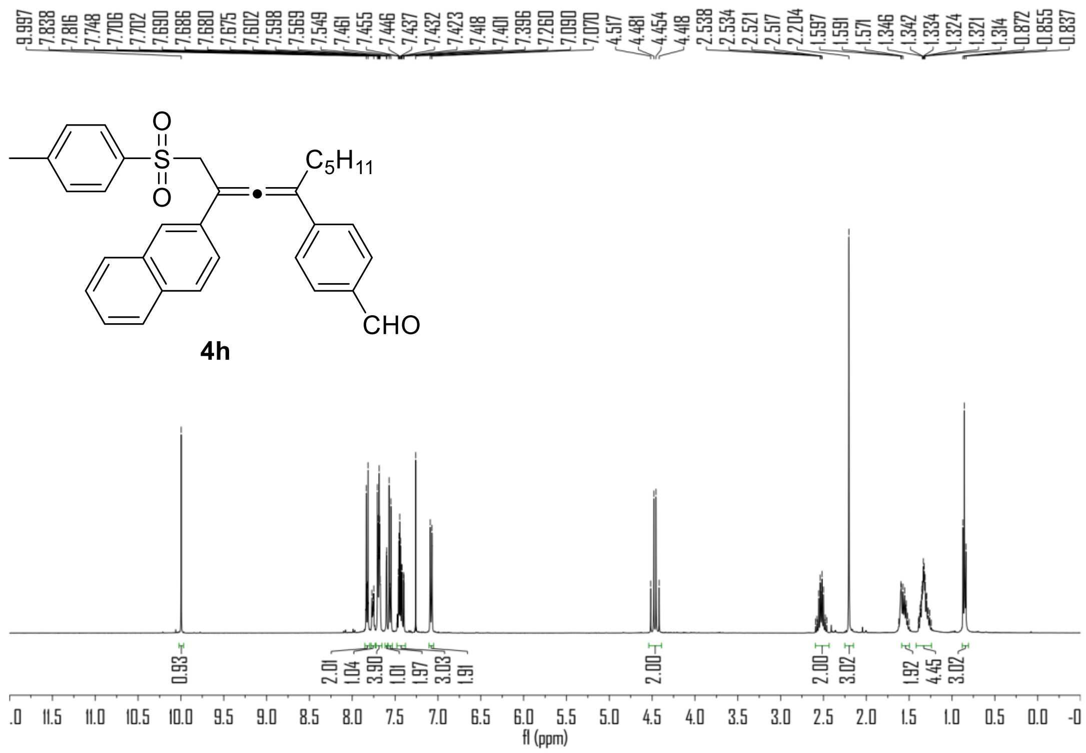
9.997 7.838 7.816 7.748 7.706 7.702 7.690 7.686 7.680 7.675 7.602 7.598 7.569 7.549 7.461 7.455 7.446 7.437 7.432 7.423 7.418 7.401 7.396 7.260 7.090 7.070 4.517 4.481 4.454 4.418 2.538 2.534 2.521 2.517 2.204 1.597 1.591 1.571 1.346 1.342 1.334 1.324 1.321 1.314 0.872 0.855 0.837
O
S
O
C_5H_{11}
CHO
4h
0.93 2.01 1.04 3.90 1.01 1.97 3.03 1.91 2.00 2.00 3.02 1.92 4.45 3.02
fl (ppm)

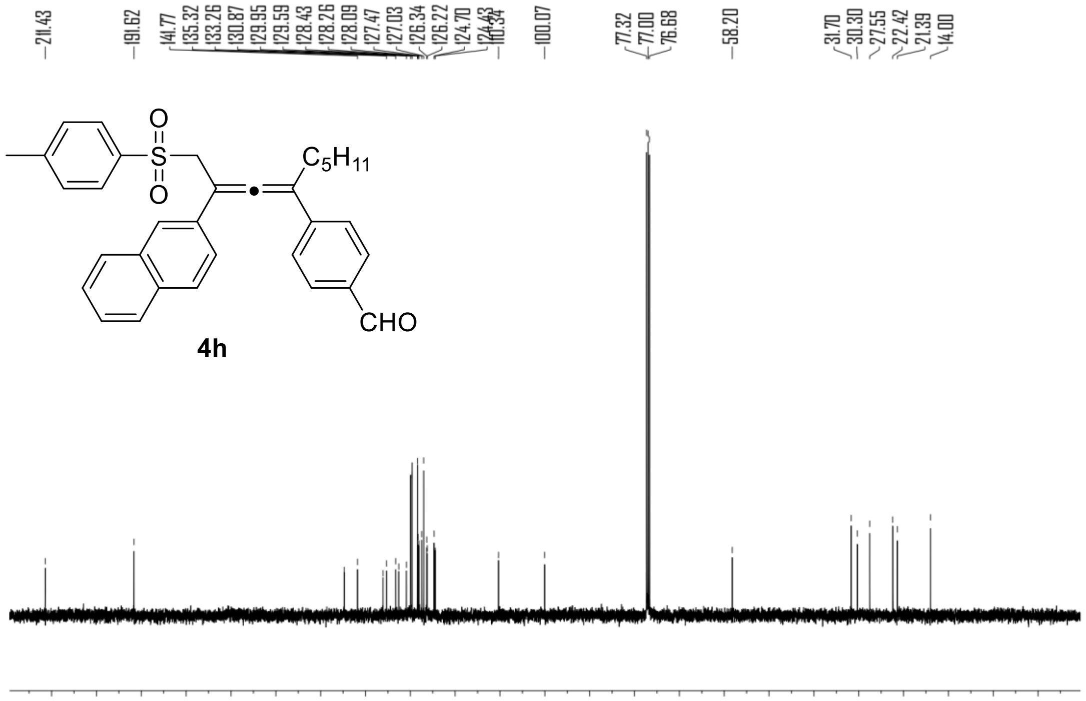
211.43 191.62 141.77 135.32 133.26 130.87 129.95 129.59 128.43 128.26 128.09 127.47 127.03 126.34 126.22 124.70 124.43 110.34 100.07 77.32 77.00 76.68 58.20 31.70 30.30 27.55 22.42 21.39 14.00
O
S
O
C_5H_{11}
CHO
4h
fl (ppm)

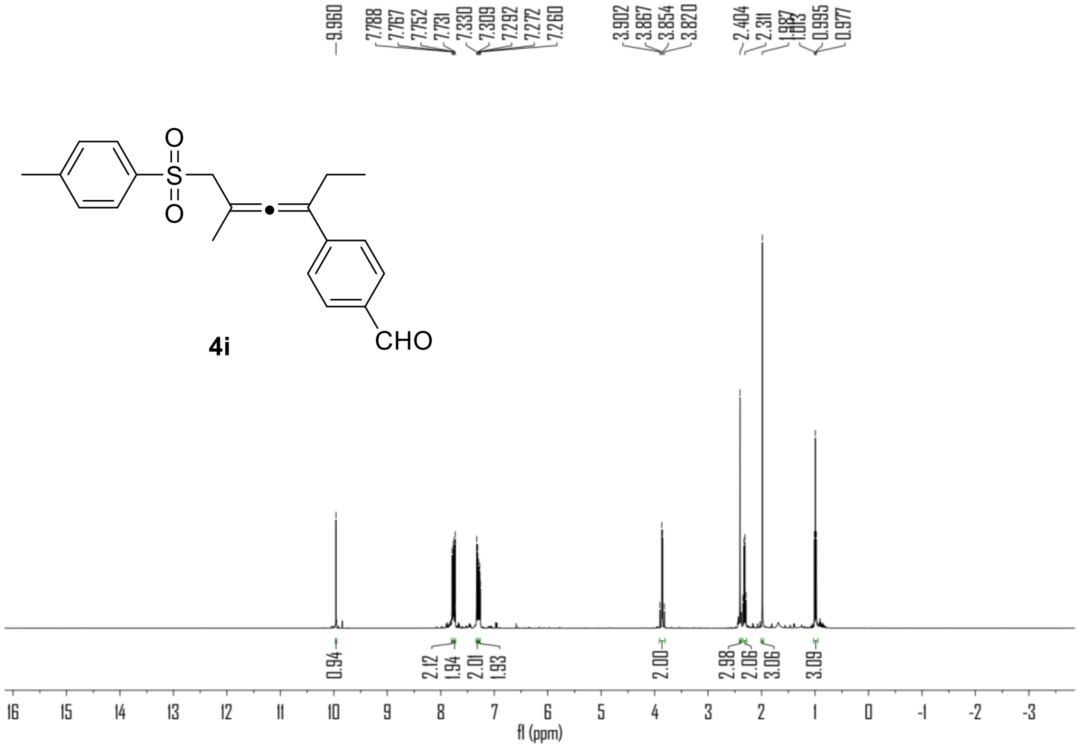

4i

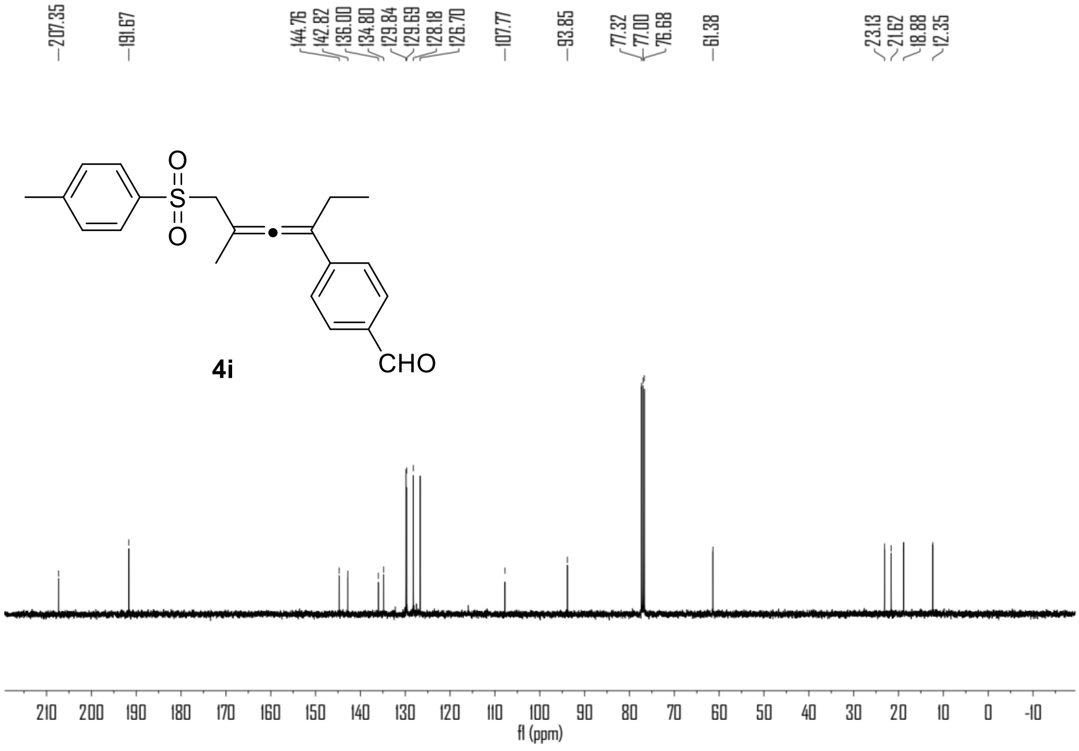

4i

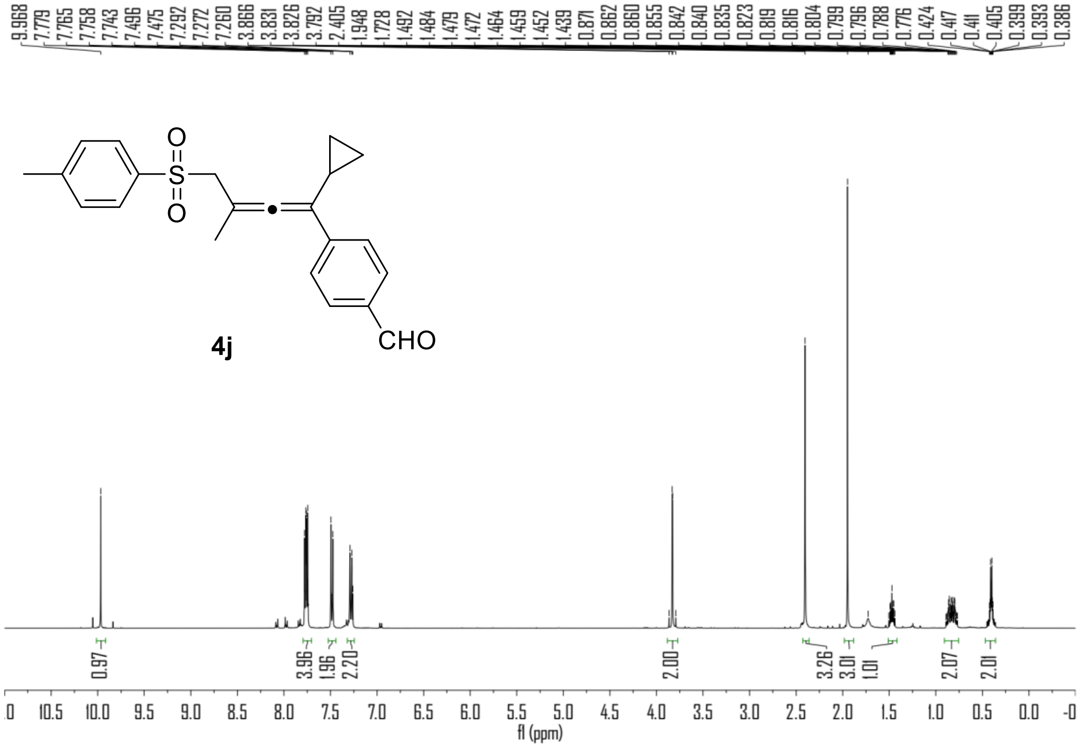
4j
CHO
fl (ppm)

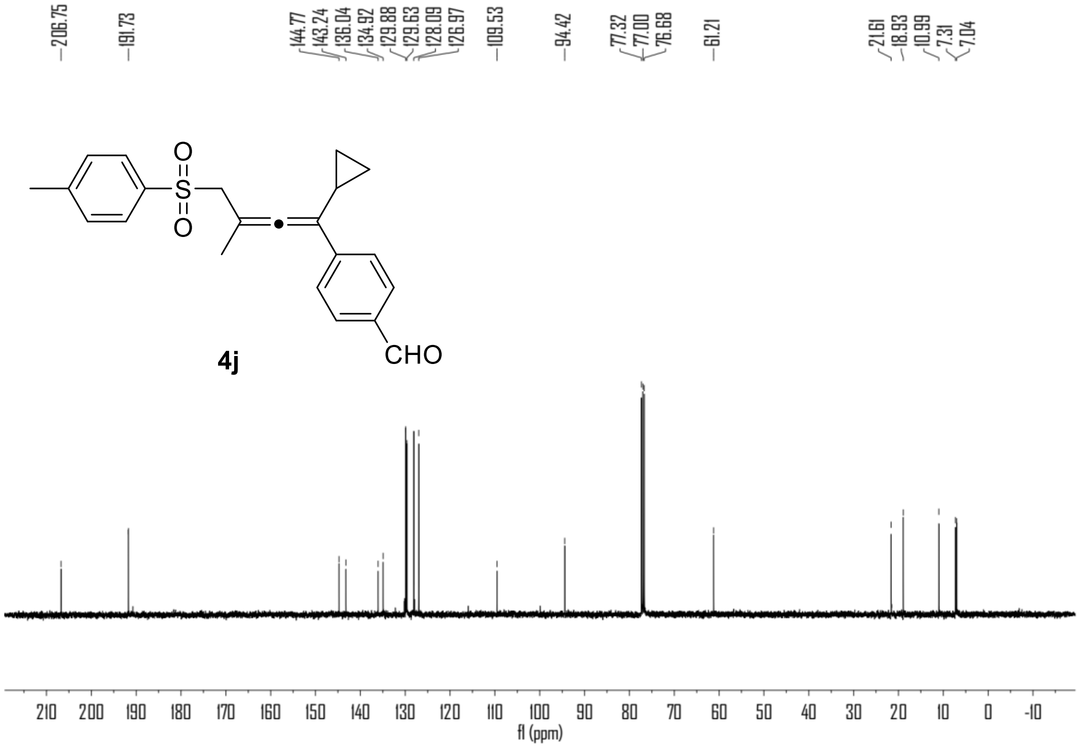
4j
CHO
fl (ppm)

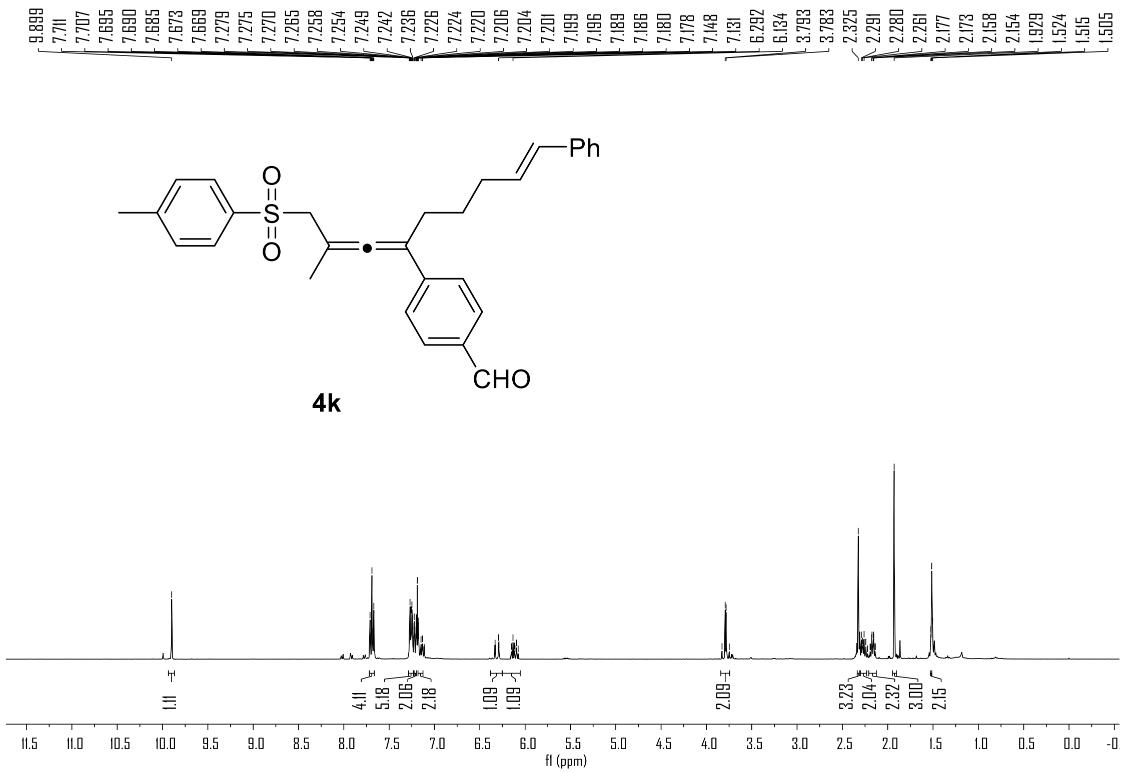
9.899 7.711 7.707 7.695 7.690 7.685 7.673 7.669 7.279 7.275 7.270 7.265 7.258 7.254 7.249 7.242 7.236 7.226 7.224 7.220 7.206 7.204 7.201 7.199 7.196 7.189 7.186 7.180 7.178 7.148 7.131 6.292 6.134 3.793 3.783 2.325 2.291 2.280 2.261 2.177 2.173 2.158 2.154 1.929 1.524 1.515 1.505
O
S
O
Ph
CHO
4k
1.11
4.11
5.18
2.06
2.18
1.09
1.09
2.09
3.23
2.04
2.32
3.00
2.15
11.5 11.0 10.5 10.0 9.5 9.0 8.5 8.0 7.5 7.0 6.5 6.0 5.5 5.0 4.5 4.0 3.5 3.0 2.5 2.0 1.5 1.0 0.5 0.0 -0
f1 (ppm)

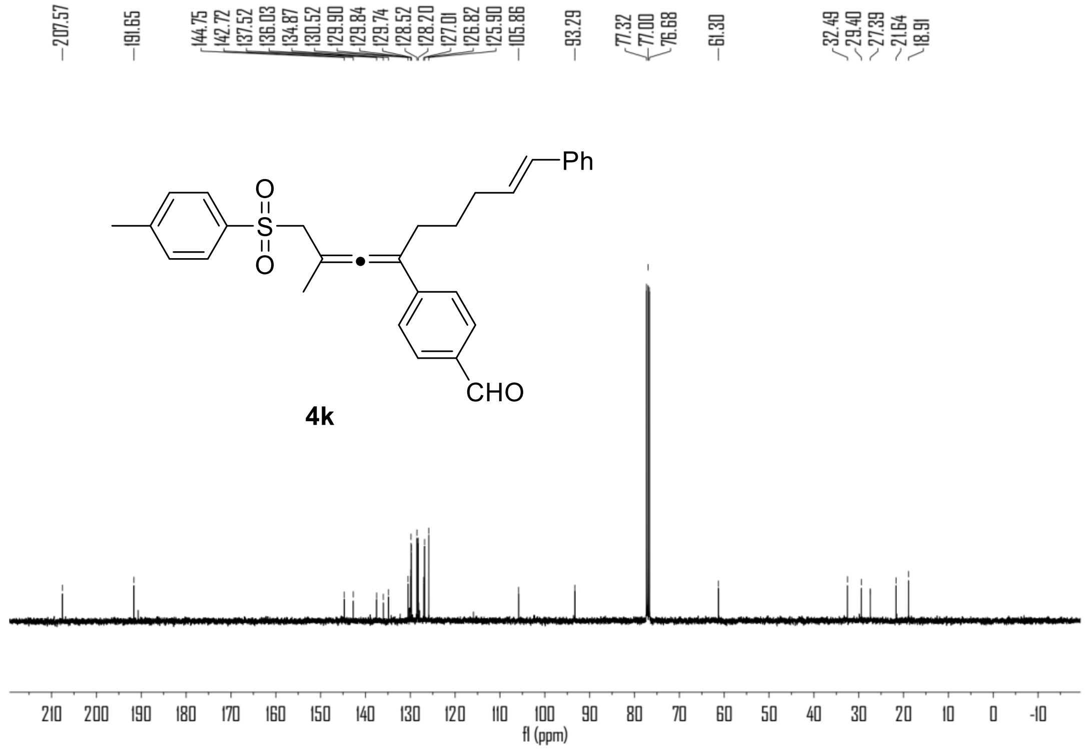
207.57 191.65 144.75 142.72 137.52 136.03 134.87 130.52 129.90 129.84 129.74 128.52 128.20 127.01 126.82 125.90 105.86 93.29 77.32 77.00 76.68 61.30 32.49 29.40 27.39 21.64 18.91
O
S
O
Ph
CHO
4k
210 200 190 180 170 160 150 140 130 120 110 100 90 80 70 60 50 40 30 20 10 0 -10
f1 (ppm)

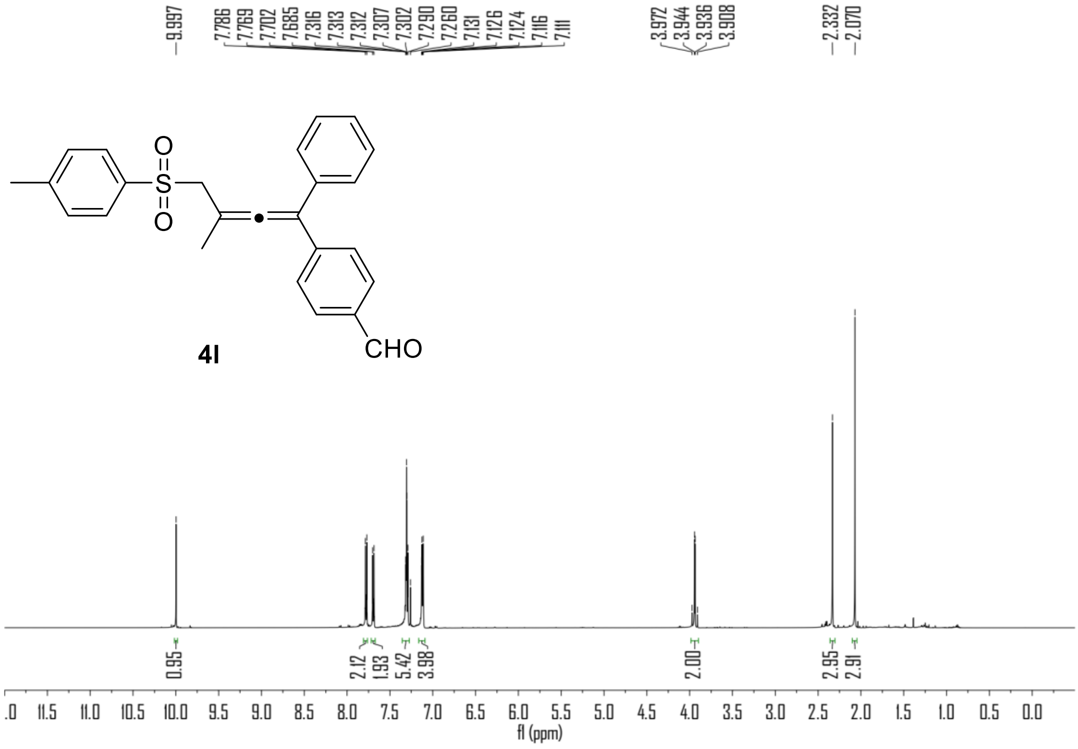

9.997
7.786 7.769 7.702 7.685 7.316 7.313 7.312 7.307 7.302 7.290 7.260 7.131 7.126 7.124 7.116 7.111
3.972 3.944 3.936 3.908
2.332
2.070
O
S
O
4l
CHO
0.95
2.12 1.93 5.42 3.98
2.00
2.95 2.91
f1 (ppm)

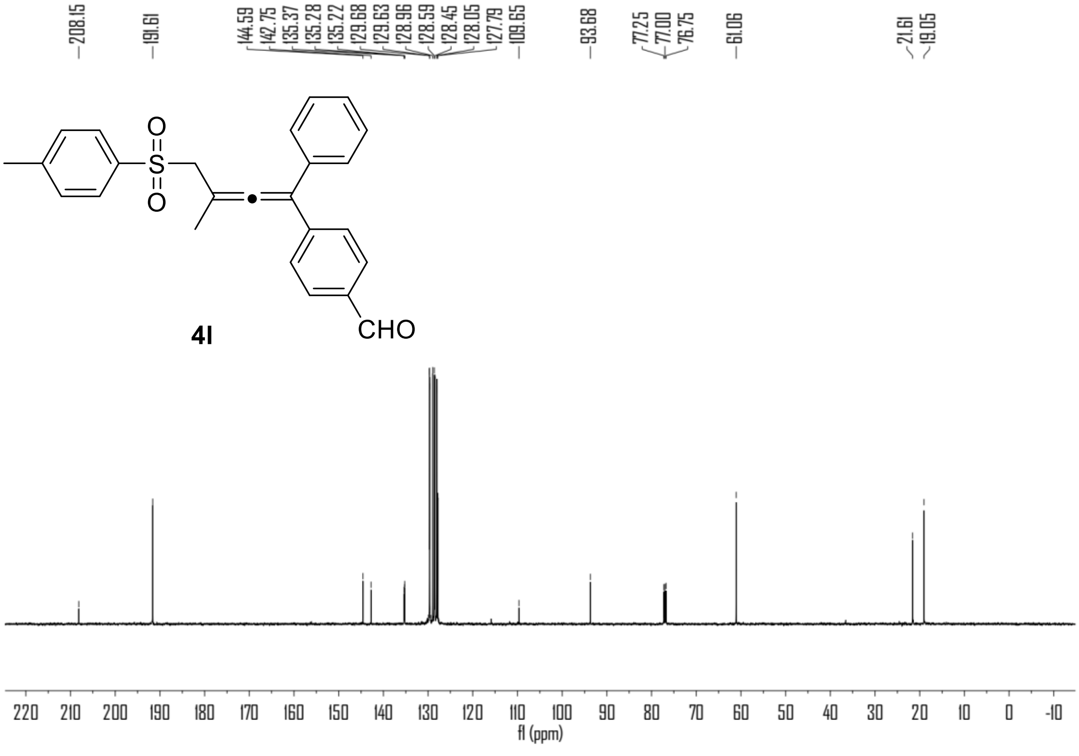

208.15
191.61
144.59 142.75 135.37 135.28 135.22 129.68 129.63 128.96 128.59 128.45 128.05 127.79
109.65
93.68
77.25 77.00 76.75
61.06
21.61 19.05
O
S
O
4l
CHO
f1 (ppm)

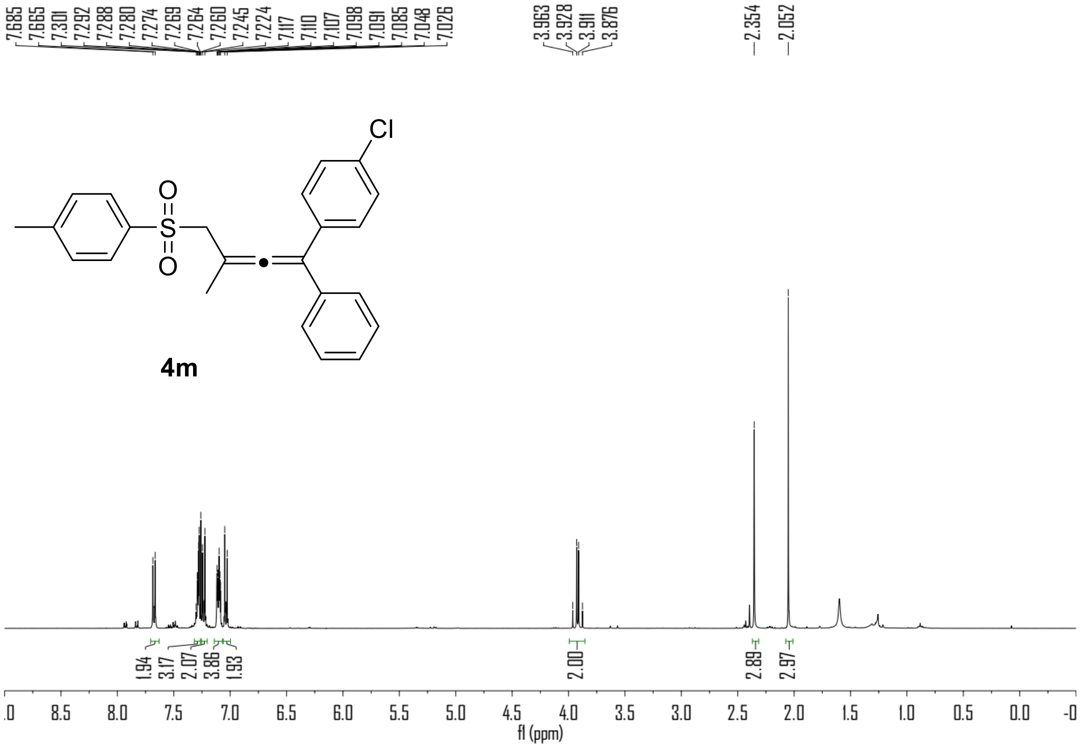

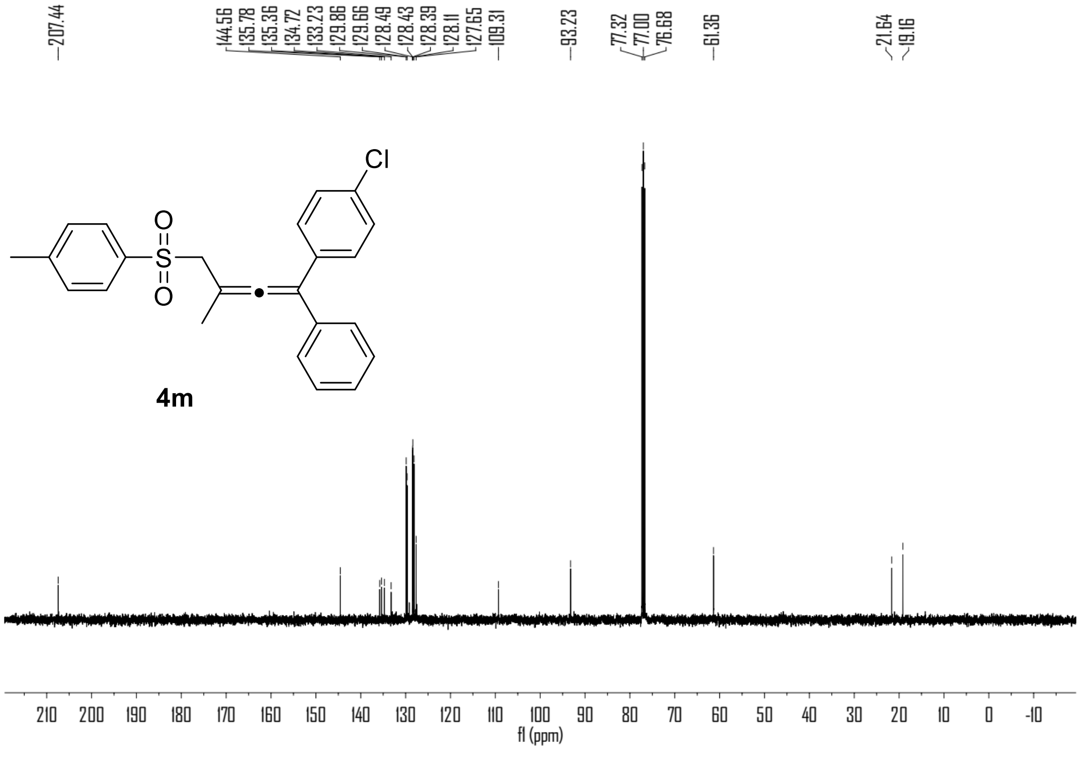

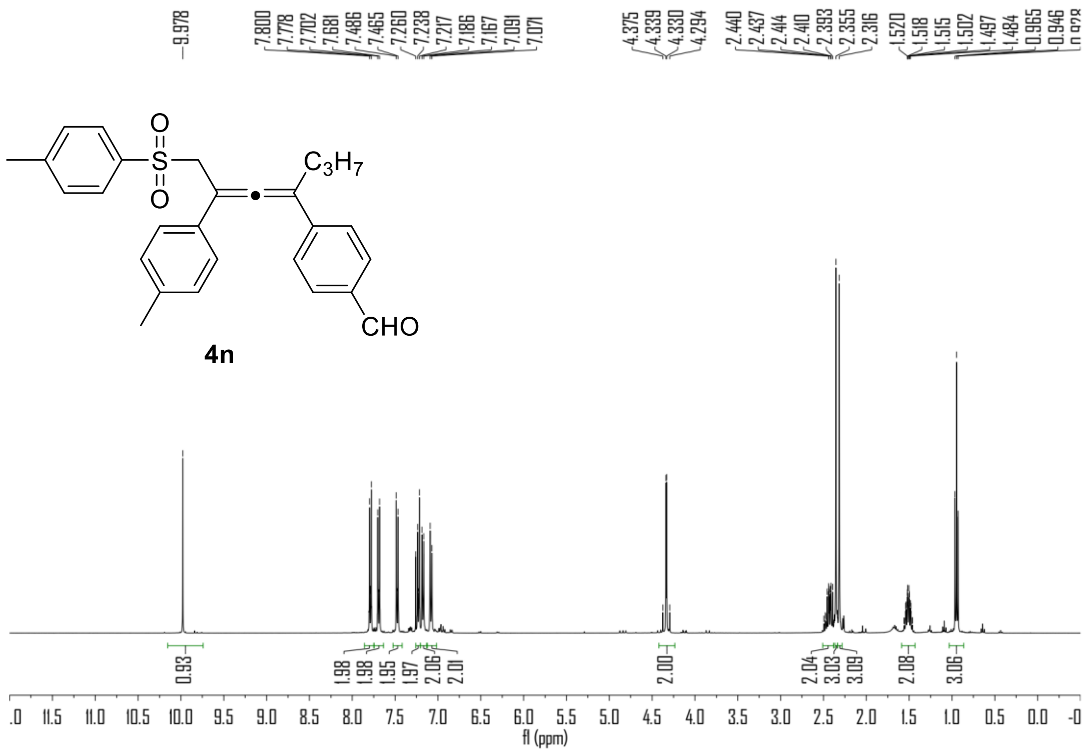
C3H7
CHO
4n
fl (ppm)

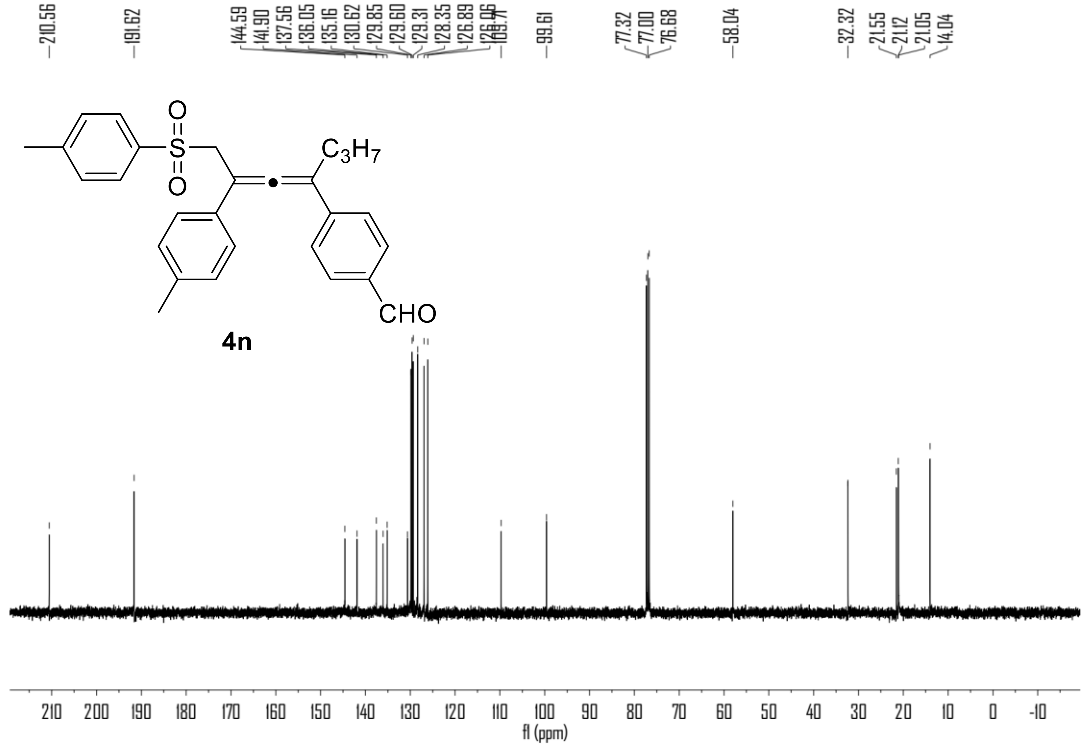
C3H7
CHO
4n
fl (ppm)

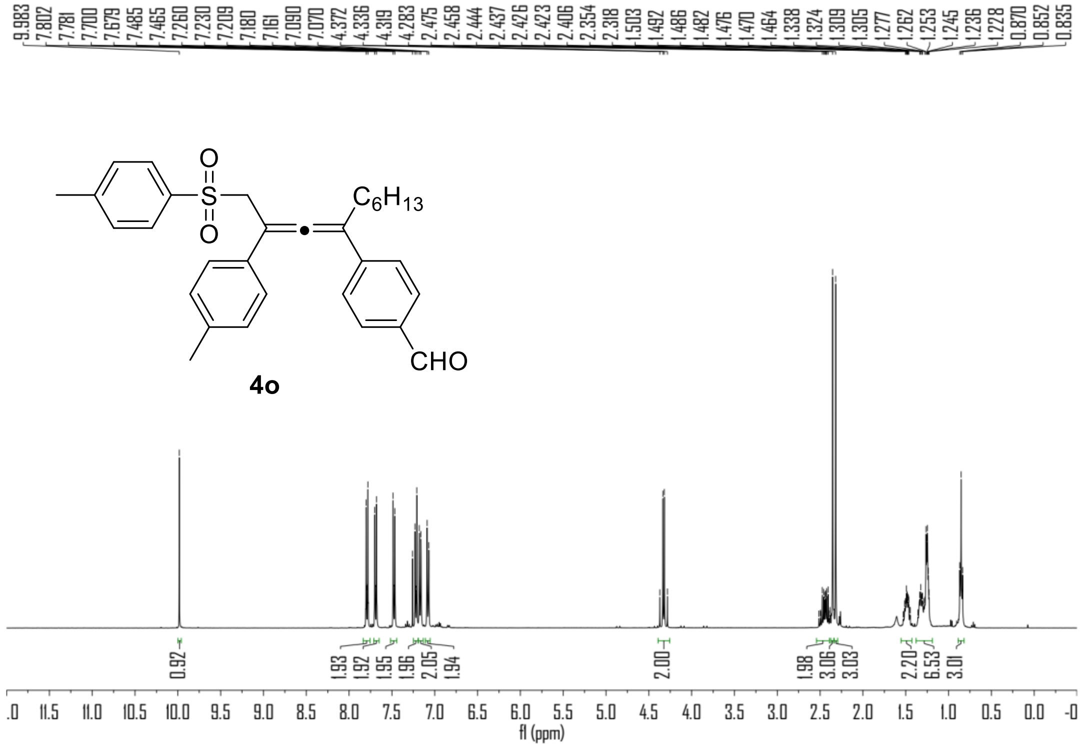
9.983
7.802
7.781
7.700
7.679
7.485
7.465
7.260
7.230
7.209
7.180
7.161
7.090
7.070
4.372
4.336
4.319
4.283
2.475
2.458
2.444
2.437
2.426
2.423
2.406
2.354
2.318
1.503
1.492
1.486
1.482
1.476
1.470
1.464
1.338
1.324
1.309
1.305
1.277
1.262
1.253
1.245
1.236
1.228
0.870
0.852
0.835
O
S
O
C6H13
CHO
4o
0.92
1.93
1.92
1.95
1.96
2.05
1.94
2.00
1.98
3.06
3.03
2.20
6.53
3.01
f1 (ppm)

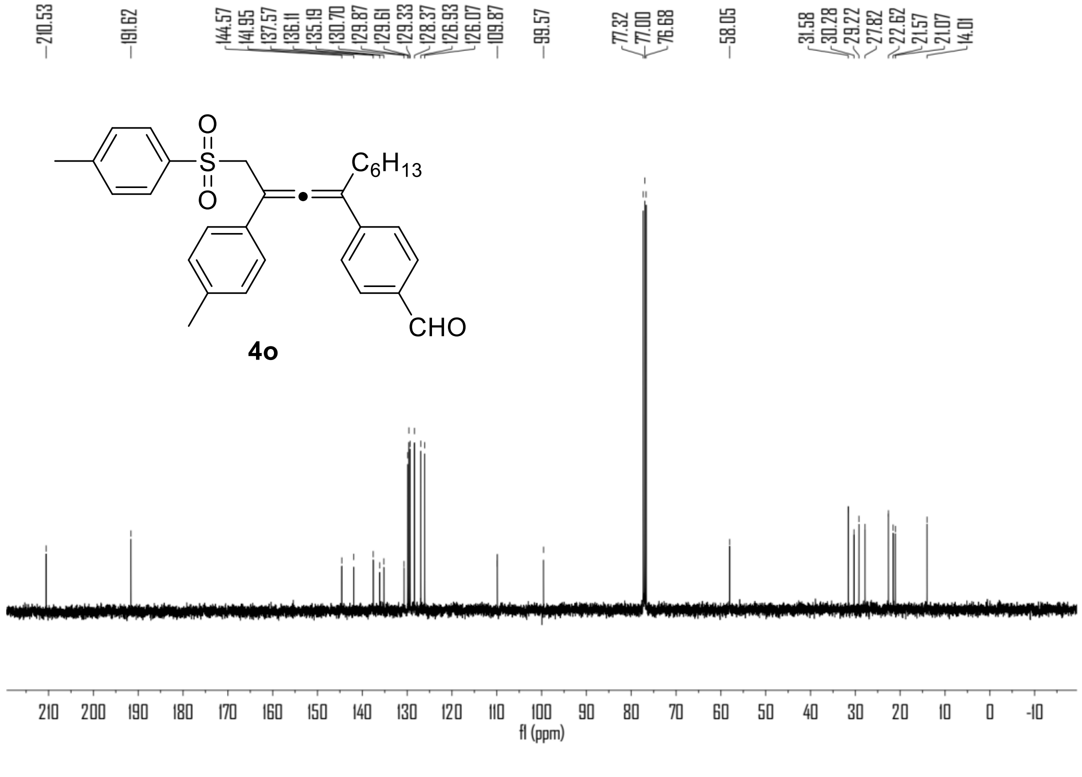
210.53
191.62
144.57
141.95
137.57
136.11
135.19
130.70
129.87
129.61
129.33
128.37
126.93
126.07
109.87
99.57
77.32
77.00
76.68
58.05
31.58
30.28
29.22
27.82
22.62
21.57
21.07
14.01
O
S
O
C6H13
CHO
4o
f1 (ppm)

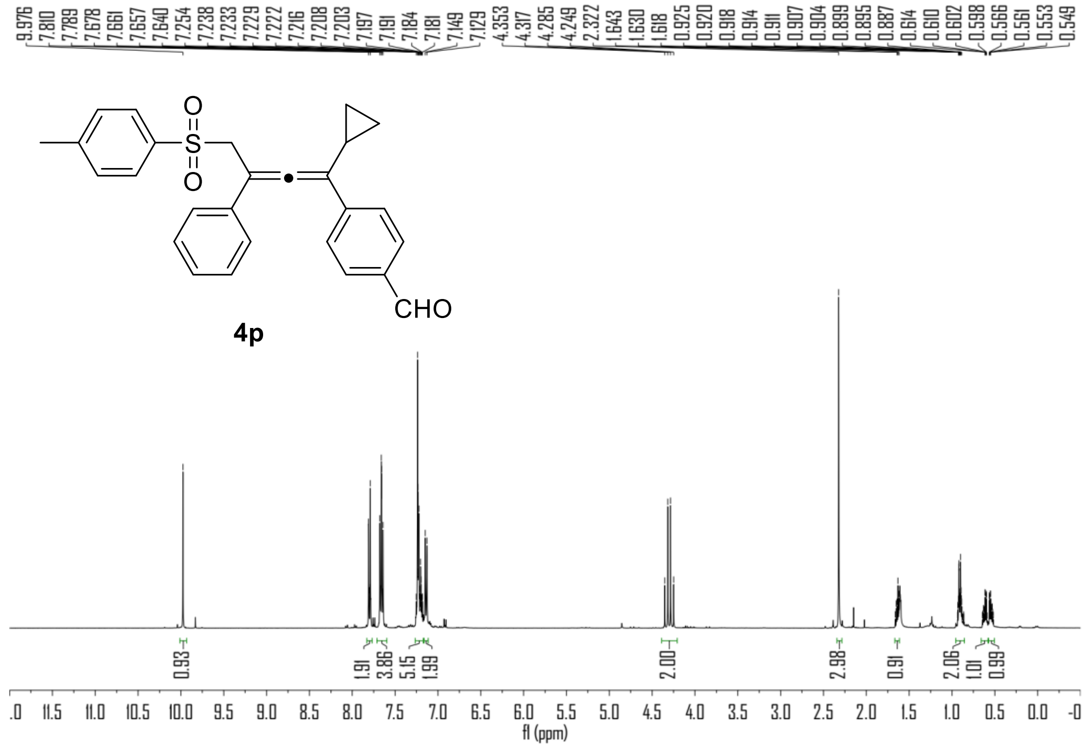
9.976 7.810 7.789 7.678 7.661 7.657 7.640 7.254 7.238 7.233 7.229 7.222 7.216 7.208 7.203 7.197 7.191 7.184 7.181 7.149 7.129 4.353 4.317 4.285 4.249 2.322 1.643 1.630 1.618 0.925 0.920 0.918 0.914 0.911 0.907 0.904 0.899 0.895 0.887 0.614 0.610 0.602 0.598 0.566 0.561 0.553 0.549
O
S
O
CHO
4p
0.93
1.91
3.86
5.15
1.99
2.00
2.98
0.91
2.06
1.01
0.99
fl (ppm)

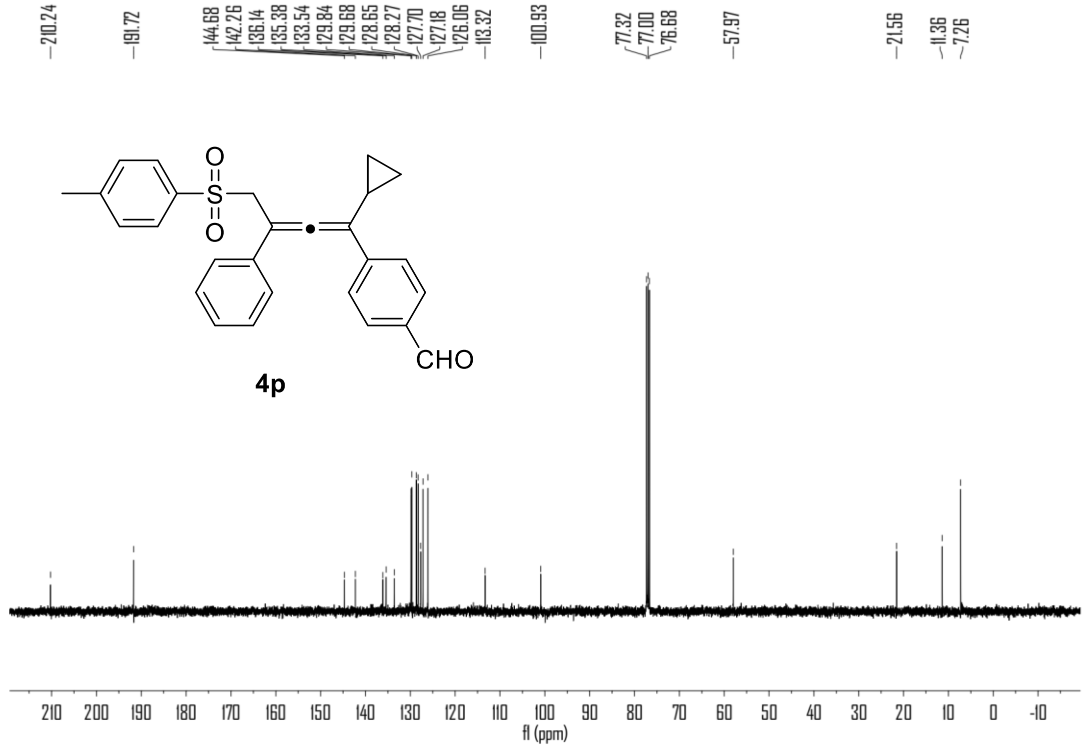
210.24 191.72 144.68 142.26 136.14 135.38 133.54 129.84 129.68 128.65 128.27 127.70 127.18 126.06 113.32 100.93 77.32 77.00 76.68 57.97 21.56 11.36 7.26
O
S
O
CHO
4p
fl (ppm)

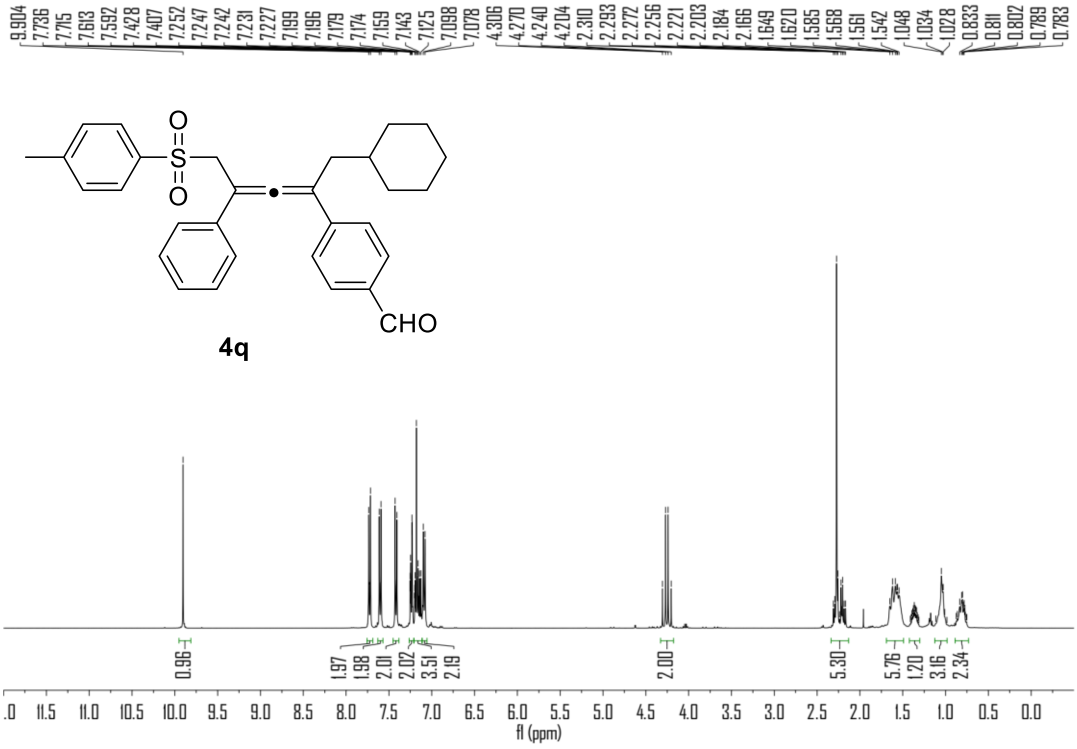
9.904 7.736 7.715 7.613 7.592 7.428 7.407 7.252 7.247 7.242 7.231 7.227 7.199 7.196 7.179 7.174 7.159 7.143 7.125 7.098 7.078 4.306 4.270 4.240 4.204 2.310 2.293 2.272 2.256 2.221 2.203 2.184 2.166 1.649 1.620 1.585 1.568 1.561 1.542 1.048 1.034 1.028 0.833 0.811 0.802 0.789 0.783
CHO
4q
0.96 1.97 1.98 2.01 2.02 3.51 2.19 2.00 5.30 5.76 1.20 3.16 2.34
fl (ppm)

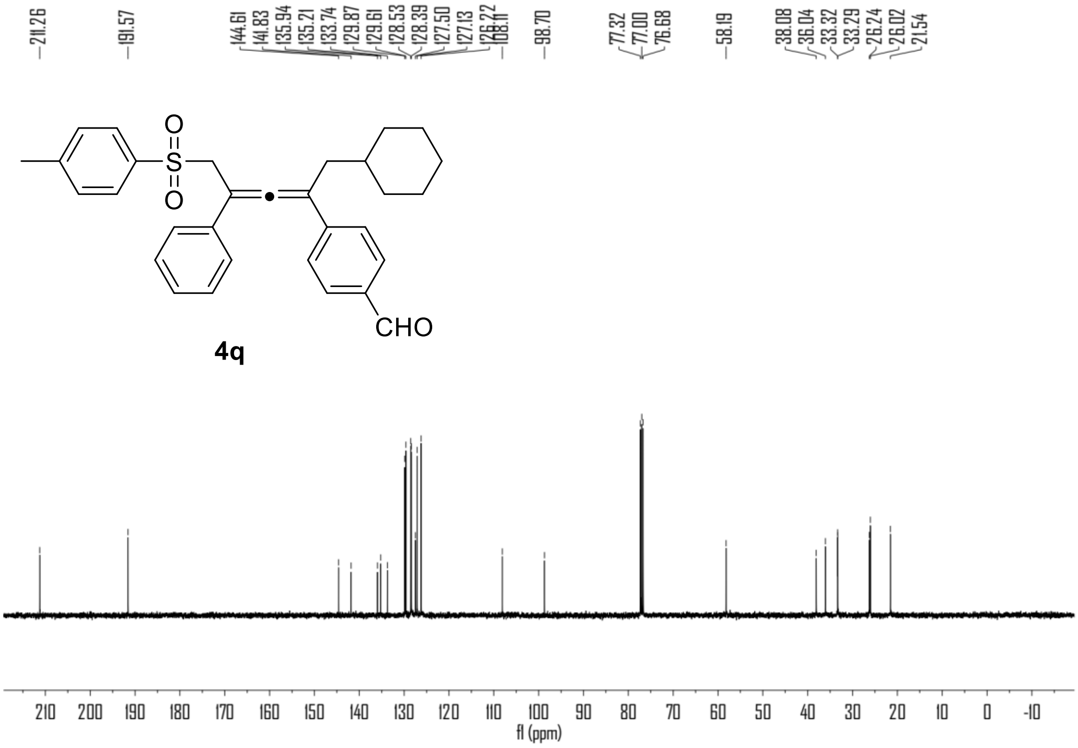
211.26 191.57 144.61 141.83 135.94 135.21 133.74 129.87 129.61 128.53 128.39 127.50 127.13 126.72 108.11 98.70 77.32 77.00 76.68 58.19 38.08 36.04 33.32 33.29 26.24 26.02 21.54
CHO
4q
fl (ppm)

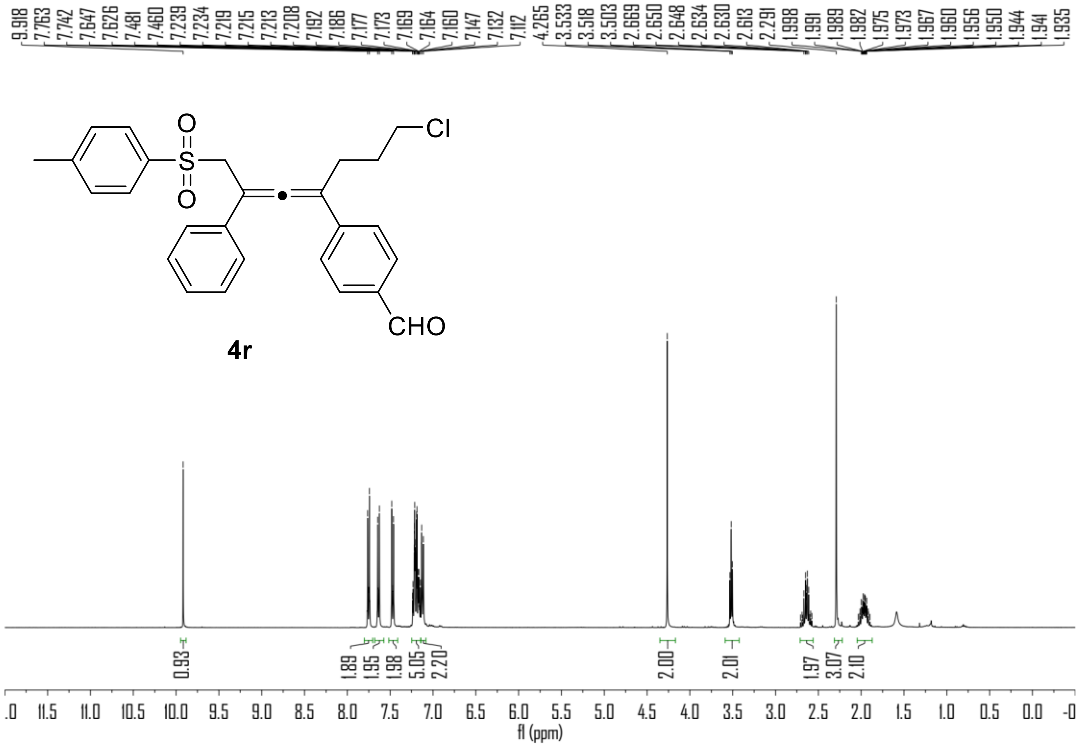
9.918
7.763
7.742
7.647
7.626
7.481
7.460
7.239
7.234
7.219
7.215
7.213
7.208
7.192
7.186
7.177
7.173
7.169
7.164
7.160
7.147
7.132
7.112
4.265
3.533
3.518
3.503
2.669
2.650
2.648
2.634
2.630
2.613
2.291
1.998
1.991
1.989
1.982
1.975
1.973
1.967
1.960
1.956
1.950
1.944
1.941
1.935
Cl
CHO
4r
0.93
1.89
1.95
1.98
5.05
2.20
2.00
2.01
1.97
3.07
2.10
fl (ppm)

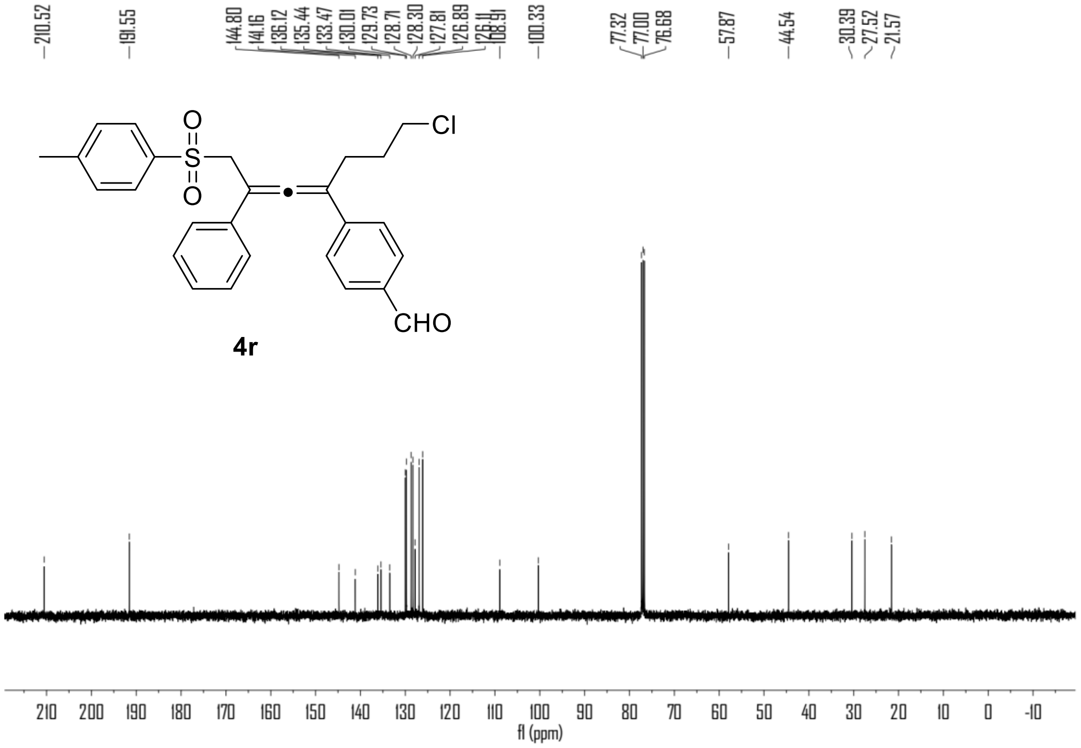
210.52
191.55
144.80
141.16
136.12
135.44
133.47
130.01
129.73
128.71
128.30
127.81
126.89
126.11
108.91
100.33
77.32
77.00
76.68
57.87
44.54
30.39
27.52
21.57
Cl
CHO
4r
fl (ppm)

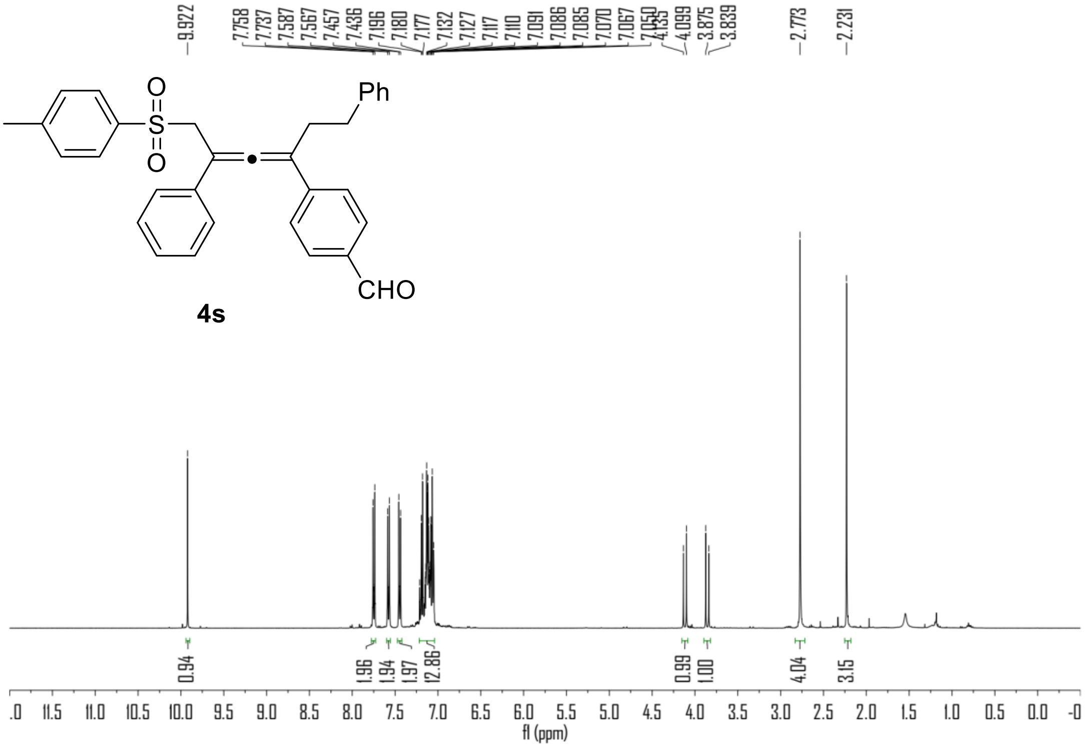

9.922
7.758
7.737
7.587
7.567
7.457
7.436
7.196
7.180
7.177
7.132
7.127
7.117
7.110
7.091
7.086
7.085
7.070
7.067
4.135
4.099
3.875
3.839
2.773
2.231
O
S
O
Ph
CHO
4s
0.94
1.96
1.94
1.97
12.86
0.99
1.00
4.04
3.15
11.5 11.0 10.5 10.0 9.5 9.0 8.5 8.0 7.5 7.0 6.5 6.0 5.5 5.0 4.5 4.0 3.5 3.0 2.5 2.0 1.5 1.0 0.5 0.0
f1 (ppm)

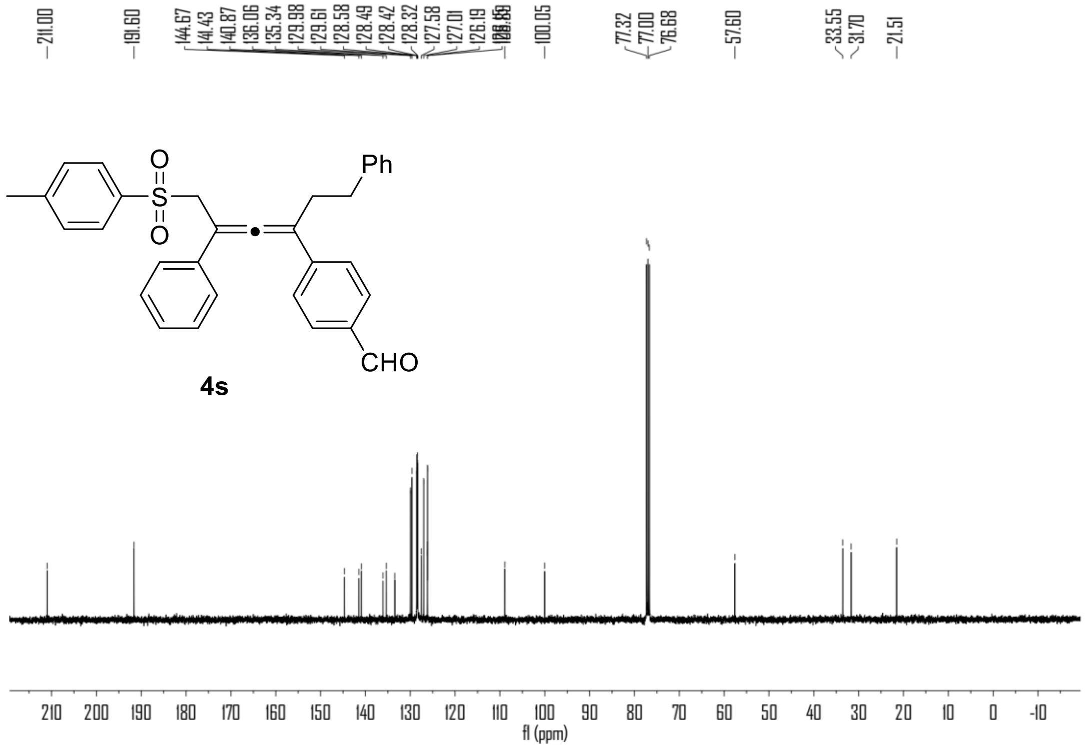

211.00
191.60
144.67
141.43
140.87
136.06
135.34
129.98
129.61
128.58
128.49
128.42
128.32
127.58
127.01
126.19
100.05
77.32
77.00
76.68
57.60
33.55
31.70
21.51
O
S
O
Ph
CHO
4s
210 200 190 180 170 160 150 140 130 120 110 100 90 80 70 60 50 40 30 20 10 0 -10
f1 (ppm)

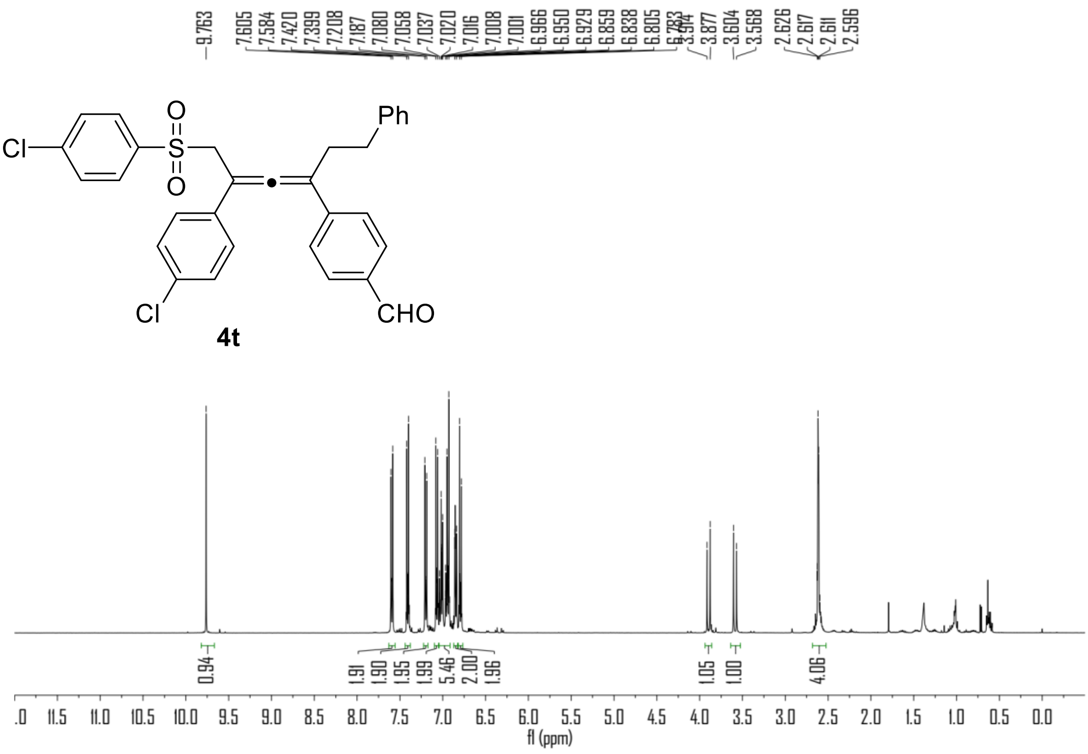
9.763
7.605
7.584
7.420
7.399
7.208
7.187
7.080
7.058
7.037
7.020
7.016
7.008
7.001
6.966
6.950
6.929
6.859
6.838
6.805
3.877
3.604
3.568
2.626
2.617
2.611
2.596
Ph
Cl
O
S
O
Cl
CHO
4t
0.94
1.91
1.90
1.95
1.99
5.46
2.00
1.96
1.05
1.00
4.06
fl (ppm)

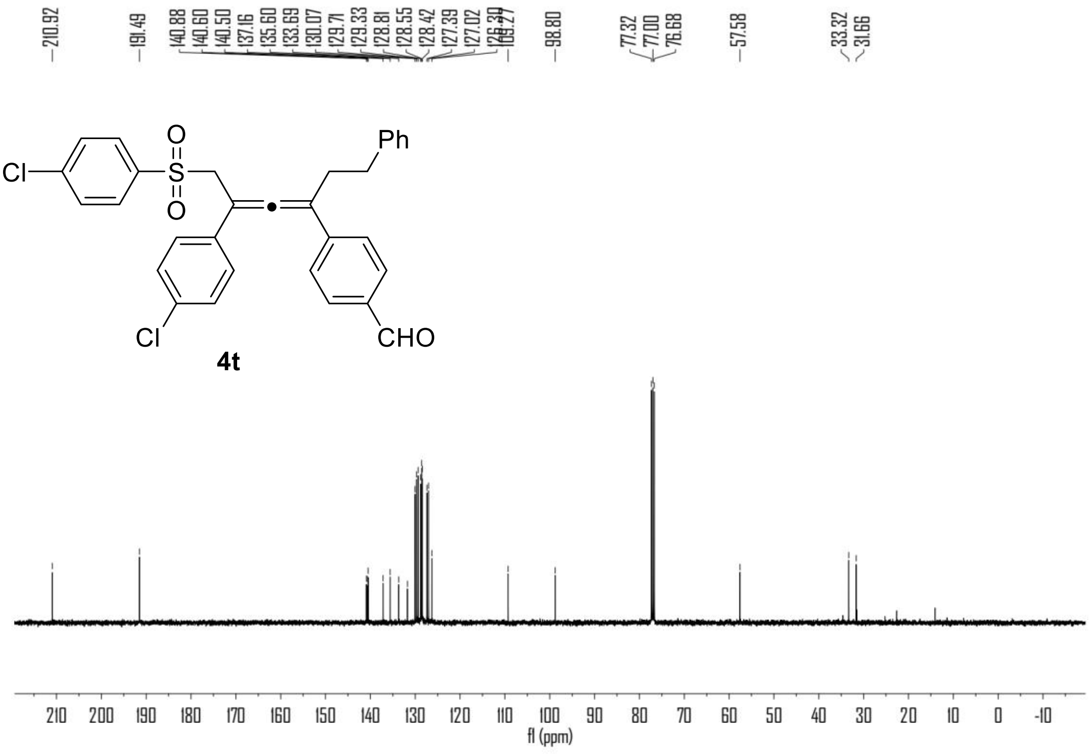
210.92
191.49
140.88
140.60
140.50
137.16
135.60
133.69
130.07
129.71
129.33
128.81
128.55
128.42
127.39
127.02
109.27
98.80
77.32
77.00
76.68
57.58
33.32
31.66
Ph
Cl
O
S
O
Cl
CHO
4t
fl (ppm)

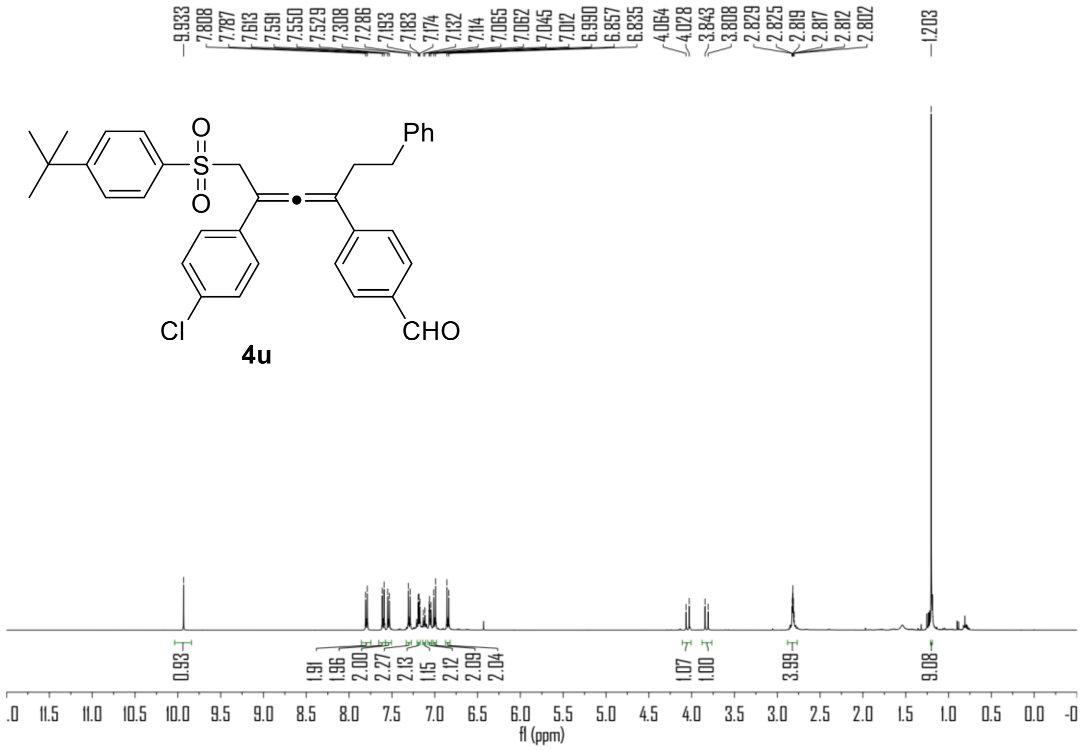
Ph
Cl
CHO
4u
f1 (ppm)

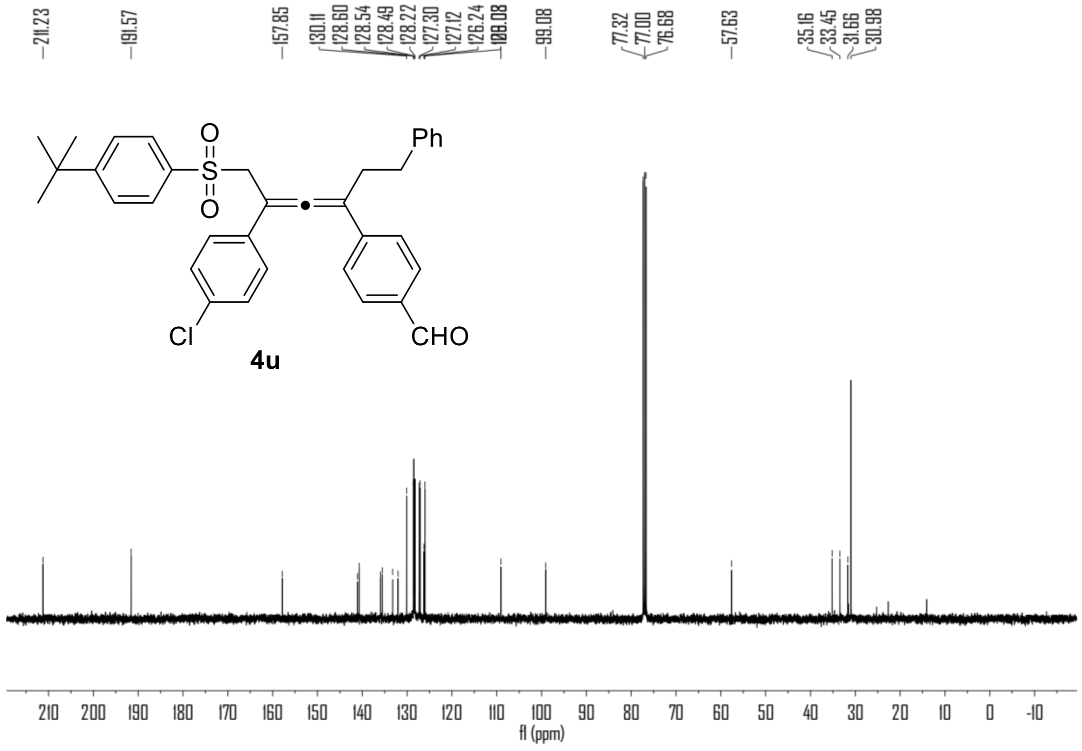
Ph
Cl
CHO
4u
f1 (ppm)

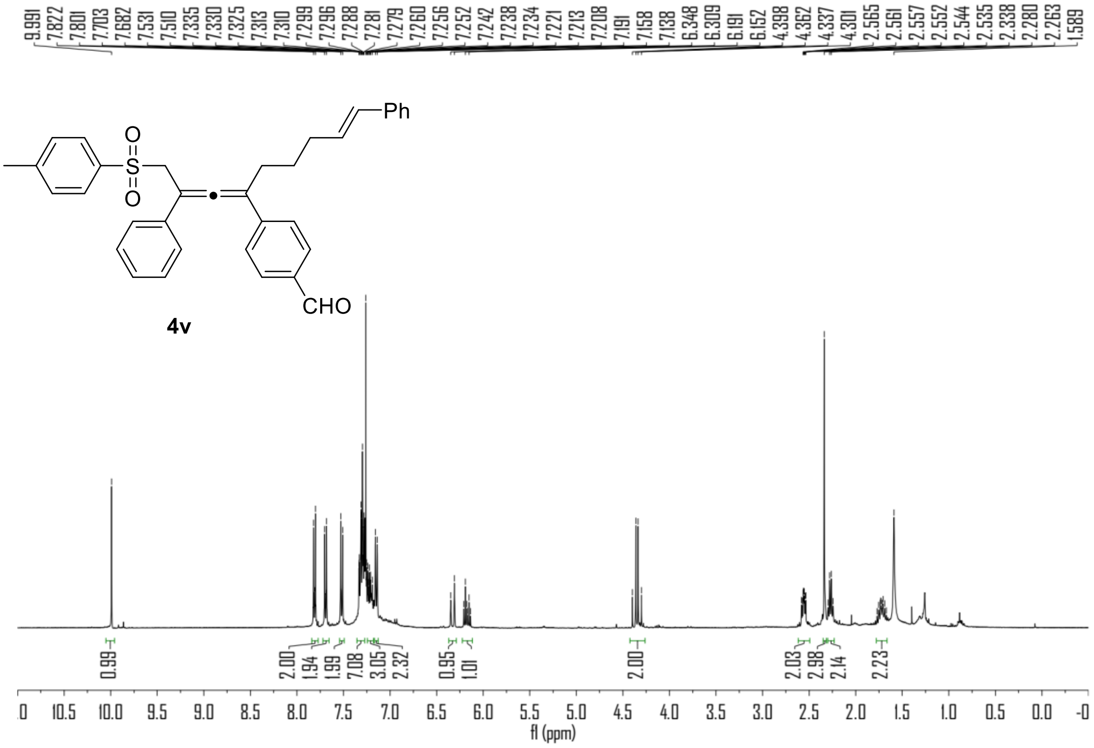
9.991
7.822
7.801
7.703
7.682
7.531
7.510
7.335
7.330
7.325
7.313
7.310
7.299
7.296
7.288
7.281
7.279
7.260
7.256
7.252
7.242
7.238
7.234
7.221
7.213
7.208
7.191
7.158
7.138
6.348
6.309
6.191
6.152
4.398
4.362
4.337
4.301
2.565
2.561
2.557
2.552
2.544
2.535
2.338
2.280
2.263
1.589
Ph
CHO
4v
0.99
2.00
1.94
1.99
7.08
3.05
2.32
0.95
1.01
2.00
2.03
2.98
2.14
2.23
fl (ppm)

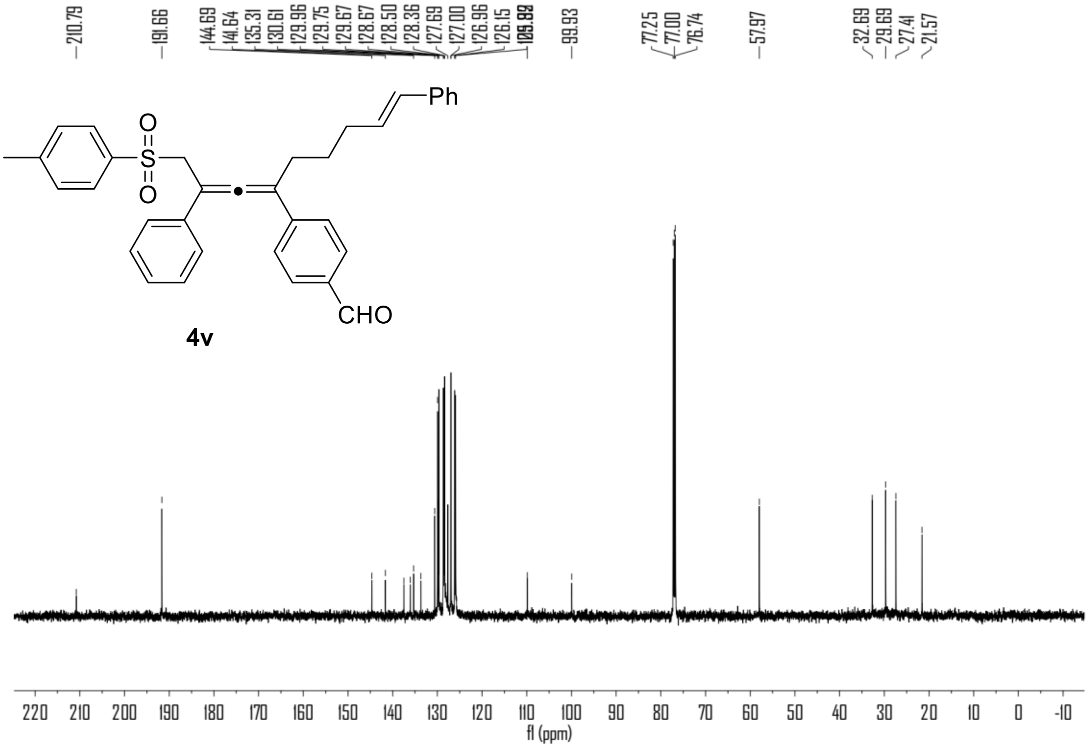
210.79
191.66
144.69
141.64
135.31
130.61
129.96
129.75
129.67
128.67
128.50
128.36
127.69
127.00
126.96
126.15
99.93
77.25
77.00
76.74
57.97
32.69
29.69
27.41
21.57
Ph
CHO
4v
fl (ppm)

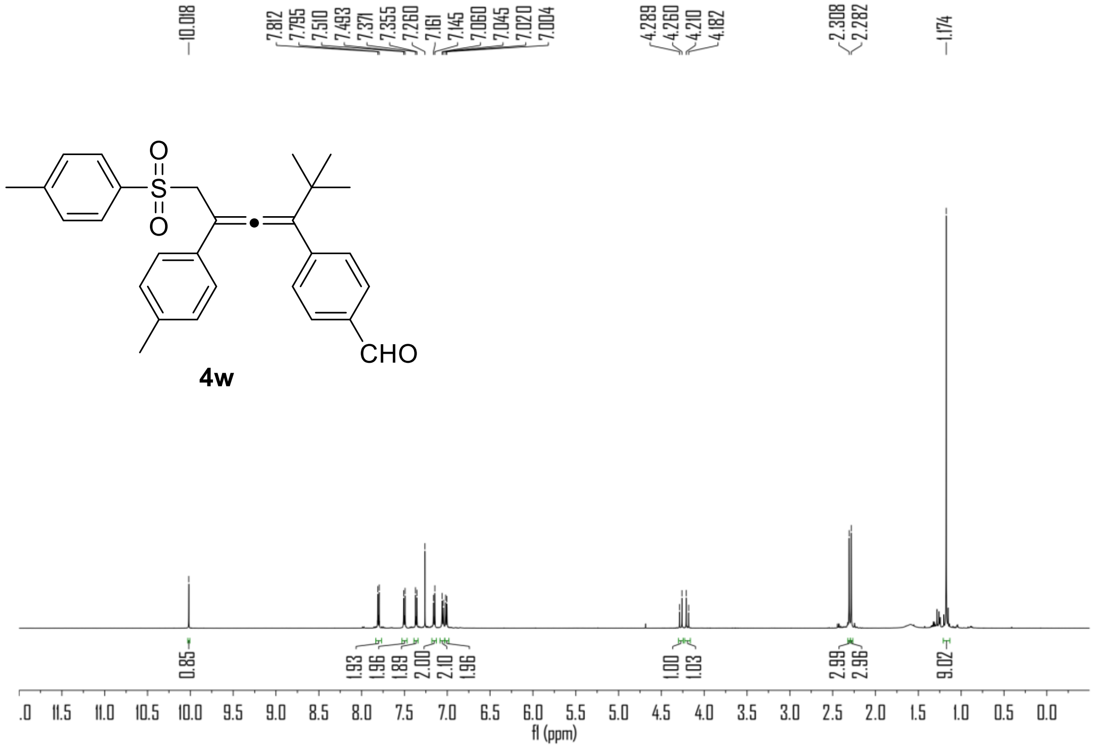
10.018
7.812
7.795
7.510
7.493
7.371
7.355
7.260
7.161
7.145
7.060
7.045
7.020
7.004
4.289
4.260
4.210
4.182
2.308
2.282
1.174
CHO
4w
0.85
1.93
1.96
1.89
2.00
2.10
1.96
1.00
1.03
2.99
2.96
9.02
fl (ppm)

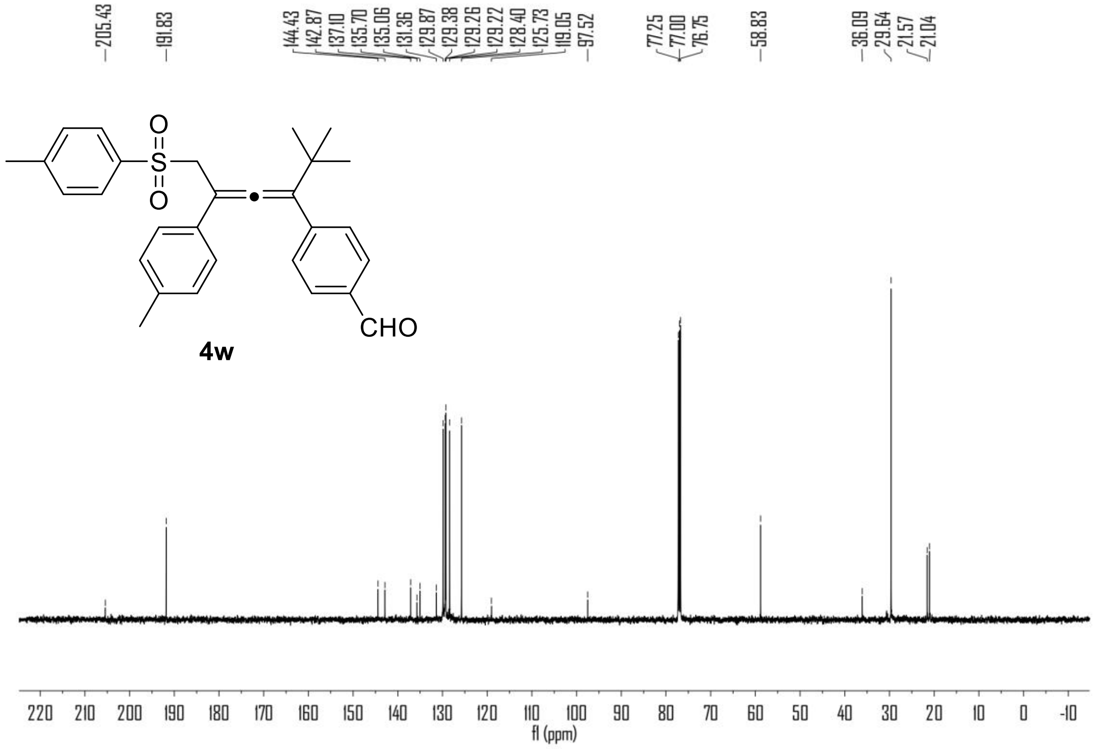
205.43
191.83
144.43
142.87
137.10
135.70
135.06
131.36
129.87
129.38
129.26
129.22
128.40
125.73
119.05
97.52
77.25
77.00
76.75
58.83
36.09
29.64
21.57
21.04
CHO
4w
fl (ppm)

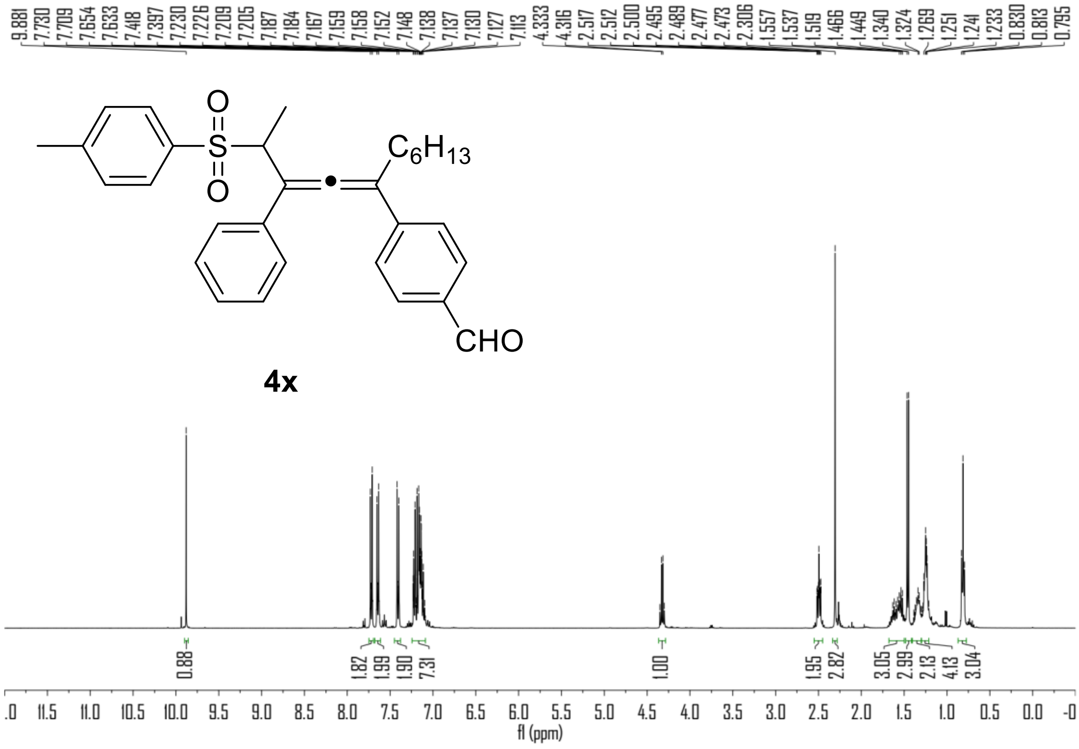
C6H13
CHO
4x
fl (ppm)

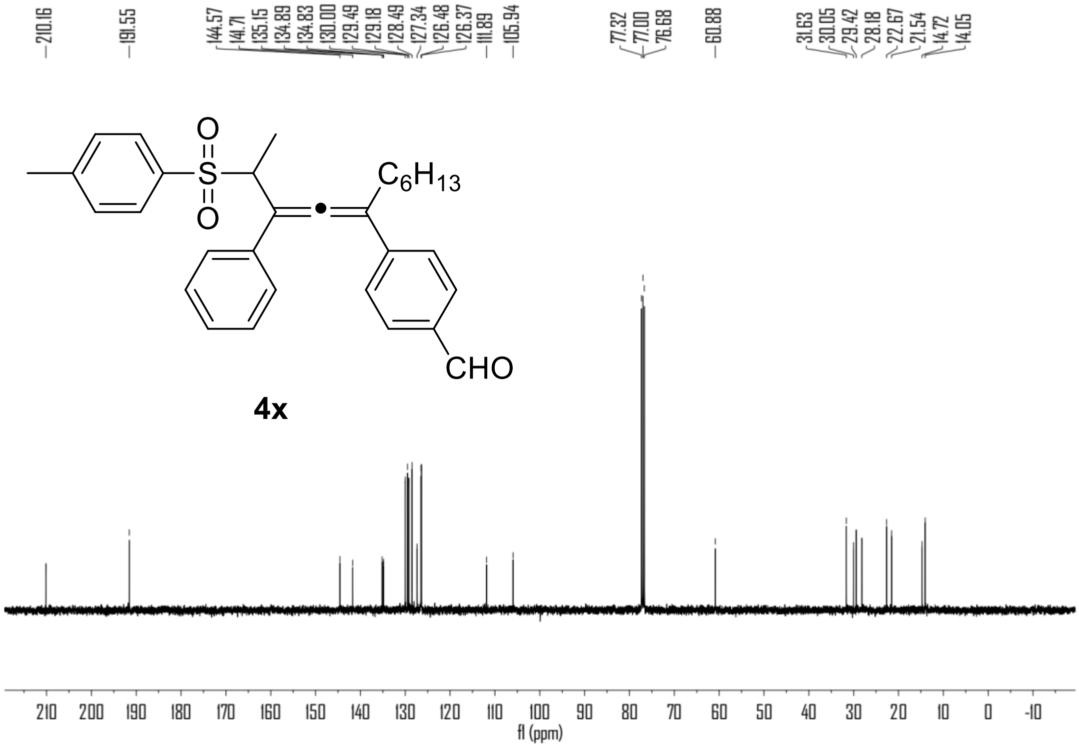
C6H13
CHO
4x
fl (ppm)

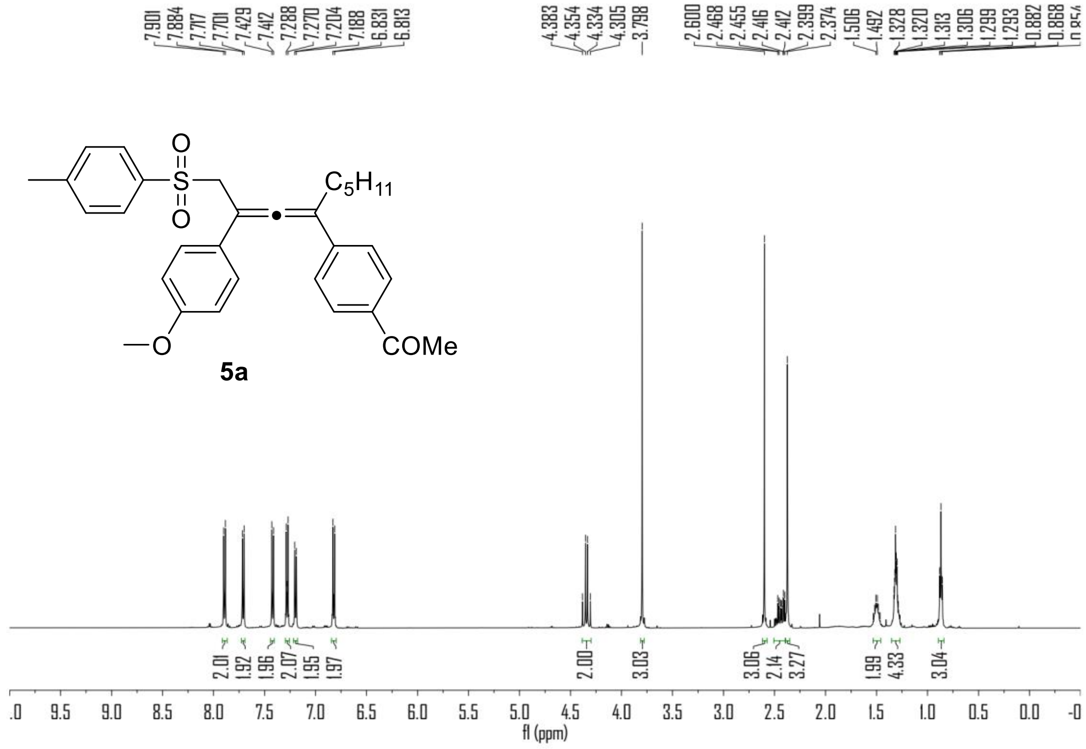

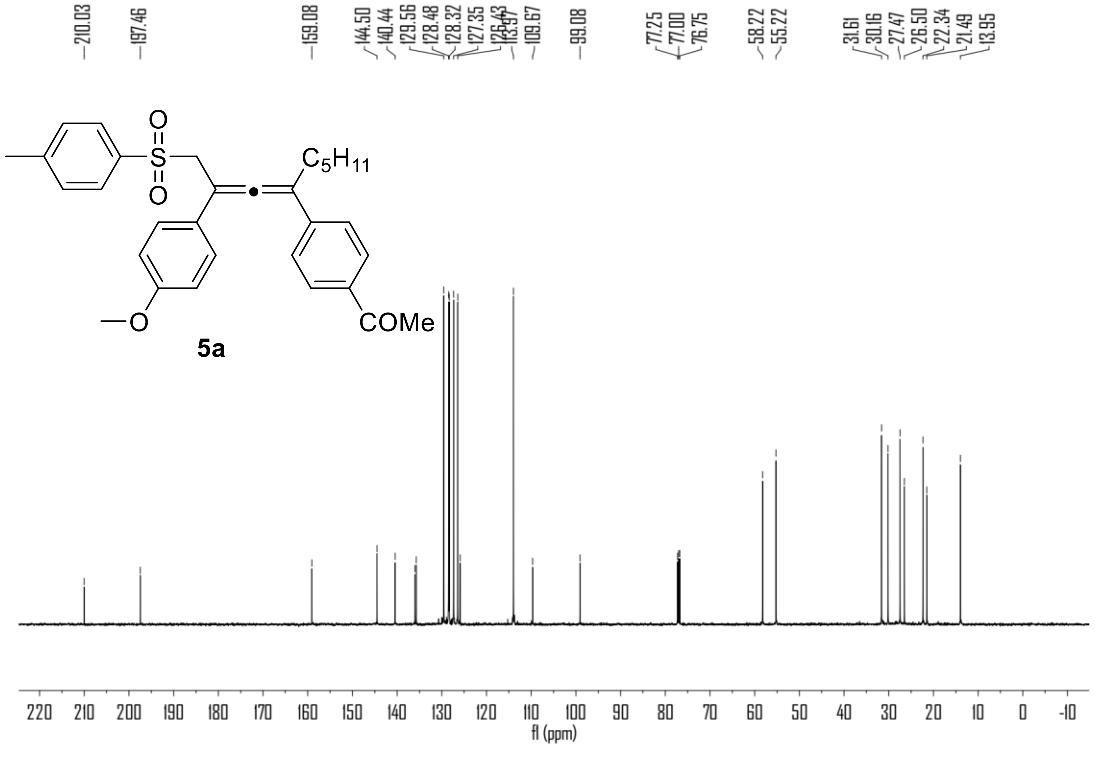

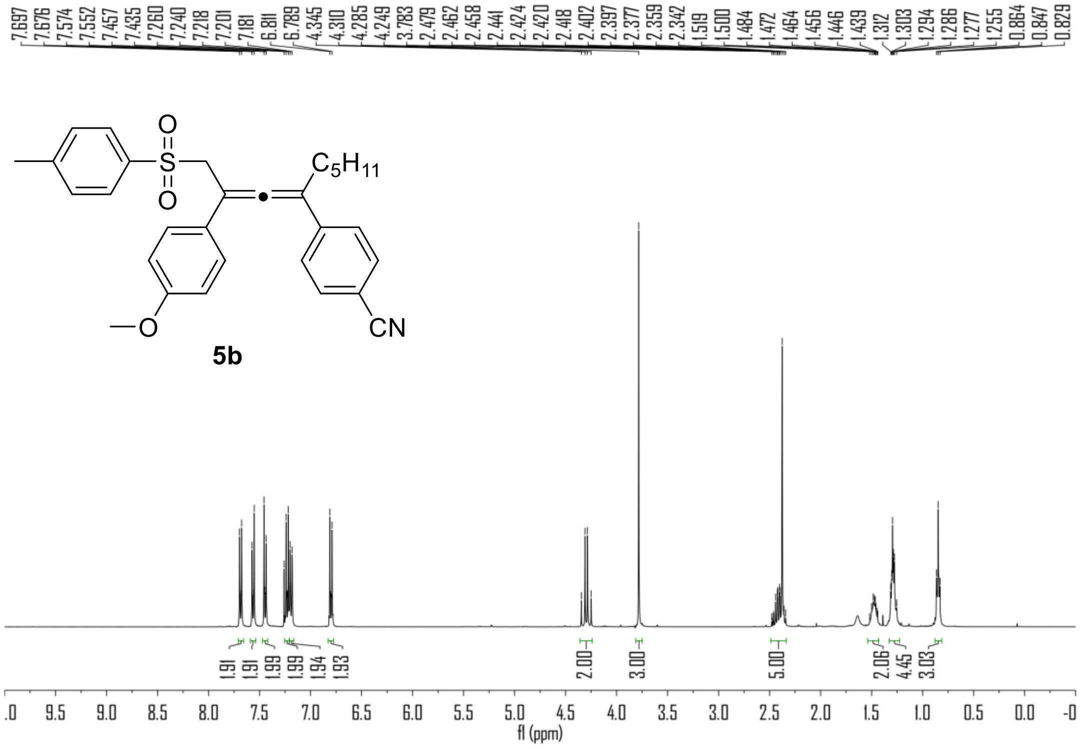
7.697 7.676 7.574 7.552 7.457 7.435 7.260 7.240 7.218 7.201 7.181 6.811 6.789 4.345 4.310 4.285 4.249 3.783 2.479 2.462 2.458 2.441 2.424 2.420 2.418 2.402 2.397 2.377 2.359 2.342 1.519 1.500 1.484 1.472 1.464 1.456 1.446 1.439 1.312 1.303 1.294 1.286 1.277 1.255 0.864 0.847 0.829
O
S
O
C5H11
O
CN
5b
1.91 1.91 1.99 1.99 1.94 1.93 2.00 3.00 5.00 2.06 4.45 3.03
fl (ppm)

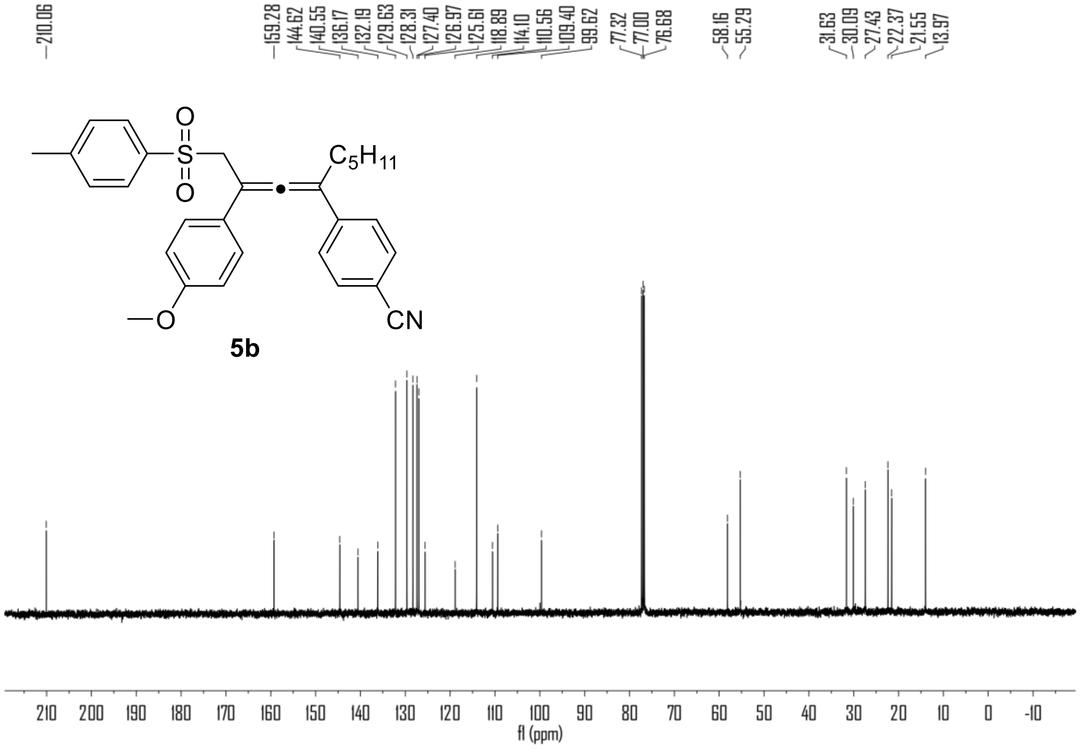
210.06 159.28 144.62 140.55 136.17 132.19 129.63 128.31 127.40 126.97 125.61 118.89 114.10 110.56 109.40 99.62 77.32 77.00 76.68 58.16 55.29 31.63 30.09 27.43 22.37 21.55 13.97
O
S
O
C5H11
O
CN
5b
fl (ppm)

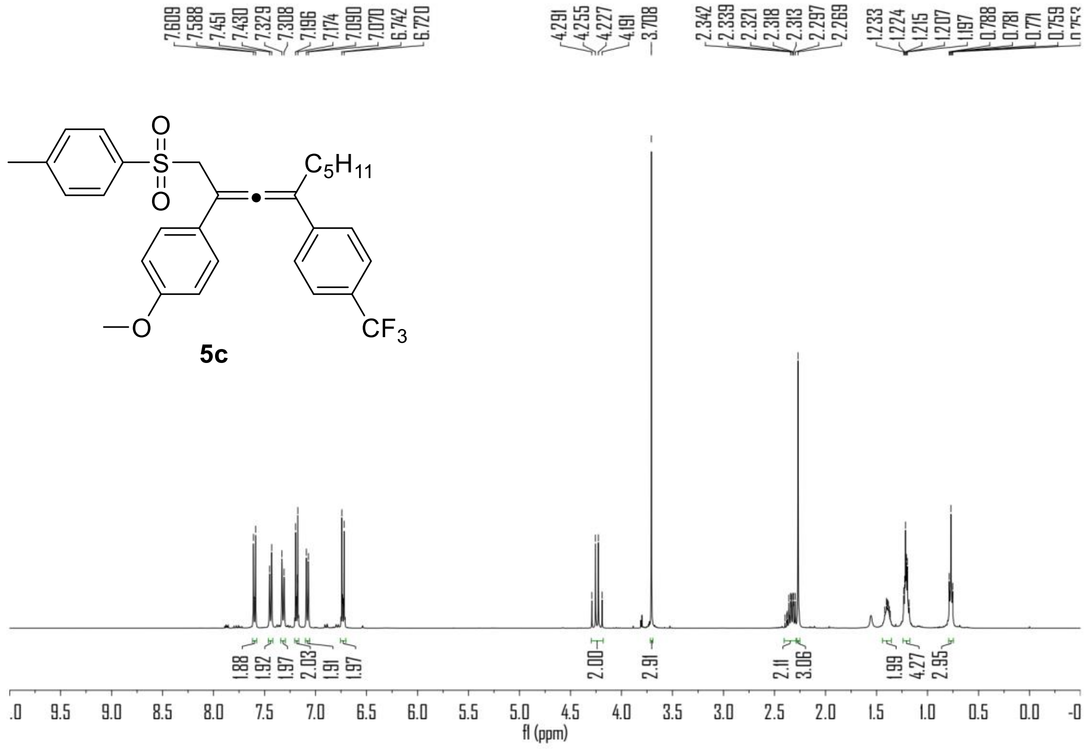
7.609
7.588
7.451
7.430
7.329
7.308
7.196
7.174
7.090
7.070
6.742
6.720
4.291
4.255
4.227
4.191
3.708
2.342
2.339
2.321
2.318
2.313
2.297
2.269
1.233
1.224
1.215
1.207
1.197
0.788
0.781
0.771
0.759
C_5H_{11}
CF_3
5c
1.88
1.92
1.97
2.03
1.91
1.97
2.00
2.91
2.11
3.06
1.99
4.27
2.95
fl (ppm)

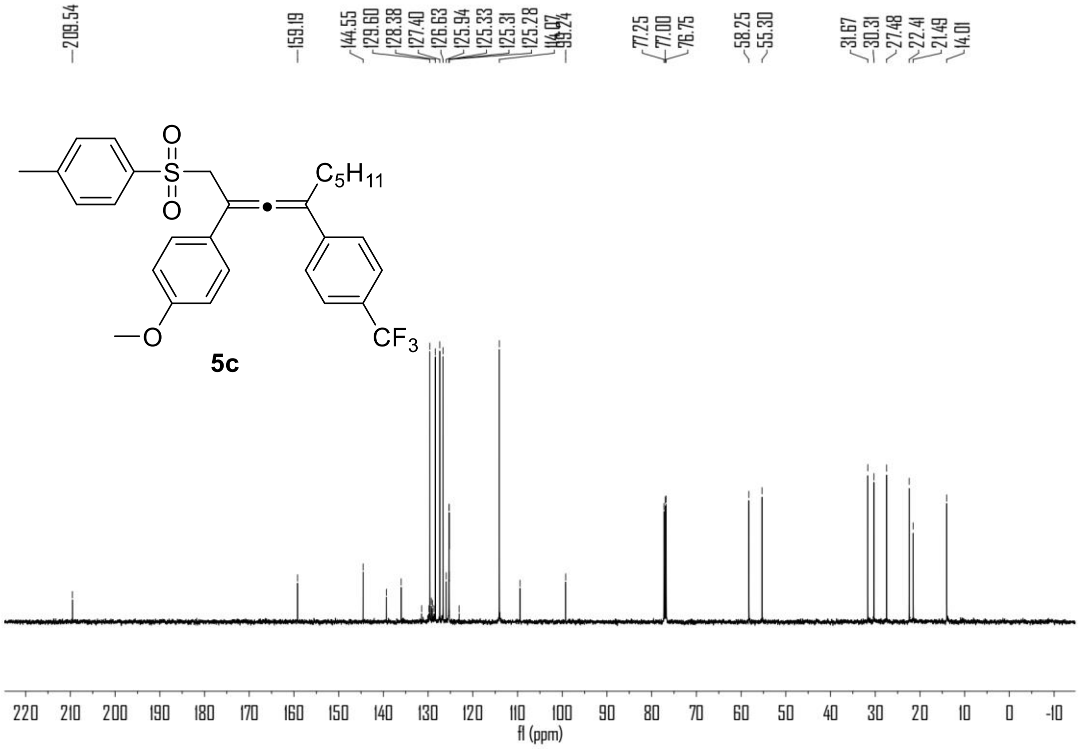
209.54
159.19
144.55
129.60
128.38
127.40
126.63
125.94
125.33
125.31
125.28
113.97
99.24
77.25
77.00
76.75
58.25
55.30
31.67
30.31
27.48
22.41
21.49
14.01
C_5H_{11}
CF_3
5c
fl (ppm)

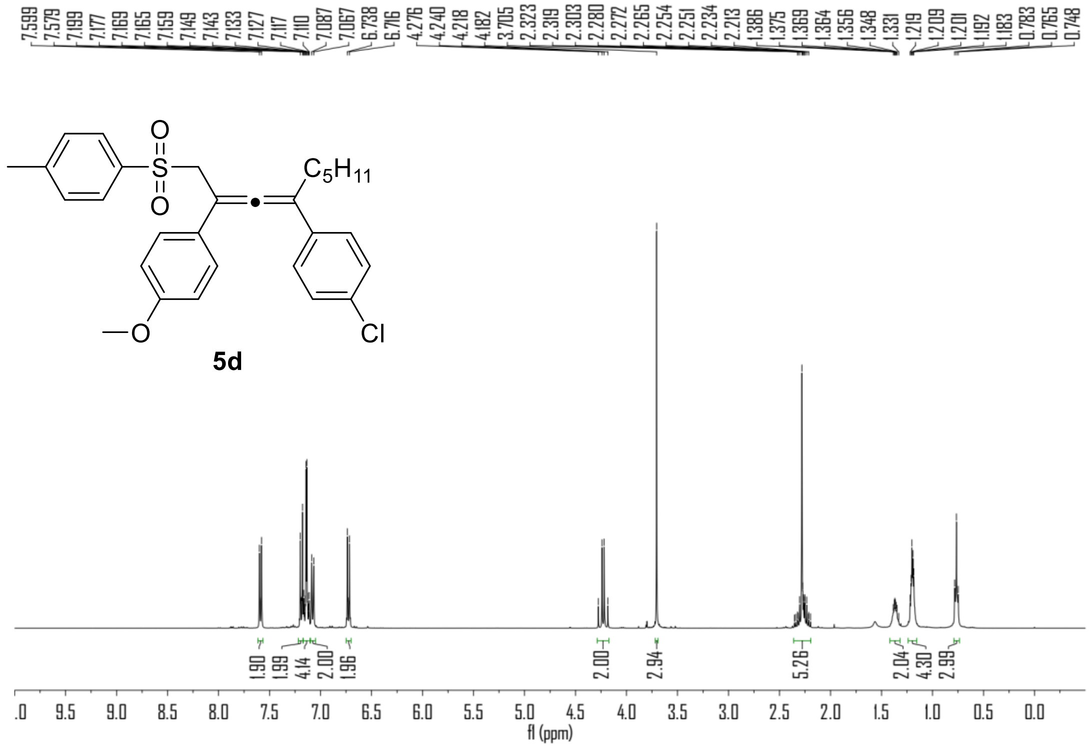

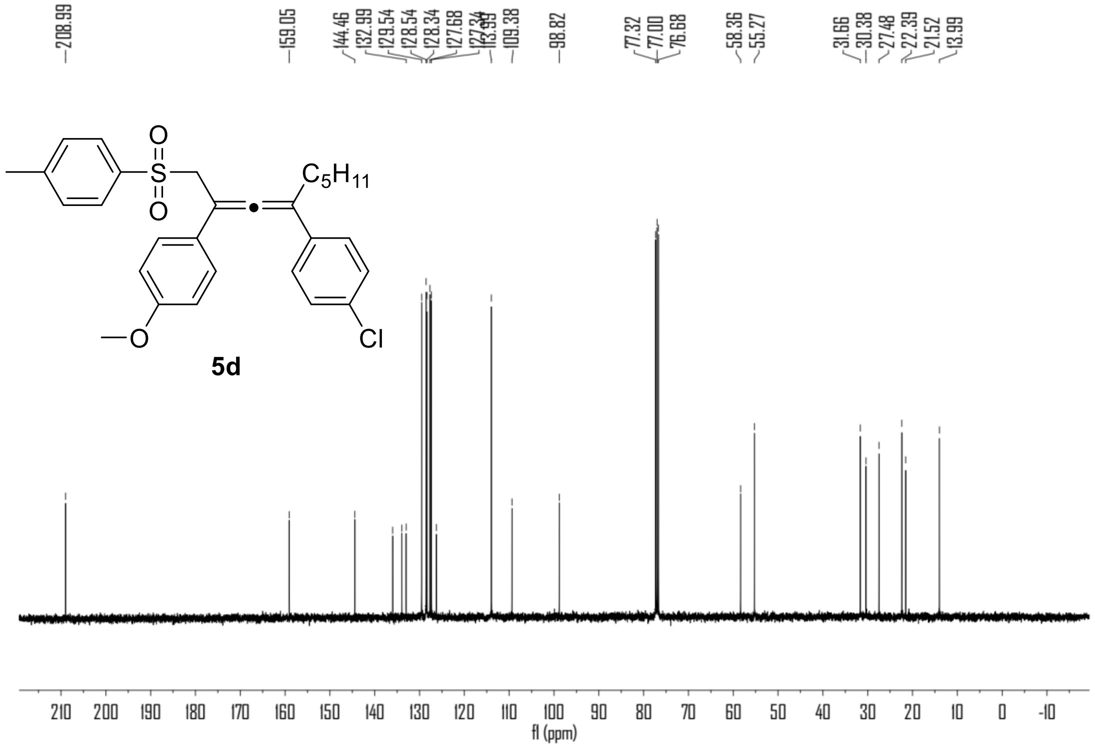

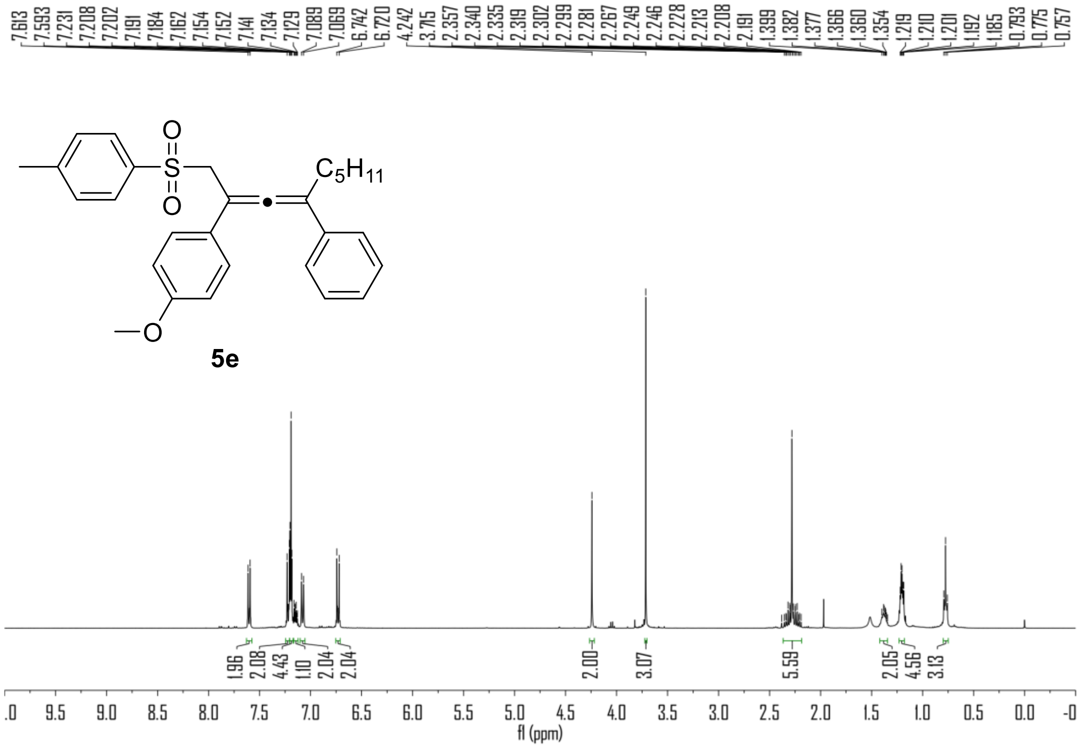
7.613
7.593
7.231
7.208
7.202
7.191
7.184
7.162
7.154
7.152
7.141
7.134
7.129
7.089
7.069
6.742
6.720
4.242
3.715
2.357
2.340
2.335
2.319
2.302
2.299
2.281
2.267
2.249
2.246
2.228
2.213
2.208
2.191
1.399
1.382
1.377
1.366
1.360
1.354
1.219
1.210
1.201
1.192
1.185
0.793
0.775
0.757
C5H11
5e
1.96
2.08
4.43
1.10
2.04
2.04
2.00
3.07
5.59
2.05
4.56
3.13
f1 (ppm)

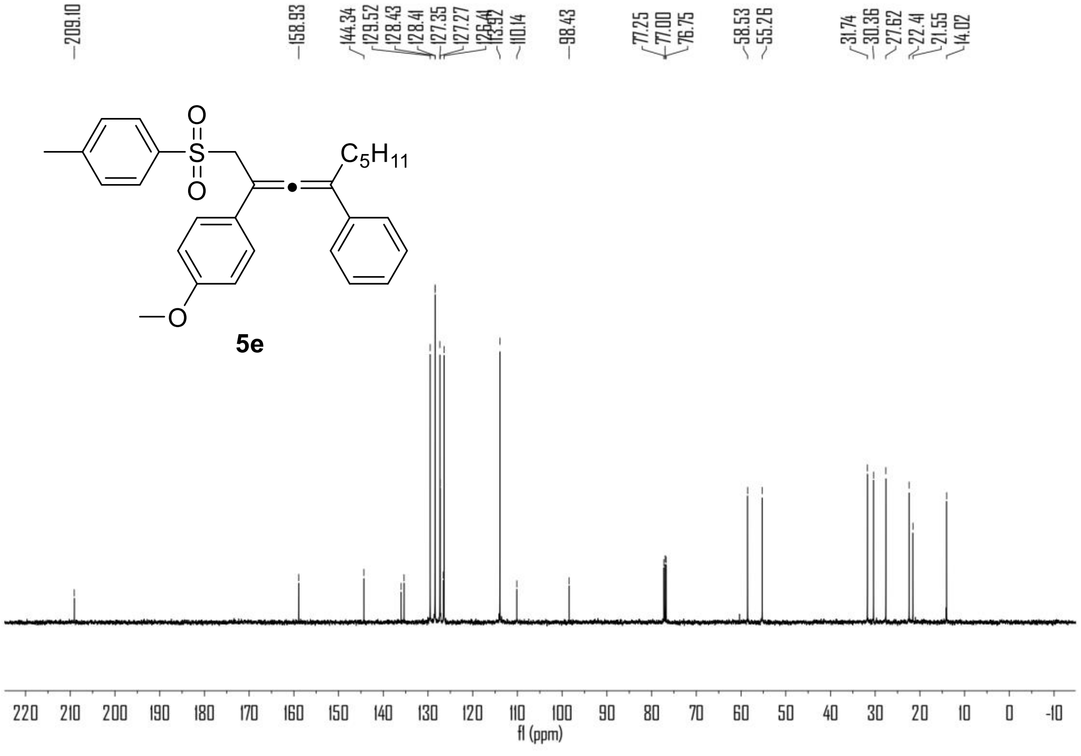
209.10
158.93
144.34
129.52
128.43
128.41
127.35
127.27
126.41
113.92
110.14
98.43
77.25
77.00
76.75
58.53
55.26
31.74
30.36
27.62
22.41
21.55
14.02
C5H11
5e
f1 (ppm)

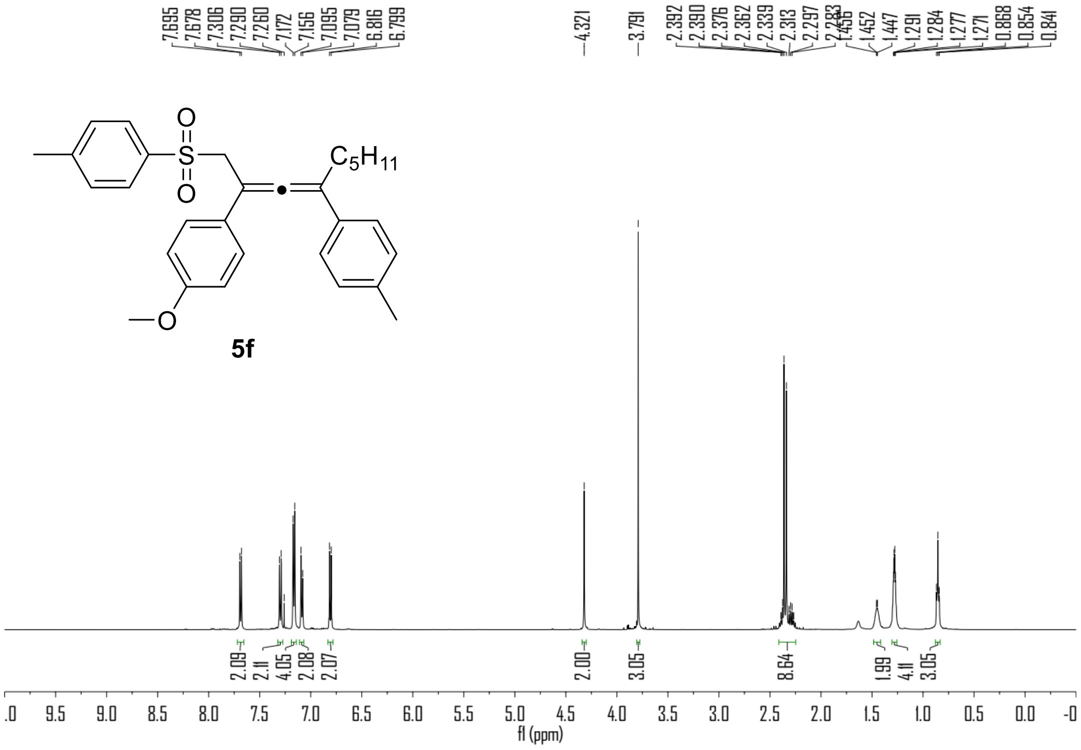
7.695
7.678
7.306
7.290
7.260
7.172
7.156
7.095
7.079
6.816
6.799
4.321
3.791
2.392
2.390
2.376
2.362
2.339
2.313
2.297
1.452
1.447
1.291
1.284
1.277
1.271
0.868
0.854
0.841
C5H11
5f
2.09
2.11
4.05
2.08
2.07
2.00
3.05
8.64
1.99
4.11
3.05
fl (ppm)

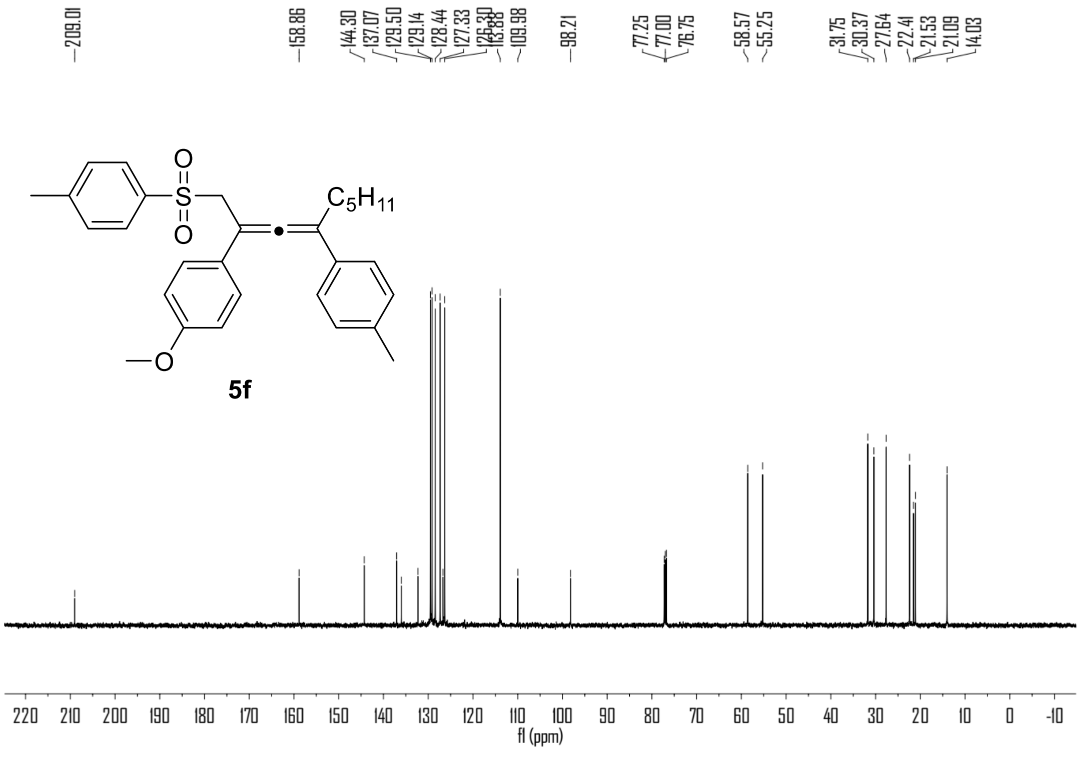
209.01
158.86
144.30
137.07
129.50
129.14
128.44
127.33
109.98
98.21
77.25
77.00
76.75
58.57
55.25
31.75
30.37
27.64
22.41
21.53
21.09
14.03
C5H11
5f
fl (ppm)

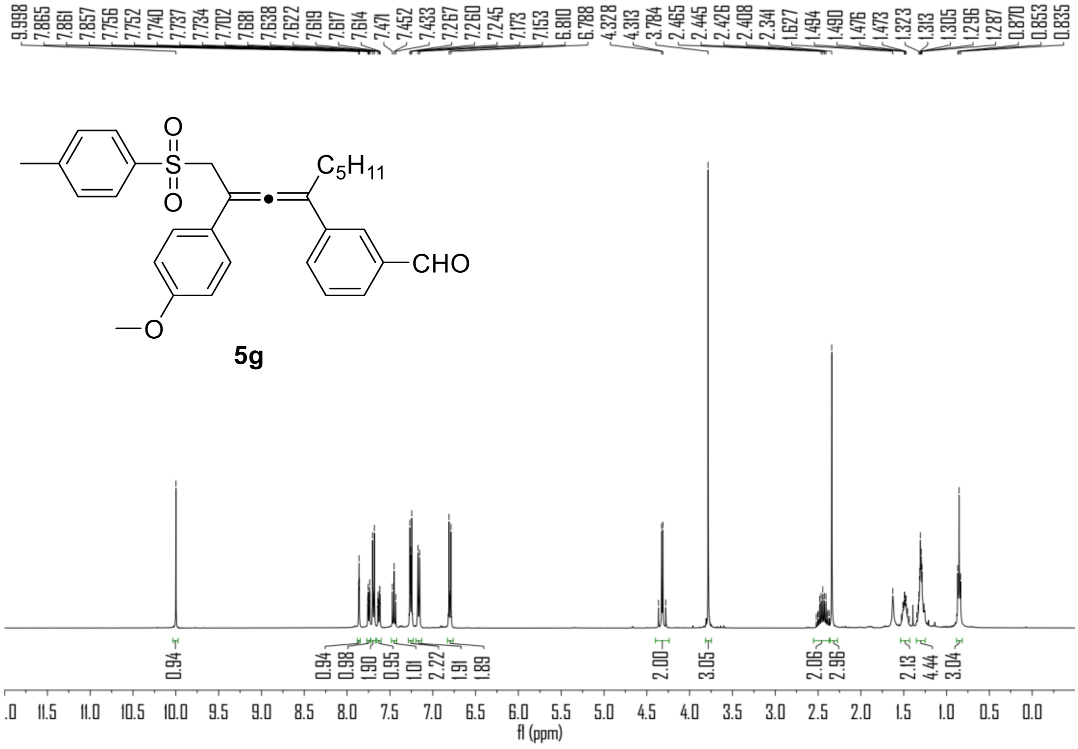

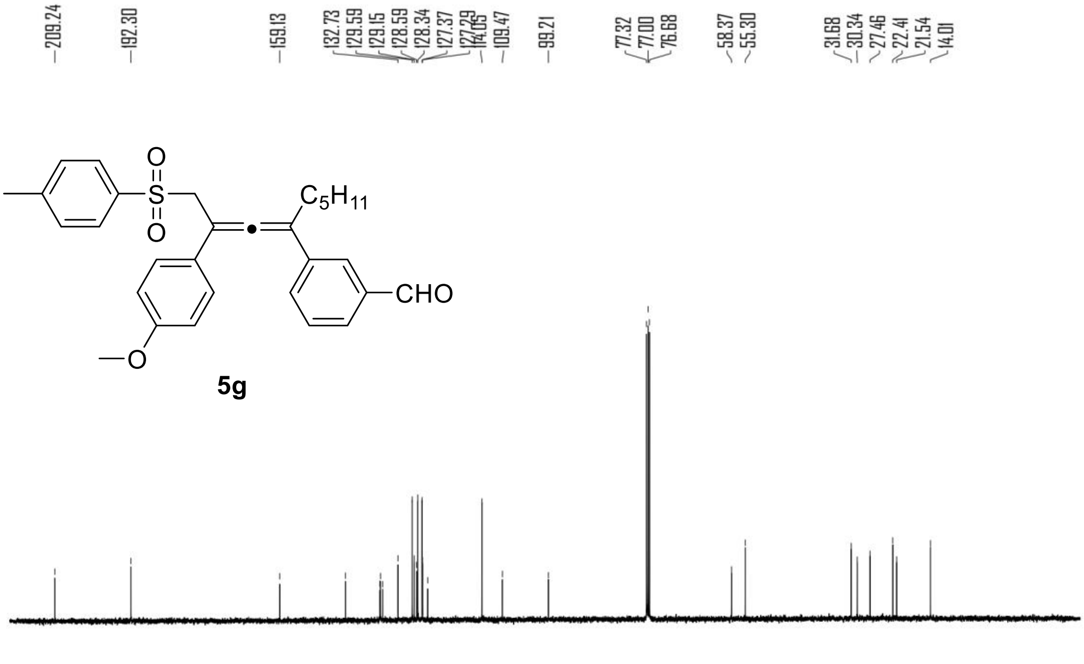

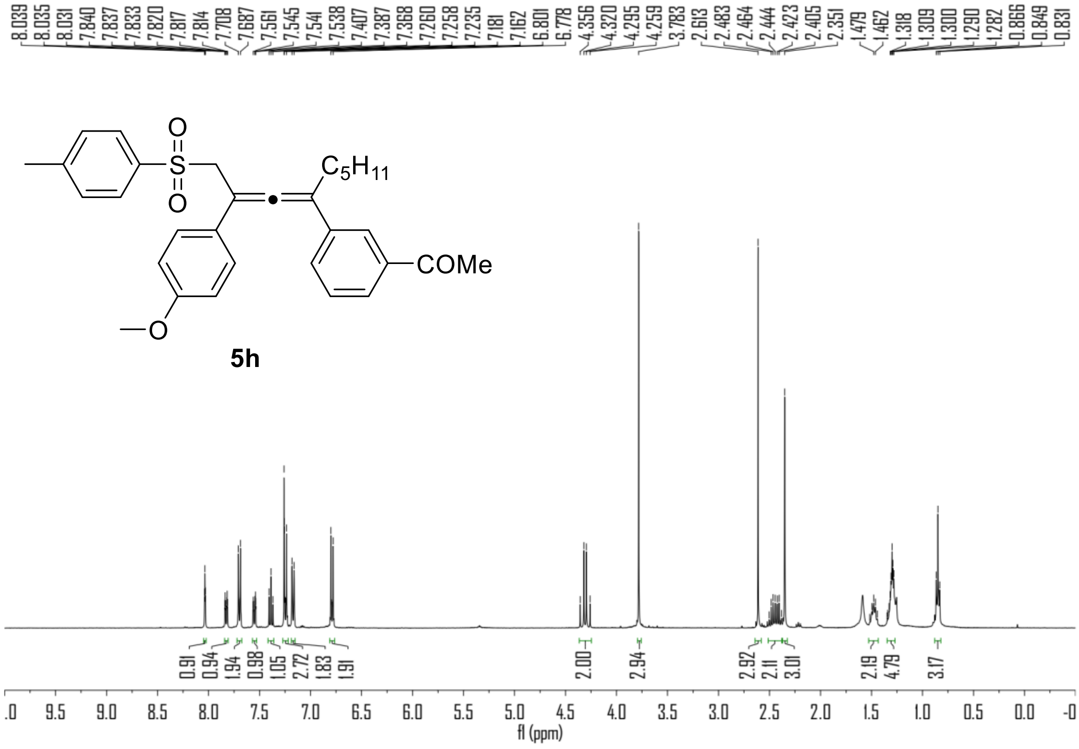
8.039 8.035 8.031 7.840 7.837 7.833 7.820 7.817 7.814 7.708 7.687 7.561 7.545 7.541 7.538 7.407 7.387 7.368 7.260 7.258 7.235 7.181 7.162 6.801 6.778 4.356 4.320 4.295 4.259 3.783 2.613 2.483 2.464 2.444 2.423 2.405 2.351 1.479 1.462 1.318 1.309 1.300 1.290 1.282 0.866 0.849 0.831
C5H11
COMe
5h
0.91 0.94 1.94 0.98 1.05 2.72 1.83 1.91 2.00 2.94 2.92 2.11 3.01 2.19 4.79 3.17
fl (ppm)

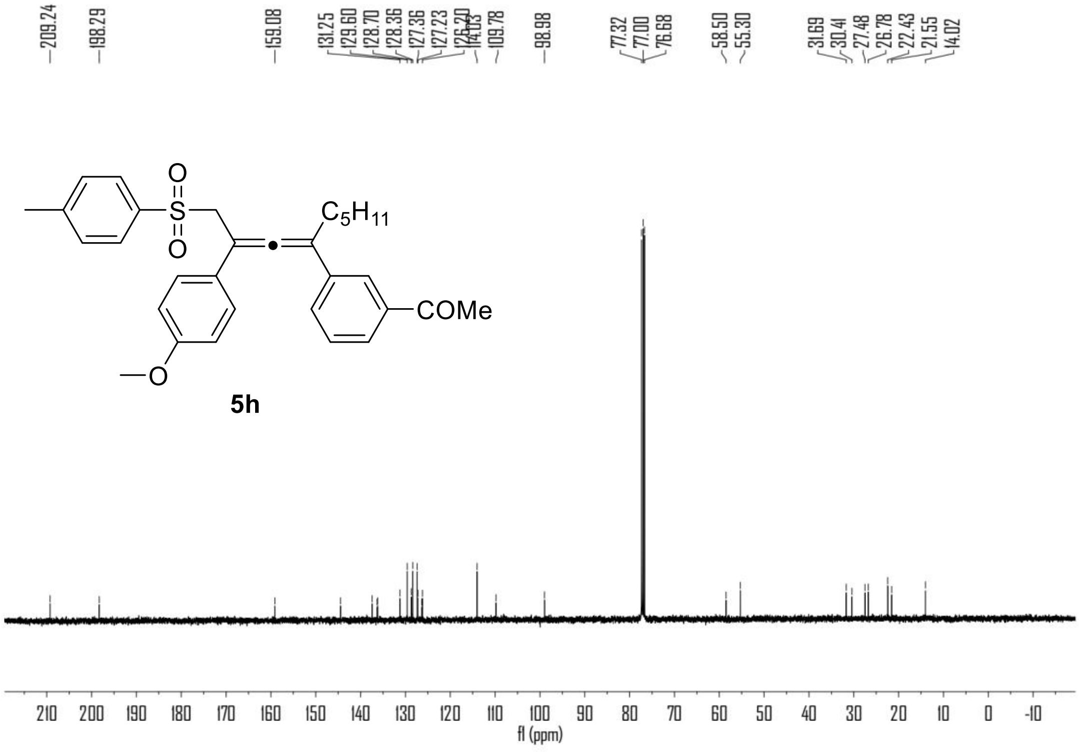
209.24 198.29 159.08 131.25 129.60 128.70 128.36 127.36 127.23 126.20 114.03 109.78 98.98 77.32 77.00 76.68 58.50 55.30 31.69 30.41 27.48 26.78 22.43 21.55 14.02
C5H11
COMe
5h
fl (ppm)

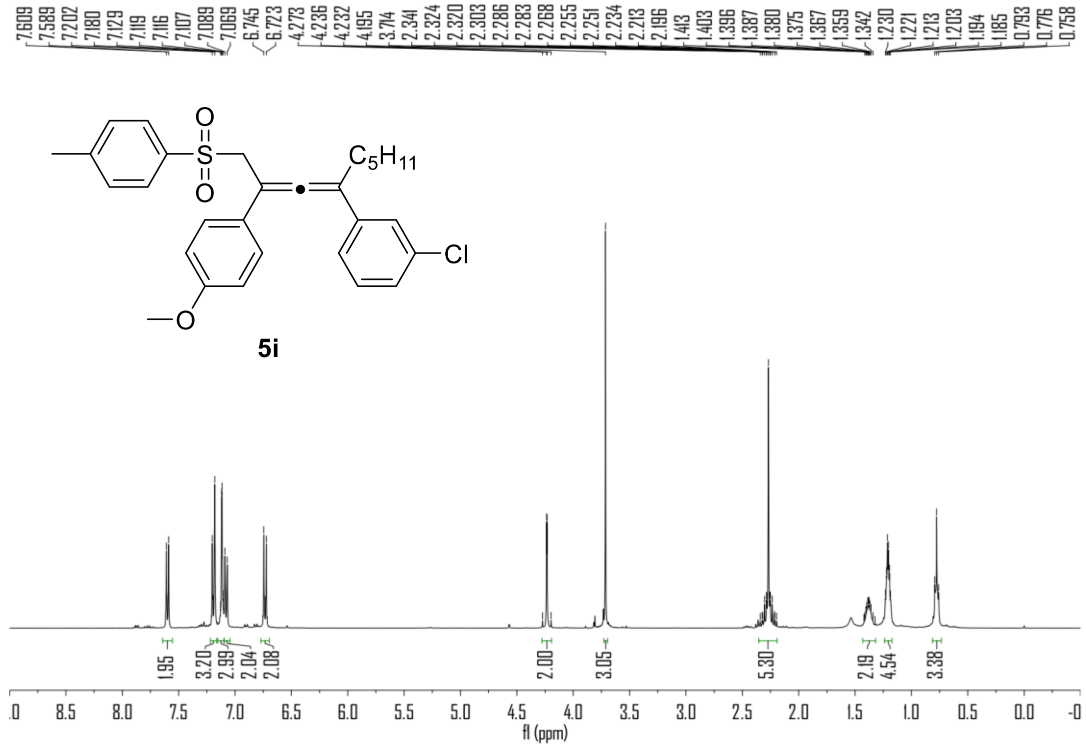
C5H11
Cl
O
S
5i
fl (ppm)

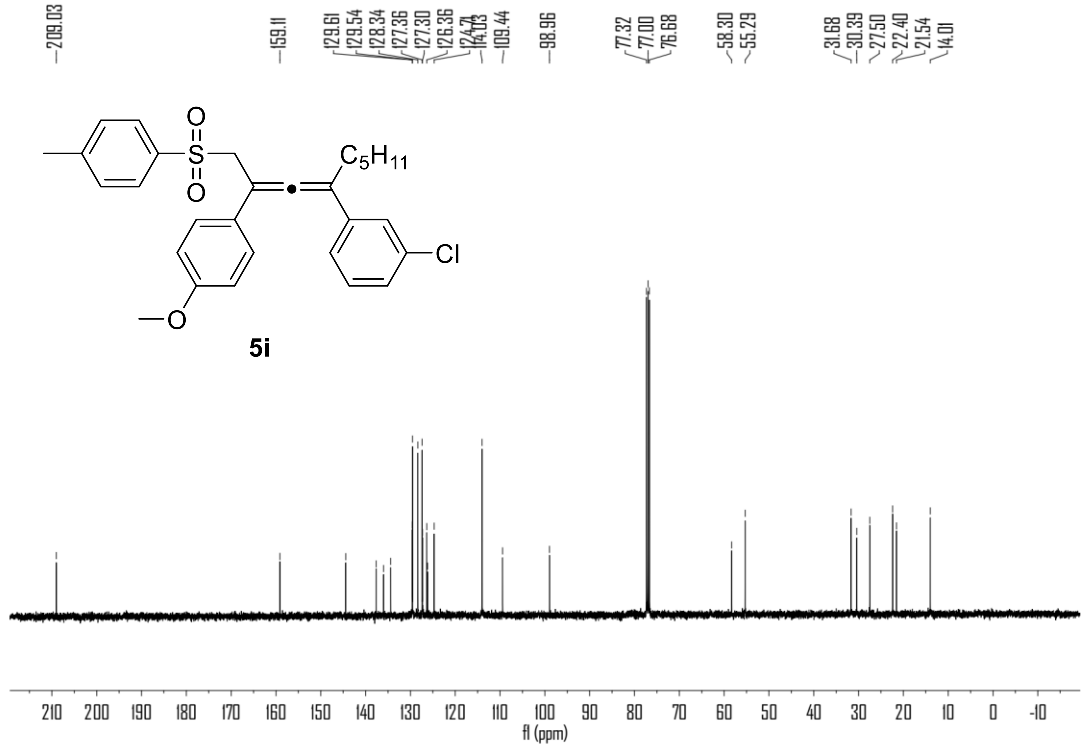
C5H11
Cl
O
S
5i
fl (ppm)

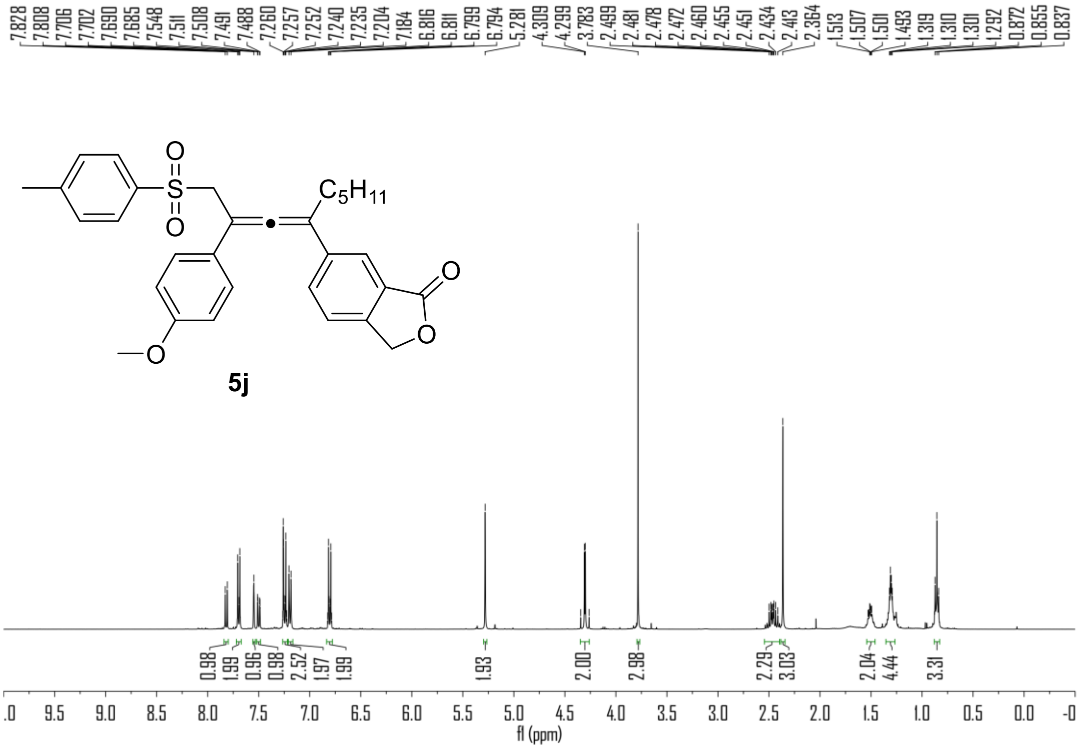
C5H11
5j
f1 (ppm)

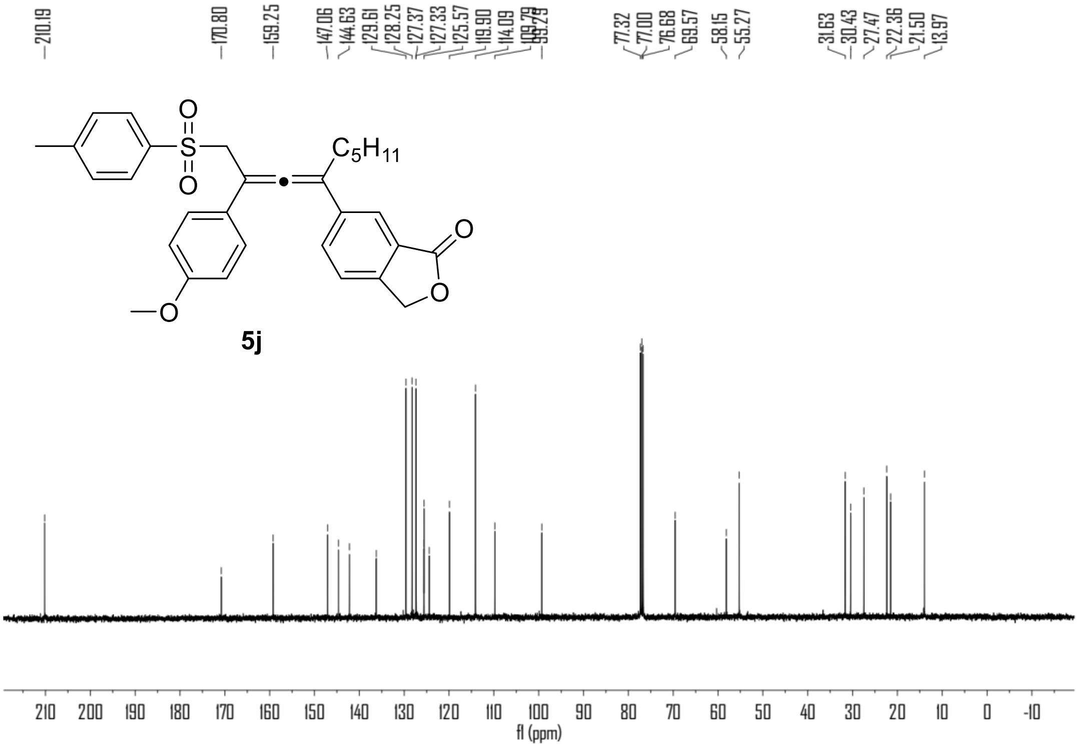
C5H11
5j
f1 (ppm)

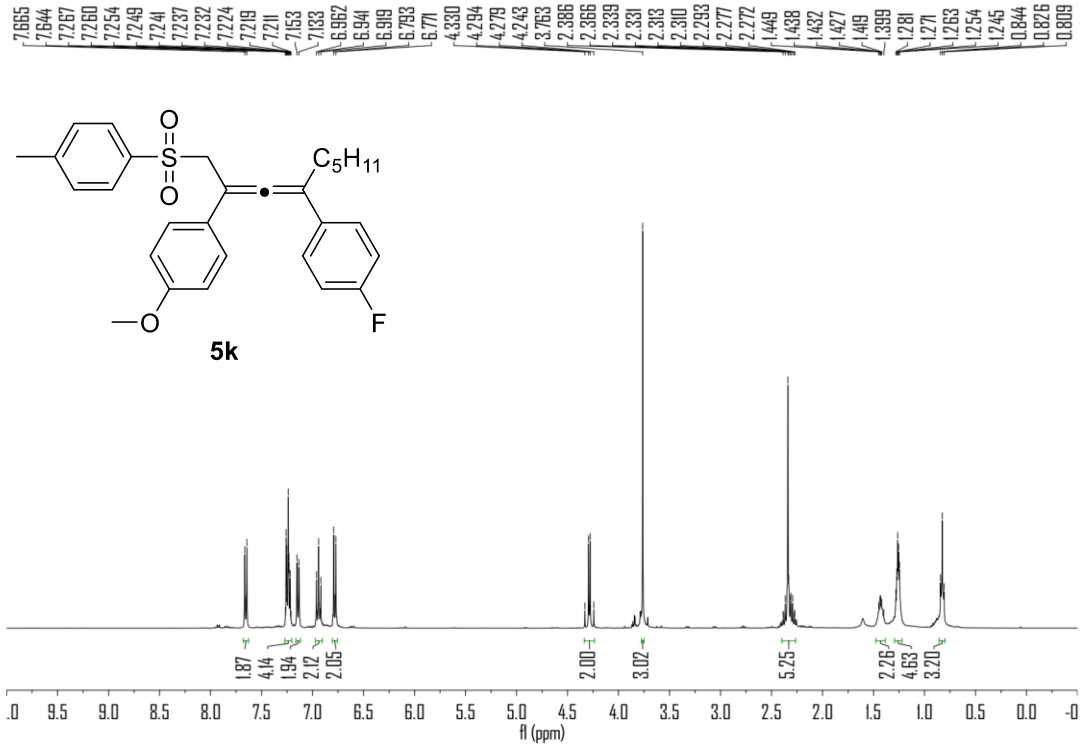
7.665 7.644 7.267 7.260 7.254 7.249 7.241 7.237 7.232 7.224 7.219 7.211 7.153 7.133 6.962 6.941 6.919 6.793 6.771 4.330 4.294 4.279 4.243 3.763 2.386 2.366 2.339 2.331 2.313 2.310 2.293 2.277 2.272 1.449 1.438 1.432 1.427 1.419 1.399 1.281 1.271 1.263 1.254 1.245 0.844 0.826 0.809
O
S
O
C_5H_{11}
O
F
5k
1.87 4.14 1.94 2.12 2.05 2.00 3.02 5.25 2.26 4.63 3.20
.0 9.5 9.0 8.5 8.0 7.5 7.0 6.5 6.0 5.5 5.0 4.5 4.0 3.5 3.0 2.5 2.0 1.5 1.0 0.5 0.0 -0
fl (ppm)

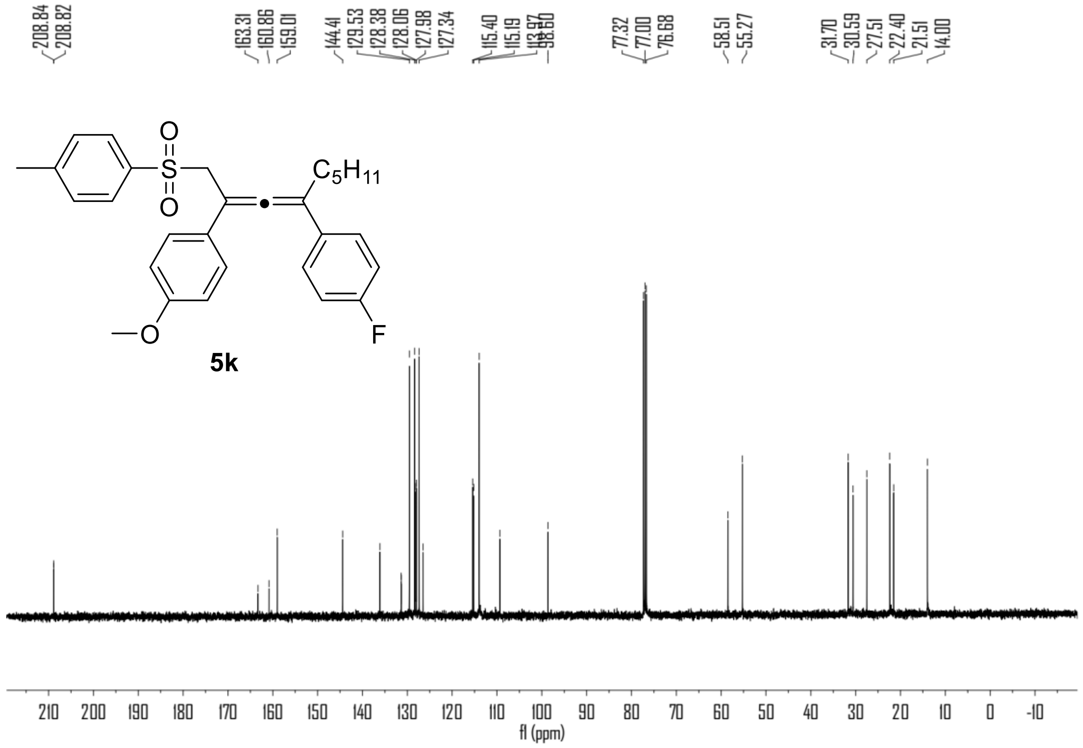
208.84 208.82 163.31 160.86 159.01 144.41 129.53 128.38 128.06 127.98 127.34 115.40 115.19 113.97 98.60 77.32 77.00 76.68 58.51 55.27 31.70 30.59 27.51 22.40 21.51 14.00
O
S
O
C_5H_{11}
O
F
5k
210 200 190 180 170 160 150 140 130 120 110 100 90 80 70 60 50 40 30 20 10 0 -10
fl (ppm)

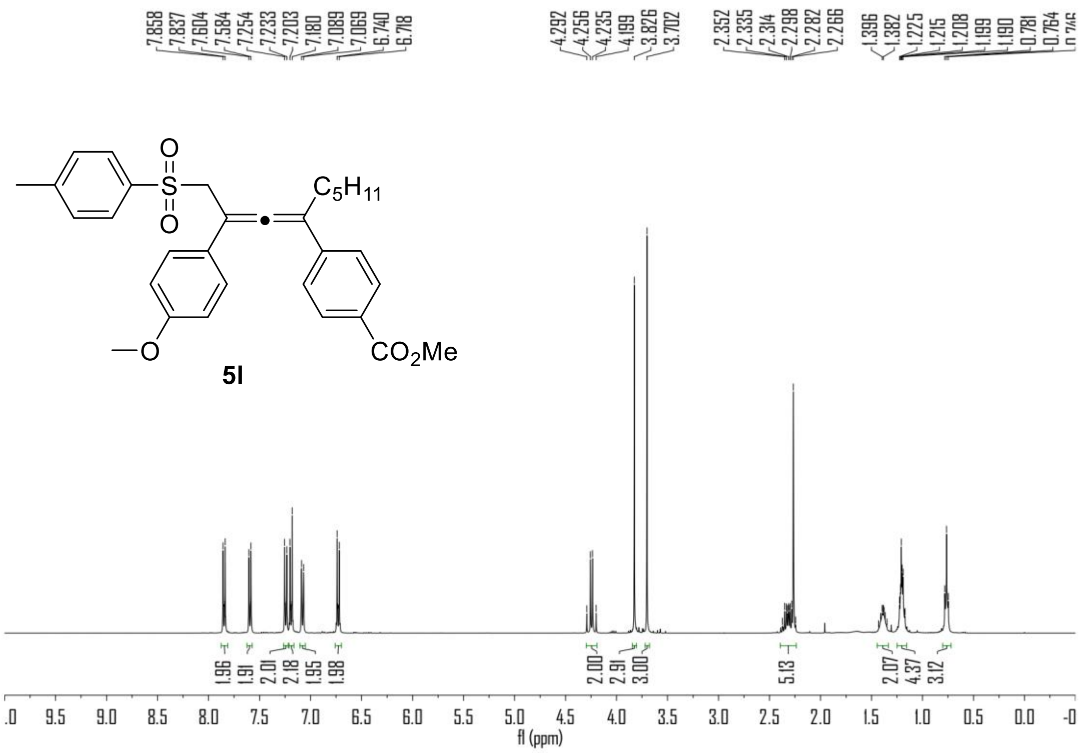

7.858 7.837 7.604 7.584 7.254 7.233 7.203 7.180 7.089 7.069 6.740 6.718
4.292 4.256 4.235 4.199 3.826 3.702
2.352 2.335 2.314 2.298 2.282 2.266
1.396 1.382 1.225 1.215 1.208 1.199 1.190 0.781 0.764
C5H11
CO2Me
5l
1.96 1.91 2.01 2.18 1.95 1.98 2.00 2.91 3.00 5.13 2.07 4.37 3.12
9.5 9.0 8.5 8.0 7.5 7.0 6.5 6.0 5.5 5.0 4.5 4.0 3.5 3.0 2.5 2.0 1.5 1.0 0.5 0.0
fl (ppm)

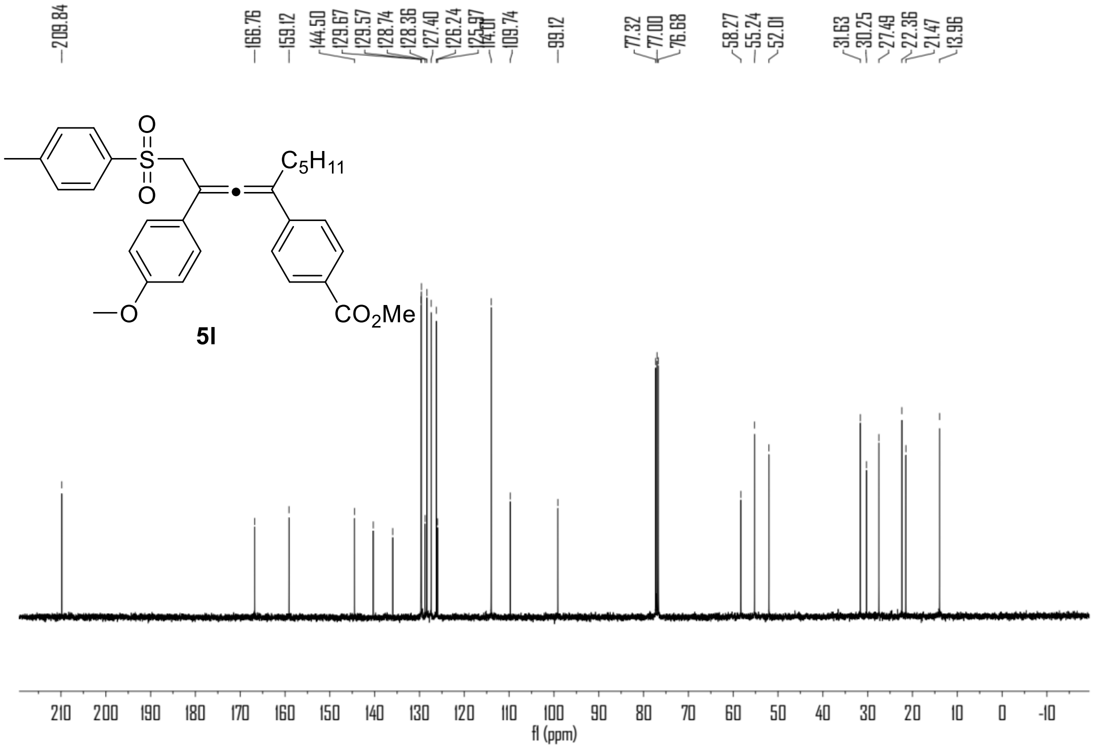

209.84
166.76 159.12 144.50 129.67 129.57 128.74 128.36 127.40 126.24 125.97 114.01 109.74
99.12
77.32 77.00 76.68
58.27 55.24 52.01
31.63 30.25 27.49 22.36 21.47 13.96
C5H11
CO2Me
5l
210 200 190 180 170 160 150 140 130 120 110 100 90 80 70 60 50 40 30 20 10 0 -10
fl (ppm)

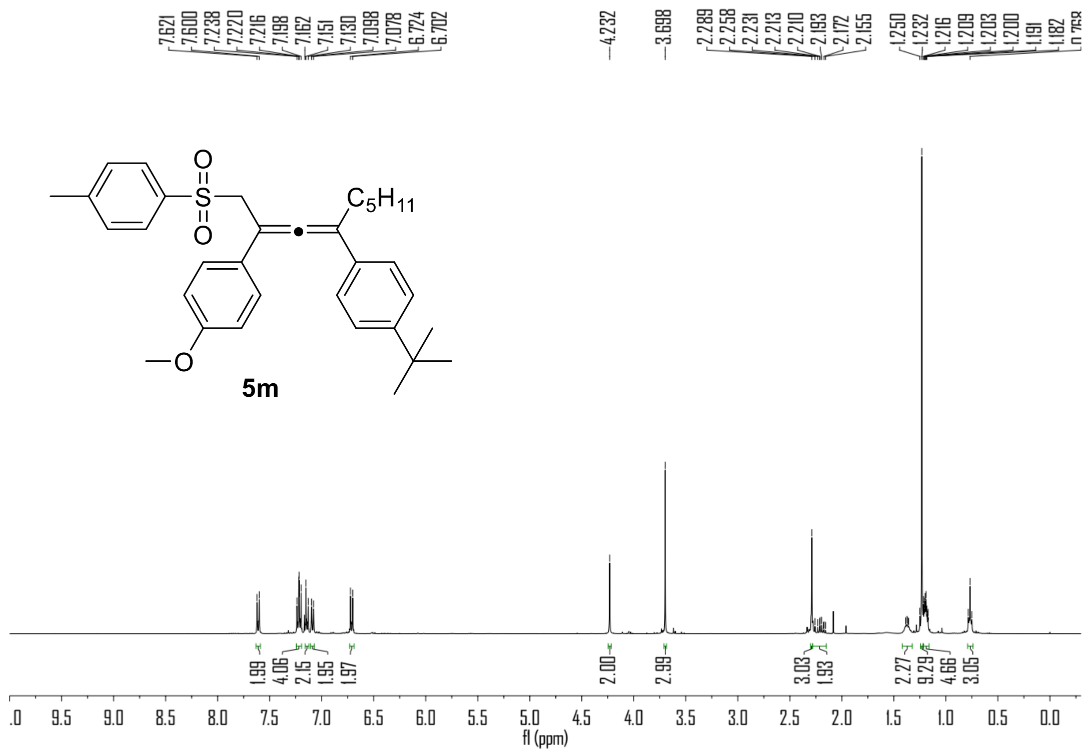
7.621
7.600
7.238
7.220
7.216
7.198
7.162
7.151
7.130
7.098
7.078
6.724
6.702
4.232
3.698
2.289
2.258
2.231
2.213
2.210
2.193
2.172
2.155
1.250
1.232
1.216
1.209
1.203
1.200
1.191
1.182
O
S
O
C5H11
O
5m
1.99
4.06
2.15
1.95
1.97
2.00
2.99
3.03
1.93
2.27
9.29
4.66
3.05
.0 9.5 9.0 8.5 8.0 7.5 7.0 6.5 6.0 5.5 5.0 4.5 4.0 3.5 3.0 2.5 2.0 1.5 1.0 0.5 0.0
f1 (ppm)

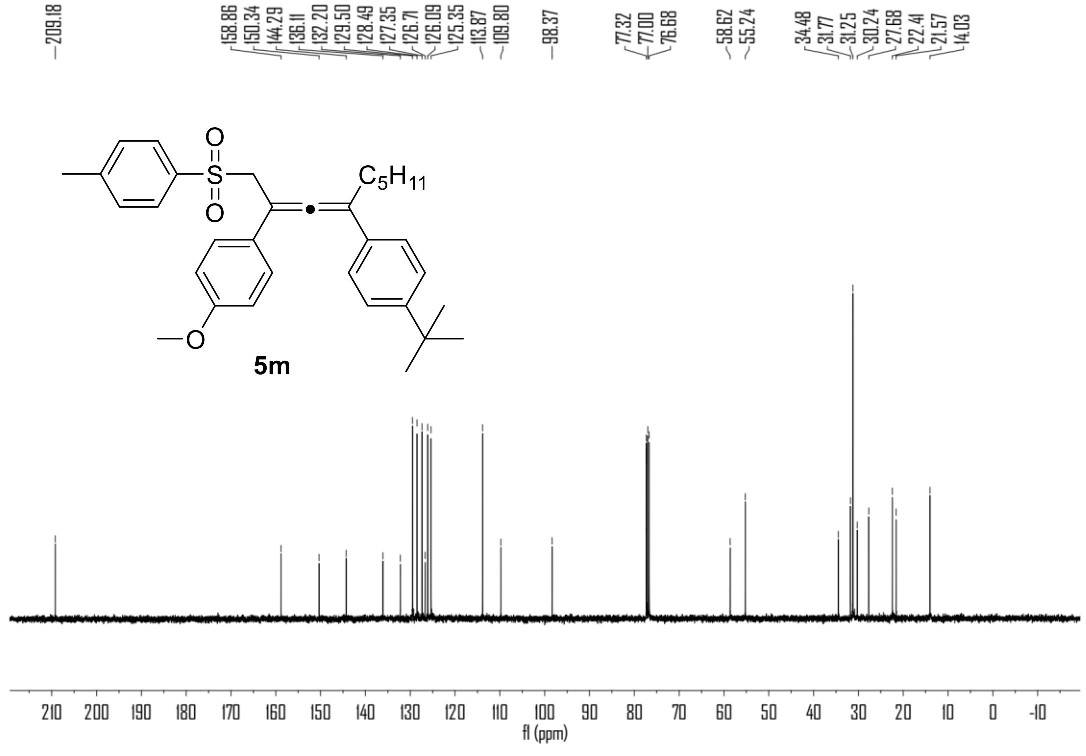
209.18
158.86
150.34
144.29
136.11
132.20
129.50
128.49
127.35
126.71
126.09
125.35
113.87
109.80
98.37
77.32
77.00
76.68
58.62
55.24
34.48
31.77
31.25
30.24
27.68
22.41
21.57
14.03
O
S
O
C5H11
O
5m
210 200 190 180 170 160 150 140 130 120 110 100 90 80 70 60 50 40 30 20 10 0 -10
f1 (ppm)

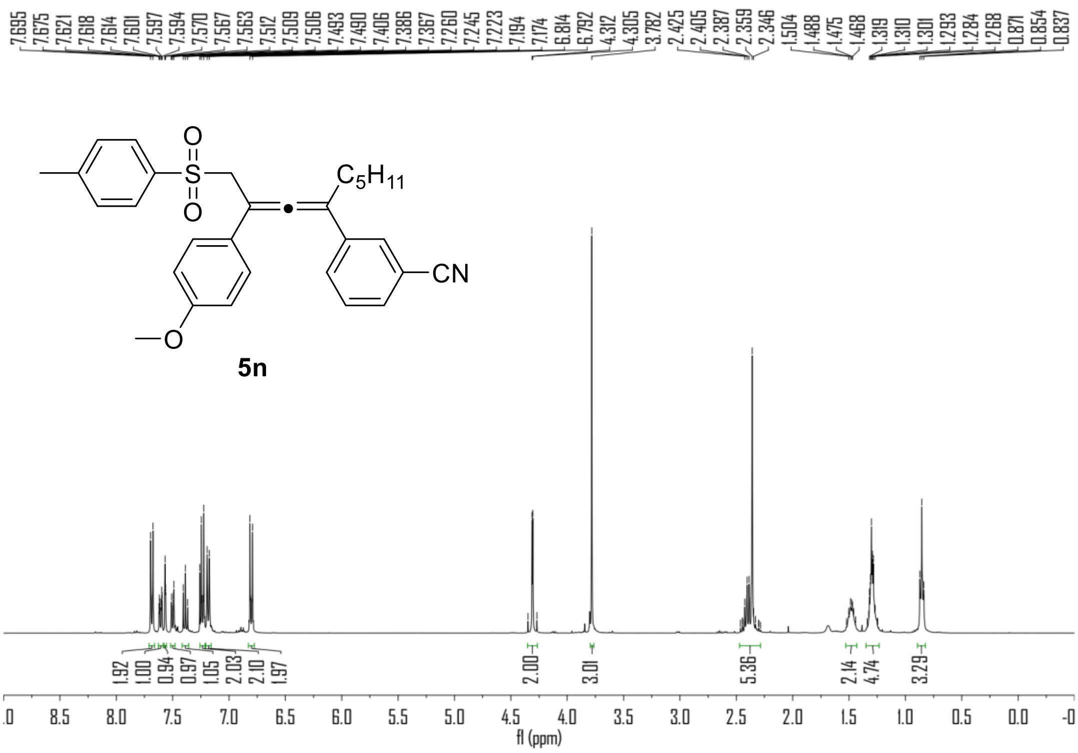
C5H11
CN
5n
fl (ppm)

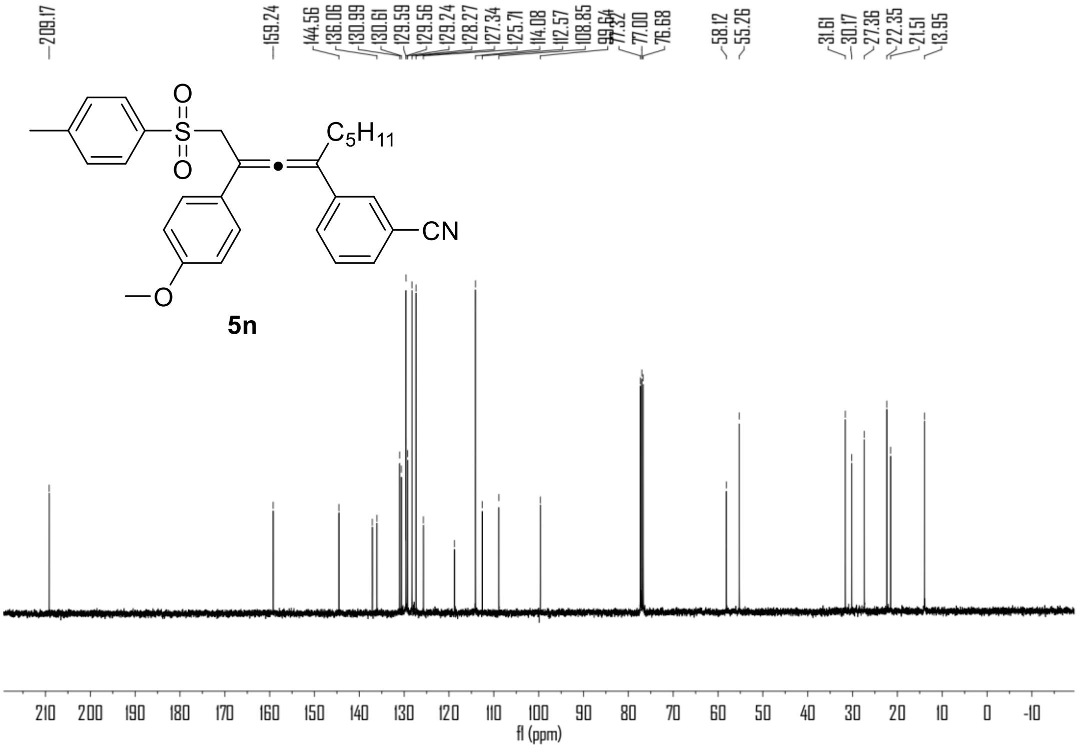
C5H11
CN
5n
fl (ppm)

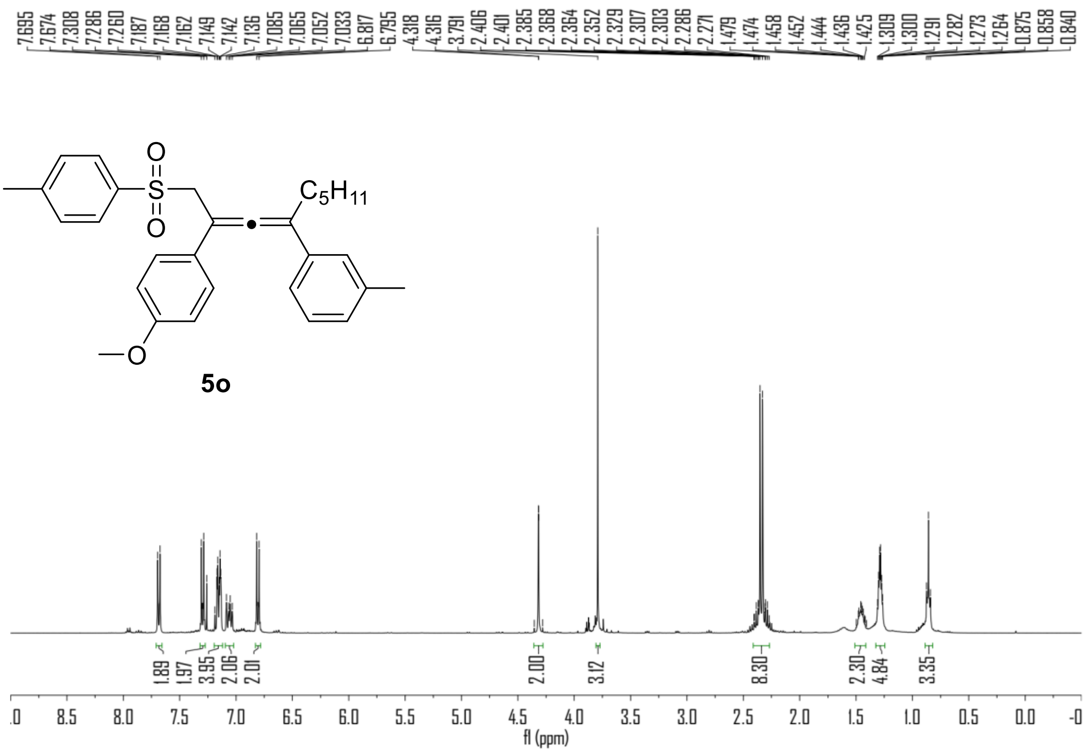
7.695 7.674 7.308 7.286 7.260 7.187 7.168 7.162 7.149 7.142 7.136 7.085 7.065 7.052 7.033 6.817 6.795 4.318 4.316 3.791 2.406 2.401 2.385 2.368 2.364 2.352 2.329 2.307 2.303 2.286 2.271 1.479 1.474 1.458 1.452 1.444 1.436 1.425 1.309 1.300 1.291 1.282 1.273 1.264 0.875 0.858 0.840
C5H11
5o
1.89 1.97 3.95 2.06 2.01 2.00 3.12 8.30 2.30 4.84 3.35
f1 (ppm)

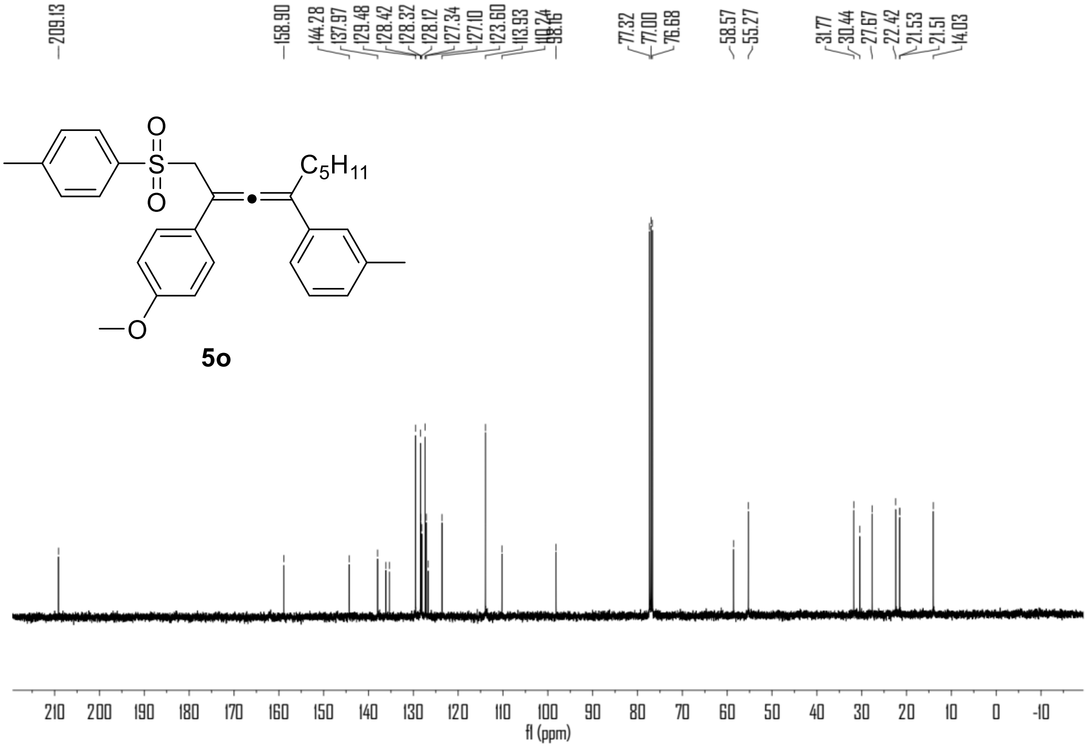
209.13 158.90 144.28 137.97 129.48 128.42 128.32 128.12 127.34 127.10 123.60 113.93 110.24 98.16 77.32 77.00 76.68 58.57 55.27 31.77 30.44 27.67 22.42 21.53 21.51 14.03
C5H11
5o
f1 (ppm)

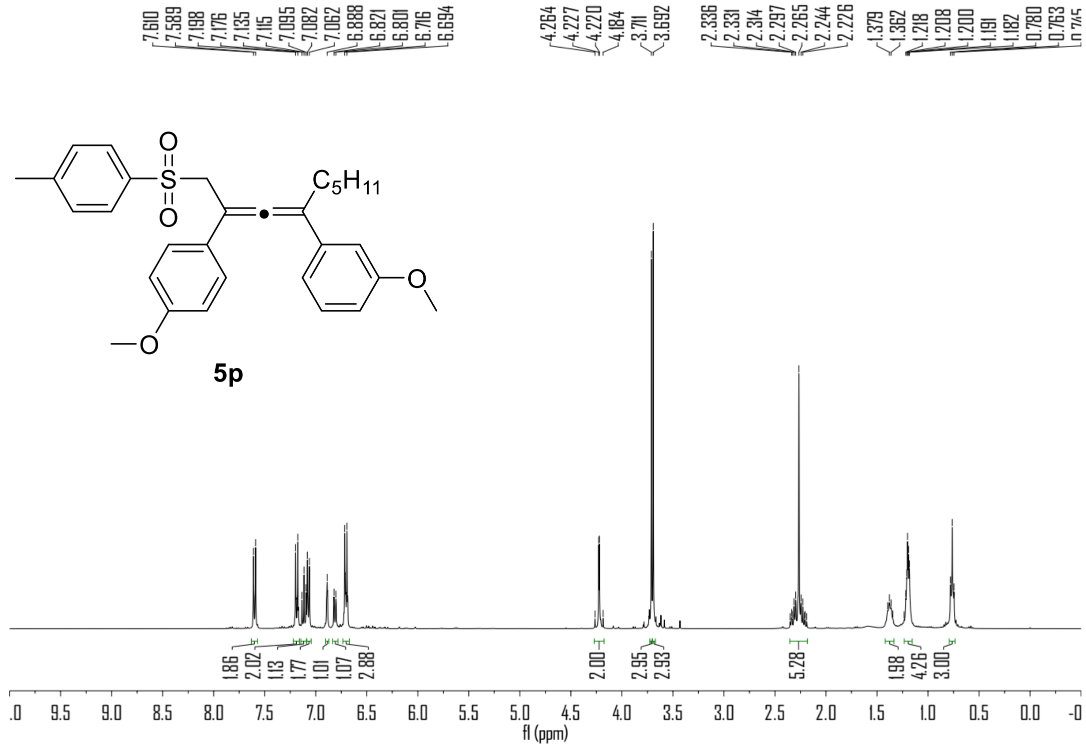
7.610
7.589
7.198
7.176
7.135
7.115
7.095
7.082
7.062
6.888
6.821
6.801
6.716
6.694
4.264
4.227
4.220
4.184
3.711
3.692
2.336
2.331
2.314
2.297
2.265
2.244
2.226
1.379
1.362
1.218
1.208
1.200
1.191
1.182
0.780
0.763
O
S
O
C5H11
O
O
5p
1.86
2.02
1.13
1.77
1.01
1.07
2.88
2.00
2.95
2.93
5.28
1.98
4.26
3.00
fl (ppm)

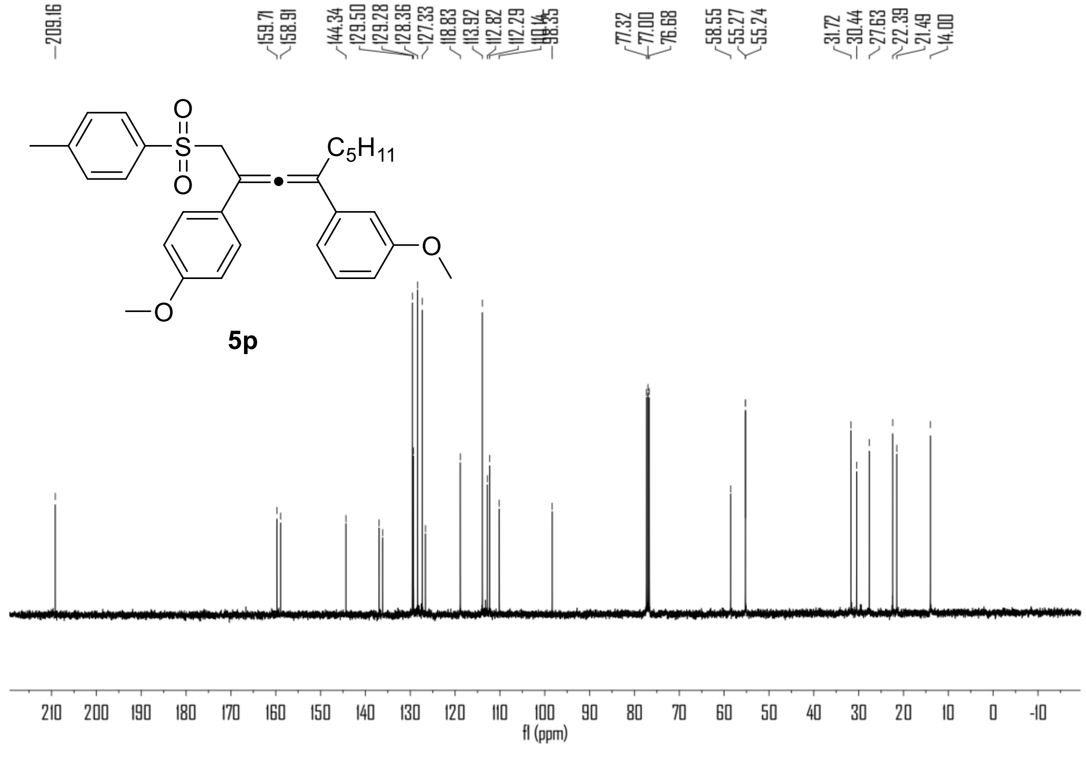
209.16
159.71
158.91
144.34
129.50
129.28
128.36
127.33
118.83
113.92
112.82
112.29
98.35
77.32
77.00
76.68
58.55
55.27
55.24
31.72
30.44
27.63
22.39
21.49
14.00
O
S
O
C5H11
O
O
5p
fl (ppm)

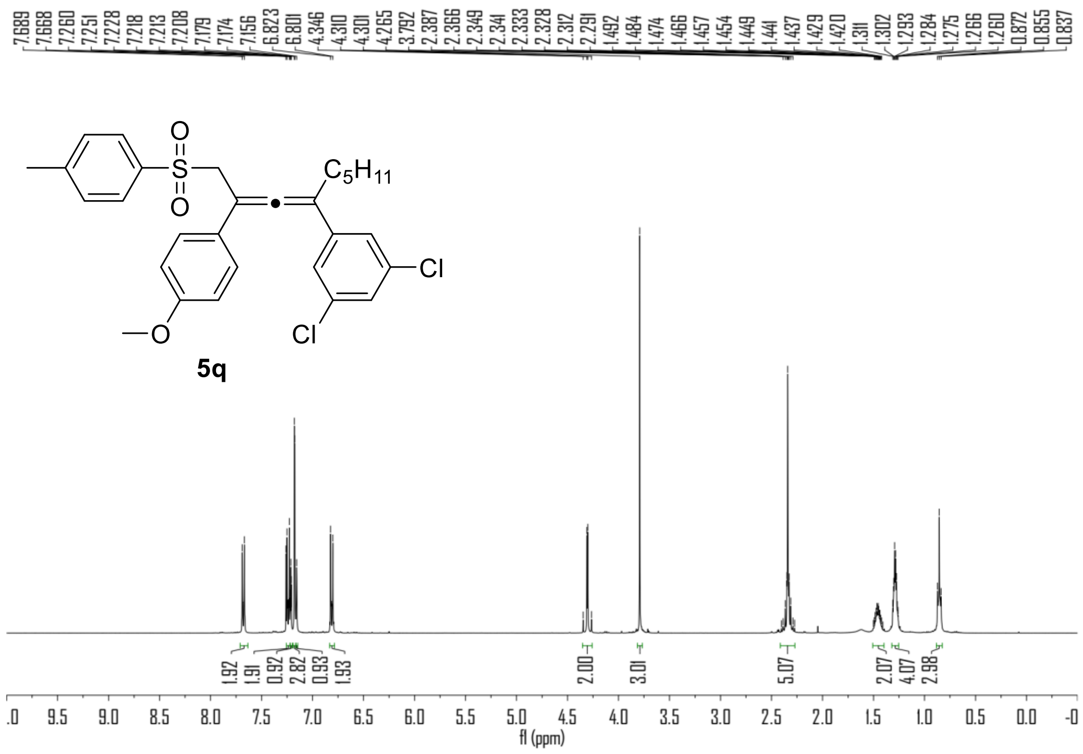
7.689 7.668 7.260 7.251 7.228 7.218 7.213 7.208 7.179 7.174 7.156 6.823 6.801 4.346 4.310 4.301 4.265 3.792 2.387 2.366 2.349 2.341 2.333 2.328 2.312 2.291 1.492 1.484 1.474 1.466 1.457 1.454 1.449 1.441 1.437 1.429 1.420 1.311 1.302 1.293 1.284 1.275 1.266 1.260 0.872 0.855 0.837
O
S
O
C5H11
Cl
Cl
O
5q
1.92 1.91 0.92 2.82 0.93 1.93
2.00
3.01
5.07
2.07 4.07 2.98
fl (ppm)

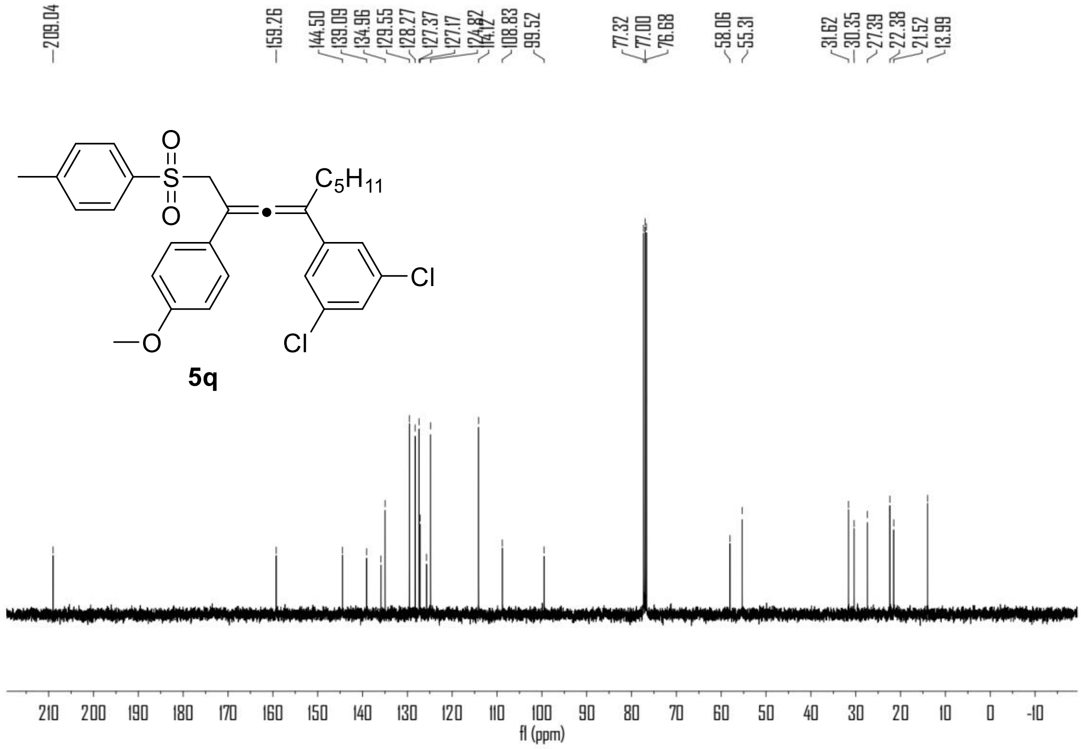
209.04
159.26
144.50 139.09 134.96 129.55 128.27 127.37 127.17 124.82 114.12 108.83 99.52
77.32 77.00 76.68
58.06 55.31
31.62 30.35 27.39 22.38 21.52 13.99
O
S
O
C5H11
Cl
Cl
O
5q
fl (ppm)

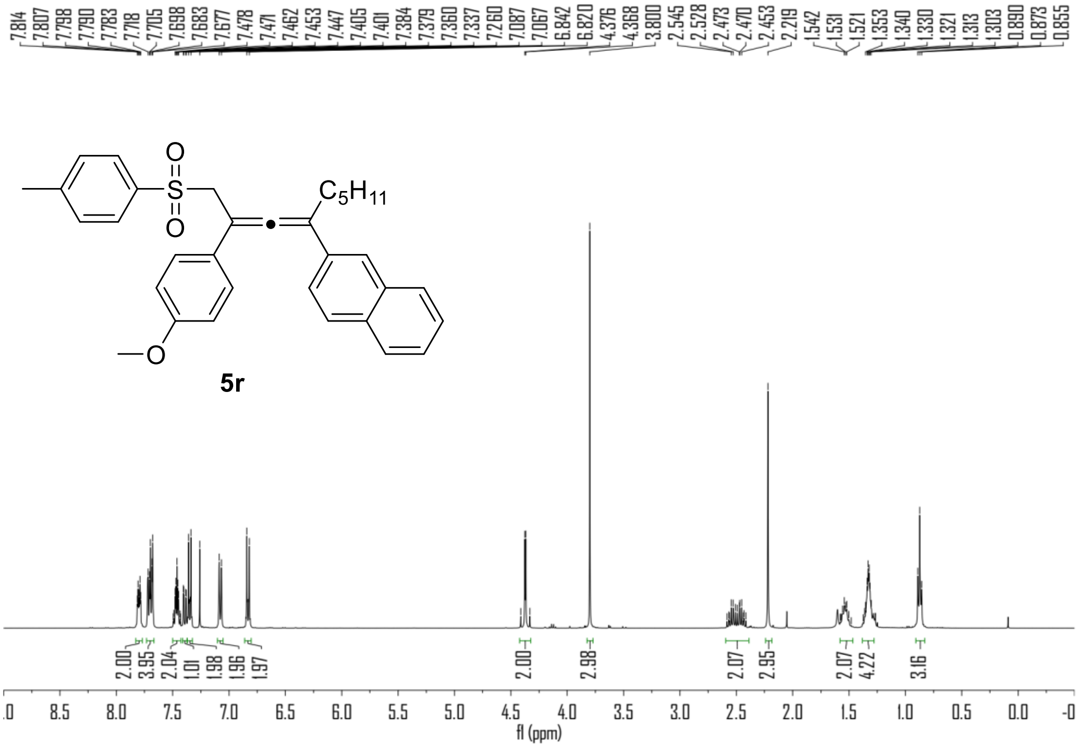
7.814
7.807
7.798
7.790
7.783
7.718
7.705
7.698
7.683
7.677
7.478
7.471
7.462
7.453
7.447
7.405
7.401
7.384
7.379
7.360
7.337
7.260
7.087
7.067
6.842
6.820
4.376
4.368
3.800
2.545
2.528
2.473
2.470
2.453
2.219
1.542
1.531
1.521
1.353
1.340
1.330
1.321
1.313
1.303
0.890
0.873
0.855
O
S
O
C_5H_{11}
O
5r
2.00
3.95
2.04
1.01
1.98
1.96
1.97
2.00
2.98
2.07
2.95
2.07
4.22
3.16
fl (ppm)

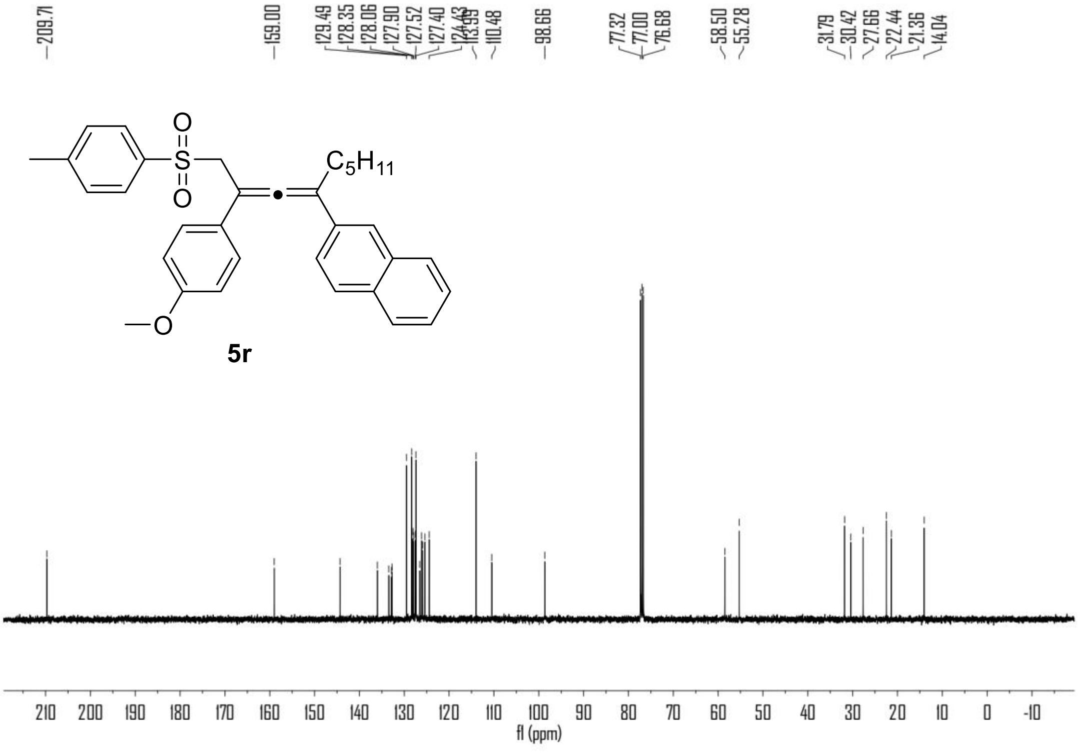
209.71
159.00
129.49
128.35
128.06
127.90
127.52
127.40
113.99
110.48
98.66
77.32
77.00
76.68
58.50
55.28
31.79
30.42
27.66
22.44
21.36
14.04
O
S
O
C_5H_{11}
O
5r
fl (ppm)

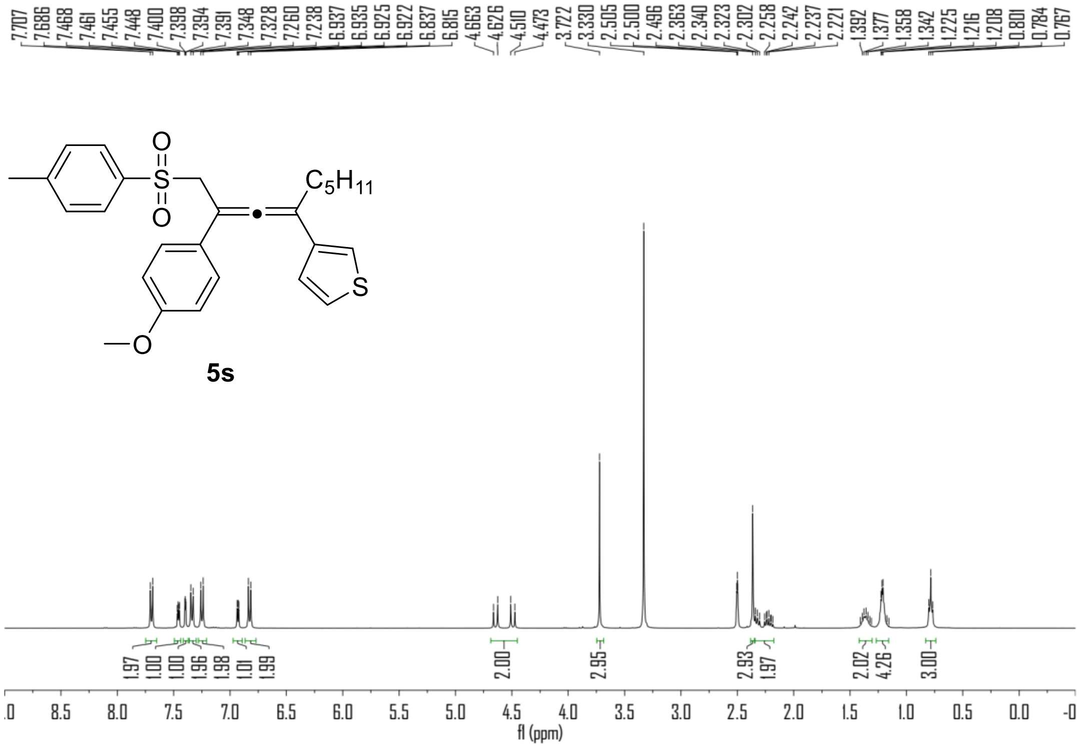

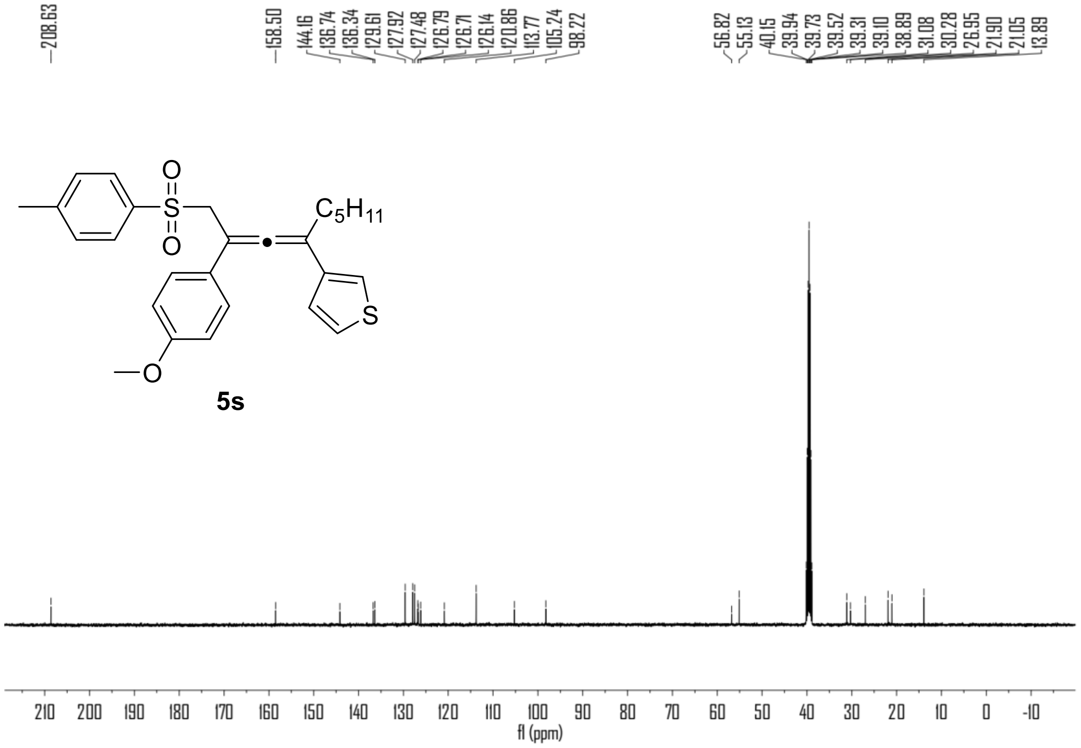

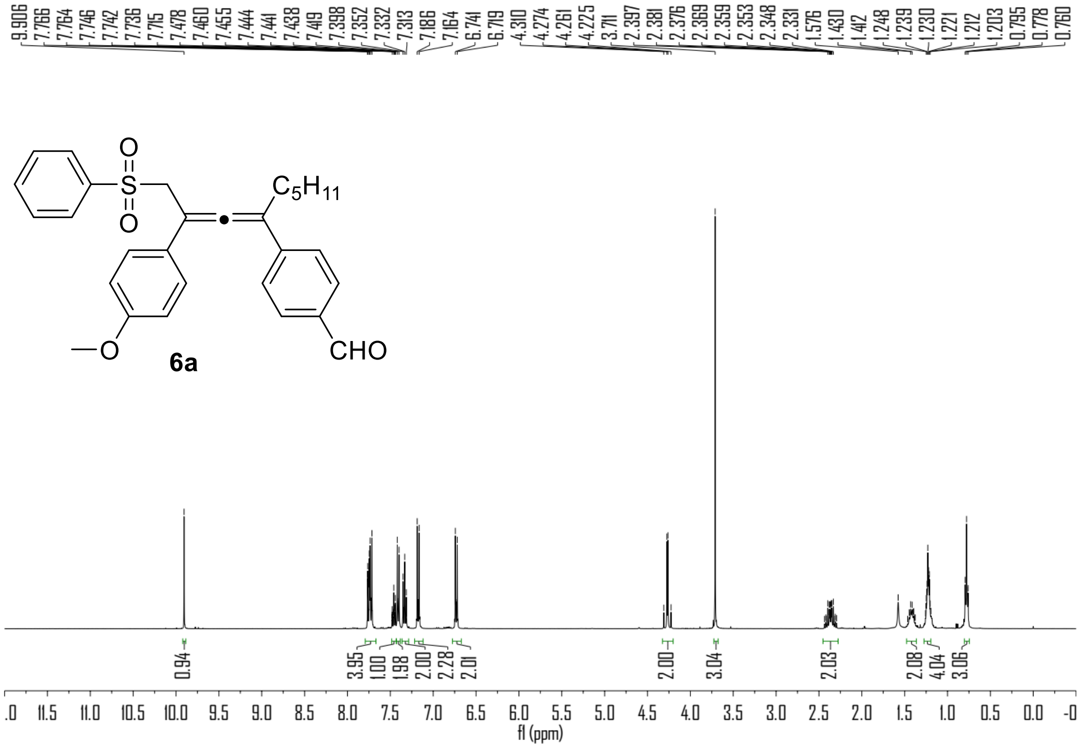

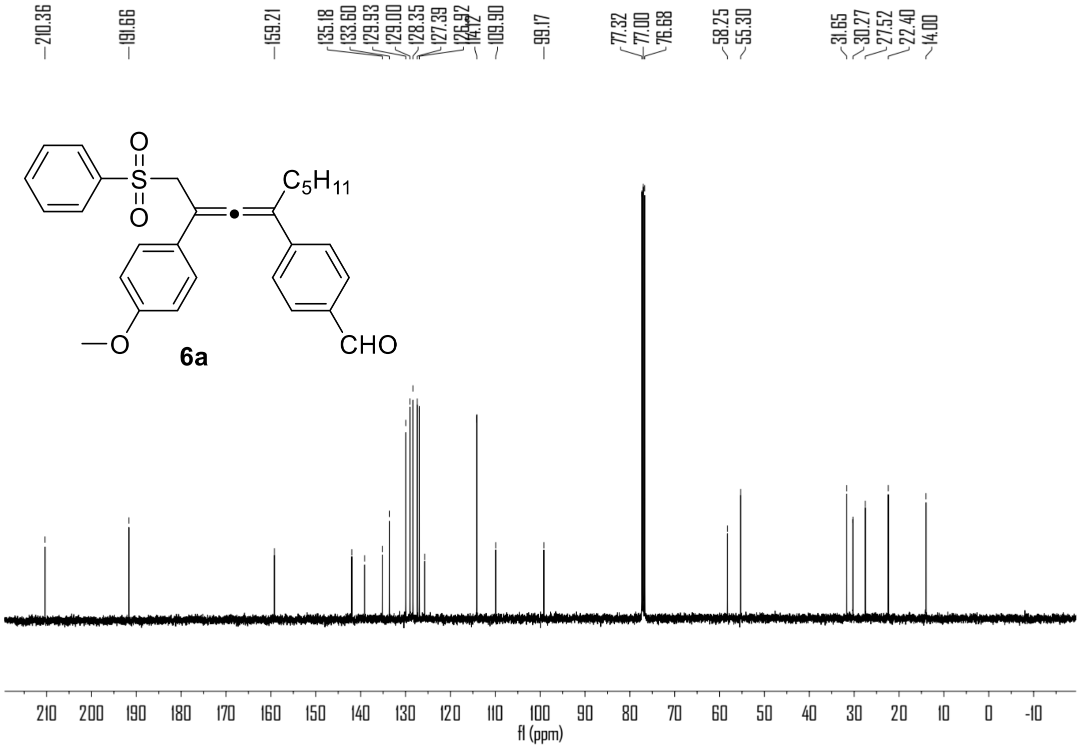

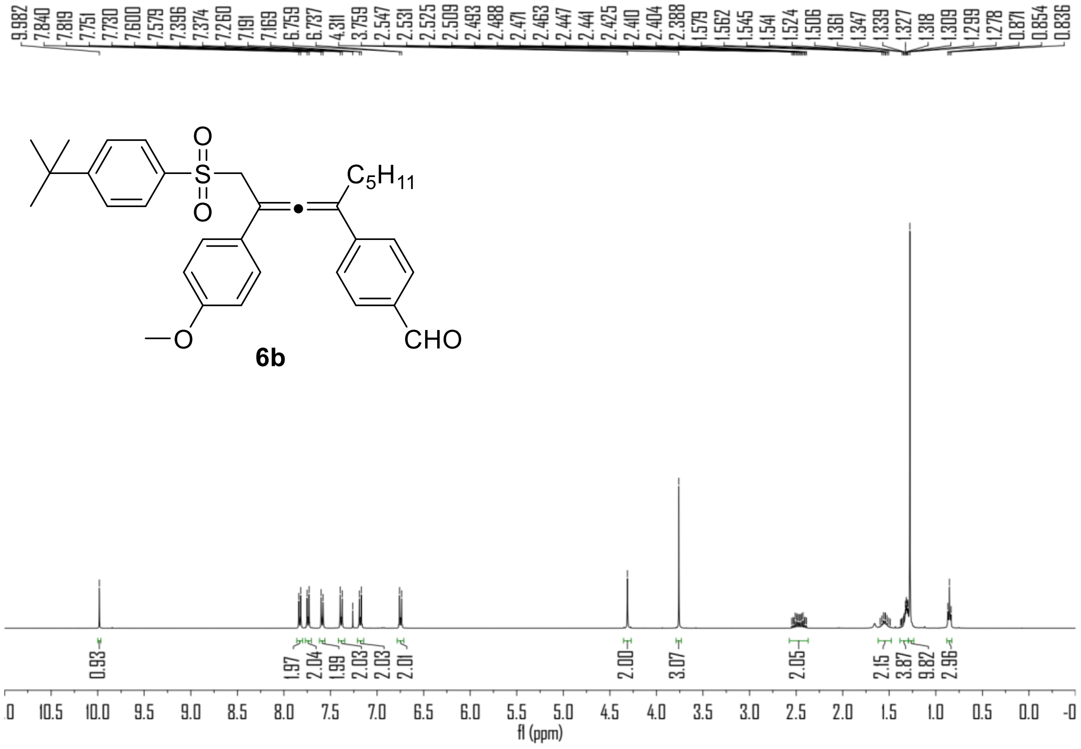
9.982 7.840 7.819 7.751 7.730 7.600 7.579 7.396 7.374 7.260 7.191 7.169 6.759 6.737 4.311 3.759 2.547 2.531 2.525 2.509 2.493 2.488 2.471 2.463 2.447 2.441 2.425 2.410 2.404 2.388 1.579 1.562 1.545 1.541 1.524 1.506 1.361 1.347 1.339 1.327 1.318 1.309 1.299 1.278 0.871 0.854 0.836
O
S
O
C_5H_{11}
O
CHO
6b
0.93
1.97
2.04
1.99
2.03
2.03
2.01
2.00
3.07
2.05
2.15
3.87
9.82
2.96
10.5 10.0 9.5 9.0 8.5 8.0 7.5 7.0 6.5 6.0 5.5 5.0 4.5 4.0 3.5 3.0 2.5 2.0 1.5 1.0 0.5 0.0
f1 (ppm)

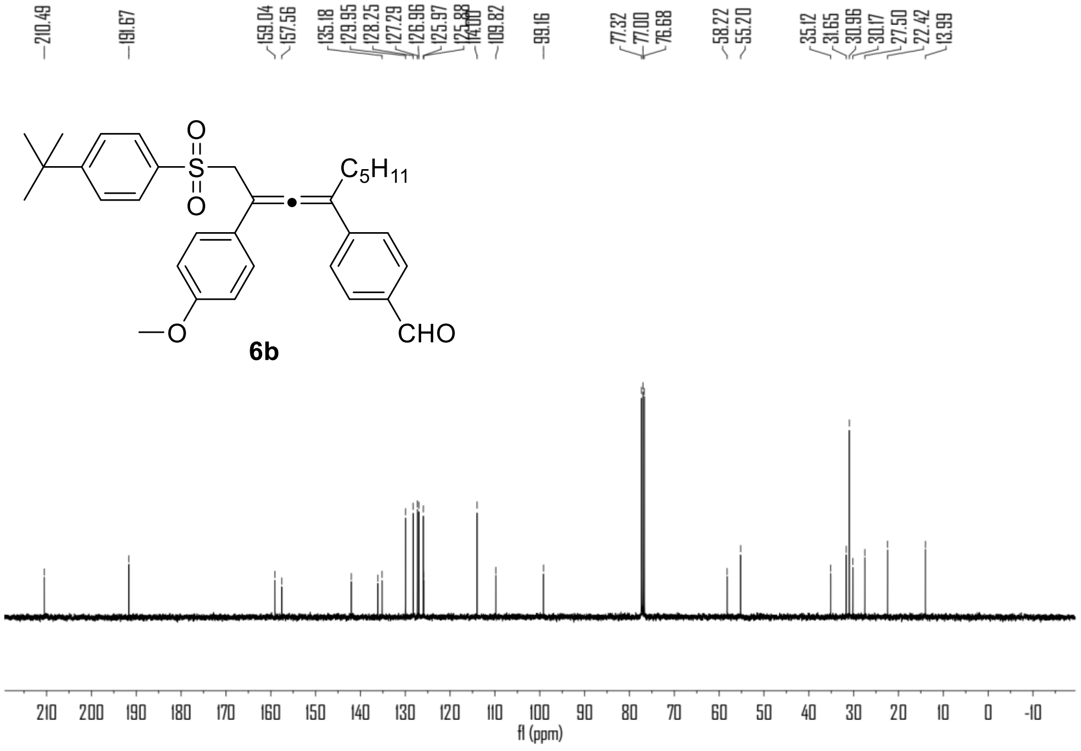
210.49 191.67 159.04 157.56 135.18 129.95 128.25 127.29 126.96 125.97 125.88 114.00 109.82 99.16 77.32 77.00 76.68 58.22 55.20 35.12 31.65 30.96 30.17 27.50 22.42 13.99
O
S
O
C_5H_{11}
O
CHO
6b
210 200 190 180 170 160 150 140 130 120 110 100 90 80 70 60 50 40 30 20 10 0 -10
f1 (ppm)

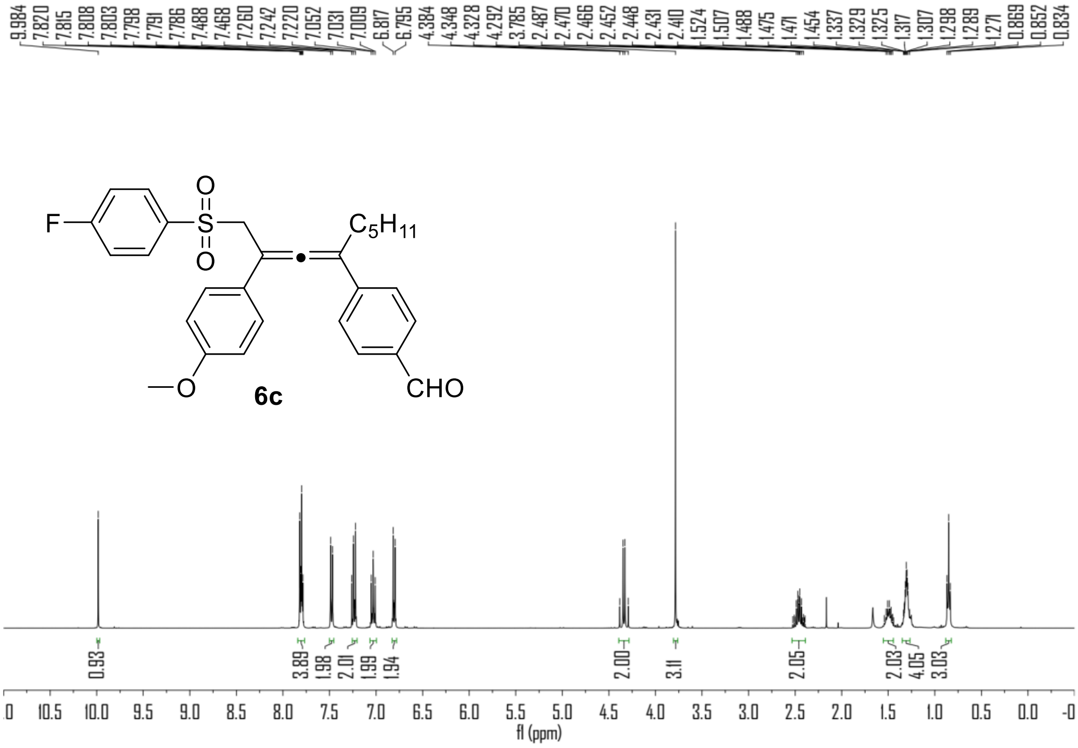
9.984
7.820
7.815
7.808
7.803
7.798
7.791
7.786
7.488
7.468
7.260
7.242
7.220
7.052
7.031
7.009
6.817
6.795
4.384
4.348
4.328
4.292
3.785
2.487
2.470
2.466
2.452
2.448
2.431
2.410
1.524
1.507
1.488
1.475
1.471
1.454
1.337
1.329
1.325
1.317
1.307
1.298
1.289
1.271
0.869
0.852
0.834
F
O
S
O
C_5H_{11}
O
6c
CHO
0.93
3.89
1.98
2.01
1.99
1.94
2.00
3.11
2.05
2.03
4.05
3.03
10.5 10.0 9.5 9.0 8.5 8.0 7.5 7.0 6.5 6.0 5.5 5.0 4.5 4.0 3.5 3.0 2.5 2.0 1.5 1.0 0.5 0.0
f1 (ppm)

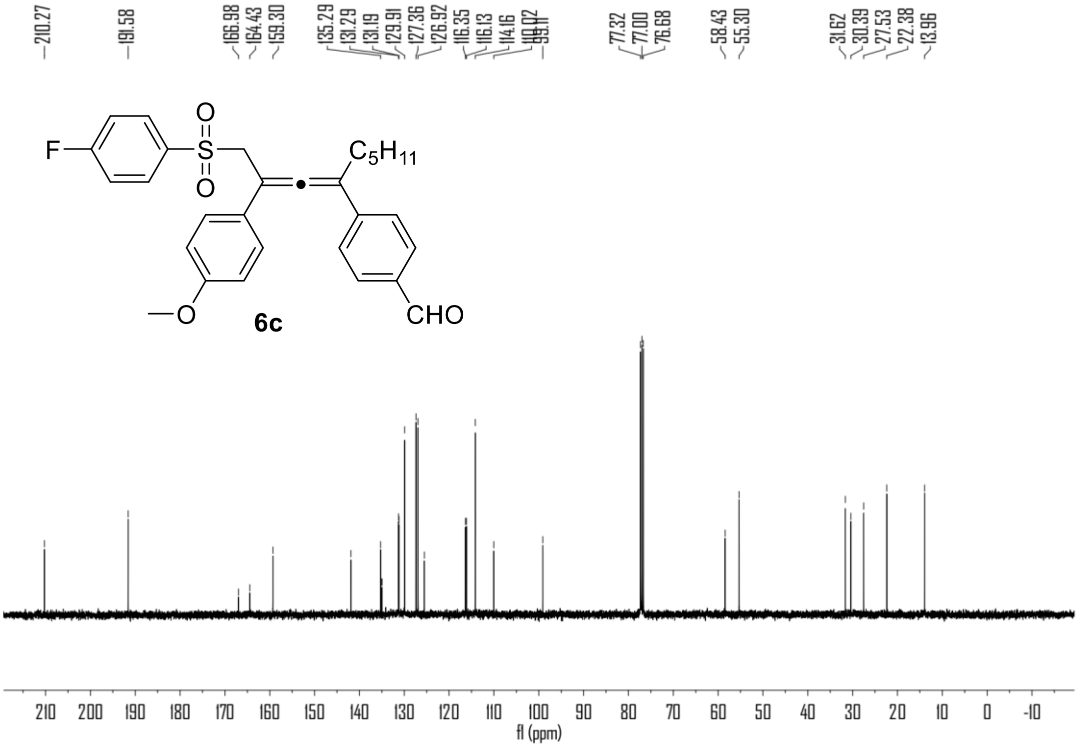
210.27
191.58
166.98
164.43
159.30
135.29
131.29
131.19
129.91
127.36
126.92
116.35
116.13
114.16
110.02
99.11
77.32
77.00
76.68
58.43
55.30
31.62
30.39
27.53
22.38
13.96
F
O
S
O
C_5H_{11}
O
6c
CHO
210 200 190 180 170 160 150 140 130 120 110 100 90 80 70 60 50 40 30 20 10 0 -10
f1 (ppm)

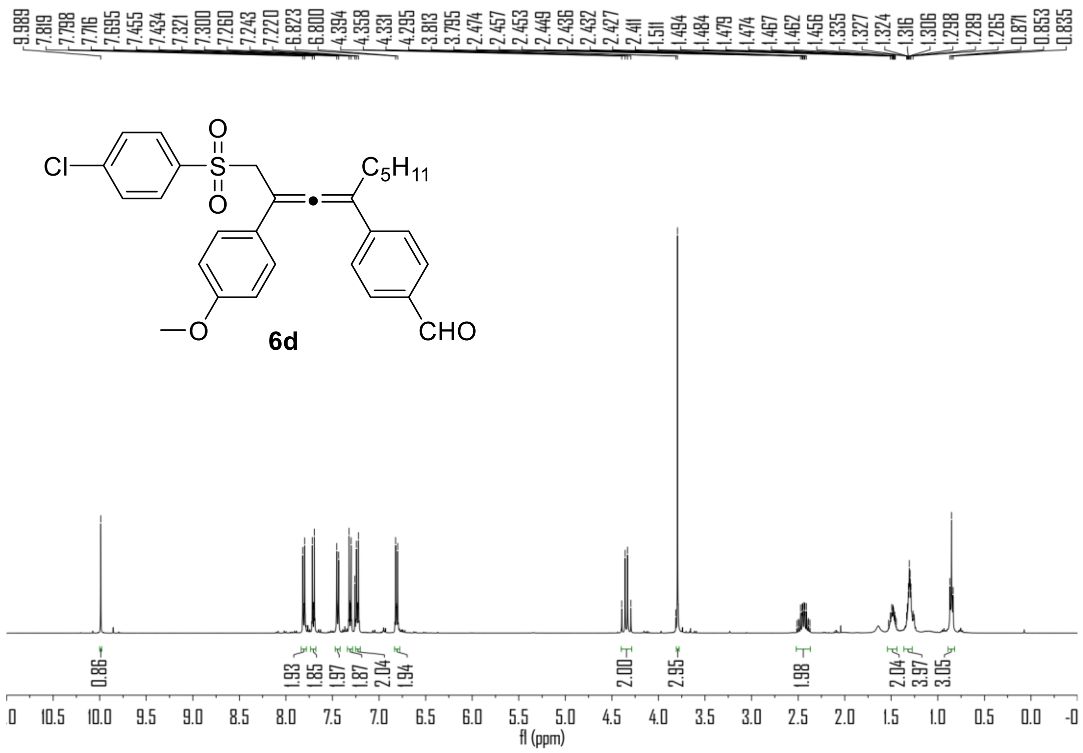

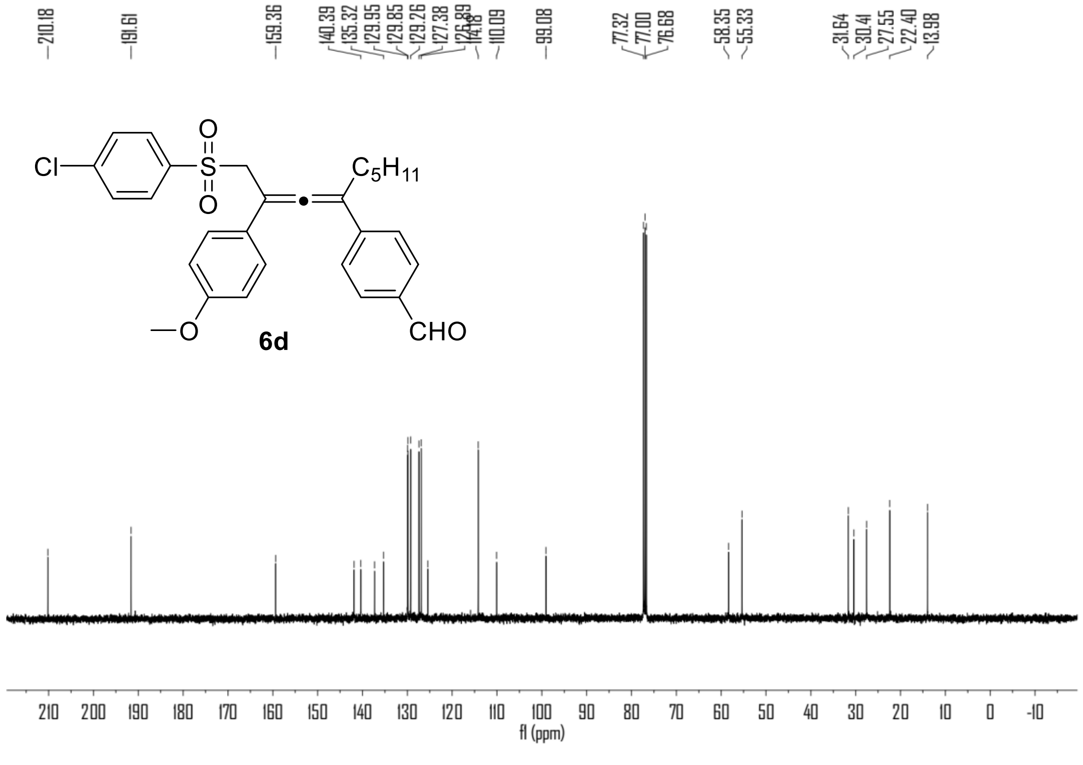

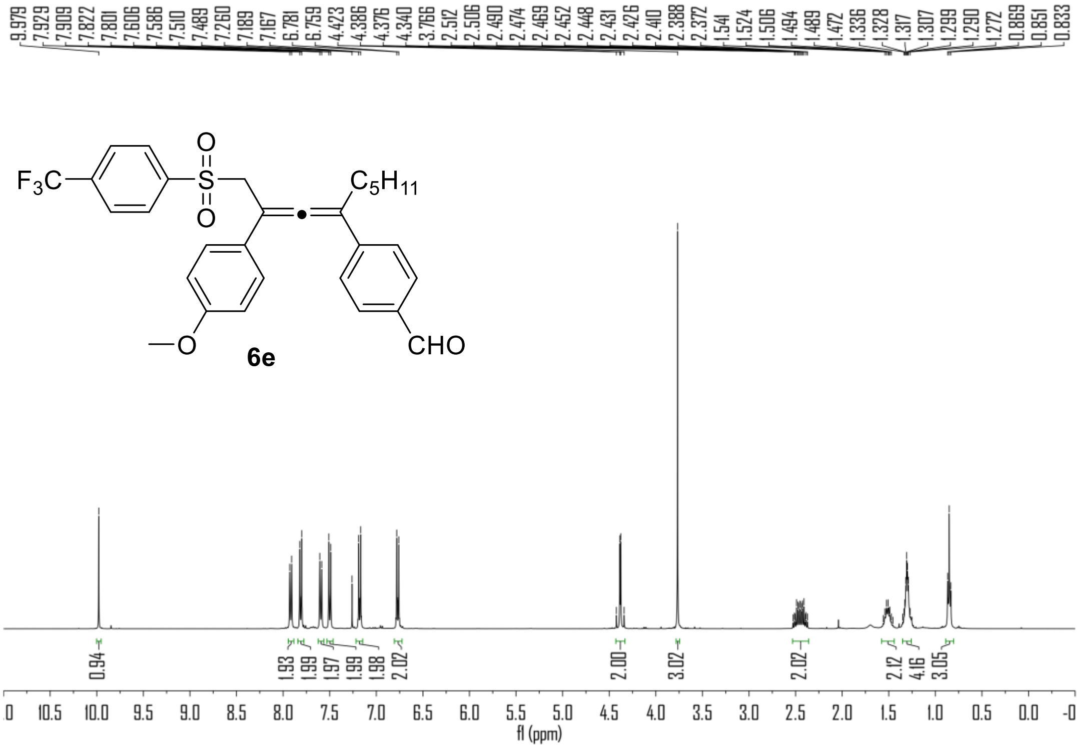
9.979
7.929
7.909
7.822
7.801
7.606
7.586
7.510
7.489
7.260
7.189
7.167
6.781
6.759
4.423
4.386
4.376
4.340
3.766
2.512
2.506
2.490
2.474
2.469
2.452
2.448
2.431
2.426
2.410
2.388
2.372
1.541
1.524
1.506
1.494
1.489
1.472
1.336
1.328
1.317
1.307
1.299
1.290
1.272
0.869
0.851
0.833
F_3C
O
S
O
C_5H_{11}
O
6e
CHO
0.94
1.93
1.99
1.97
1.99
1.98
2.02
2.00
3.02
2.02
2.12
4.16
3.05
10.5
10.0
9.5
9.0
8.5
8.0
7.5
7.0
6.5
6.0
5.5
5.0
4.5
4.0
3.5
3.0
2.5
2.0
1.5
1.0
0.5
0.0
fl (ppm)

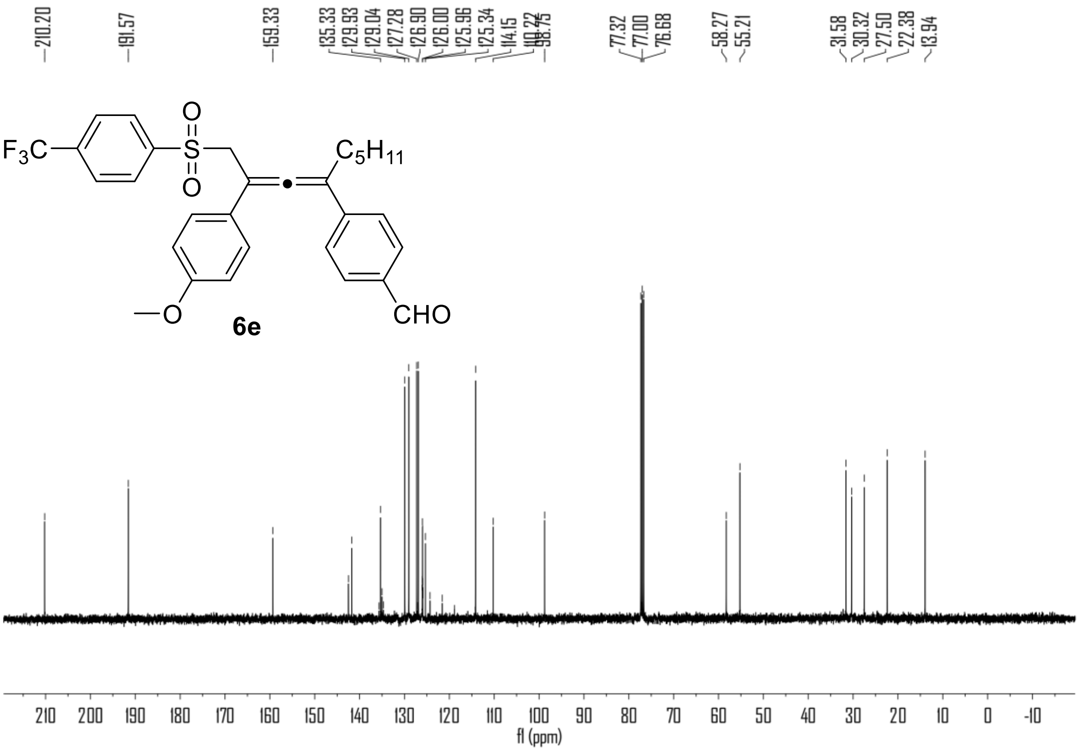
210.20
191.57
159.33
135.33
129.93
129.04
127.28
126.90
126.00
125.96
125.34
114.15
110.22
98.75
77.32
77.00
76.68
58.27
55.21
31.58
30.32
27.50
22.38
13.94
F_3C
O
S
O
C_5H_{11}
O
6e
CHO
210
200
190
180
170
160
150
140
130
120
110
100
90
80
70
60
50
40
30
20
10
0
-10
fl (ppm)

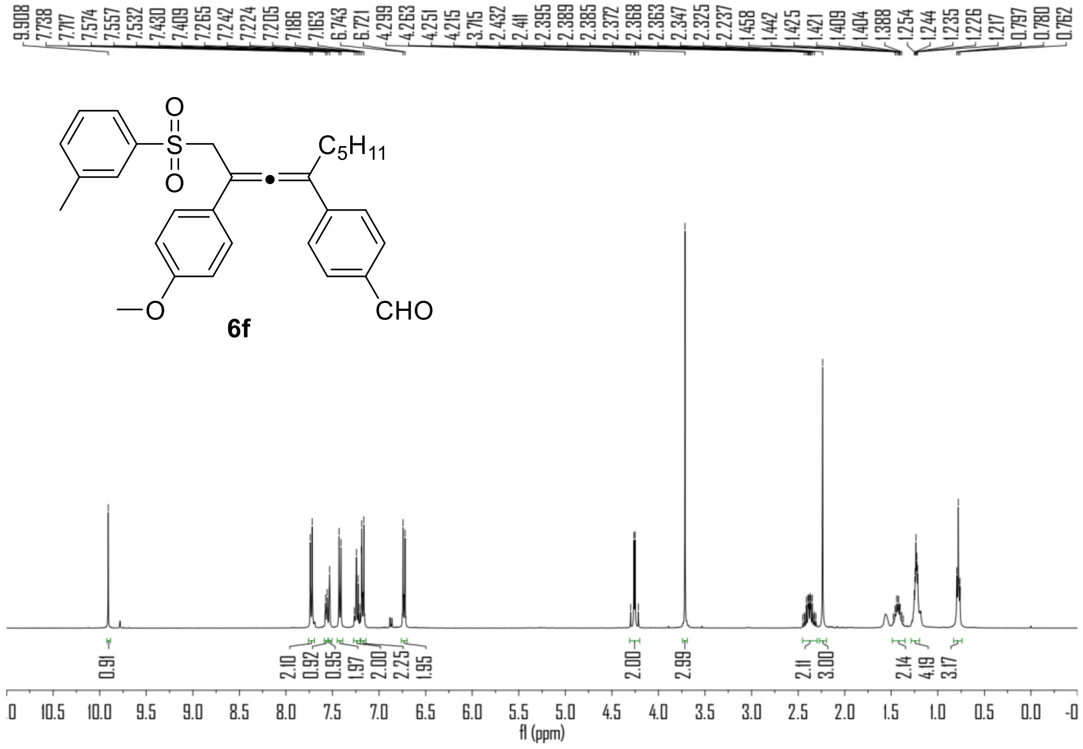
9.908 7.738 7.717 7.574 7.557 7.532 7.430 7.409 7.265 7.242 7.224 7.205 7.186 7.163 6.743 6.721 4.299 4.263 4.251 4.215 3.715 2.432 2.411 2.395 2.389 2.385 2.372 2.368 2.363 2.347 2.325 2.237 1.458 1.442 1.425 1.421 1.409 1.404 1.388 1.254 1.244 1.235 1.226 1.217 0.797 0.780 0.762
C5H11
O
S
O
O
CHO
6f
0.91
2.10 0.92 0.95 1.97 2.00 2.25 1.95
2.00
2.99
2.11 3.00
2.14 4.19 3.17
10.5 10.0 9.5 9.0 8.5 8.0 7.5 7.0 6.5 6.0 5.5 5.0 4.5 4.0 3.5 3.0 2.5 2.0 1.5 1.0 0.5 0.0
f1 (ppm)

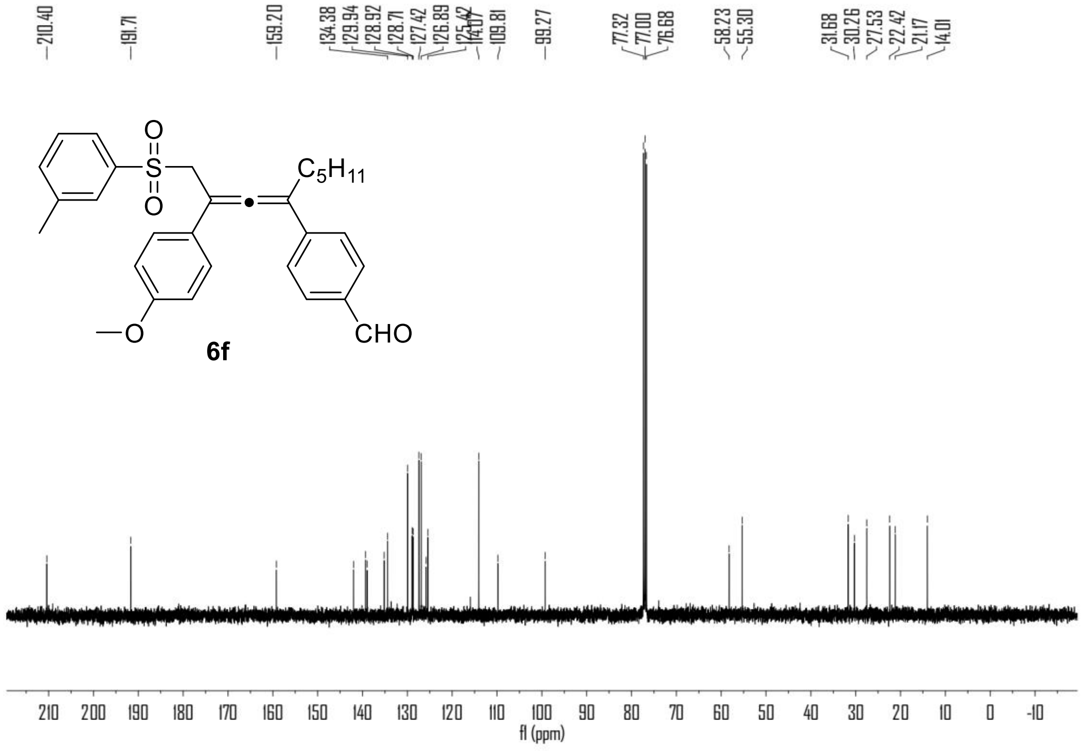
210.40 191.71 159.20 134.38 129.94 128.92 128.71 127.42 126.89 125.42 114.07 109.81 99.27 77.32 77.00 76.68 58.23 55.30 31.68 30.26 27.53 22.42 21.17 14.01
C5H11
O
S
O
O
CHO
6f
210 200 190 180 170 160 150 140 130 120 110 100 90 80 70 60 50 40 30 20 10 0 -10
f1 (ppm)

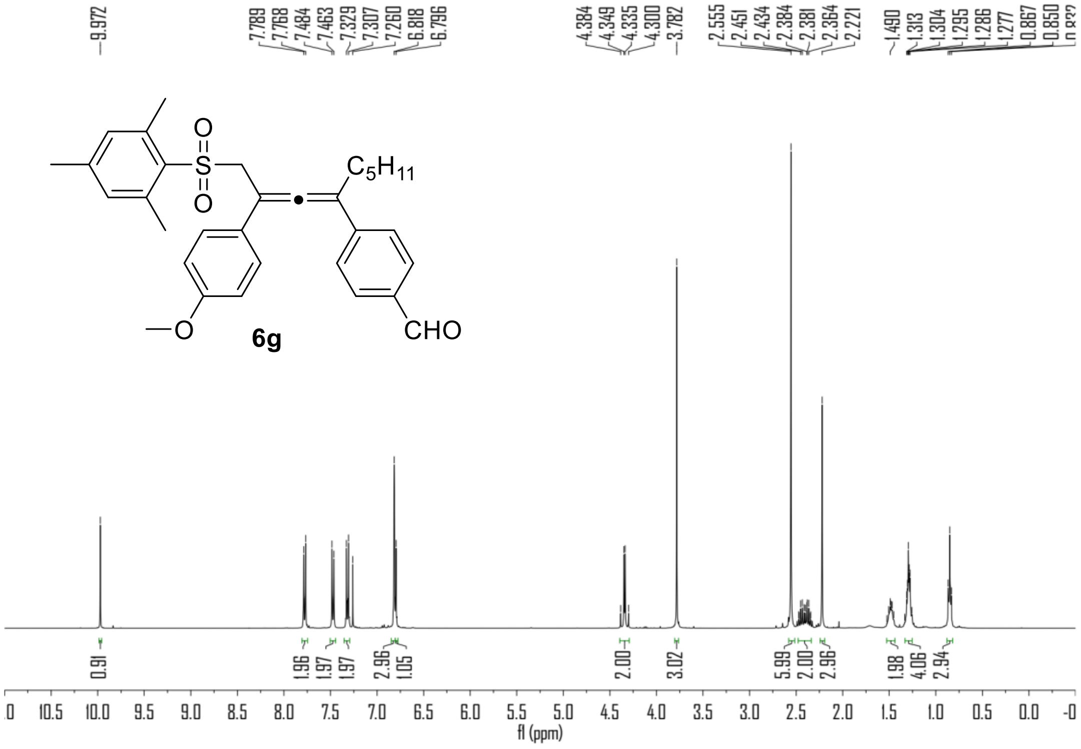

6g
CHO
fl (ppm)

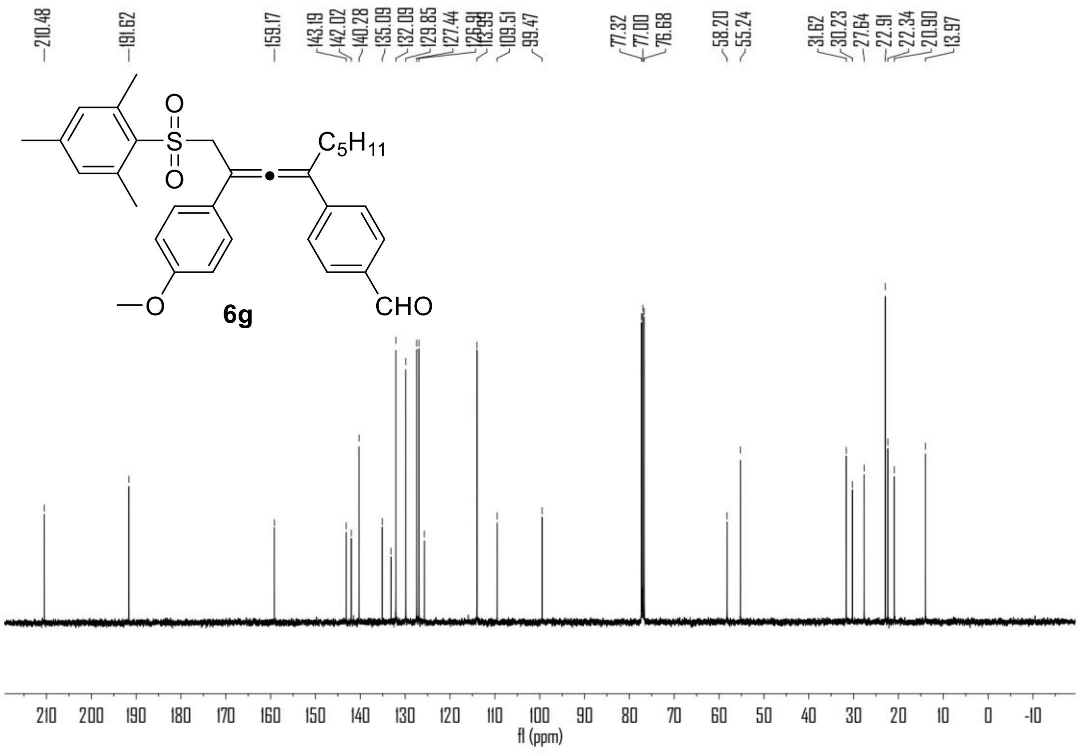

6g
CHO
fl (ppm)

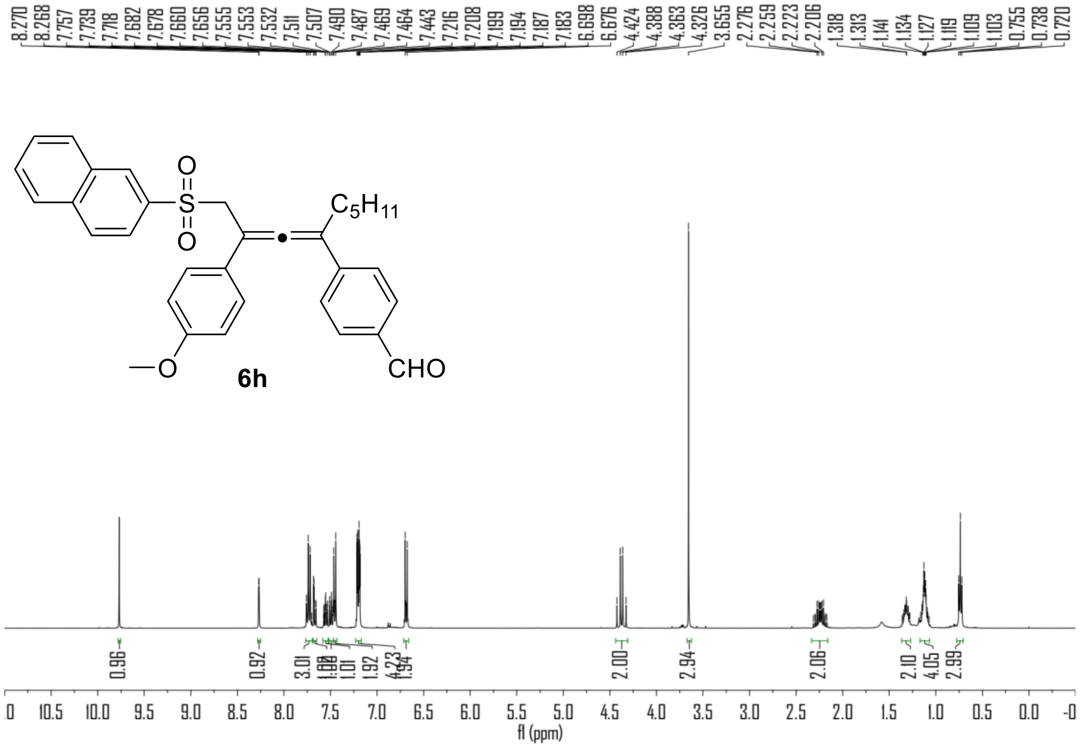

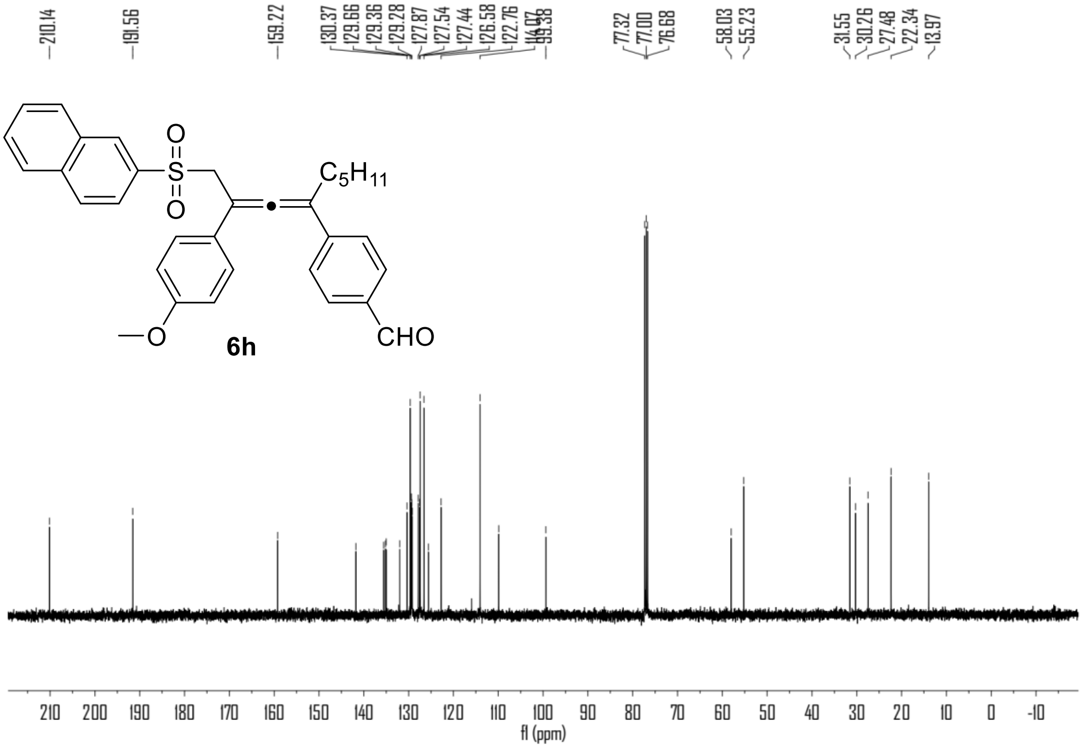

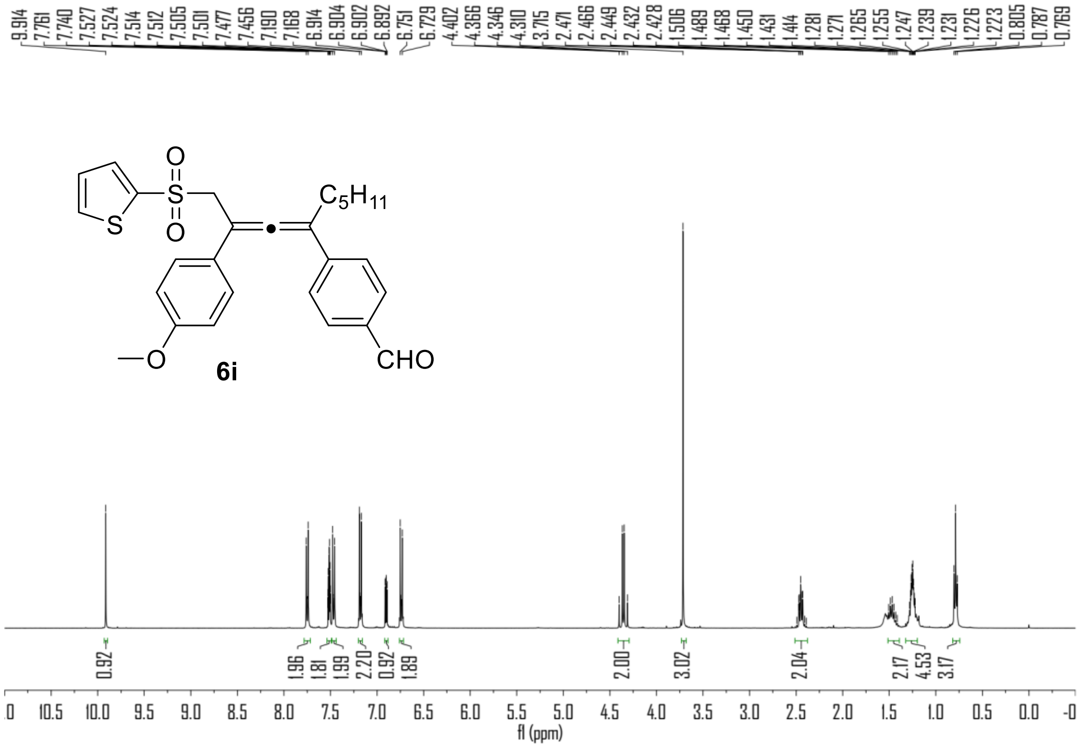

C5H11
CHO
6i
fl (ppm)

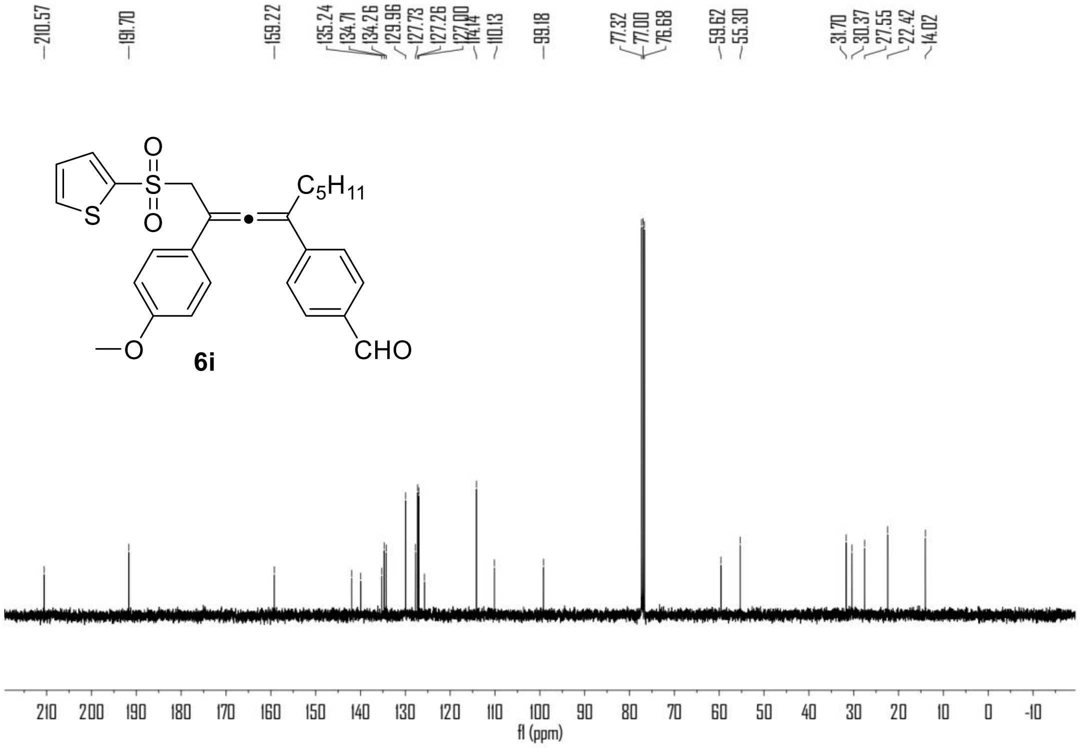

C5H11
CHO
6i
fl (ppm)

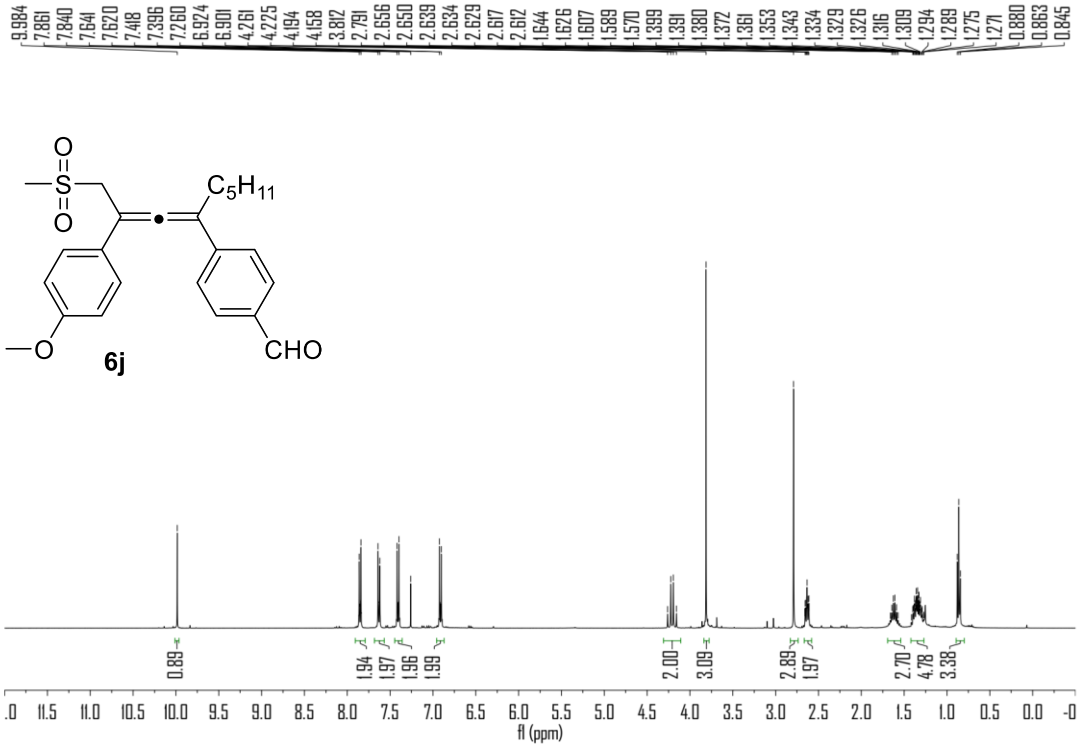
9.984
7.861
7.840
7.641
7.620
7.418
7.396
7.260
6.924
6.901
4.261
4.225
4.194
4.158
3.812
2.791
2.656
2.650
2.639
2.634
2.629
2.617
2.612
1.644
1.626
1.607
1.589
1.570
1.399
1.391
1.380
1.372
1.361
1.353
1.343
1.334
1.329
1.326
1.316
1.309
1.294
1.289
1.275
1.271
0.880
0.863
0.845
C5H11
CHO
6j
0.89
1.94
1.97
1.96
1.99
2.00
3.09
2.89
1.97
2.70
4.78
3.38
fl (ppm)

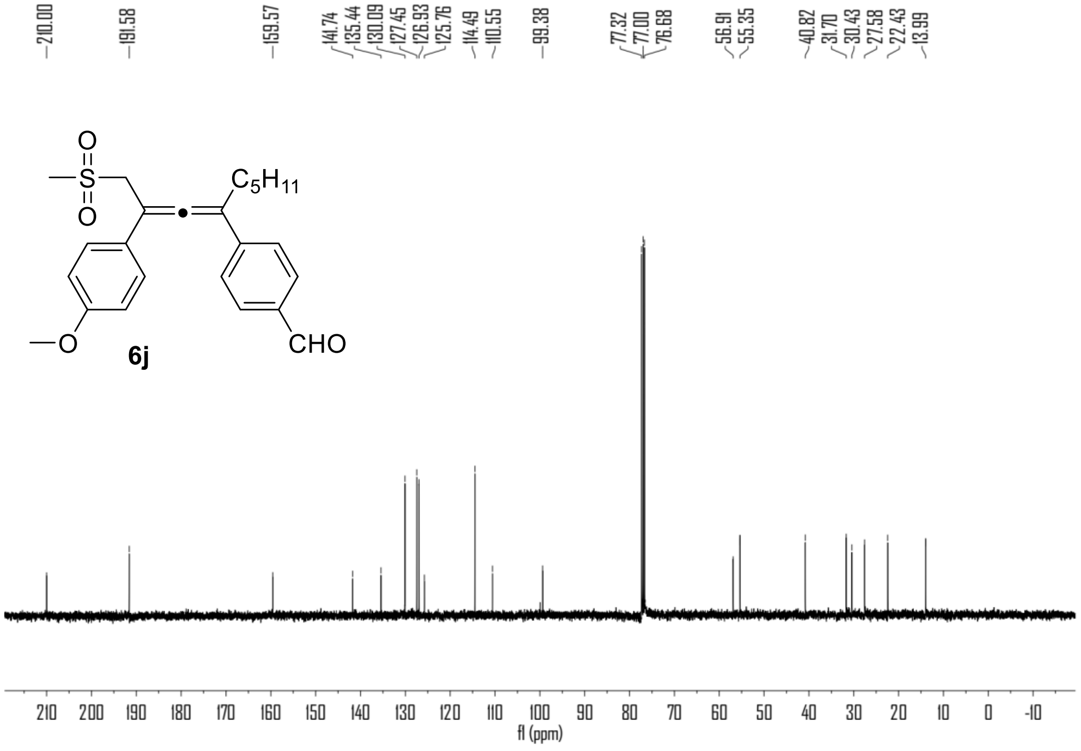
210.00
191.58
159.57
141.74
135.44
130.09
127.45
126.93
125.76
114.49
110.55
99.38
77.32
77.00
76.68
56.91
55.35
40.82
31.70
30.43
27.58
22.43
13.99
C5H11
CHO
6j
fl (ppm)

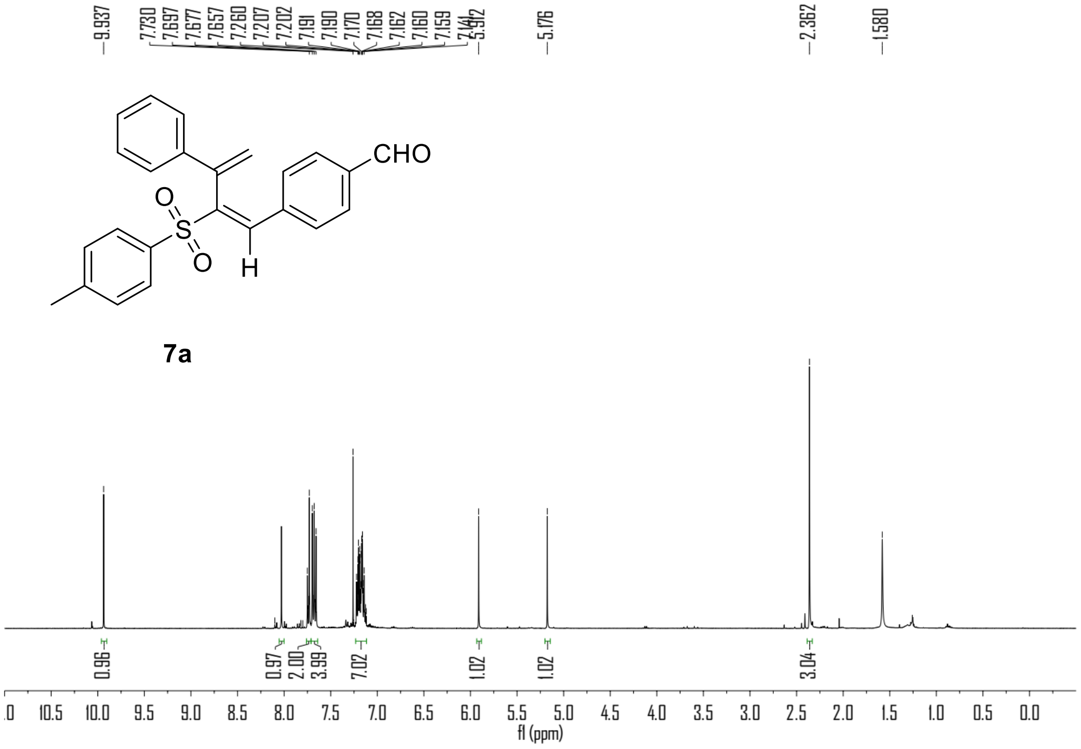
9.937
7.730
7.697
7.677
7.657
7.260
7.207
7.202
7.191
7.190
7.170
7.168
7.162
7.160
7.159
7.141
5.912
5.176
2.362
1.580
CHO
O
S
O
H
7a
0.96
0.97
2.00
3.99
7.02
1.02
1.02
3.04
fl (ppm)

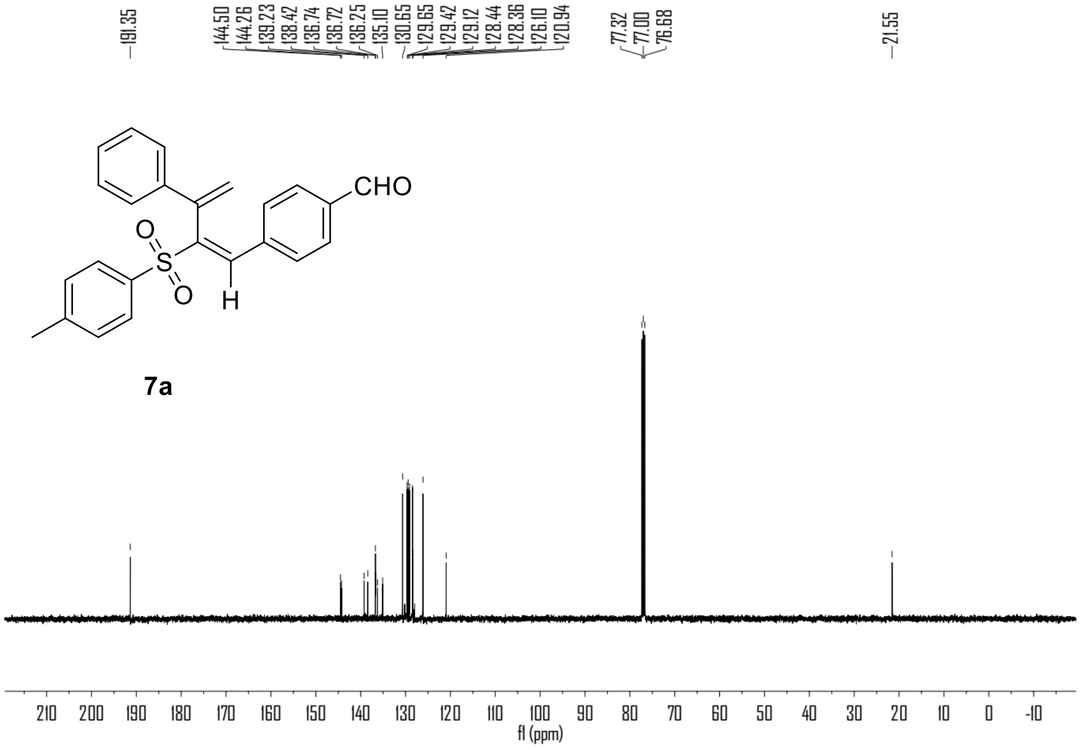
191.35
144.50
144.26
139.23
138.42
136.74
136.72
136.25
135.10
130.65
129.65
129.42
129.12
128.44
128.36
126.10
120.94
77.32
77.00
76.68
21.55
CHO
O
S
O
H
7a
fl (ppm)

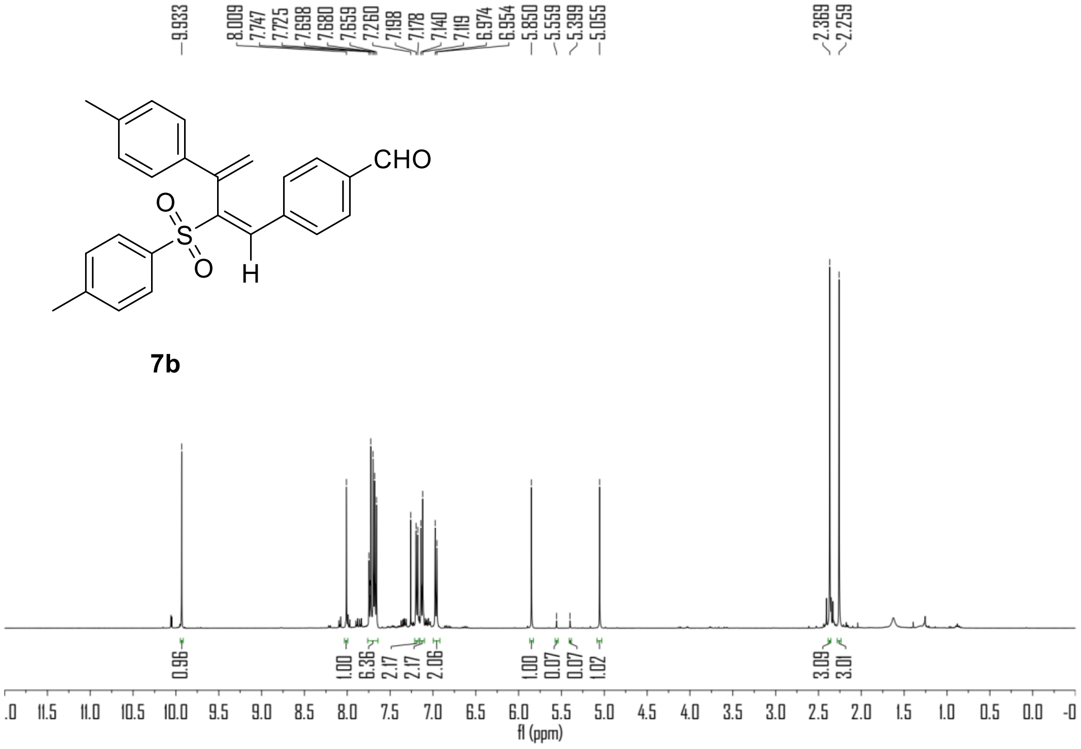

9.933
8.009
7.747
7.725
7.698
7.680
7.659
7.260
7.198
7.178
7.140
7.119
6.974
6.954
5.850
5.559
5.399
5.055
2.369
2.259
CHO
O
S
O
H
7b
0.96
1.00
6.36
2.17
2.17
2.06
1.00
0.07
0.07
1.02
3.09
3.01
fl (ppm)

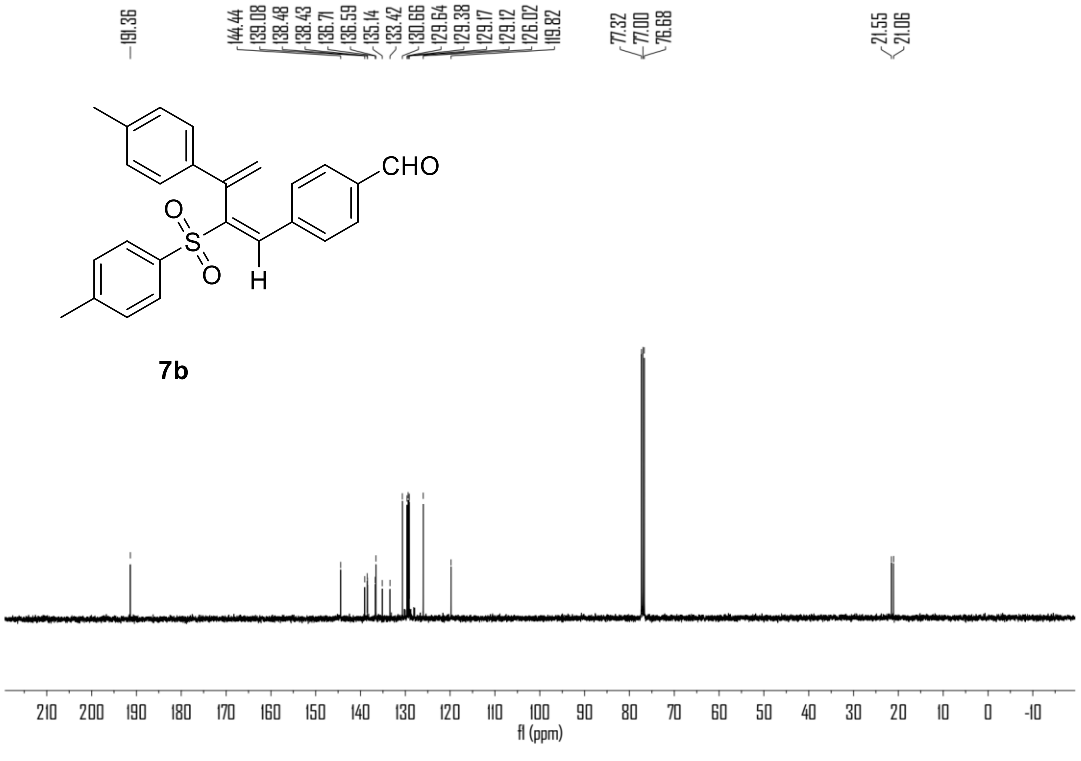

191.36
144.44
139.08
138.48
138.43
136.71
136.59
135.14
133.42
130.66
129.64
129.38
129.17
129.12
126.02
119.82
77.32
77.00
76.68
21.55
21.06
CHO
O
S
O
H
7b
fl (ppm)

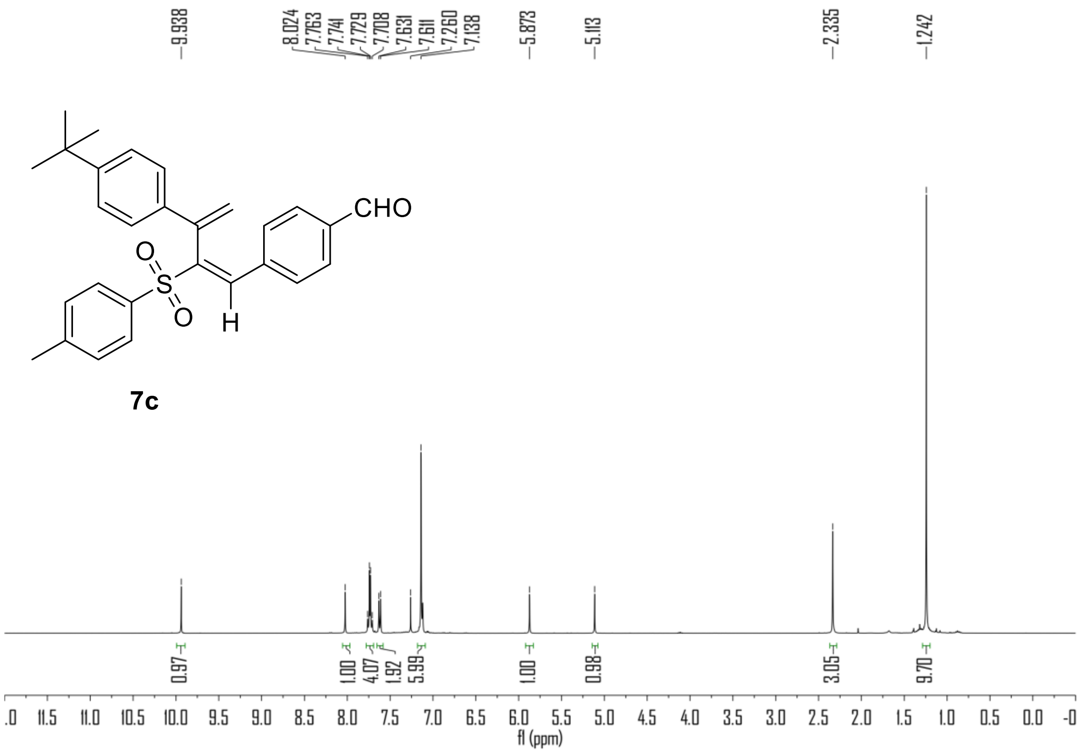
9.938
8.024
7.763
7.741
7.729
7.708
7.631
7.611
7.260
7.138
5.873
5.113
2.335
1.242
CHO
O
S
O
H
7c
0.97
1.00
4.07
1.92
5.99
1.00
0.98
3.05
9.70
f1 (ppm)

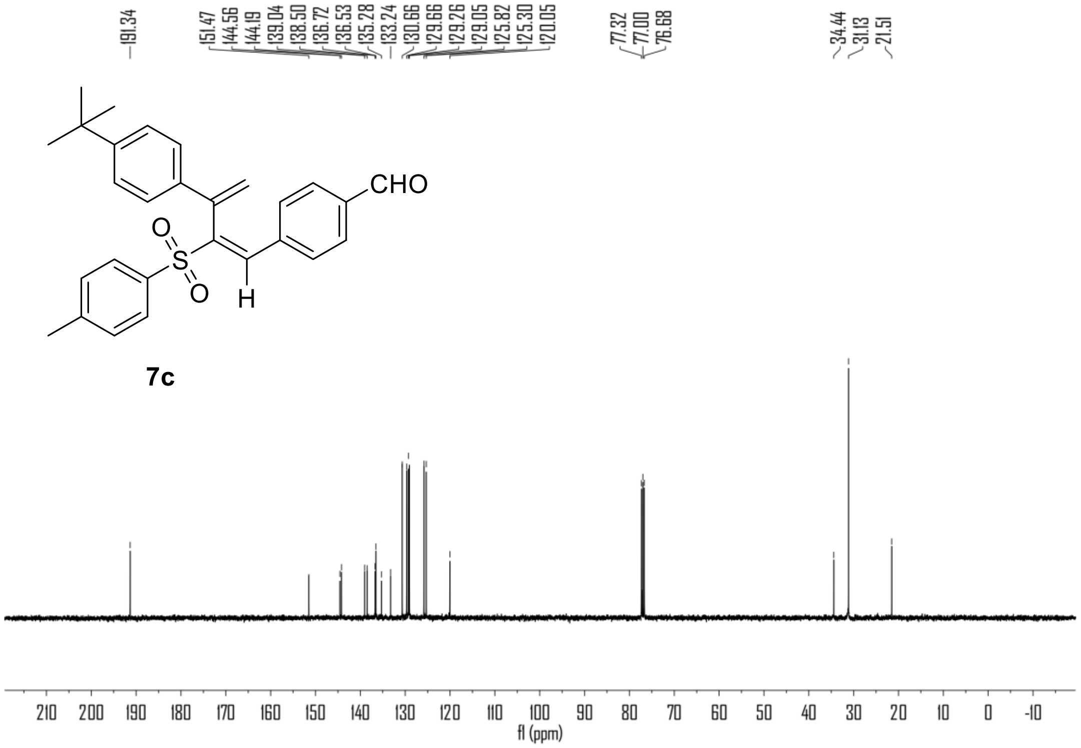
191.34
151.47
144.56
144.19
139.04
138.50
136.72
136.53
135.28
133.24
130.66
129.66
129.26
129.05
125.82
125.30
120.05
77.32
77.00
76.68
34.44
31.13
21.51
CHO
O
S
O
H
7c
f1 (ppm)

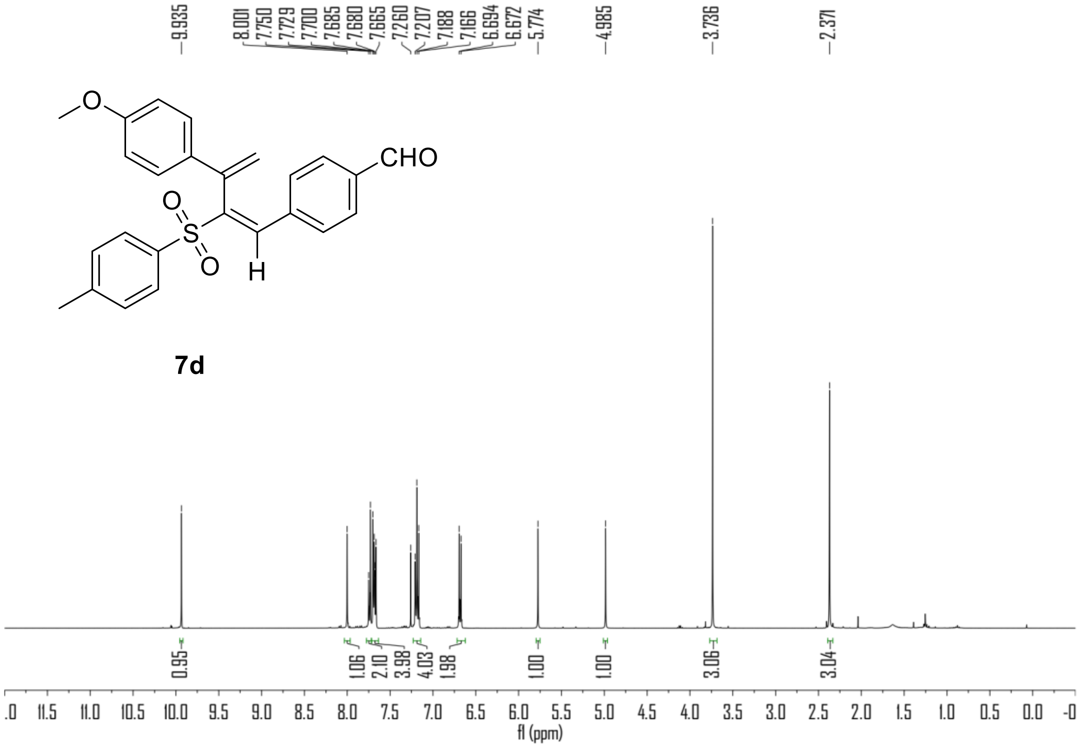

9.935
8.001 7.750 7.729 7.700 7.685 7.680 7.665 7.260 7.207 7.188 7.166 6.694 6.672 5.774
4.985
3.736
2.371
CHO
O
S
O
O
H
7d
0.95
1.06 2.10 3.98 4.03 1.98
1.00
1.00
3.06
3.04
fl (ppm)

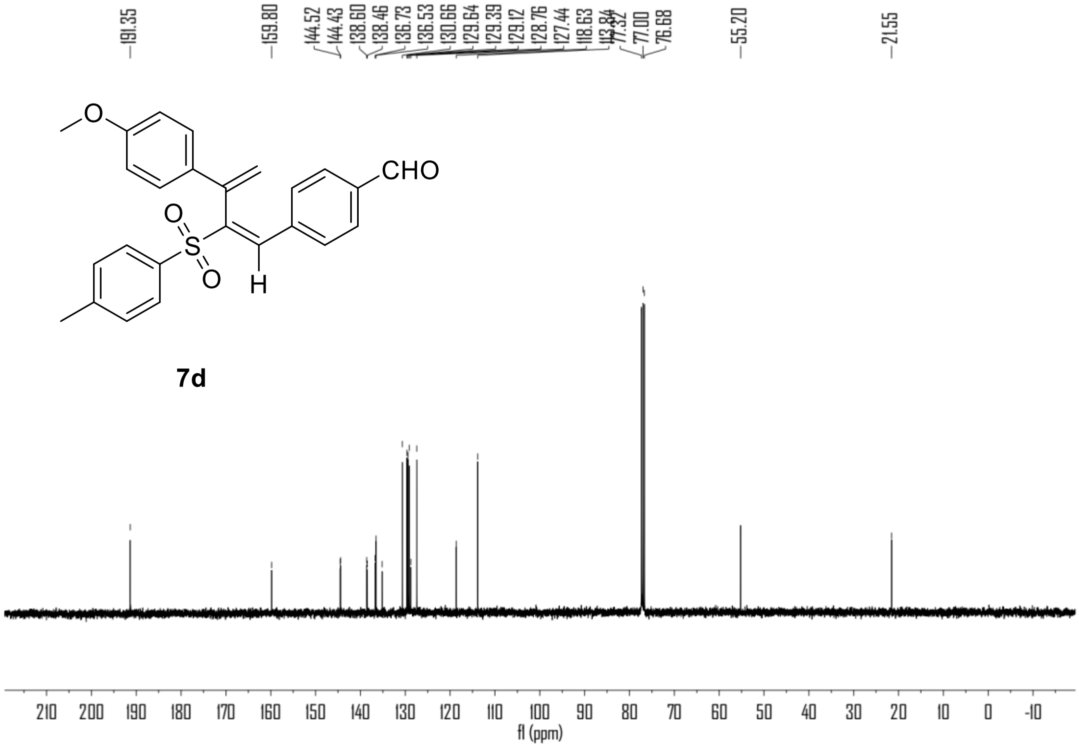

191.35
159.80
144.52 144.43 138.60 138.46 136.73 136.53 130.66 129.64 129.39 129.12 128.76 127.44 118.63 113.84 77.32 77.00 76.68
55.20
21.55
CHO
O
S
O
O
H
7d
fl (ppm)

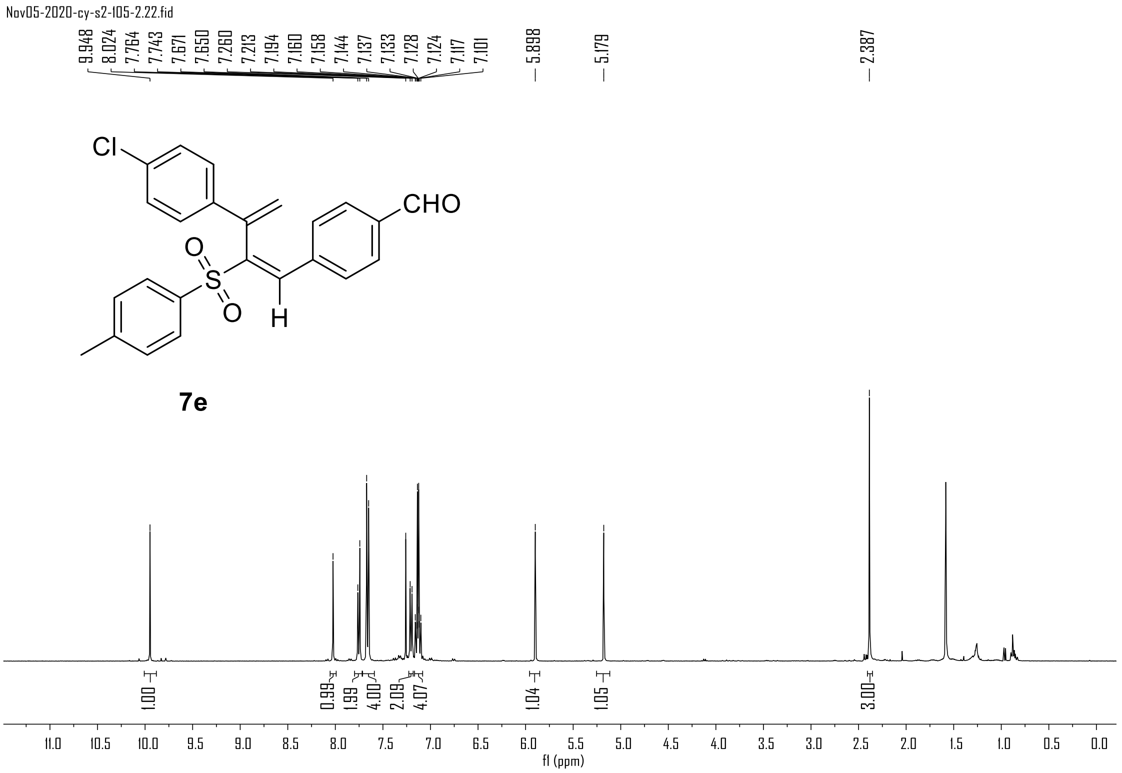
Nov05-2020-cy-s2-105-2.22.fid
9.948
8.024
7.764
7.743
7.671
7.650
7.260
7.213
7.194
7.160
7.158
7.144
7.137
7.133
7.128
7.124
7.117
7.101
5.898
5.179
2.387
Cl
CHO
O
S
O
H
7e
1.00
0.99
1.99
4.00
2.09
4.07
1.04
1.05
3.00
11.0 10.5 10.0 9.5 9.0 8.5 8.0 7.5 7.0 6.5 6.0 5.5 5.0 4.5 4.0 3.5 3.0 2.5 2.0 1.5 1.0 0.5 0.0
f1 (ppm)

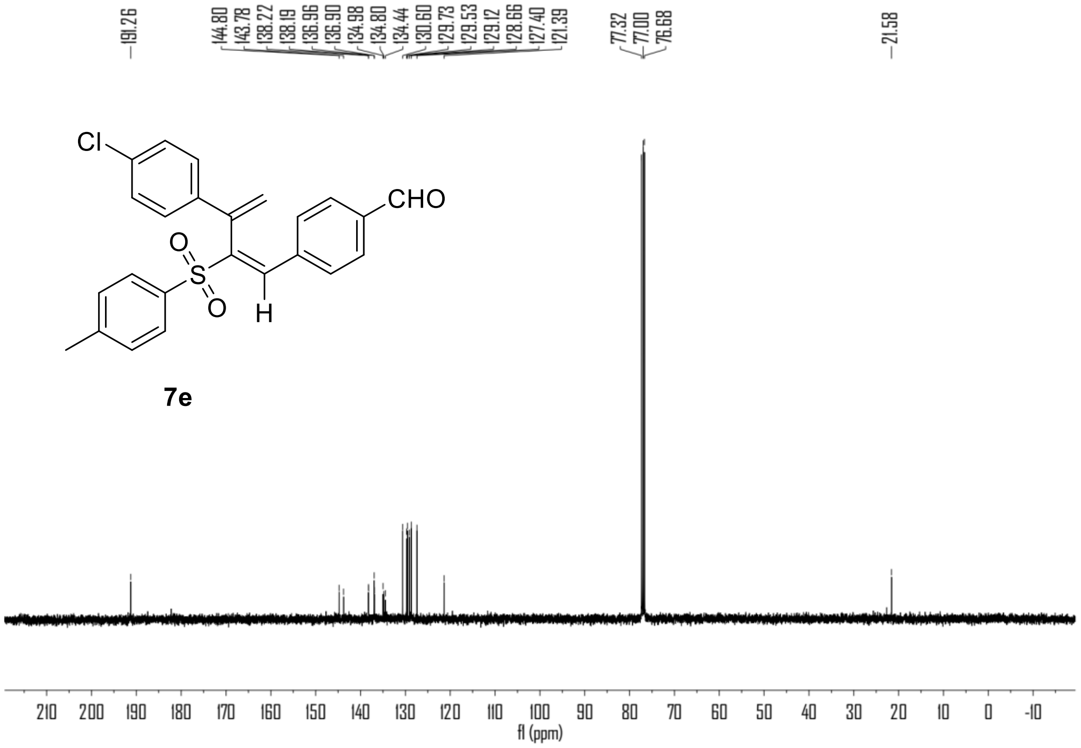
191.26
144.80
143.78
138.22
138.19
136.96
136.90
134.98
134.80
134.44
130.60
129.73
129.53
129.12
128.66
127.40
121.39
77.32
77.00
76.68
21.58
Cl
CHO
O
S
O
H
7e
210 200 190 180 170 160 150 140 130 120 110 100 90 80 70 60 50 40 30 20 10 0 -10
f1 (ppm)

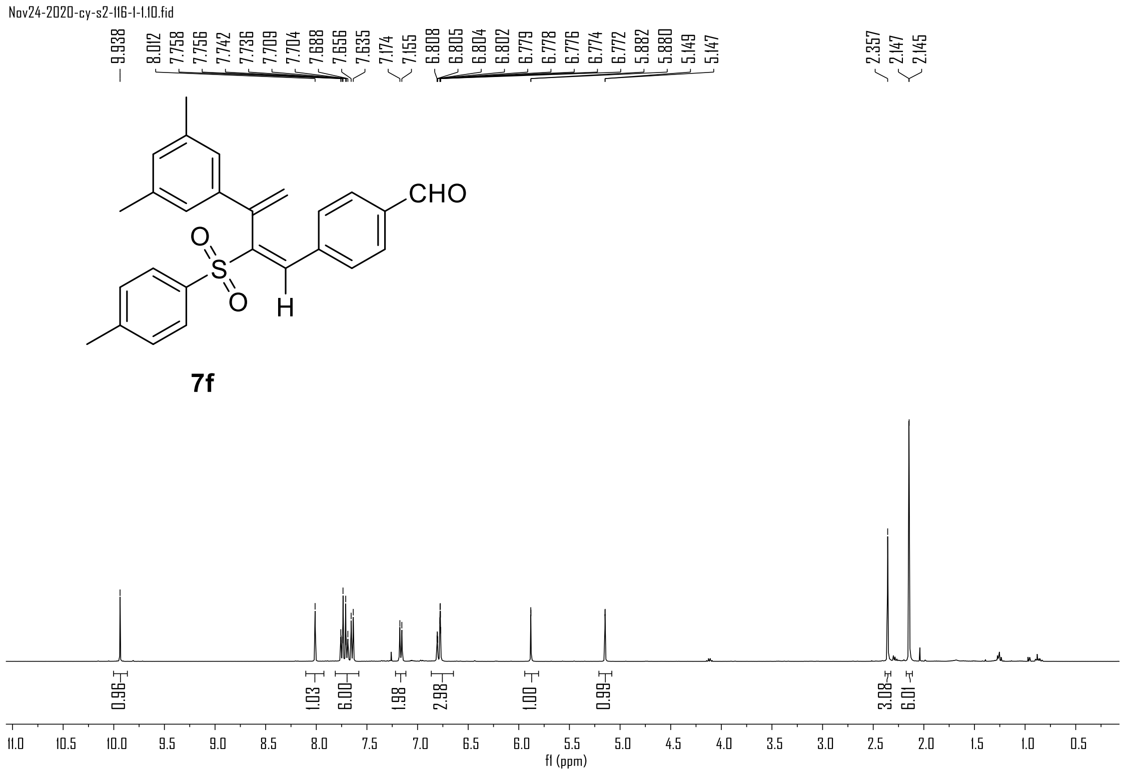

Nov24-2020-cy-s2-116-1-1.10.fid
9.938
8.012 7.758 7.756 7.742 7.736 7.709 7.704 7.688 7.656 7.635
7.174 7.155
6.808 6.805 6.804 6.802 6.779 6.778 6.776 6.774 6.772
5.882 5.880
5.149 5.147
2.357 2.147 2.145
CHO
O
S
O
H
0.96
1.03
6.00
1.98
2.98
1.00
0.99
3.08
6.01
11.0 10.5 10.0 9.5 9.0 8.5 8.0 7.5 7.0 6.5 6.0 5.5 5.0 4.5 4.0 3.5 3.0 2.5 2.0 1.5 1.0 0.5
f1 (ppm)

7f

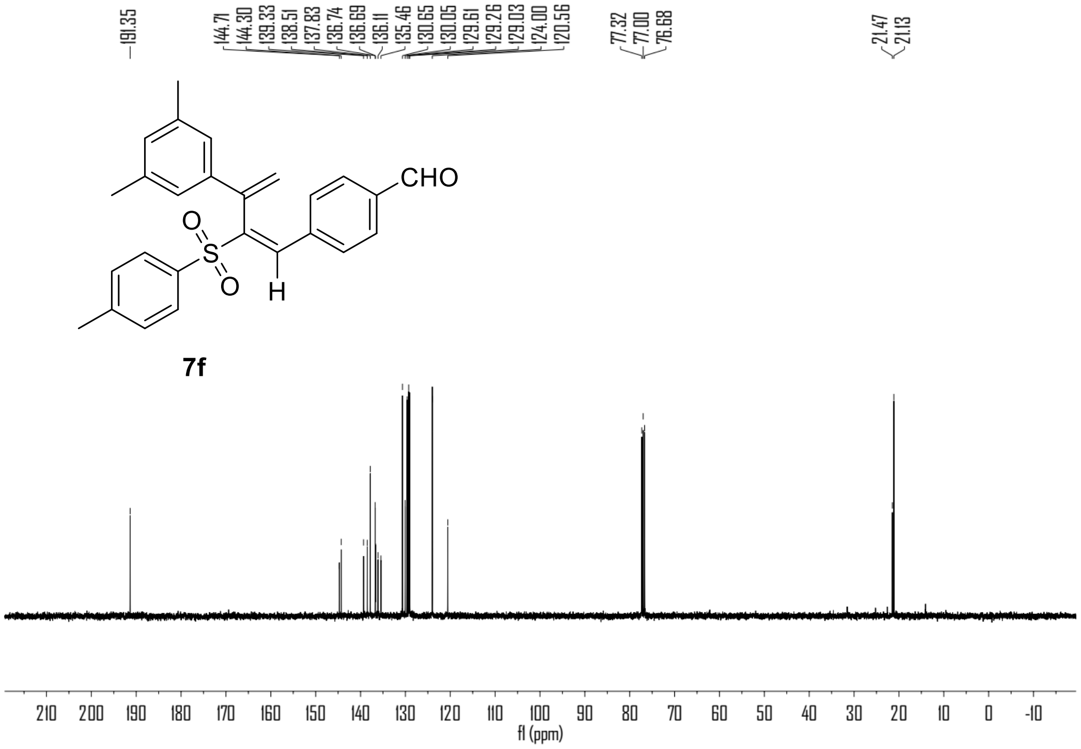

191.35
144.71 144.30 139.33 138.51 137.83 136.74 136.69 136.11 135.46 130.65 130.05 129.61 129.26 129.03 124.00 120.56
77.32 77.00 76.68
21.47 21.13
CHO
O
S
O
H
210 200 190 180 170 160 150 140 130 120 110 100 90 80 70 60 50 40 30 20 10 0 -10
f1 (ppm)

7f

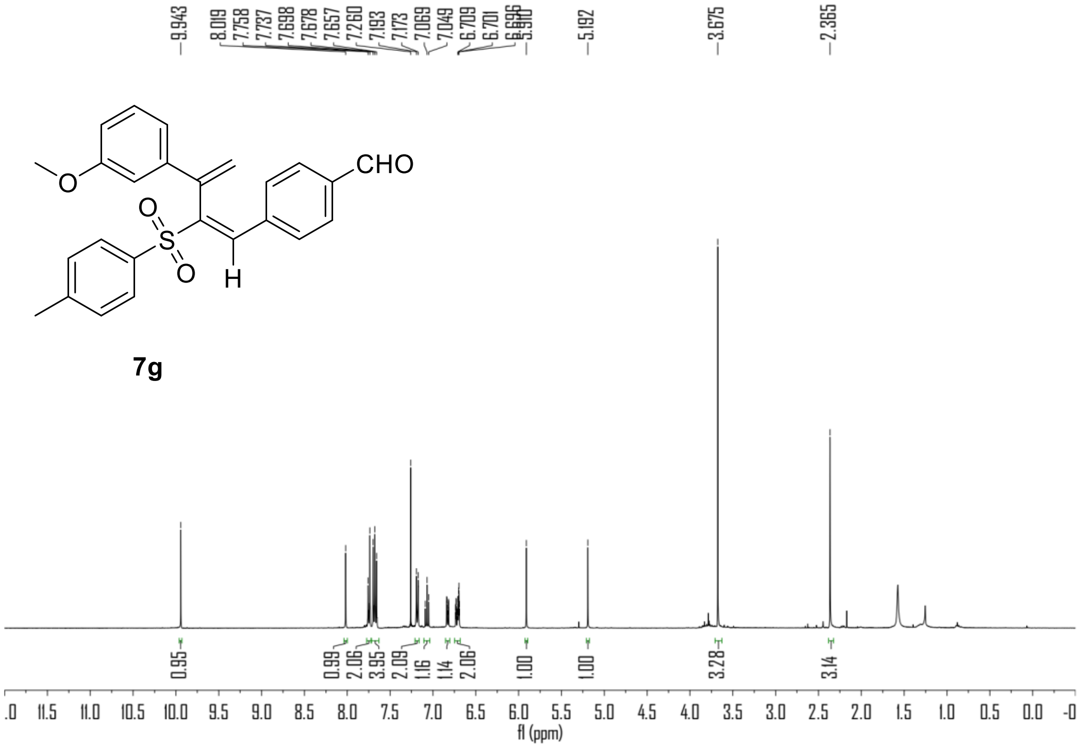
CHO
O
S
H
7g
f1 (ppm)

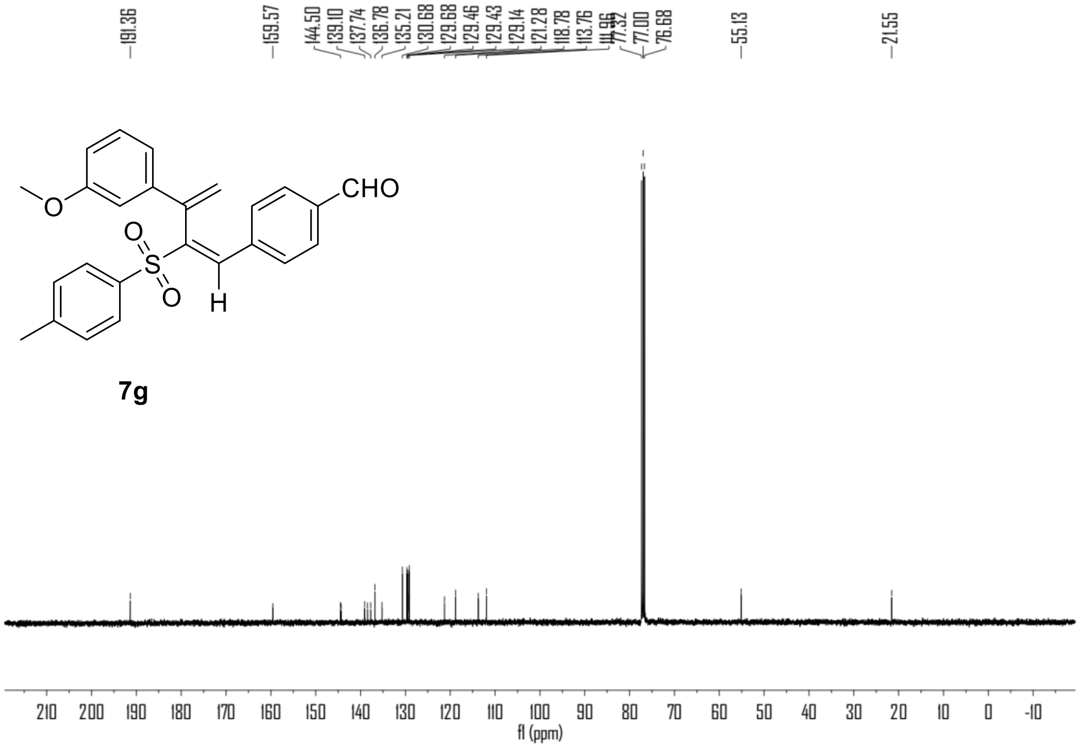
CHO
O
S
H
7g
f1 (ppm)

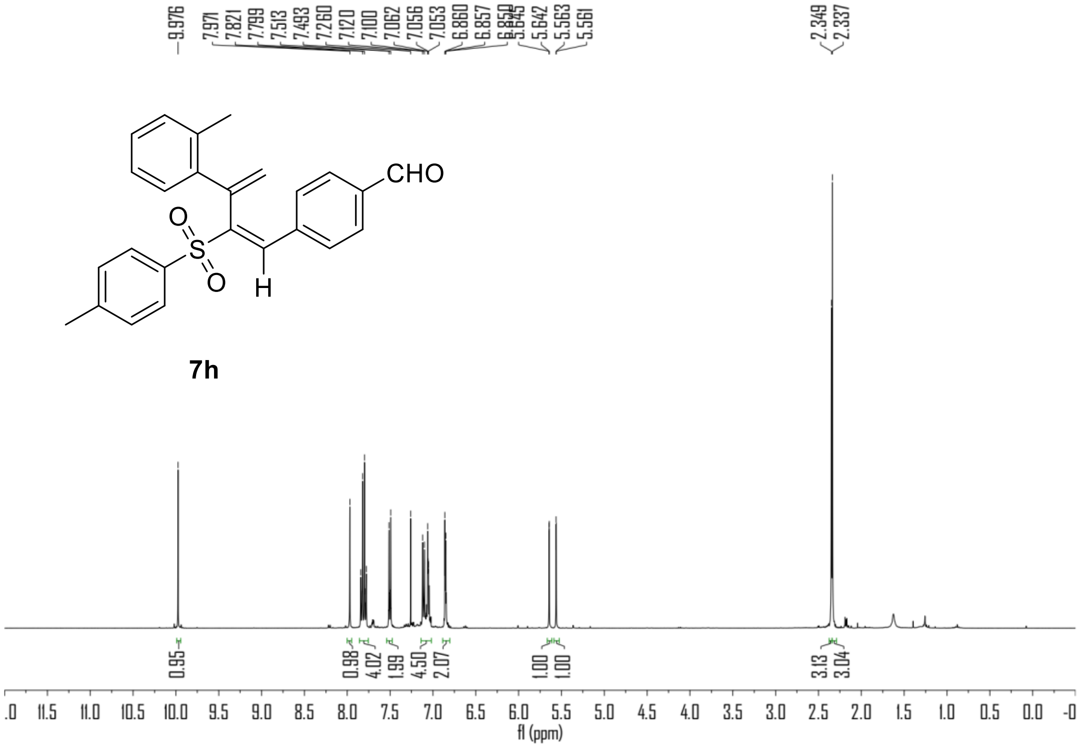
9.976
7.971
7.821
7.799
7.513
7.493
7.260
7.120
7.100
7.062
7.056
7.053
6.860
6.857
5.642
5.563
5.561
2.349
2.337
CHO
O
S
O
H
7h
0.95
0.98
4.02
1.99
4.50
2.07
1.00
1.00
3.13
3.04
fl (ppm)

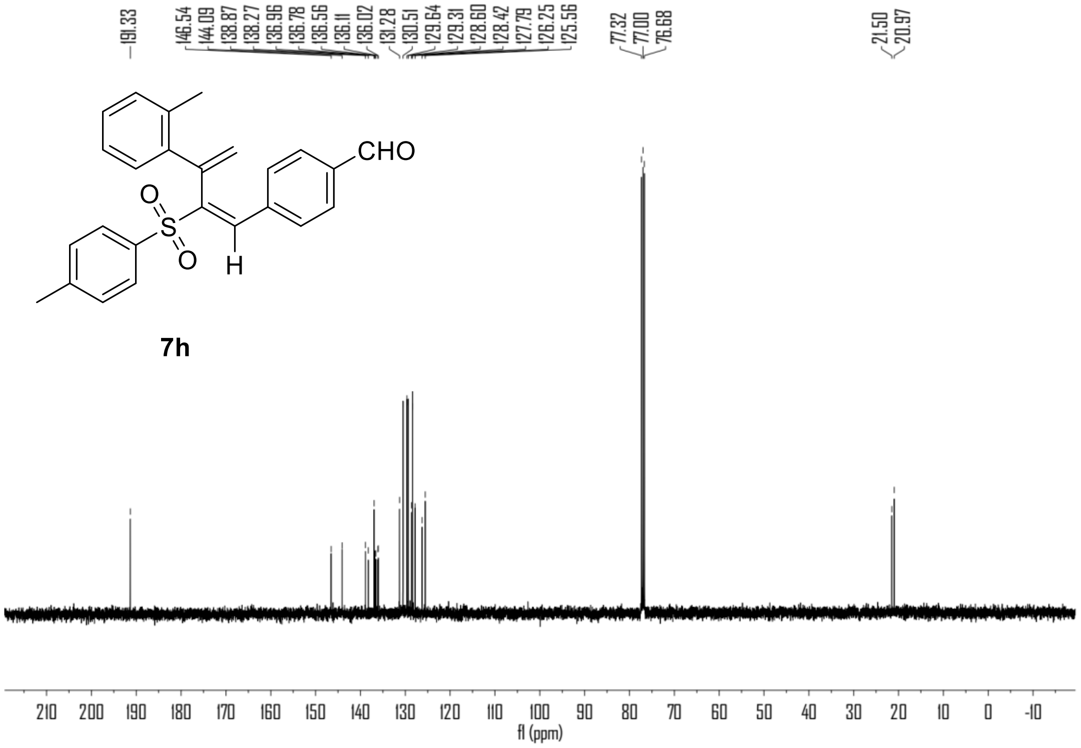
191.33
146.54
144.09
138.87
138.27
136.96
136.78
136.56
136.11
136.02
131.28
130.51
129.64
129.31
128.60
128.42
127.79
126.25
125.56
77.32
77.00
76.68
21.50
20.97
CHO
O
S
O
H
7h
fl (ppm)

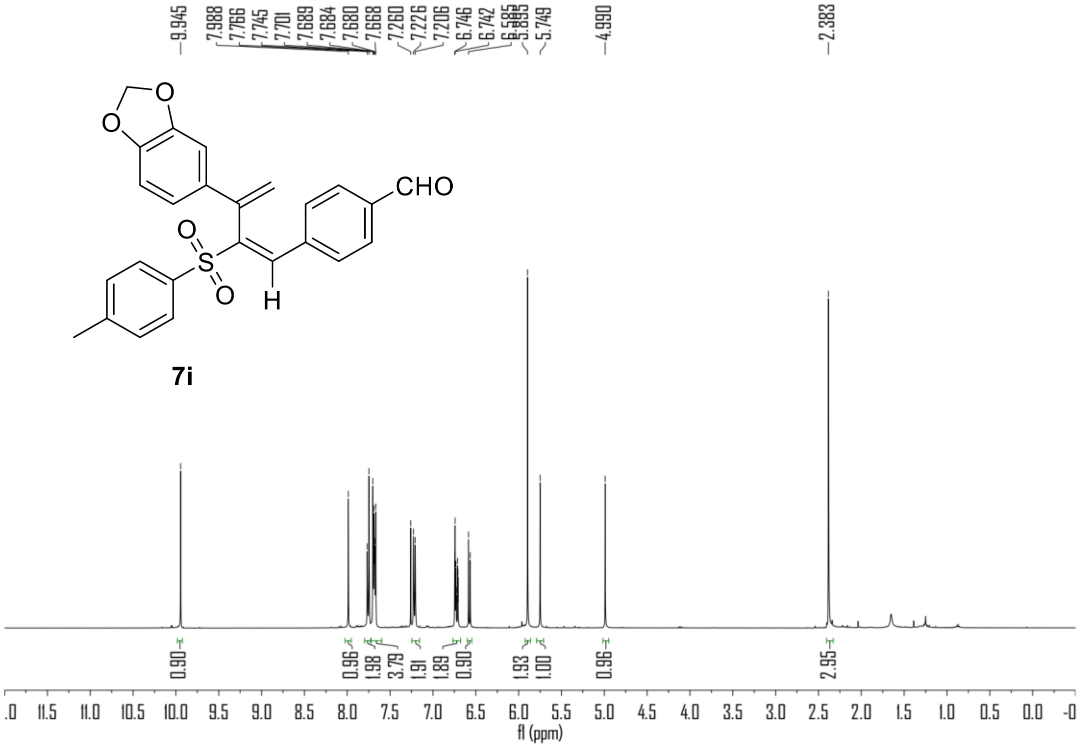
9.945
7.988
7.766
7.745
7.701
7.689
7.684
7.680
7.668
7.260
7.226
7.206
6.746
6.742
6.585
5.749
4.990
2.383
CHO
O
O
S
O
O
H
7i
0.90
0.96
1.98
3.79
1.91
1.89
0.90
1.93
1.00
0.96
2.95
fl (ppm)

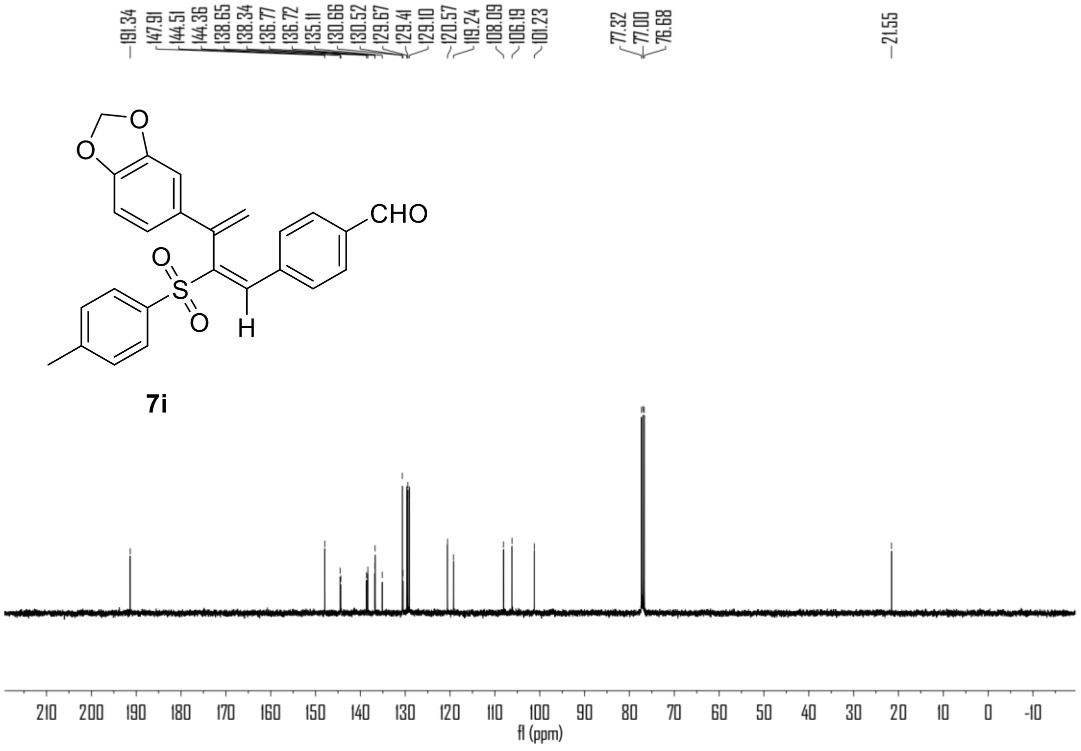
191.34
147.91
144.51
144.36
138.65
138.34
136.77
136.72
135.11
130.66
130.52
129.67
129.41
129.10
120.57
119.24
108.09
106.19
101.23
77.32
77.00
76.68
21.55
CHO
O
O
S
O
O
H
7i
fl (ppm)

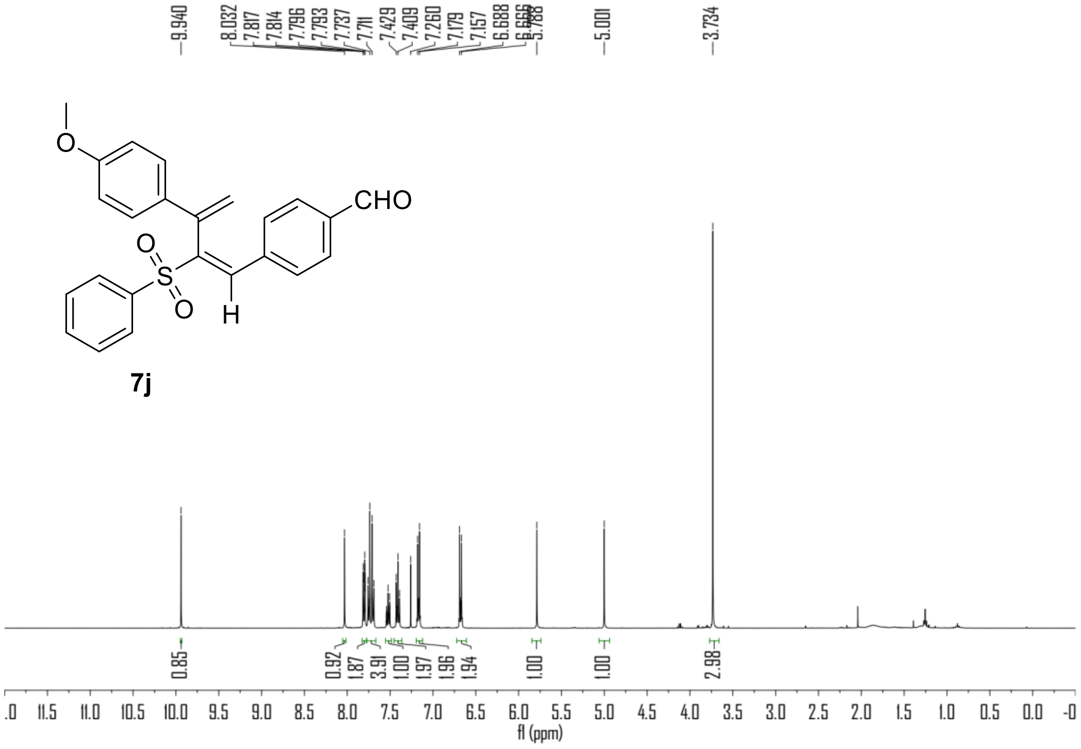
9.940
8.032
7.817
7.814
7.796
7.793
7.737
7.711
7.429
7.409
7.260
7.179
7.157
6.688
5.001
3.734
CHO
O
S
H
7j
0.85
0.92
1.87
3.91
1.00
1.97
1.96
1.94
1.00
1.00
2.98
fl (ppm)

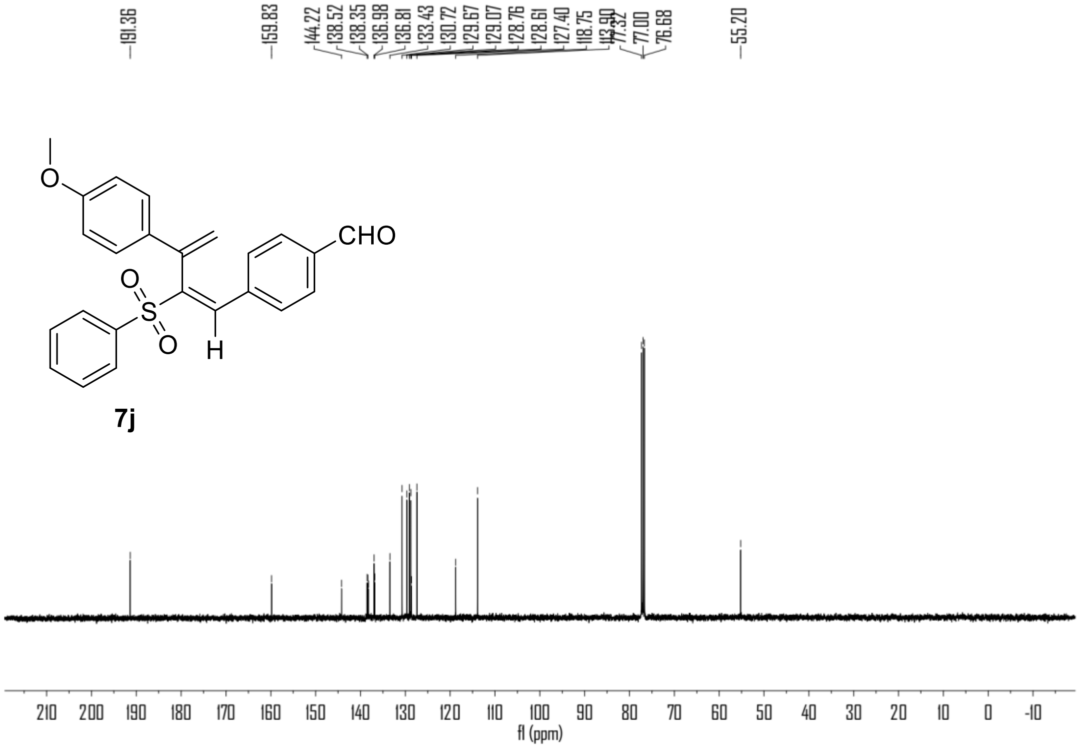
191.36
159.83
144.22
138.52
138.35
136.98
136.81
133.43
130.72
129.67
129.07
128.76
128.61
127.40
118.75
77.00
76.68
55.20
CHO
O
S
H
7j
fl (ppm)

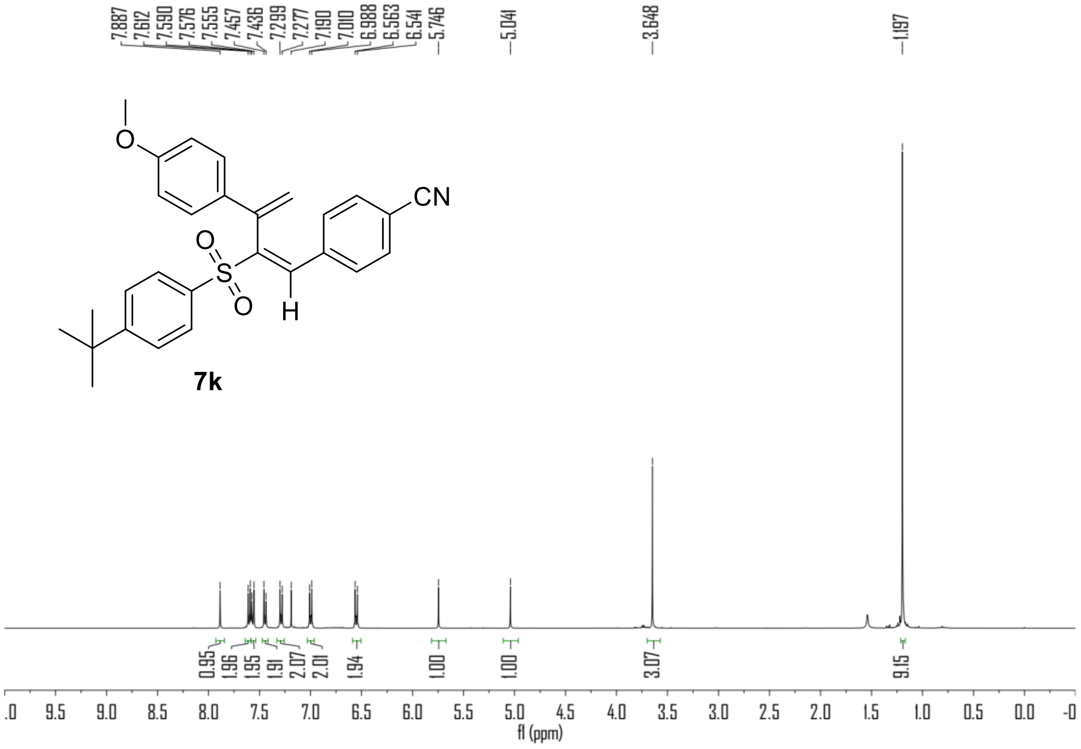
7.887
7.612
7.590
7.576
7.555
7.457
7.436
7.299
7.277
7.190
7.010
6.988
6.563
6.541
5.746
5.041
3.648
1.197
CN
O
S
H
7k
0.95
1.96
1.95
1.91
2.07
2.01
1.94
1.00
1.00
3.07
9.15
fl (ppm)

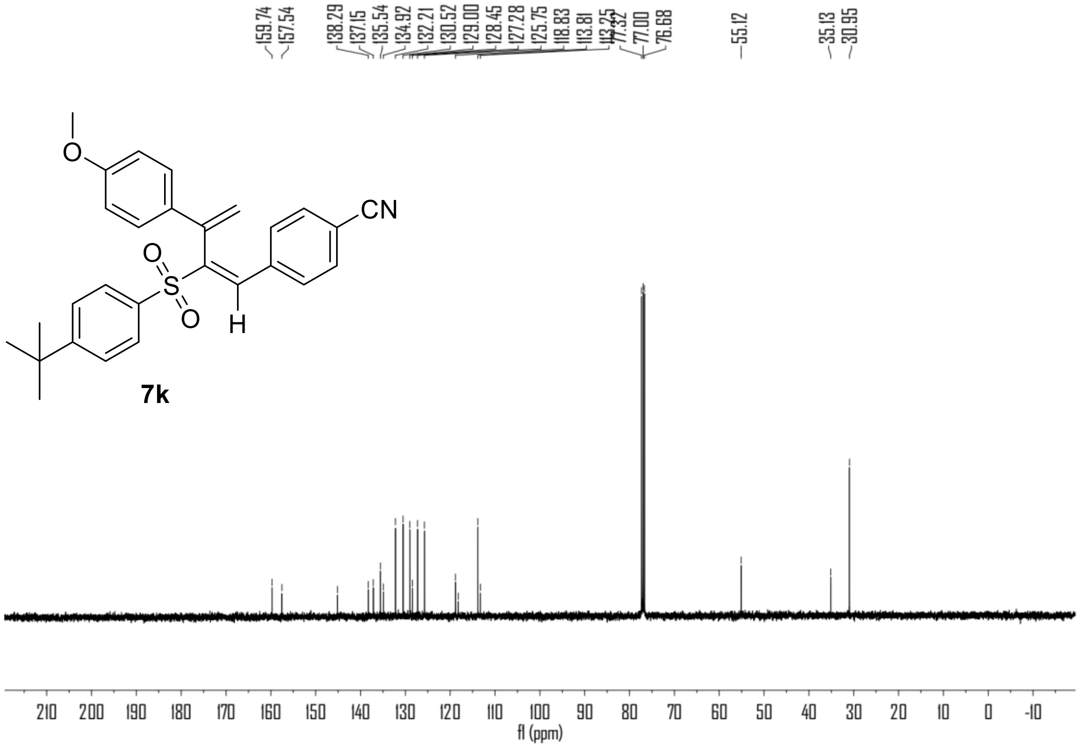
159.74
157.54
138.29
137.15
135.54
134.92
132.21
130.52
129.00
128.45
127.28
125.75
118.83
113.81
113.25
77.32
77.00
76.68
55.12
35.13
30.95
CN
O
S
H
7k
fl (ppm)

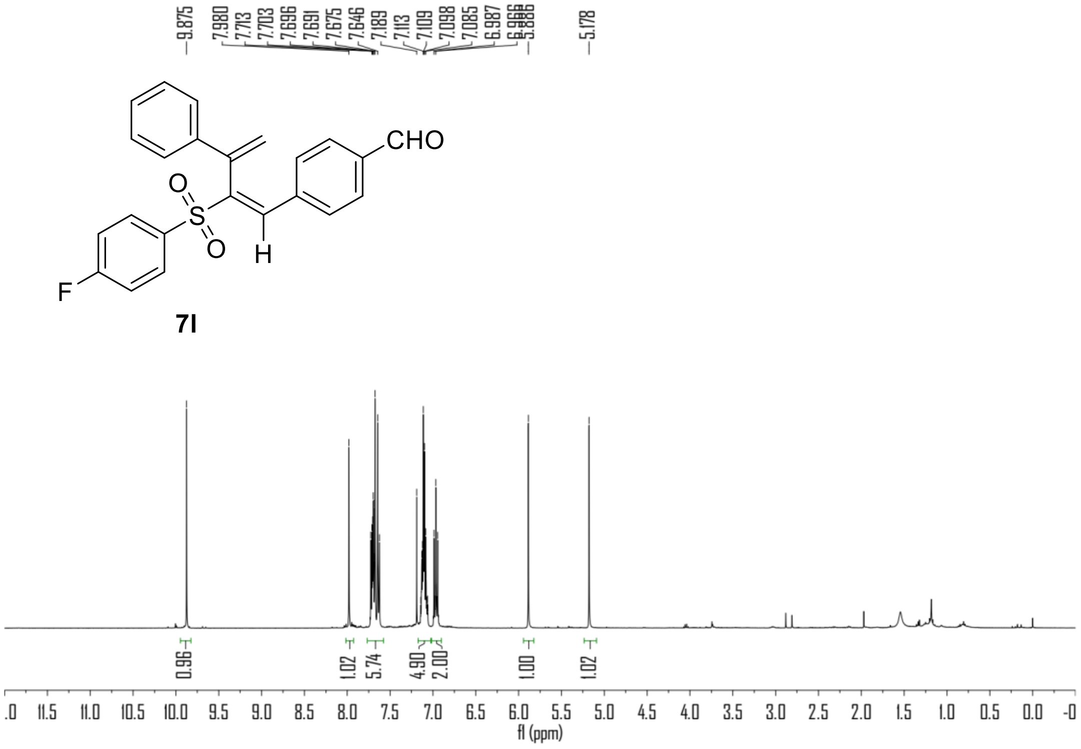
9.875
7.980
7.713
7.703
7.696
7.691
7.675
7.646
7.189
7.113
7.109
7.098
7.085
6.987
5.178
CHO
O
S
O
H
F
7l
0.96
1.02
5.74
4.90
2.00
1.00
1.02
fl (ppm)

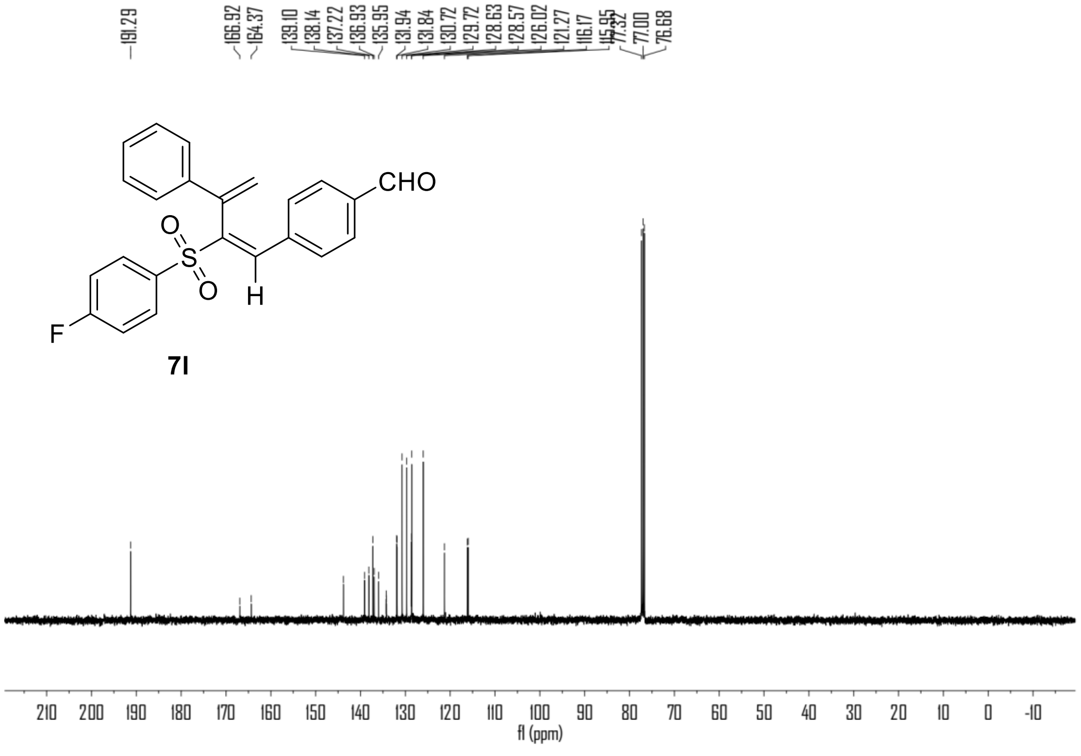
191.29
166.92
164.37
139.10
138.14
137.22
136.93
135.95
131.94
131.84
130.72
129.72
128.63
128.57
126.02
121.27
116.17
77.32
77.00
76.68
CHO
O
S
O
H
F
7l
fl (ppm)

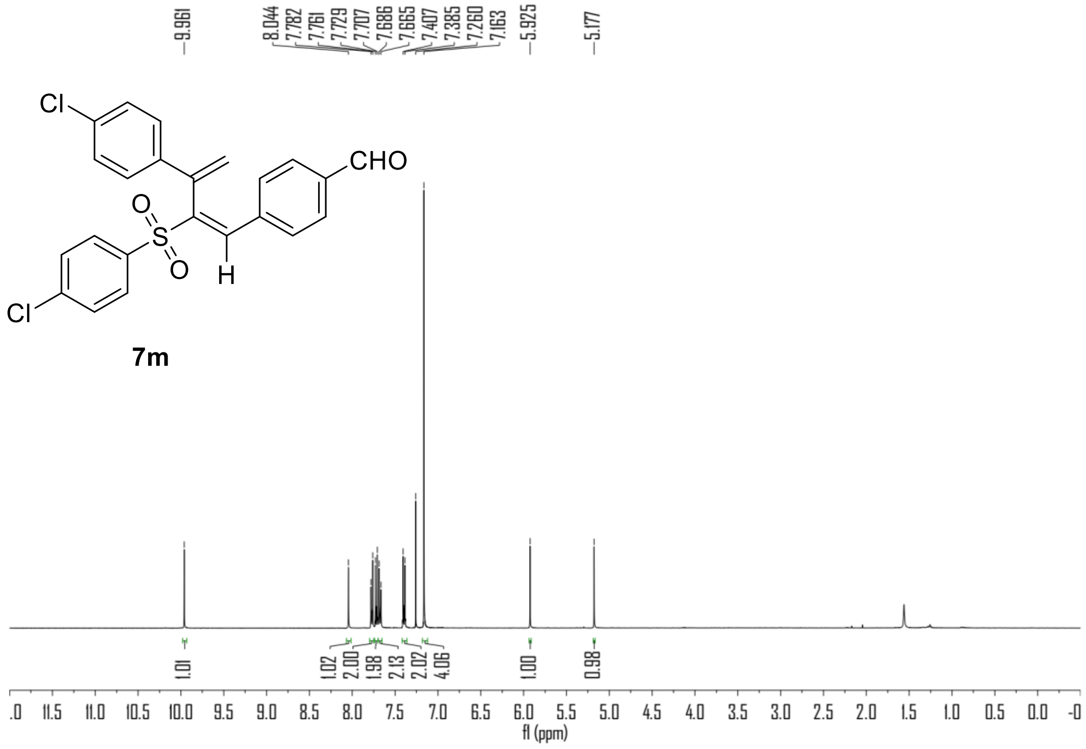
9.961
8.044
7.782
7.761
7.729
7.707
7.686
7.665
7.407
7.385
7.260
7.163
5.925
5.177
Cl
CHO
O
S
O
H
Cl
7m
1.01
1.02
2.00
1.98
2.13
2.02
4.06
1.00
0.98
fl (ppm)

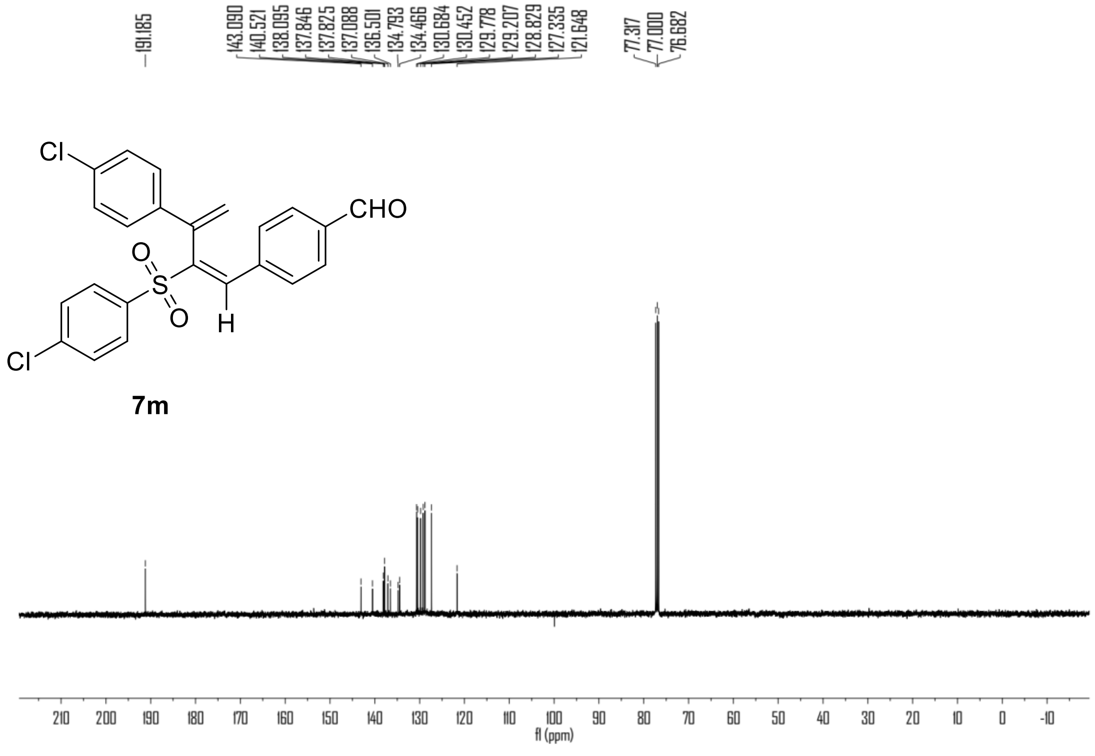
191.185
143.090
140.521
138.095
137.846
137.825
137.088
136.501
134.793
134.466
130.684
130.452
129.778
129.207
128.829
127.335
121.648
77.317
77.000
76.682
Cl
CHO
O
S
O
H
Cl
7m
fl (ppm)

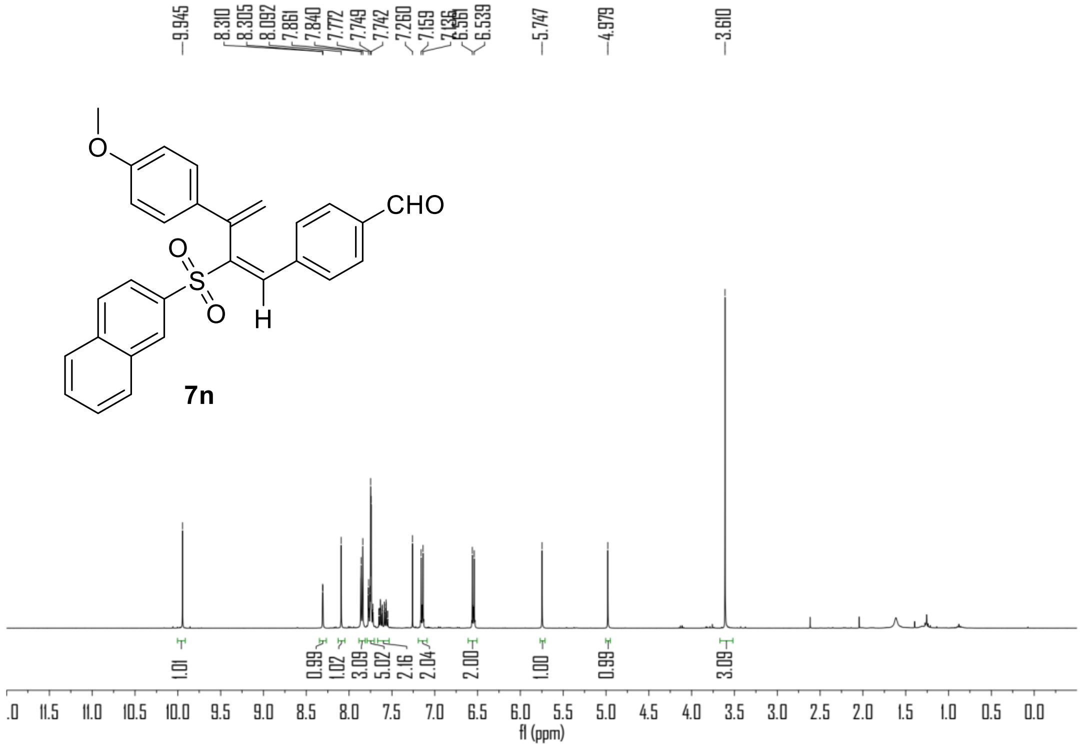

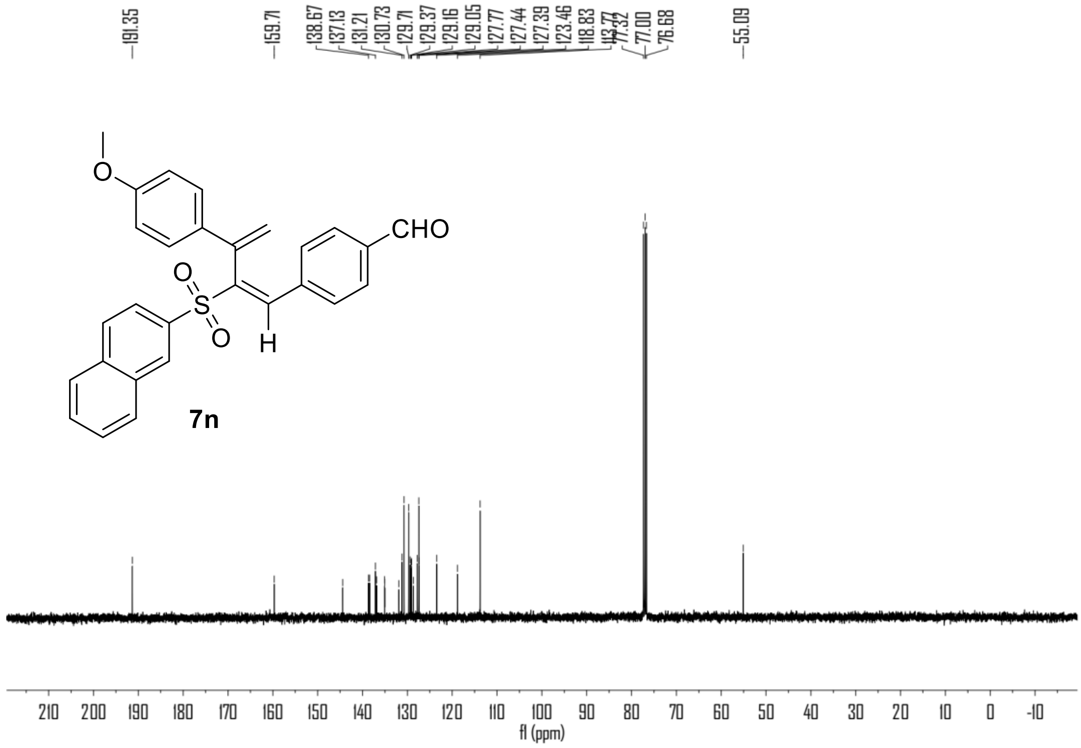

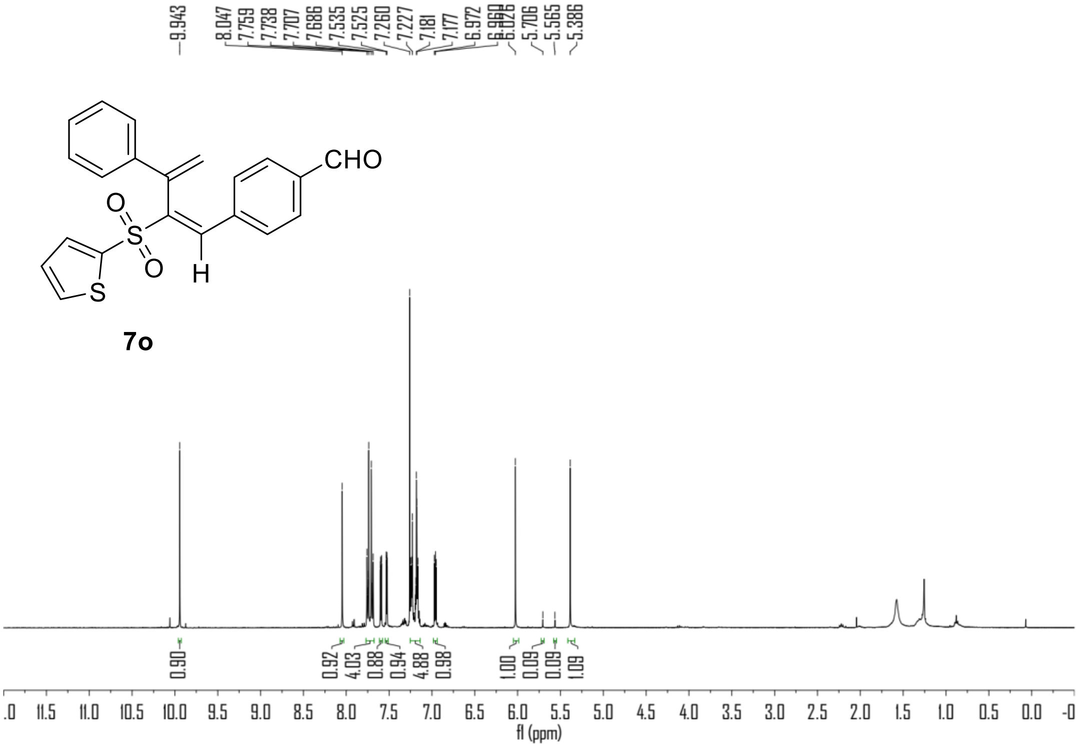

9.943
8.047
7.759
7.738
7.707
7.686
7.535
7.525
7.260
7.227
7.181
7.177
6.972
[illegible]
5.706
5.565
5.386
CHO
O
S
O
S
H
7o
0.90
0.92
4.03
0.88
0.94
4.88
0.98
1.00
0.09
0.09
1.09
f1 (ppm)

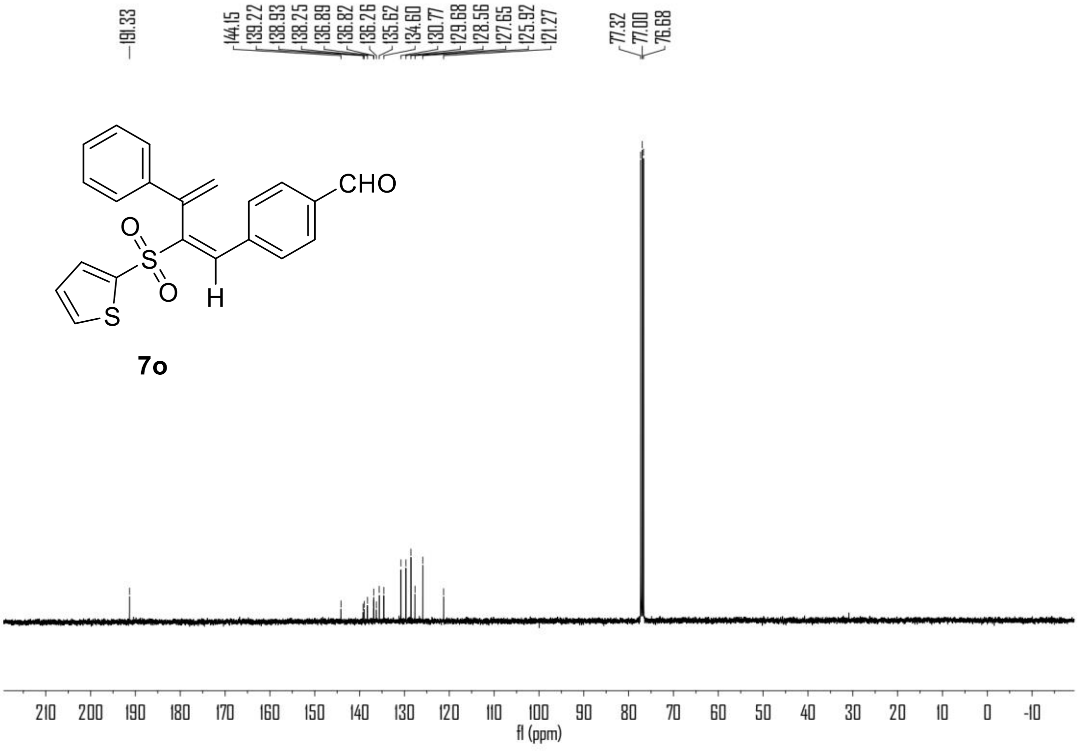

191.33
144.15
139.22
138.93
138.25
136.89
136.82
136.26
135.62
134.60
130.77
129.68
128.56
127.65
125.92
121.27
77.32
77.00
76.68
CHO
O
S
O
S
H
7o
f1 (ppm)

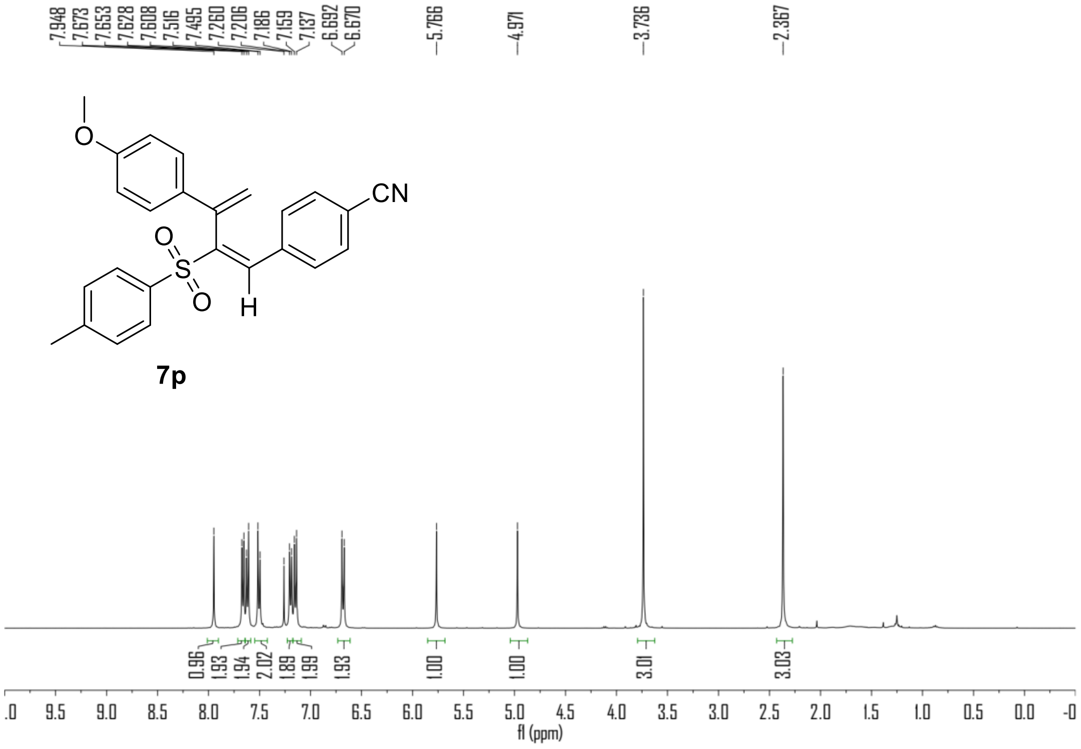
7.948
7.673
7.653
7.628
7.608
7.516
7.495
7.260
7.206
7.186
7.159
7.137
6.692
6.670
5.766
4.971
3.736
2.367
CN
O
S
O
O
H
7p
0.96
1.93
1.94
2.02
1.89
1.99
1.93
1.00
1.00
3.01
3.03
f1 (ppm)

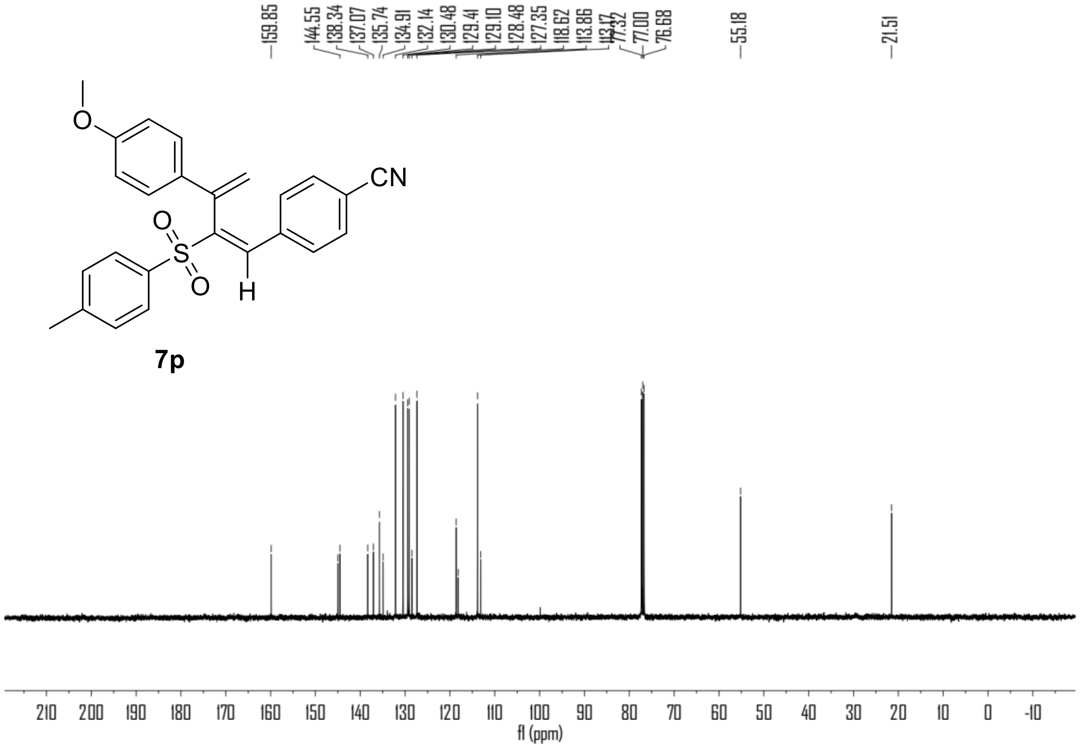
159.85
144.55
138.34
137.07
135.74
134.91
132.14
130.48
129.41
129.10
128.48
127.35
118.62
113.86
77.32
77.00
76.68
55.18
21.51
CN
O
S
O
O
H
7p
f1 (ppm)

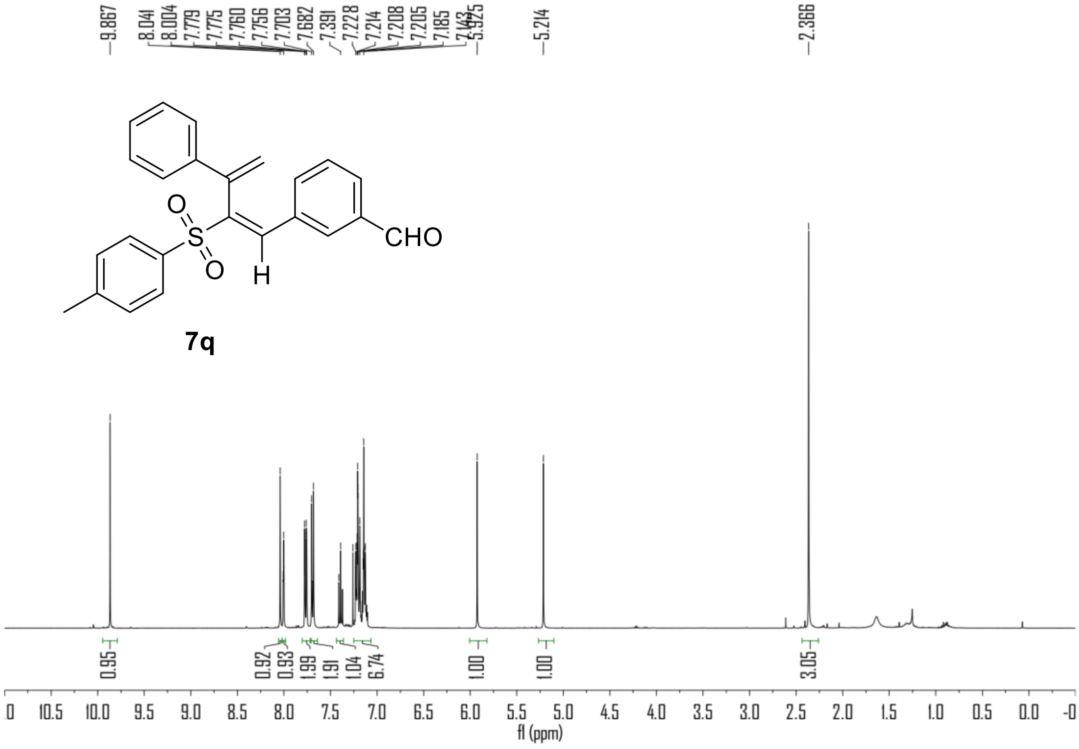
9.867
8.044
8.004
7.779
7.775
7.760
7.756
7.703
7.682
7.391
7.228
7.214
7.208
7.205
7.185
5.925
5.214
2.366
CHO
H
O
S
7q
0.95
0.92
0.93
1.99
1.91
1.04
6.74
1.00
1.00
3.05
fl (ppm)

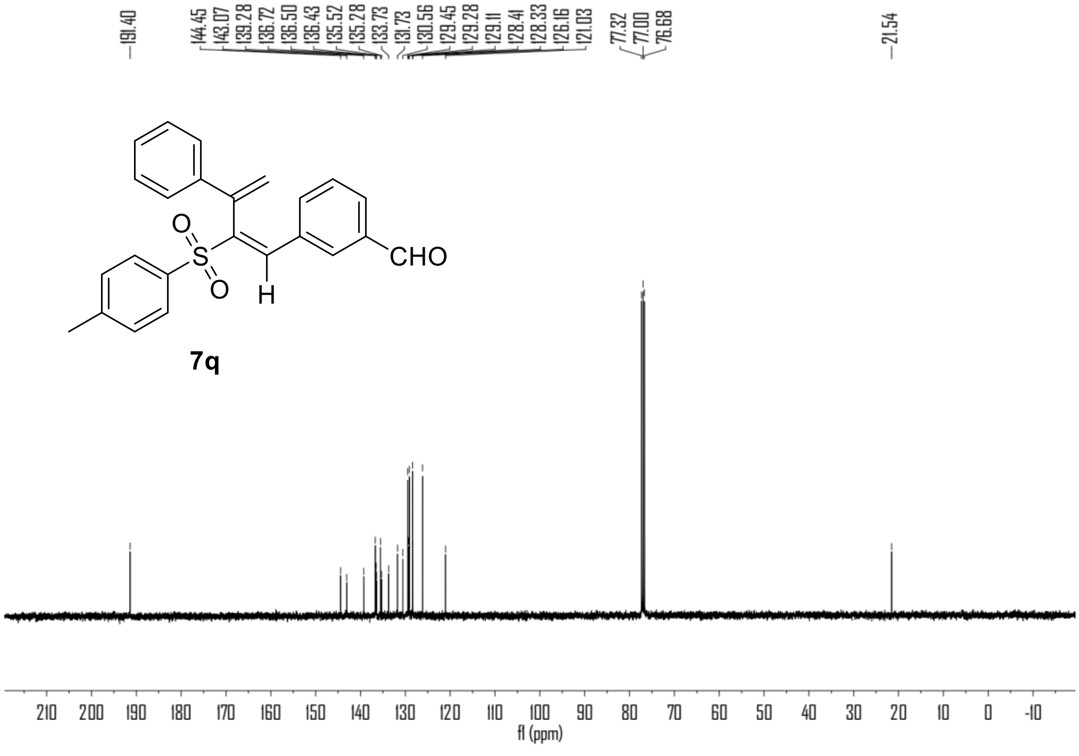
191.40
144.45
143.07
139.28
136.72
136.50
136.43
135.52
135.28
133.73
131.73
130.56
129.45
129.28
129.11
128.41
128.33
126.16
121.03
77.32
77.00
76.68
21.54
CHO
H
O
S
7q
fl (ppm)

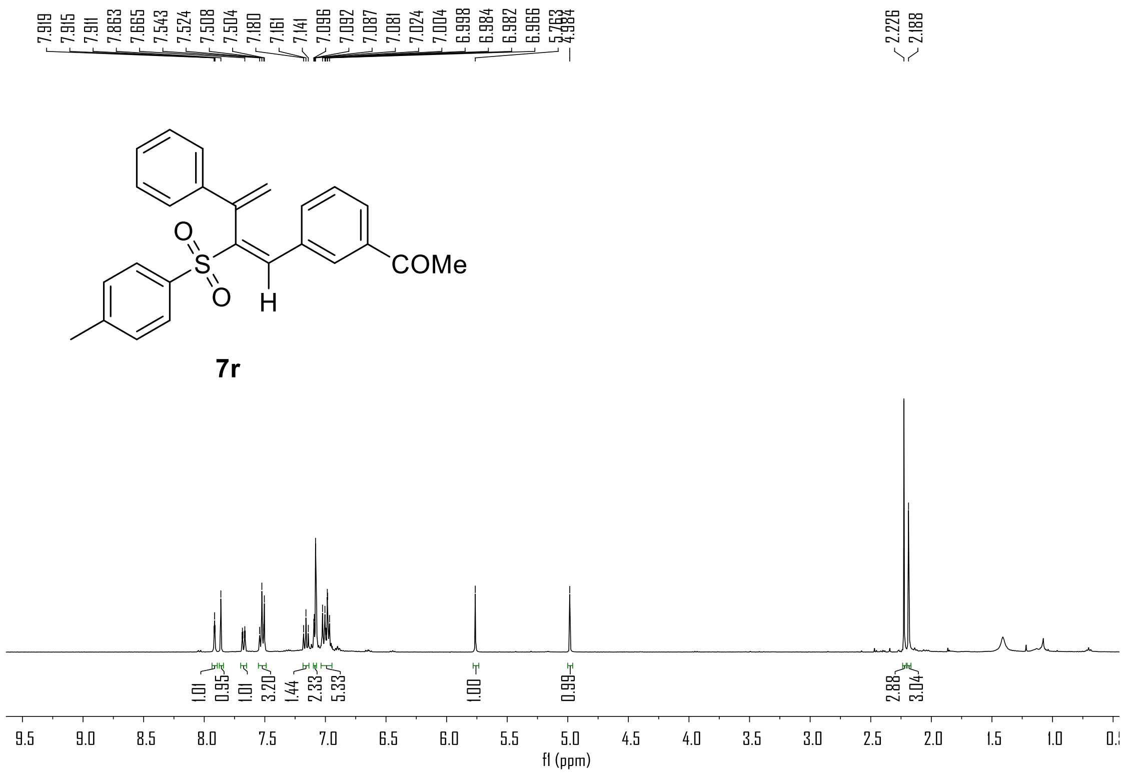
7.919
7.915
7.911
7.863
7.665
7.543
7.524
7.508
7.504
7.180
7.161
7.141
7.096
7.092
7.087
7.081
7.024
7.004
6.998
6.984
6.982
6.966
5.763
4.984
2.226
2.188
COMe
H
7r
1.01
0.95
1.01
3.20
1.44
2.33
5.33
1.00
0.99
2.88
3.04
fl (ppm)

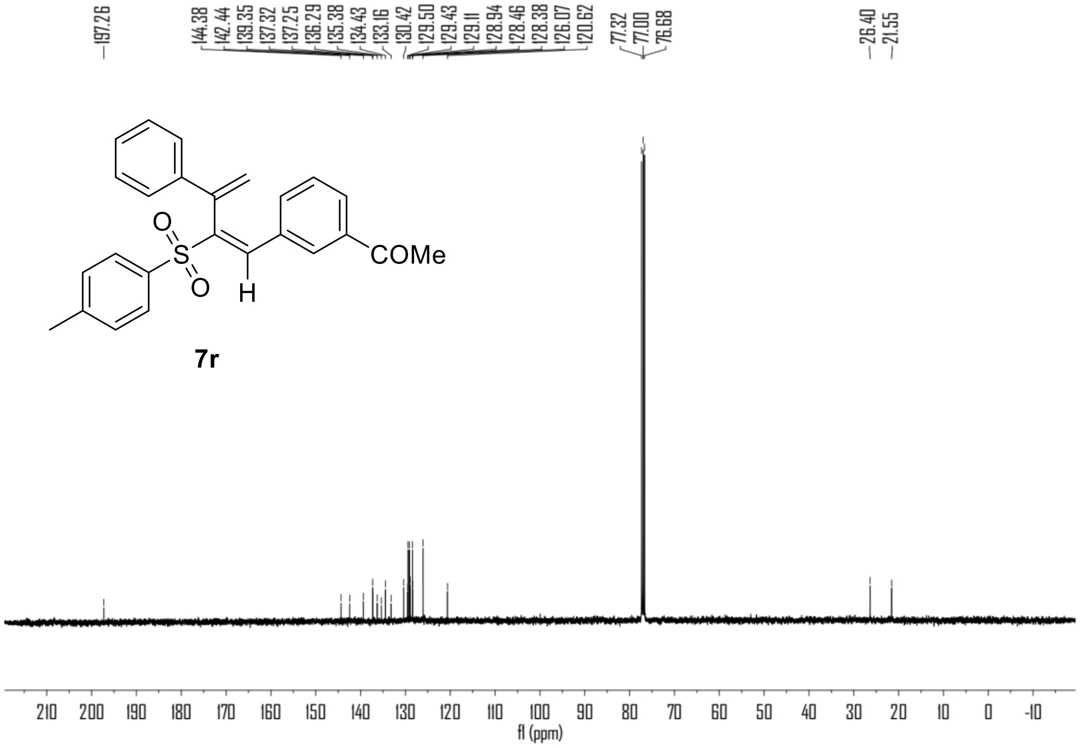
197.26
144.38
142.44
139.35
137.32
137.25
136.29
135.38
134.43
133.16
130.42
129.50
129.43
129.11
128.94
128.46
128.38
126.07
120.62
77.32
77.00
76.68
26.40
21.55
COMe
H
7r
fl (ppm)

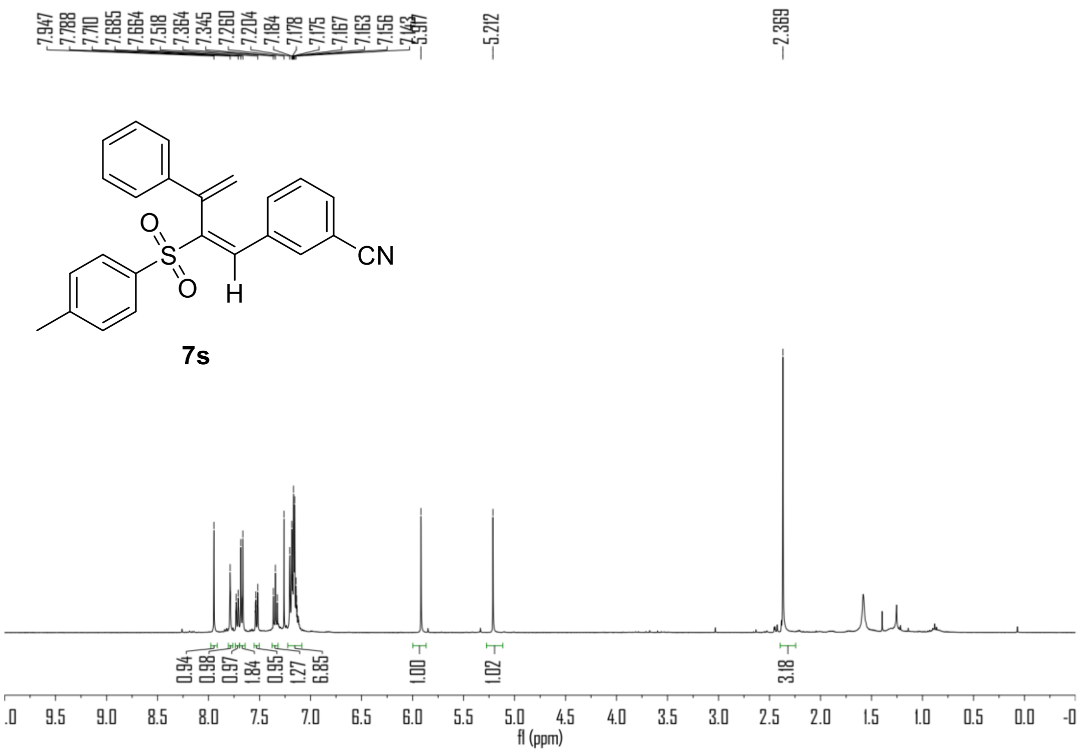
7.947
7.788
7.710
7.685
7.664
7.518
7.364
7.345
7.260
7.204
7.184
7.178
7.175
7.167
7.163
7.156
7.143
5.917
5.212
2.369
CN
H
O
S
O
7s
0.94
0.98
0.97
1.84
0.95
1.27
6.85
1.00
1.02
3.18
fl (ppm)

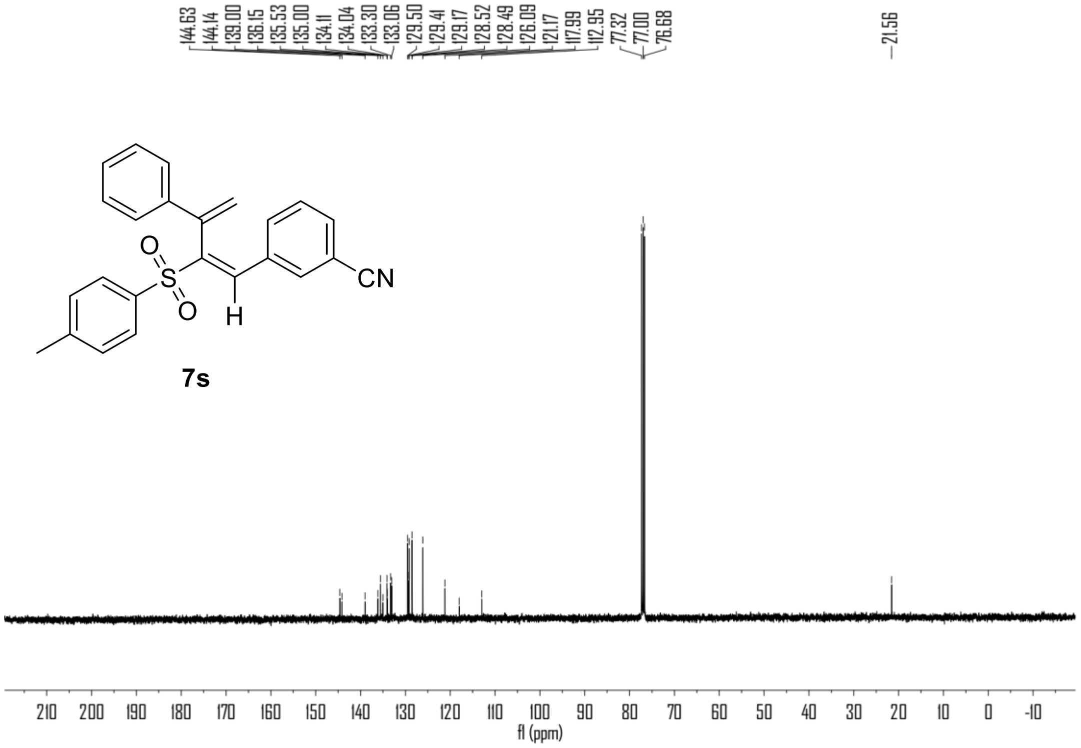
144.63
144.14
139.00
136.15
135.53
135.00
134.11
134.04
133.30
133.06
129.50
129.41
129.17
128.52
128.49
126.09
121.17
117.99
112.95
77.32
77.00
76.68
21.56
CN
H
O
S
O
7s
fl (ppm)

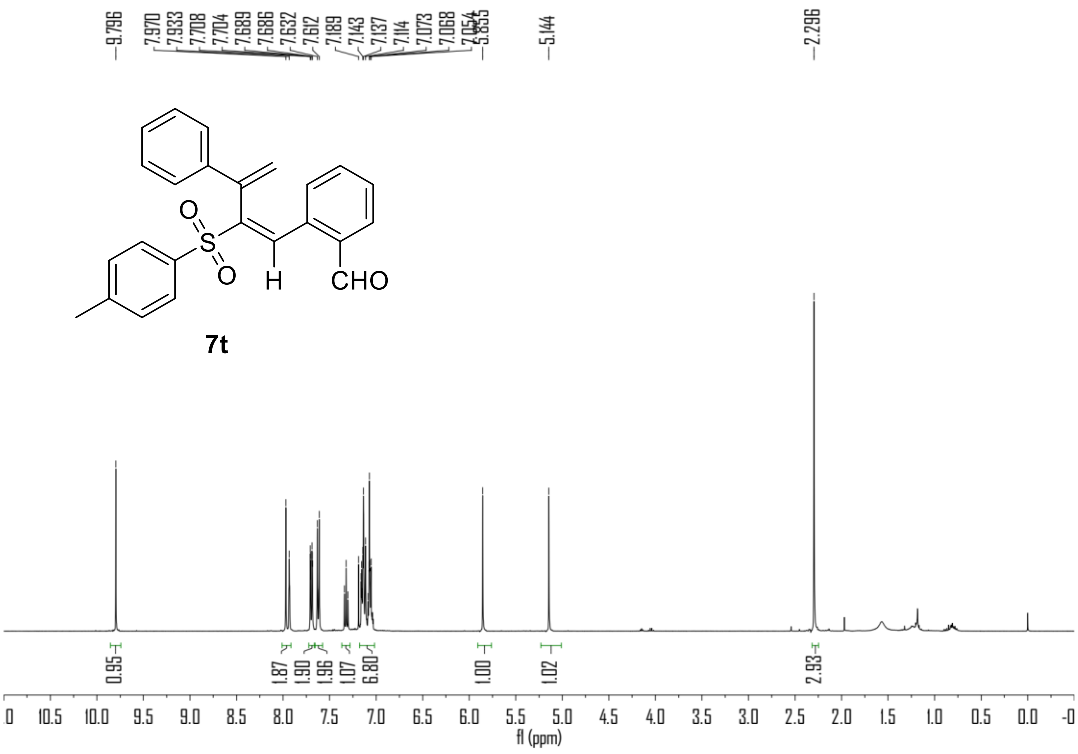
9.796
7.970
7.933
7.708
7.704
7.689
7.686
7.632
7.612
7.189
7.143
7.137
7.114
7.073
7.068
7.054
5.853
5.144
2.296
O
S
O
H
CHO
7t
0.95
1.87
1.90
1.96
1.07
6.80
1.00
1.02
2.93
10.5
10.0
9.5
9.0
8.5
8.0
7.5
7.0
6.5
6.0
5.5
5.0
4.5
4.0
3.5
3.0
2.5
2.0
1.5
1.0
0.5
0.0
fl (ppm)

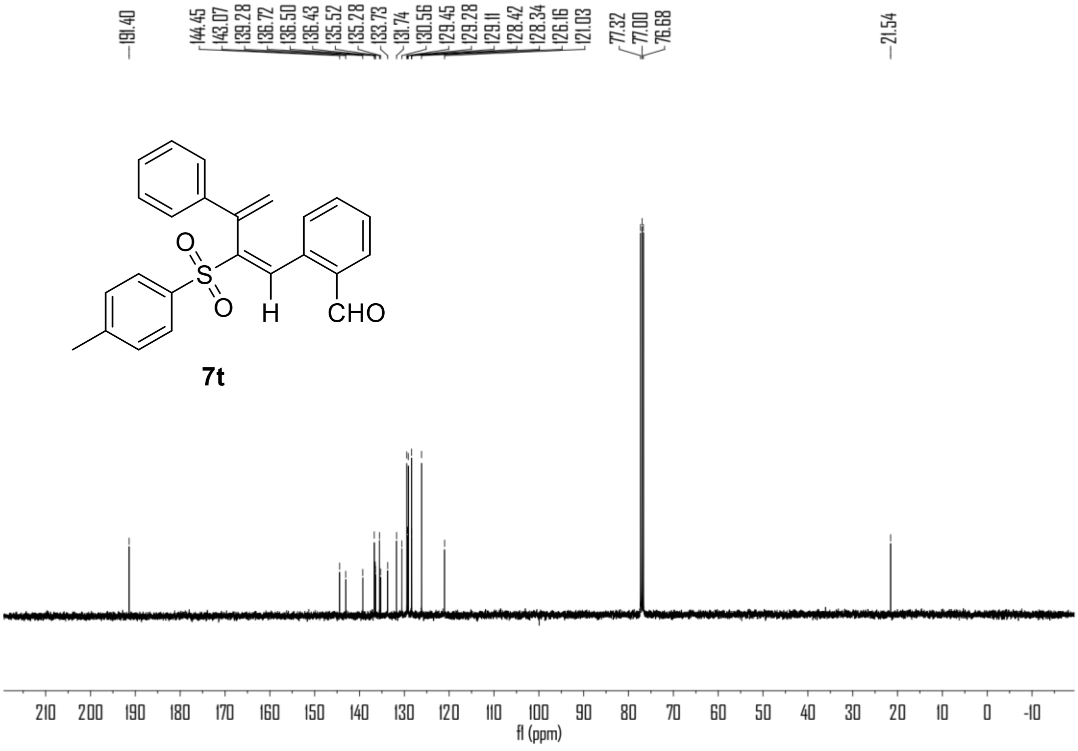
191.40
144.45
143.07
139.28
136.72
136.50
136.43
135.52
135.28
133.73
131.74
130.56
129.45
129.28
129.11
128.42
128.34
126.16
121.03
77.32
77.00
76.68
21.54
O
S
O
H
CHO
7t
210
200
190
180
170
160
150
140
130
120
110
100
90
80
70
60
50
40
30
20
10
0
-10
fl (ppm)

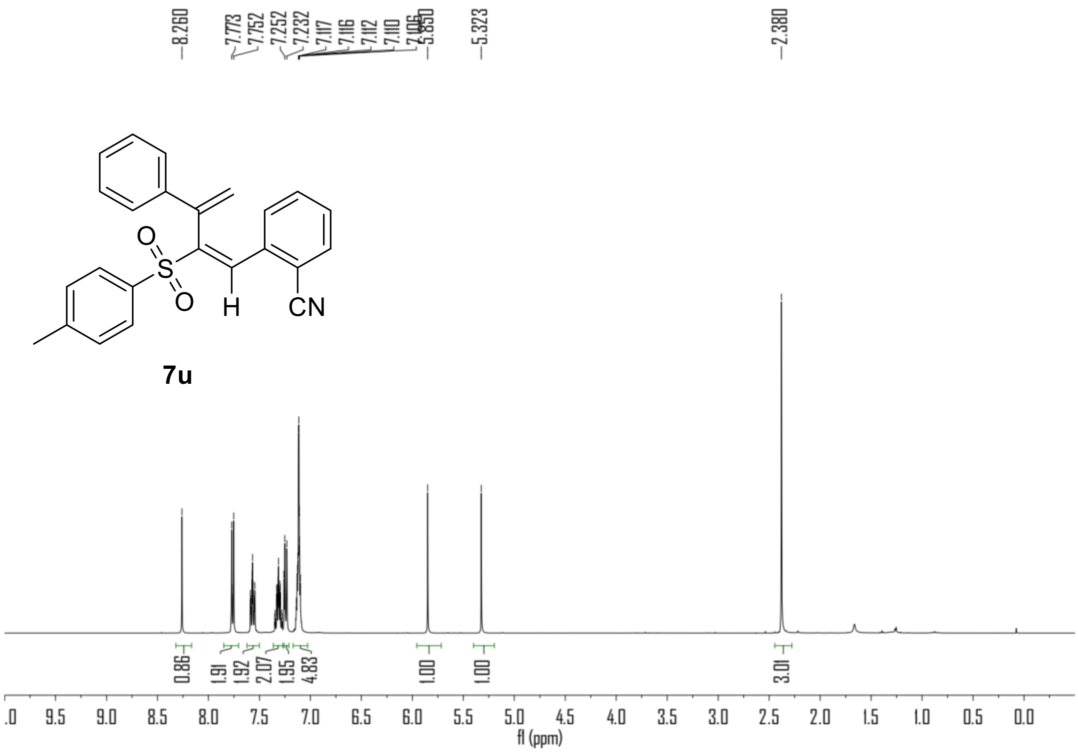

7u

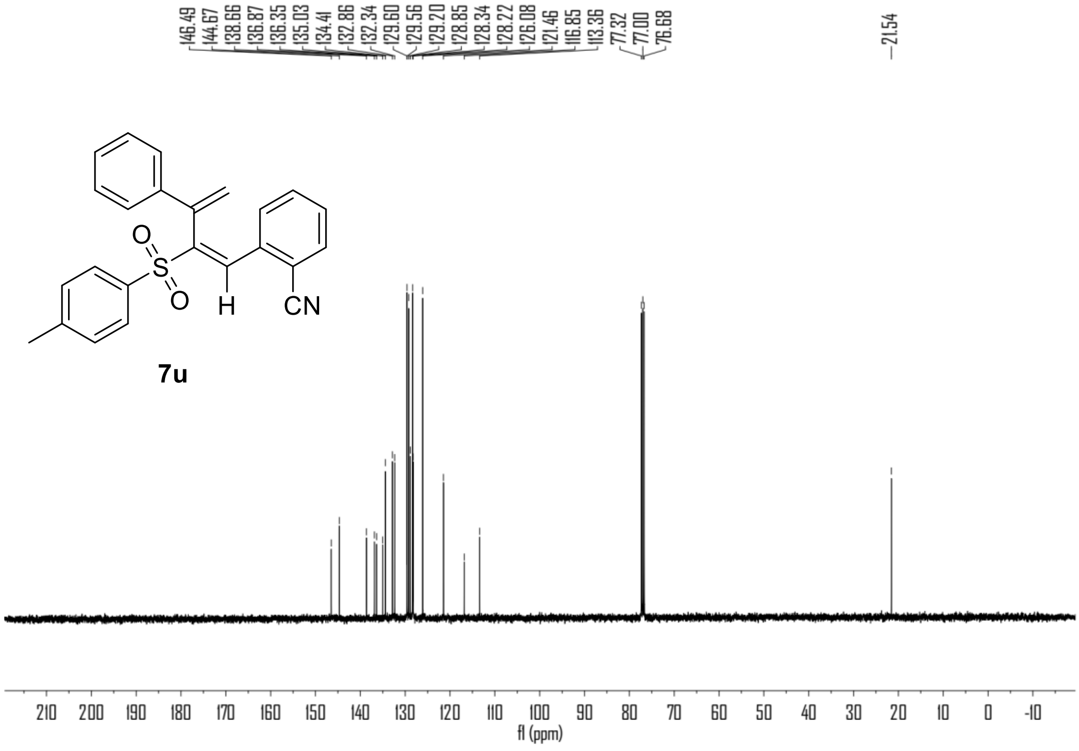

7u

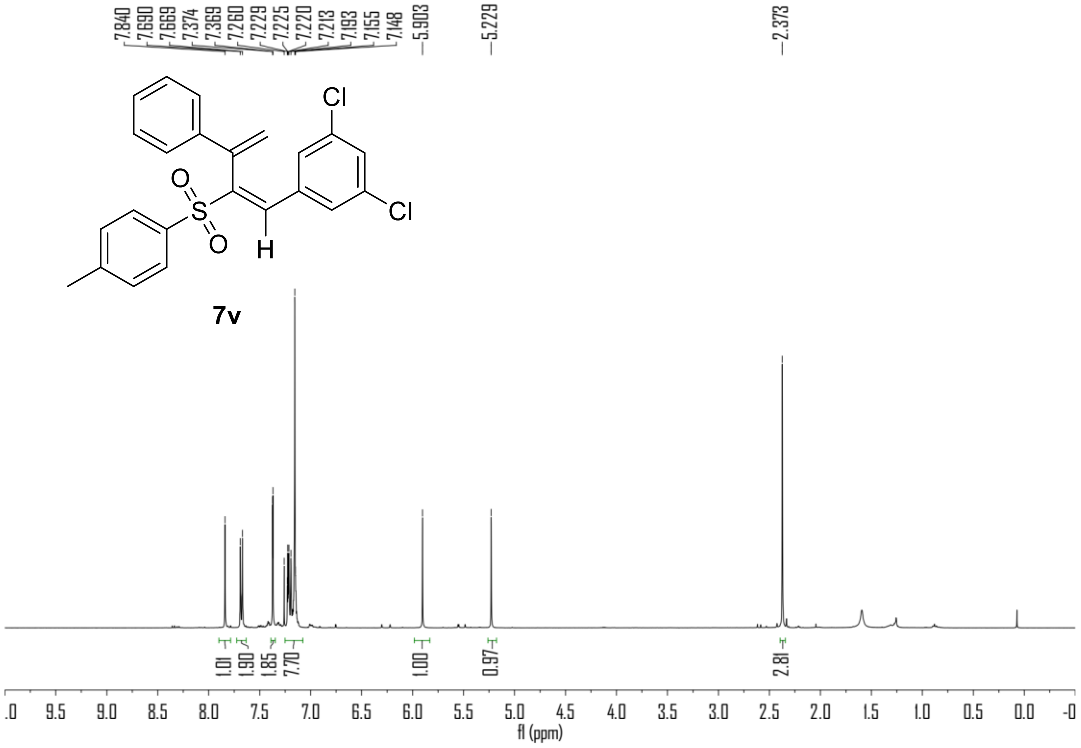
7.840
7.690
7.669
7.374
7.369
7.260
7.229
7.225
7.220
7.213
7.193
7.155
7.148
5.903
5.229
2.373
Cl
Cl
O
S
O
H
7v
1.01
1.90
1.85
7.70
1.00
0.97
2.81
fl (ppm)

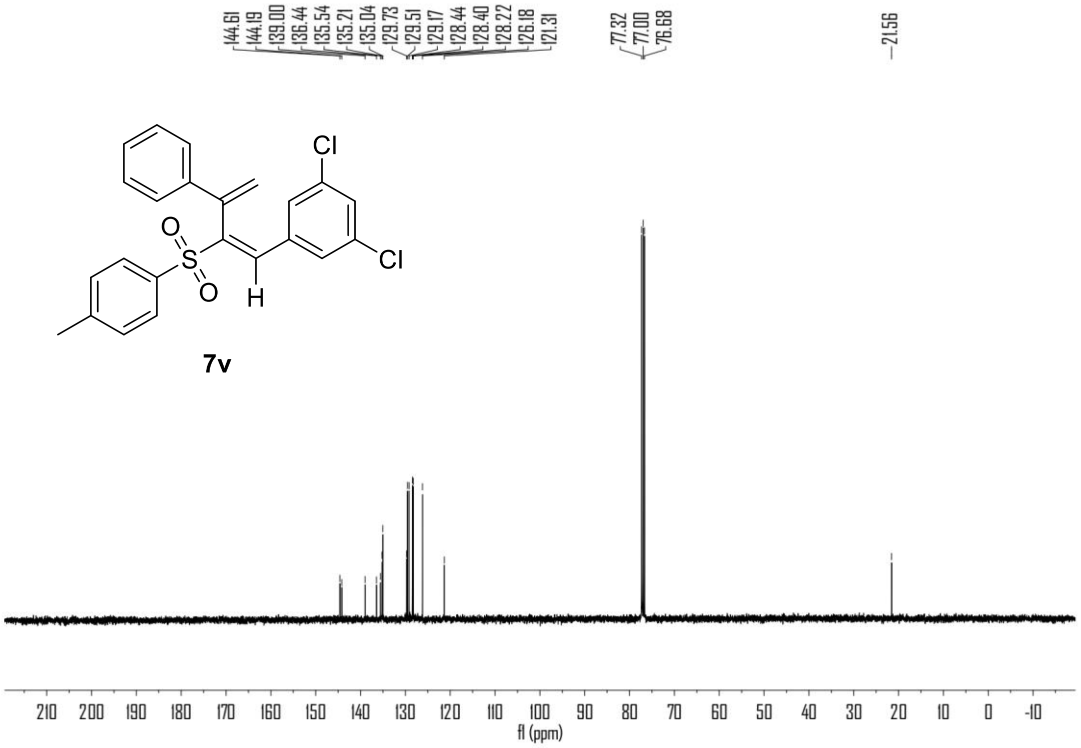
144.61
144.19
139.00
136.44
135.54
135.21
135.04
129.73
129.51
129.17
128.44
128.40
128.22
126.18
121.31
77.32
77.00
76.68
21.56
Cl
Cl
O
S
O
H
7v
fl (ppm)

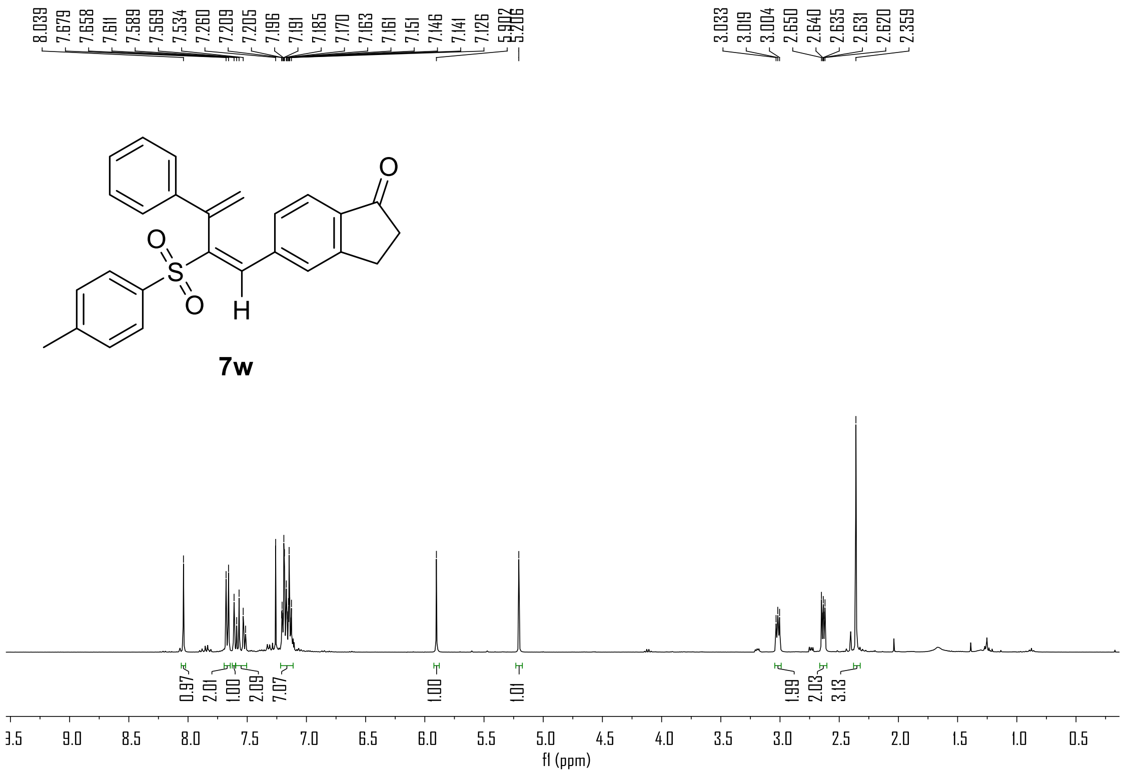
8.039
7.679
7.658
7.611
7.589
7.569
7.534
7.260
7.209
7.205
7.196
7.191
7.185
7.170
7.163
7.161
7.151
7.146
7.141
7.126
5.206
3.033
3.019
3.004
2.650
2.640
2.635
2.631
2.620
2.359
0.97
2.01
1.00
2.09
7.07
1.00
1.01
1.99
2.03
3.13
fl (ppm)

7w

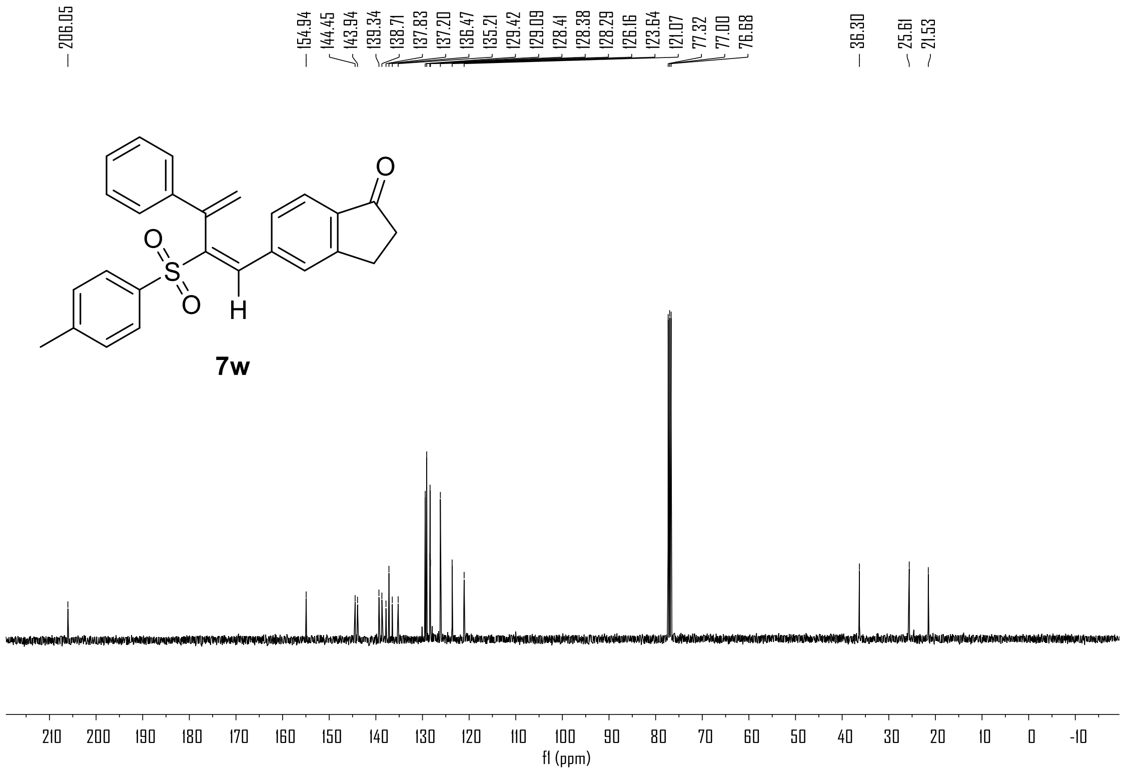
206.05
154.94
144.45
143.94
139.34
138.71
137.83
137.20
136.47
135.21
129.42
129.09
128.41
128.38
128.29
126.16
123.64
121.07
77.32
77.00
76.68
36.30
25.61
21.53
fl (ppm)

7w

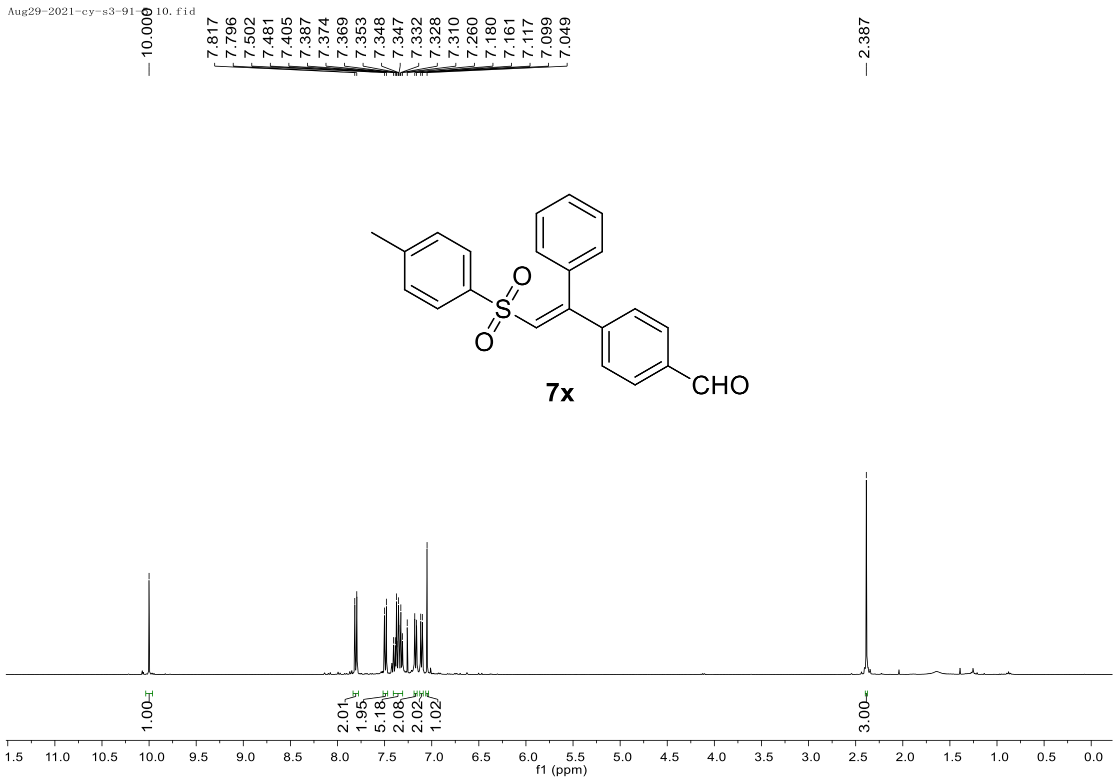

Aug29-2021-cy-s3-91-1-10.fid
10.000
7.817
7.796
7.502
7.481
7.405
7.387
7.374
7.369
7.353
7.348
7.347
7.332
7.328
7.310
7.260
7.180
7.161
7.117
7.099
7.049
2.387
CHO
7x
1.00
2.01
1.95
5.18
2.08
2.02
1.02
3.00
f1 (ppm)

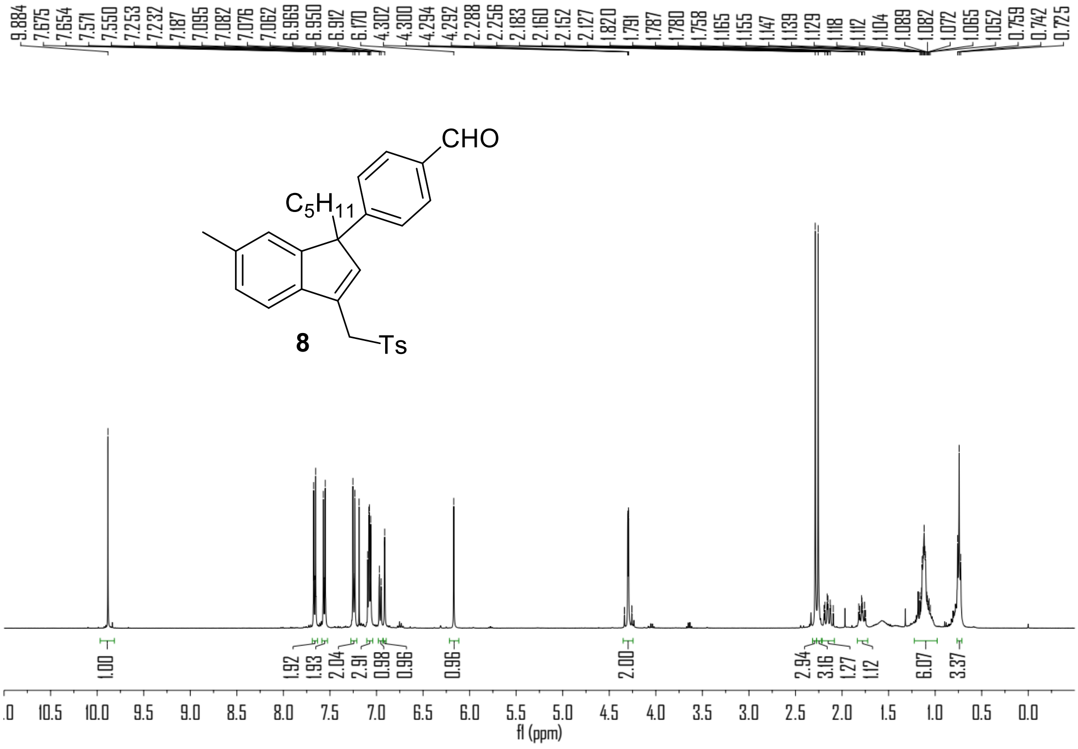
9.884 7.675 7.654 7.571 7.550 7.253 7.232 7.187 7.095 7.082 7.076 7.062 6.969 6.950 6.912 6.170 4.302 4.300 4.294 4.292 2.288 2.256 2.183 2.160 2.152 2.127 1.820 1.791 1.787 1.780 1.758 1.165 1.155 1.147 1.139 1.129 1.118 1.112 1.104 1.089 1.082 1.072 1.065 1.052 0.759 0.742 0.725
CHO
C_5H_{11}
8
Ts
1.00 1.92 1.93 2.04 2.91 0.98 0.96 0.96 2.00 2.94 3.16 1.27 1.12 6.07 3.37
fl (ppm)

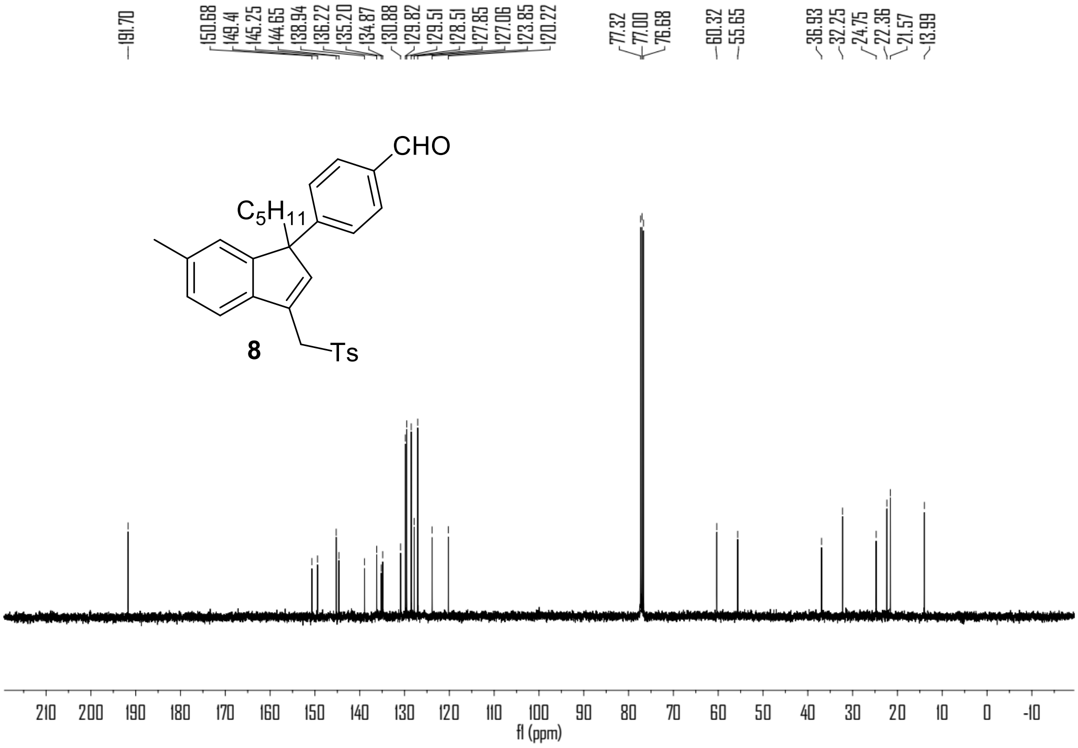
191.70 150.68 149.41 145.25 144.65 138.94 136.22 135.20 134.87 130.88 129.82 129.51 128.51 127.85 127.06 123.85 120.22 77.32 77.00 76.68 60.32 55.65 36.93 32.25 24.75 22.36 21.57 13.99
CHO
C_5H_{11}
8
Ts
fl (ppm)

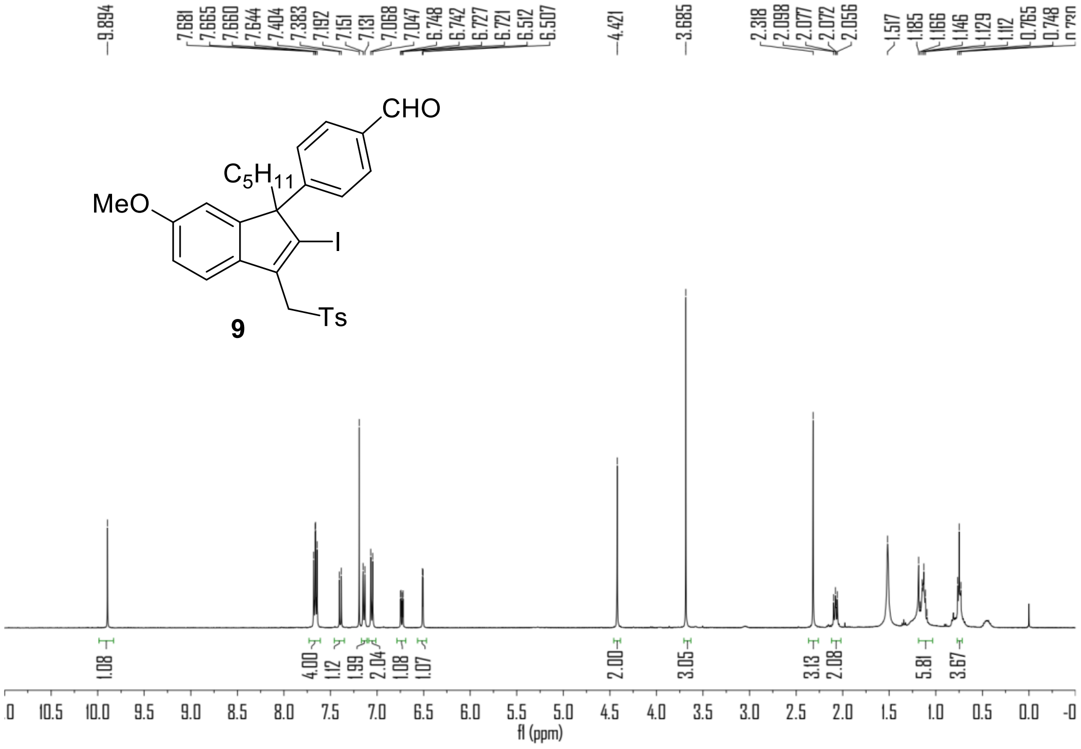
9.894
7.681 7.665 7.660 7.644 7.404 7.383 7.192 7.151 7.131 7.068 7.047 6.748 6.742 6.727 6.721 6.512 6.507
4.421
3.685
2.318 2.098 2.077 2.072 2.056
1.517 1.185 1.166 1.146 1.129 1.112 0.765 0.748
CHO
C5H11
MeO
I
Ts
9
1.08
4.00 1.12 1.99 2.04 1.08 1.07
2.00
3.05
3.13 2.08
5.81
3.67
10.5 10.0 9.5 9.0 8.5 8.0 7.5 7.0 6.5 6.0 5.5 5.0 4.5 4.0 3.5 3.0 2.5 2.0 1.5 1.0 0.5 0.0
f1 (ppm)

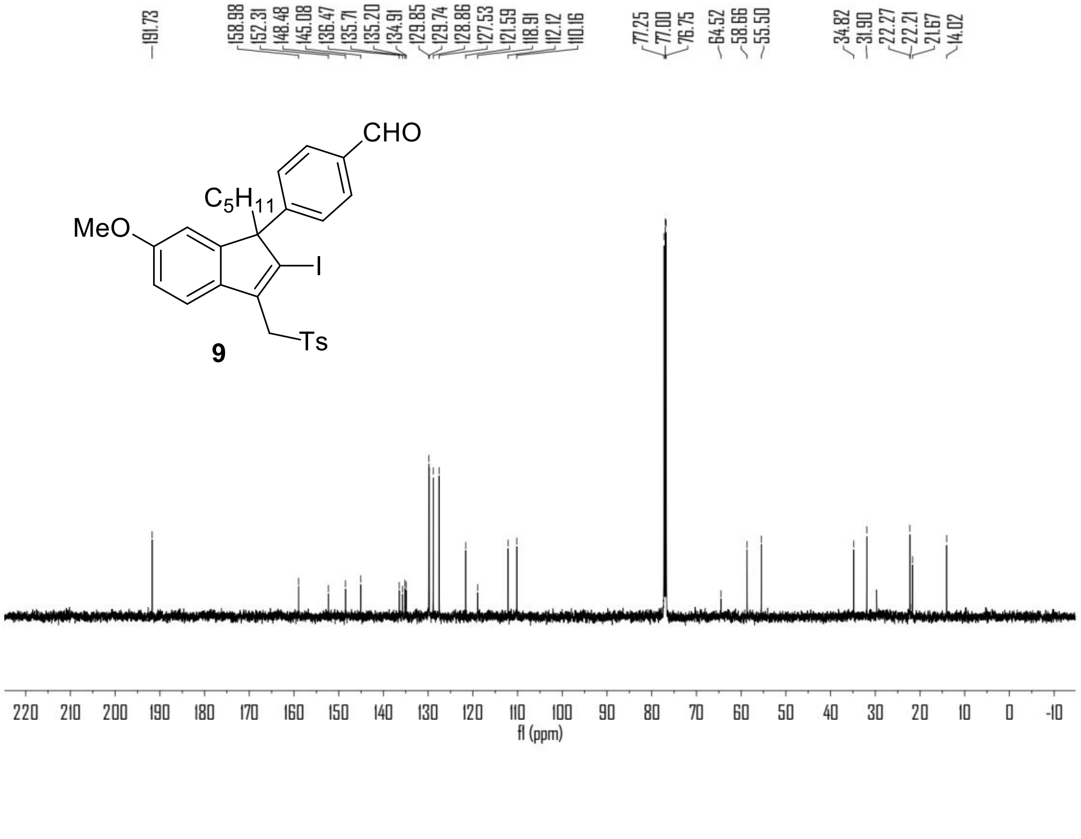
191.73
158.98 152.31 148.48 145.08 136.47 135.71 135.20 134.91 129.85 129.74 128.86 127.53 121.59 118.91 112.12 110.16
77.25 77.00 76.75 64.52 58.66 55.50
34.82 31.90 22.27 22.21 21.67 14.02
CHO
C5H11
MeO
I
Ts
9
220 210 200 190 180 170 160 150 140 130 120 110 100 90 80 70 60 50 40 30 20 10 0 -10
f1 (ppm)

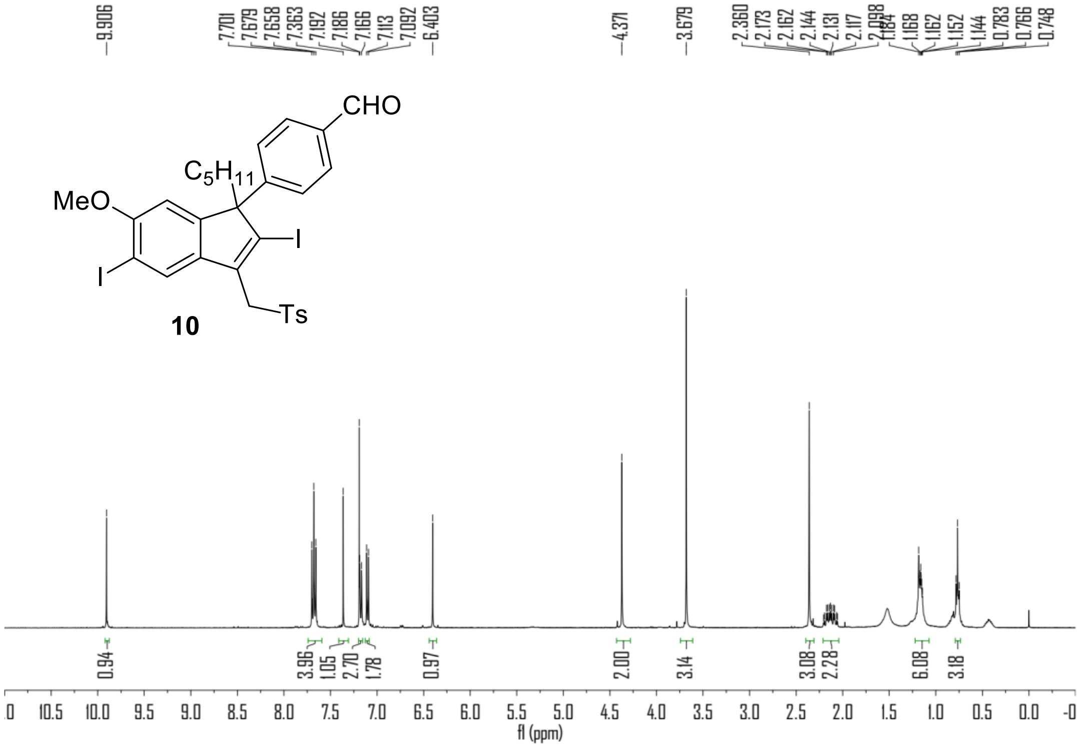
CHO
C_5H_{11}
MeO
I
I
Ts
10
fl (ppm)

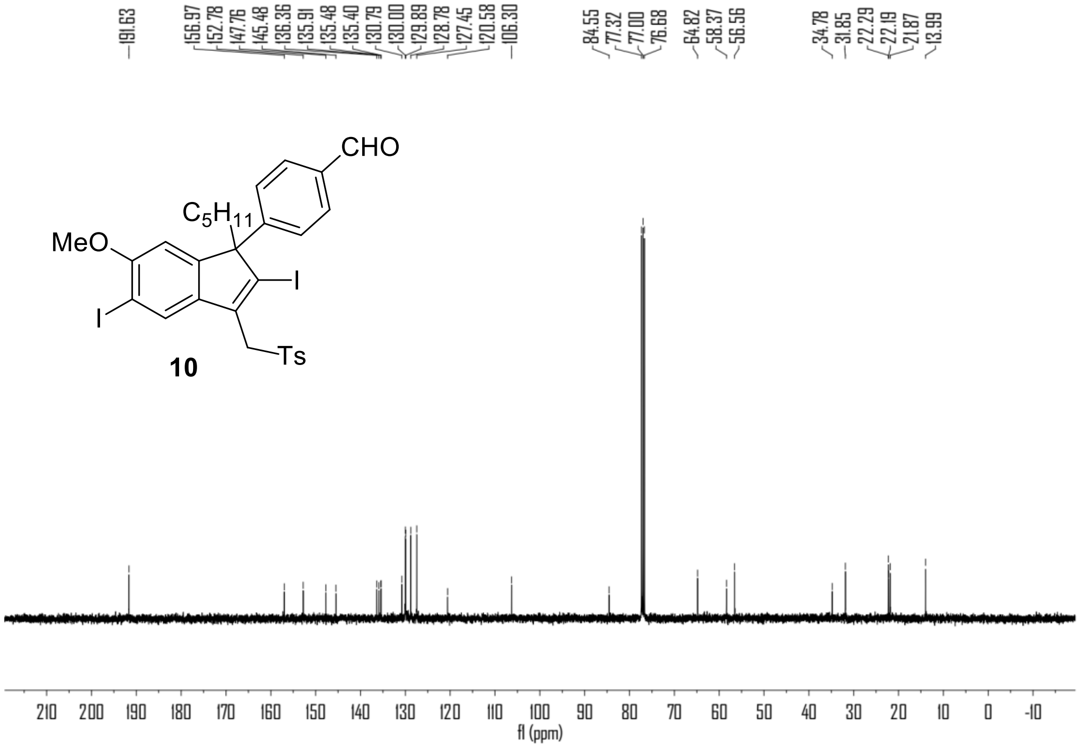
CHO
C_5H_{11}
MeO
I
I
Ts
10
fl (ppm)

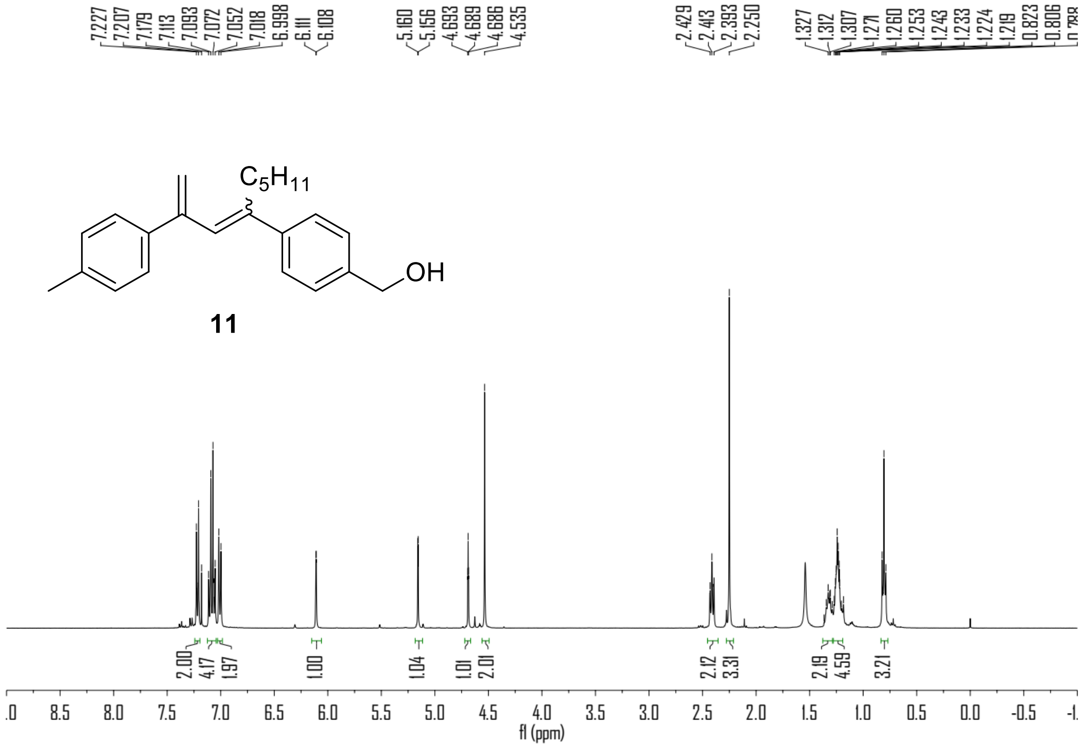
7.227
7.207
7.179
7.113
7.093
7.072
7.052
7.018
6.998
6.111
6.108
5.160
5.156
4.693
4.689
4.686
4.535
2.429
2.413
2.393
2.250
1.327
1.312
1.307
1.271
1.260
1.253
1.243
1.233
1.224
1.219
0.823
0.806
C5H11
OH
11
2.00
4.17
1.97
1.00
1.04
1.01
2.01
2.12
3.31
2.19
4.59
3.21
fl (ppm)

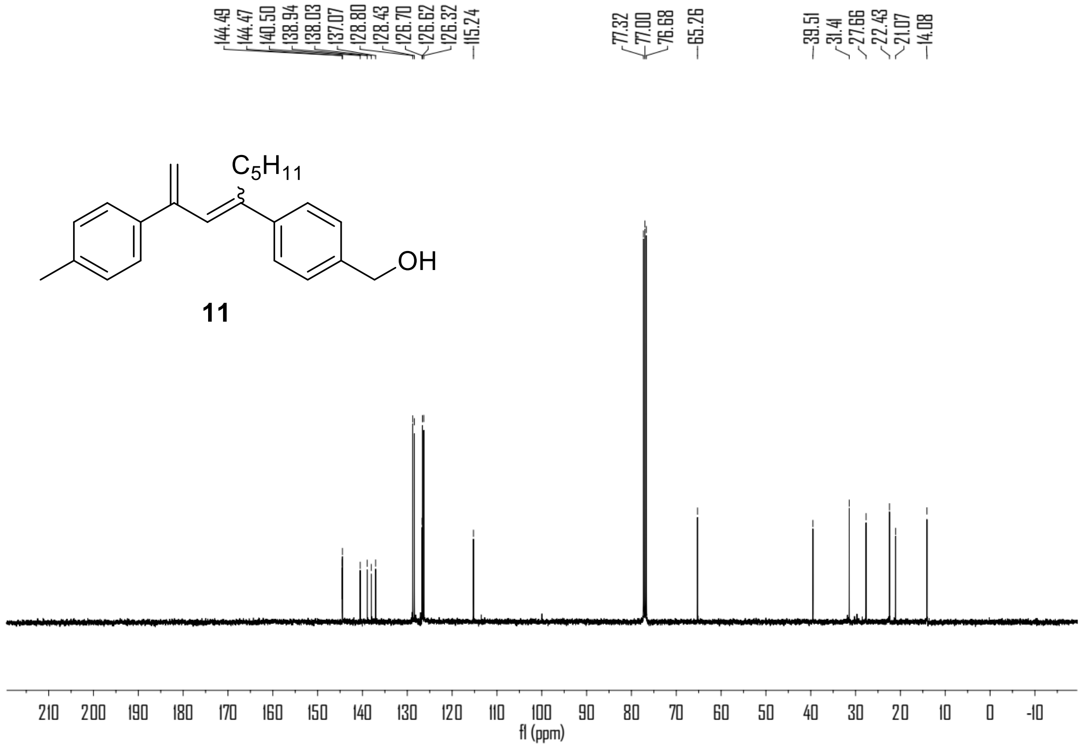
144.49
144.47
140.50
138.94
138.03
137.07
128.80
128.43
126.70
126.62
126.32
115.24
77.32
77.00
76.68
65.26
39.51
31.41
27.66
22.43
21.07
14.08
C5H11
OH
11
fl (ppm)

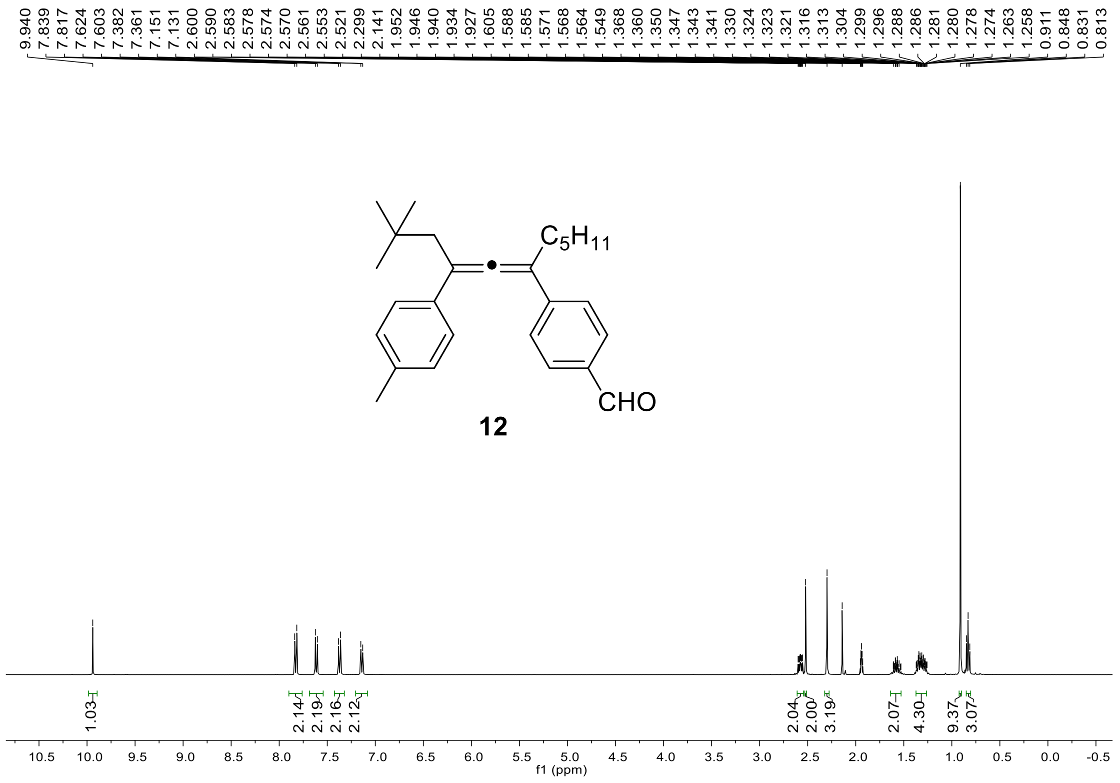
9.940 7.839 7.817 7.624 7.603 7.382 7.361 7.151 7.131 2.600 2.590 2.583 2.578 2.574 2.570 2.561 2.553 2.521 2.299 2.141 1.952 1.946 1.940 1.934 1.927 1.605 1.588 1.585 1.571 1.568 1.564 1.549 1.368 1.360 1.350 1.347 1.343 1.341 1.330 1.324 1.323 1.321 1.316 1.313 1.304 1.299 1.296 1.288 1.286 1.281 1.280 1.278 1.274 1.263 1.258 0.911 0.848 0.831 0.813
C_5H_{11}
CHO
12
1.03 2.14 2.19 2.16 2.12 2.04 2.00 3.19 2.07 4.30 9.37 3.07
f1 (ppm)

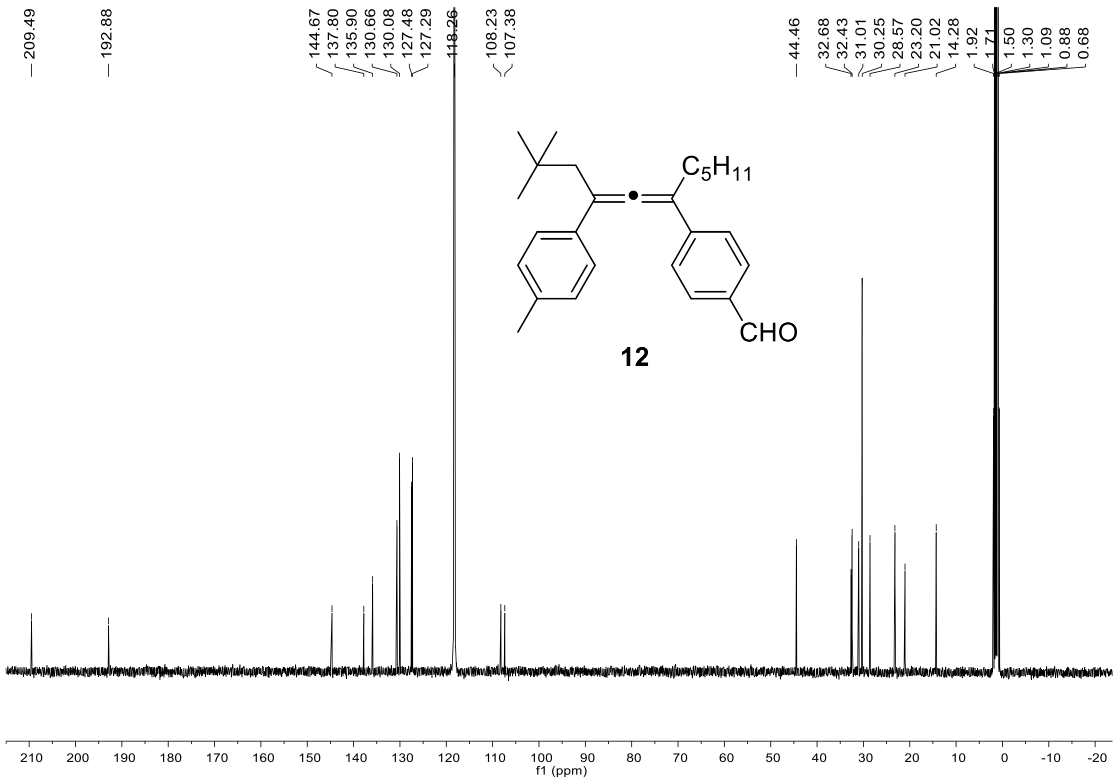
209.49 192.88 144.67 137.80 135.90 130.66 130.08 127.48 127.29 118.26 108.23 107.38 44.46 32.68 32.43 31.01 30.25 28.57 23.20 21.02 14.28 1.92 1.71 1.50 1.30 1.09 0.88 0.68
C_5H_{11}
CHO
12
f1 (ppm)

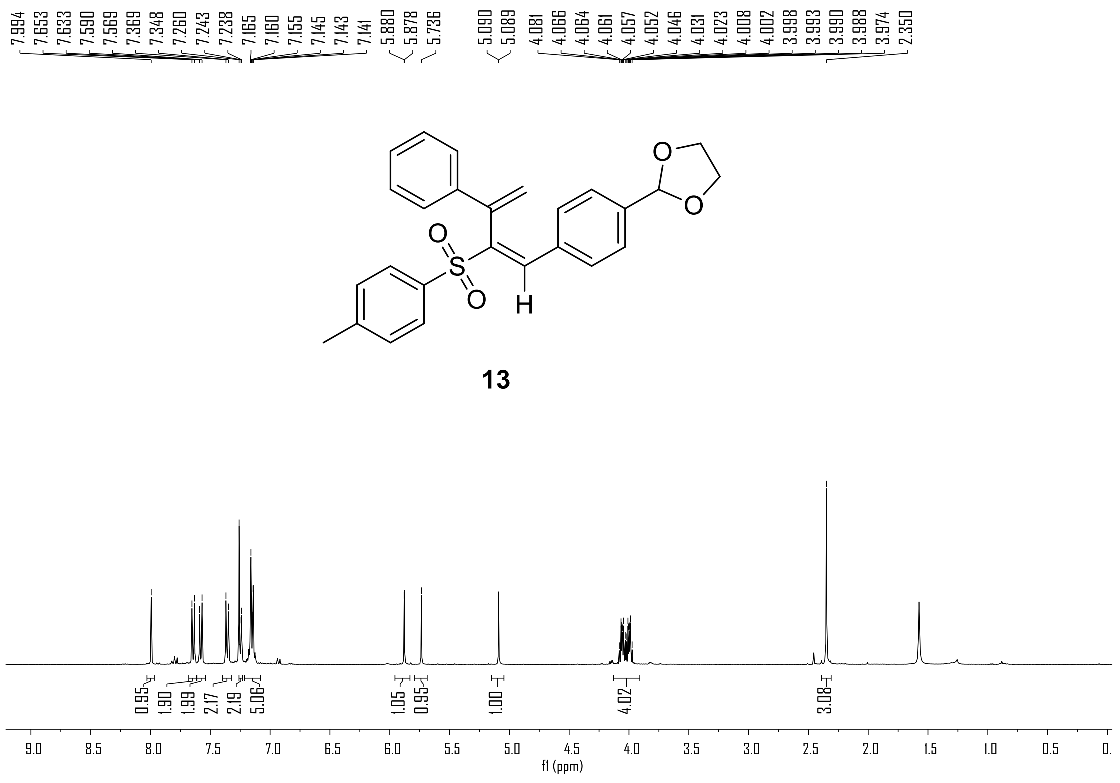
7.994
7.653
7.633
7.590
7.569
7.369
7.348
7.260
7.243
7.238
7.165
7.160
7.155
7.145
7.143
7.141
5.880
5.878
5.736
5.090
5.089
4.081
4.066
4.064
4.061
4.057
4.052
4.046
4.031
4.023
4.008
4.002
3.998
3.993
3.990
3.988
3.974
2.350
O
O
O
S
O
H
13
0.95
1.90
1.99
2.17
2.19
5.06
1.05
0.95
1.00
4.02
3.08
f1 (ppm)

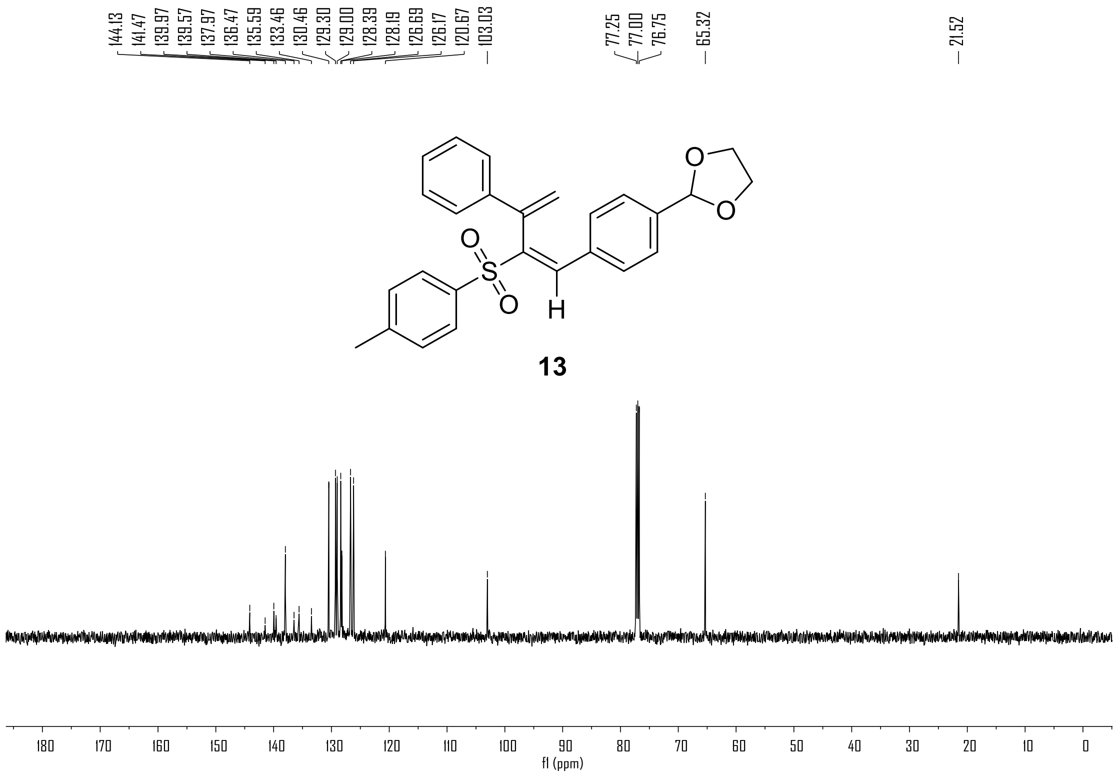
144.13
141.47
139.97
139.57
137.97
136.47
135.59
133.46
130.46
129.30
129.00
128.39
128.19
126.69
126.17
120.67
103.03
77.25
77.00
76.75
65.32
21.52
O
O
O
S
O
H
13
f1 (ppm)

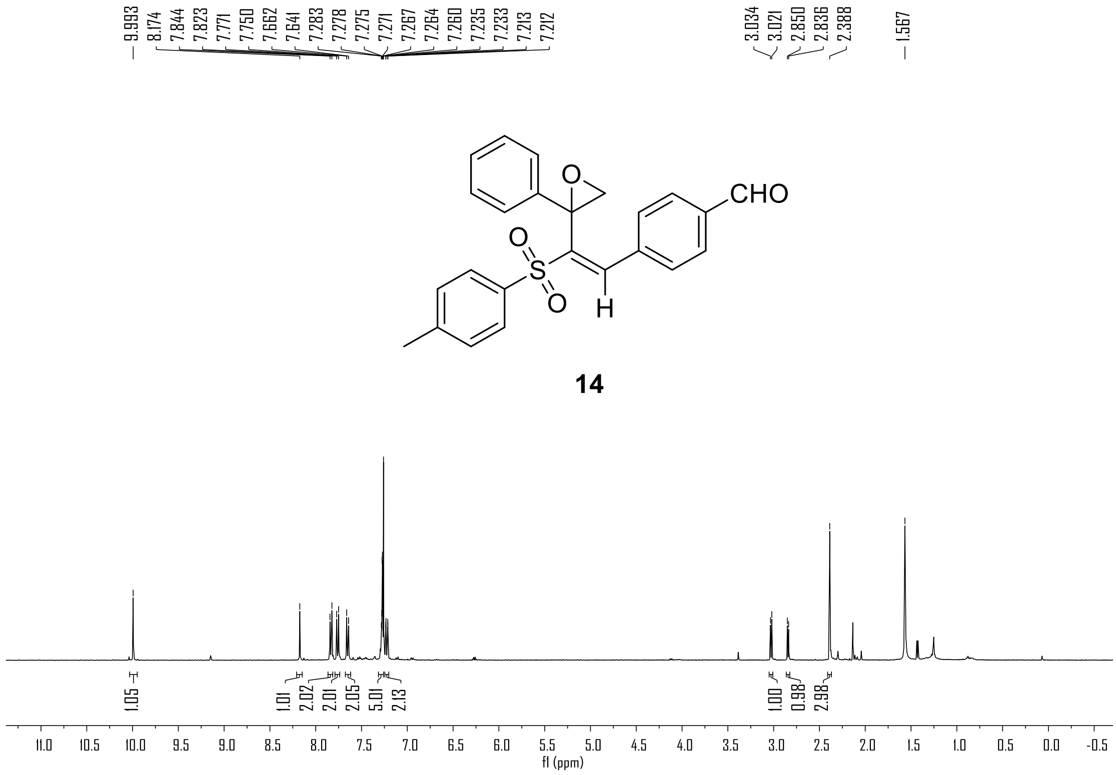
9.993
8.174
7.844
7.823
7.771
7.750
7.662
7.641
7.283
7.278
7.275
7.271
7.267
7.264
7.260
7.235
7.233
7.213
7.212
3.034
3.021
2.850
2.836
2.388
1.567
O
CHO
O
S
O
H
14
1.05
1.01
2.02
2.01
2.05
5.01
2.13
1.00
0.98
2.98
f1 (ppm)

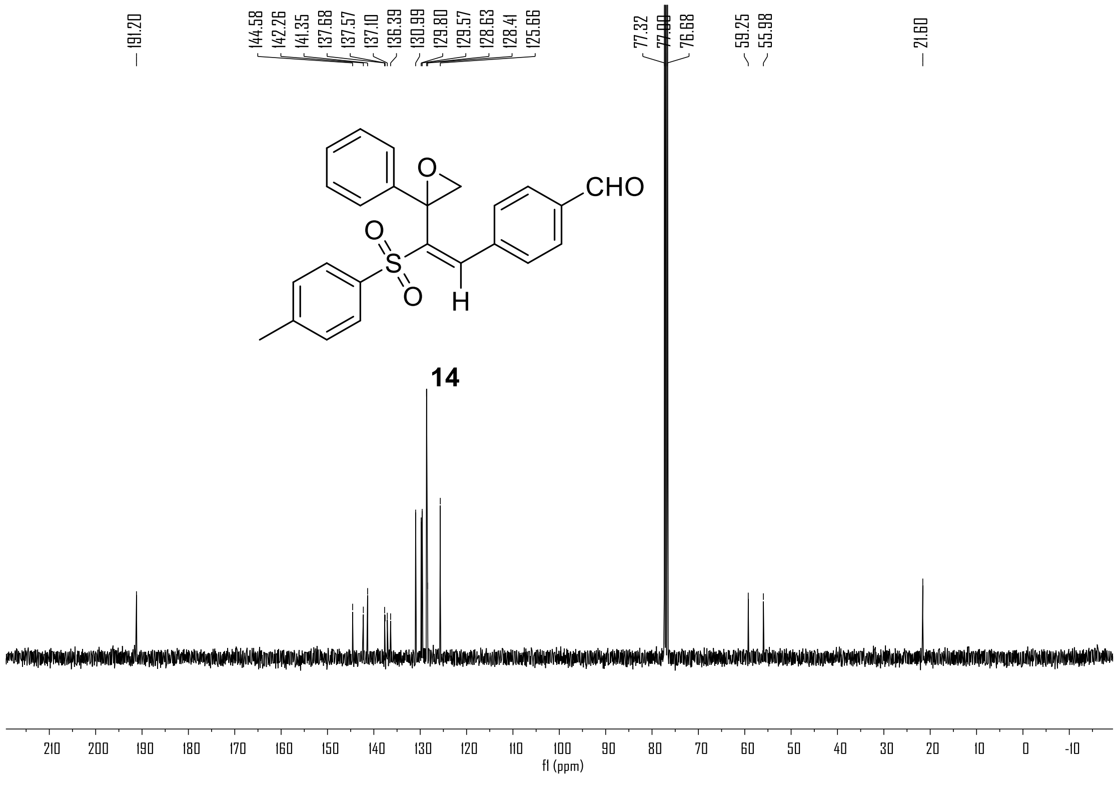
191.20
144.58
142.26
141.35
137.68
137.57
137.10
136.39
130.99
129.80
129.57
128.63
128.41
125.66
77.32
77.00
76.68
59.25
55.98
21.60
O
CHO
O
S
O
H
14
f1 (ppm)

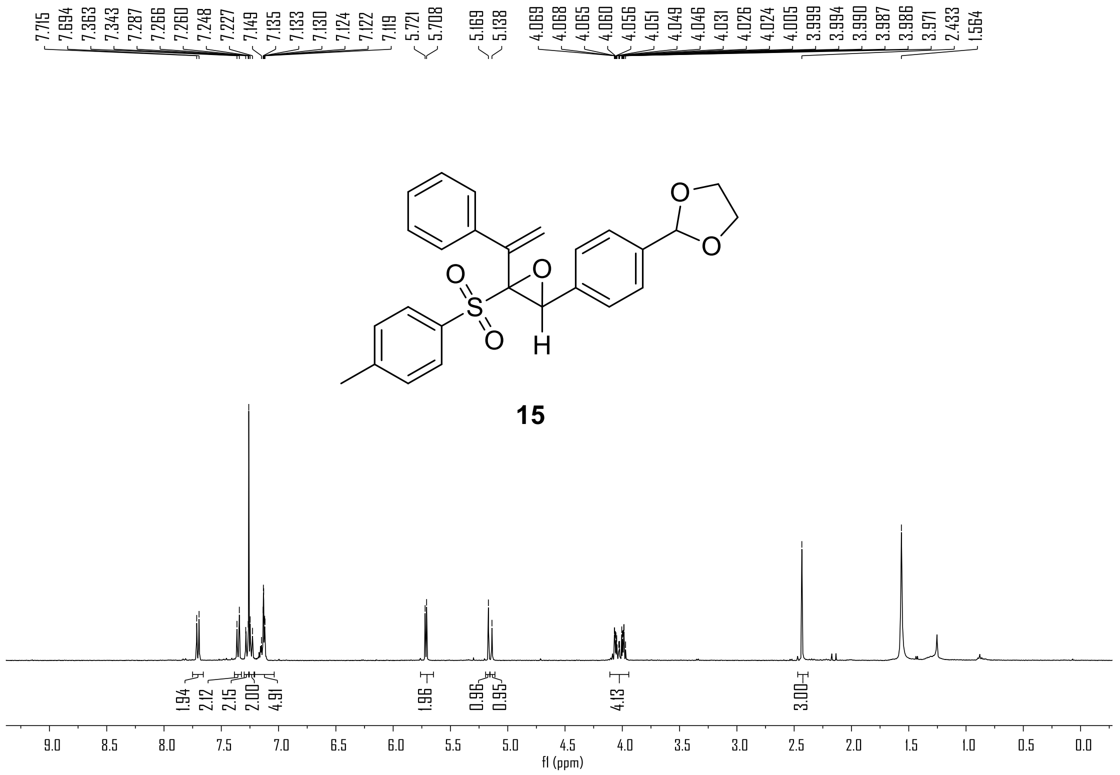
7.715 7.694 7.363 7.343 7.287 7.266 7.260 7.248 7.227 7.149 7.135 7.133 7.130 7.124 7.122 7.119 5.721 5.708 5.169 5.138 4.069 4.068 4.065 4.060 4.056 4.051 4.049 4.046 4.031 4.026 4.024 4.005 3.999 3.994 3.990 3.987 3.986 3.971 2.433 1.564
O
O
O
S
O
O
H
1.94 2.12 2.15 2.00 4.91 1.96 0.96 0.95 4.13 3.00
9.0 8.5 8.0 7.5 7.0 6.5 6.0 5.5 5.0 4.5 4.0 3.5 3.0 2.5 2.0 1.5 1.0 0.5 0.0
f1 (ppm)

15

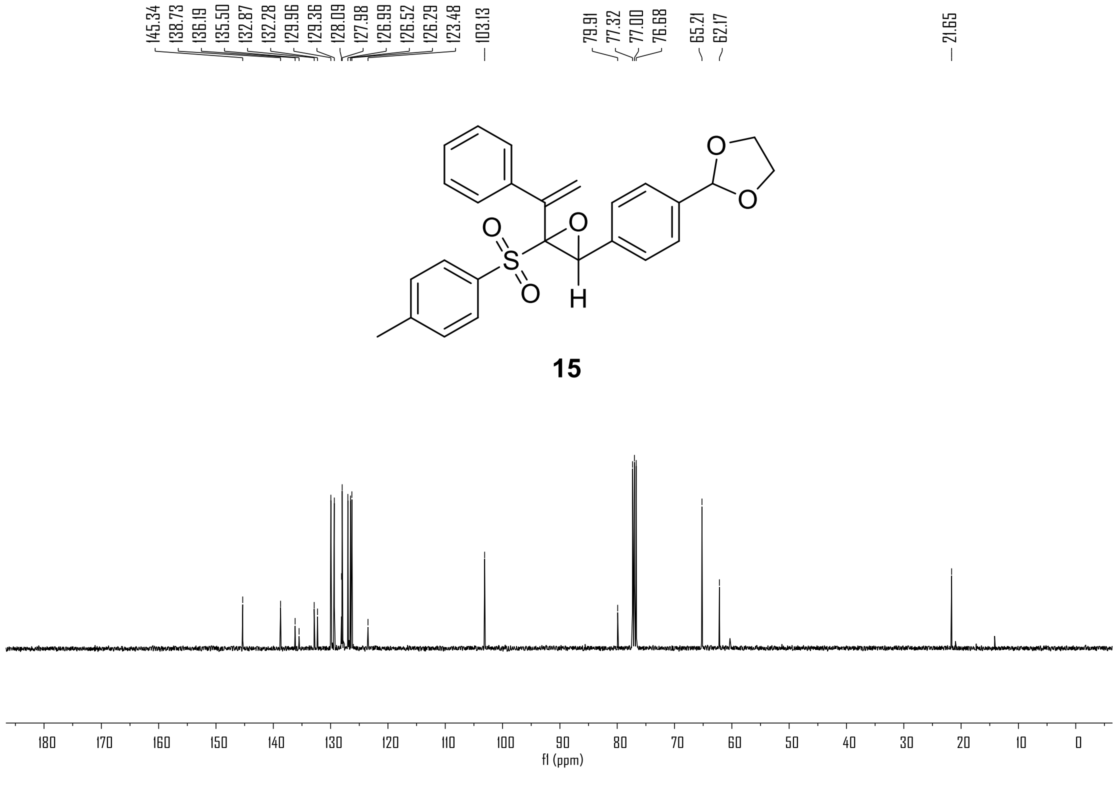
145.34 138.73 136.19 135.50 132.87 132.28 129.96 129.36 128.09 127.98 126.99 126.52 126.29 123.48 103.13
79.91 77.32 77.00 76.68 65.21 62.17 21.65
O
O
O
S
O
O
H
180 170 160 150 140 130 120 110 100 90 80 70 60 50 40 30 20 10 0
f1 (ppm)

15

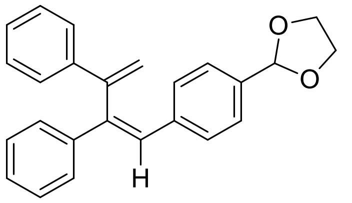

16

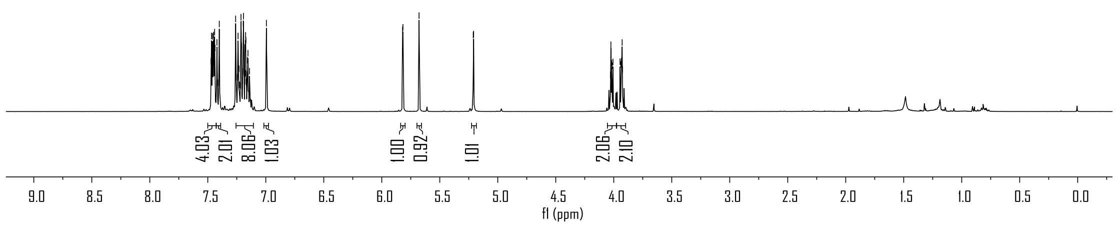
7.472
7.468
7.462
7.458
7.454
7.453
7.450
7.447
7.445
7.441
7.438
7.421
7.401
7.260
7.239
7.231
7.214
7.209
7.198
7.194
7.192
7.188
7.187
7.180
7.177
7.174
7.171
7.158
7.154
7.140
6.995
5.821
5.817
5.678
5.211
5.208
4.025
4.023
4.019
4.014
4.007
3.946
3.938
3.933
3.929
3.927
3.924
4.03
2.01
8.06
1.03
1.00
0.92
1.01
2.06
2.10
9.0
8.5
8.0
7.5
7.0
6.5
6.0
5.5
5.0
4.5
4.0
3.5
3.0
2.5
2.0
1.5
1.0
0.5
0.0
f1 (ppm)

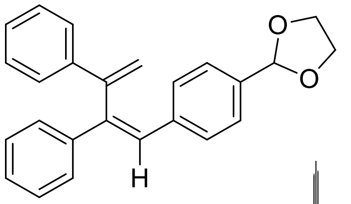

16

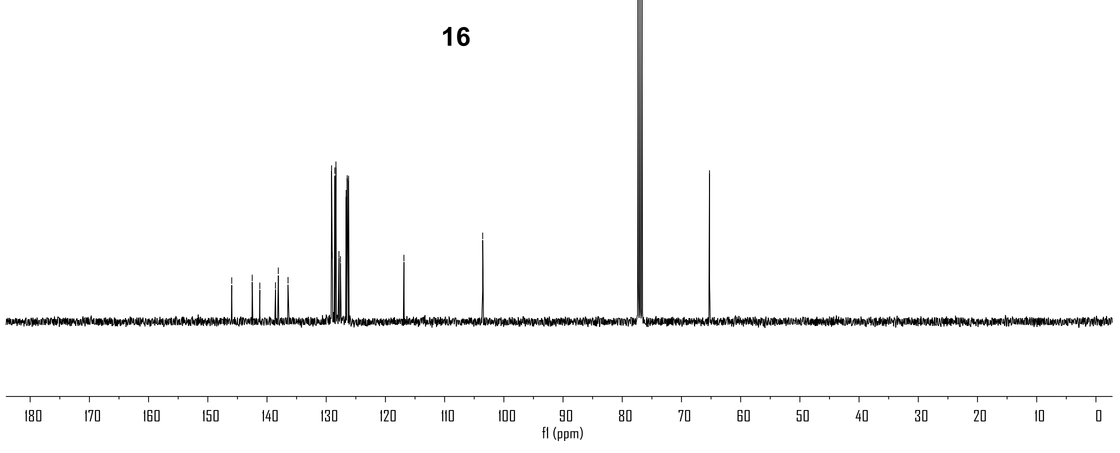
145.96
142.50
141.22
138.57
138.09
136.45
129.09
128.98
128.56
128.36
127.85
127.60
126.63
126.45
126.22
116.88
103.57
77.32
77.00
76.68
65.26
180
170
160
150
140
130
120
110
100
90
80
70
60
50
40
30
20
10
0
f1 (ppm)

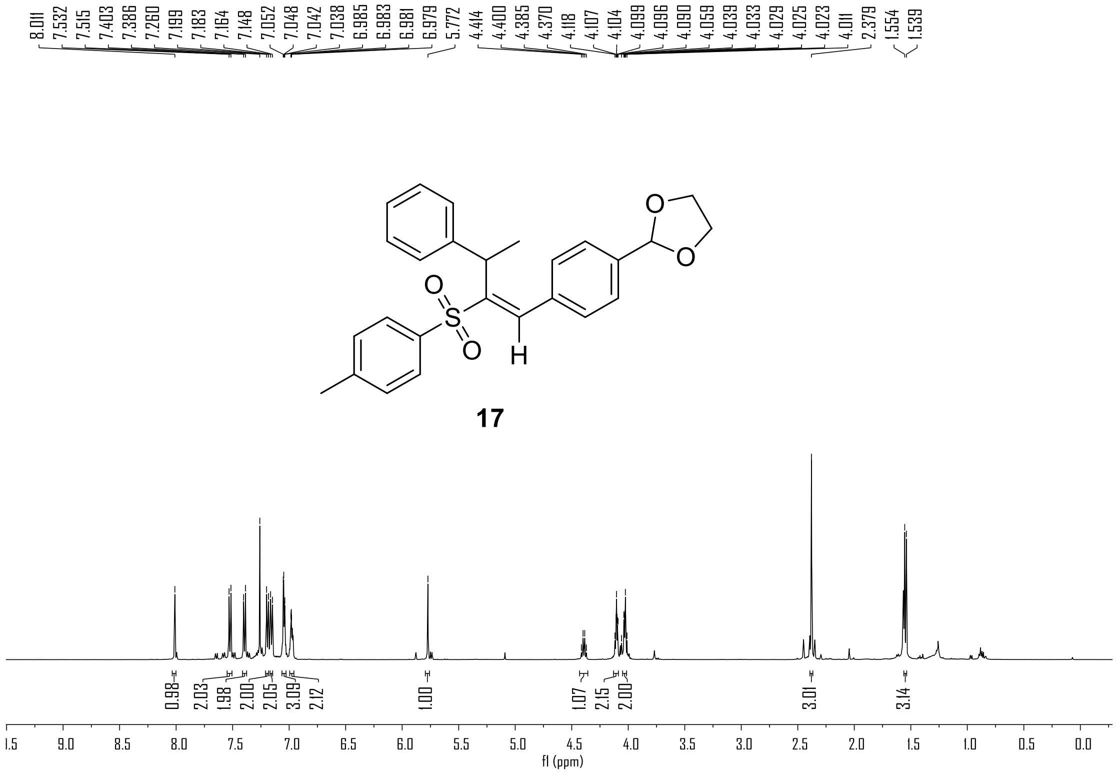
8.011
7.532
7.515
7.403
7.386
7.260
7.199
7.183
7.164
7.148
7.052
7.048
7.042
7.038
6.985
6.983
6.981
6.979
5.772
4.414
4.400
4.385
4.370
4.118
4.107
4.104
4.099
4.096
4.090
4.059
4.039
4.033
4.029
4.025
4.023
4.011
2.379
1.554
1.539
O
O
O
S
O
H
17
0.98
2.03
1.98
2.00
2.05
3.09
2.12
1.00
1.07
2.15
2.00
3.01
3.14
9.0
8.5
8.0
7.5
7.0
6.5
6.0
5.5
5.0
4.5
4.0
3.5
3.0
2.5
2.0
1.5
1.0
0.5
0.0
fl (ppm)

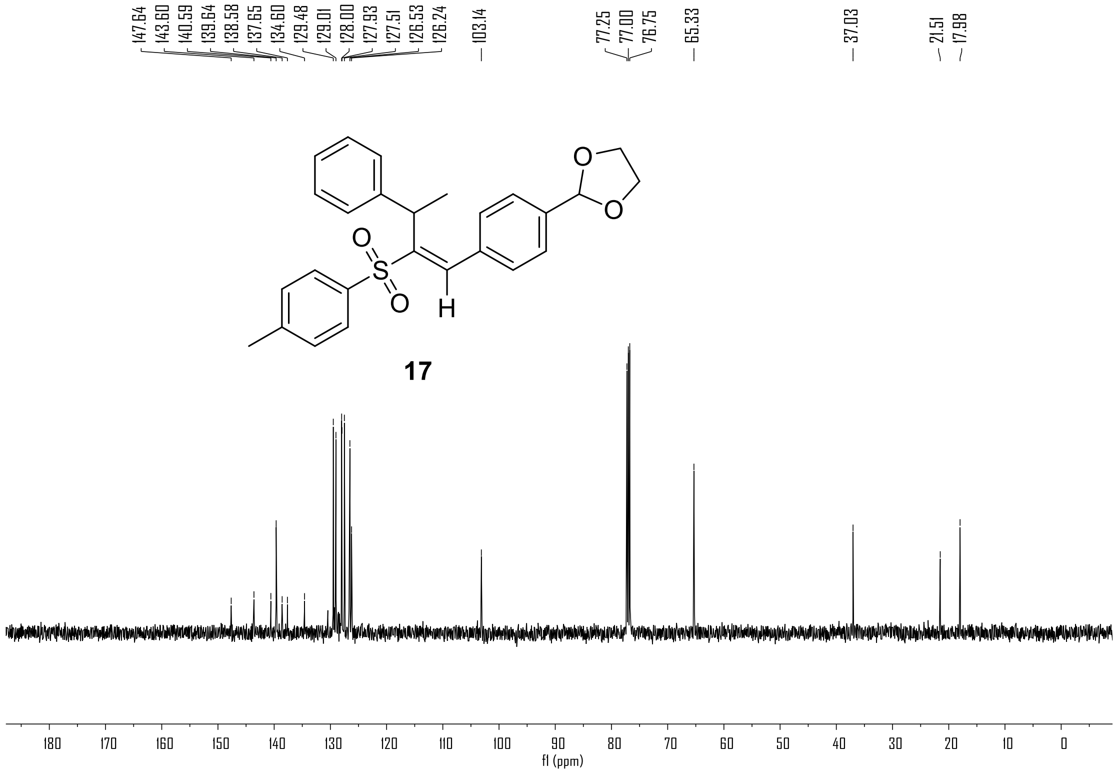
147.64
143.60
140.59
139.64
138.58
137.65
134.60
129.48
129.01
128.00
127.93
127.51
126.53
126.24
103.14
77.25
77.00
76.75
65.33
37.03
21.51
17.98
O
O
O
S
O
H
17
180
170
160
150
140
130
120
110
100
90
80
70
60
50
40
30
20
10
0
fl (ppm)

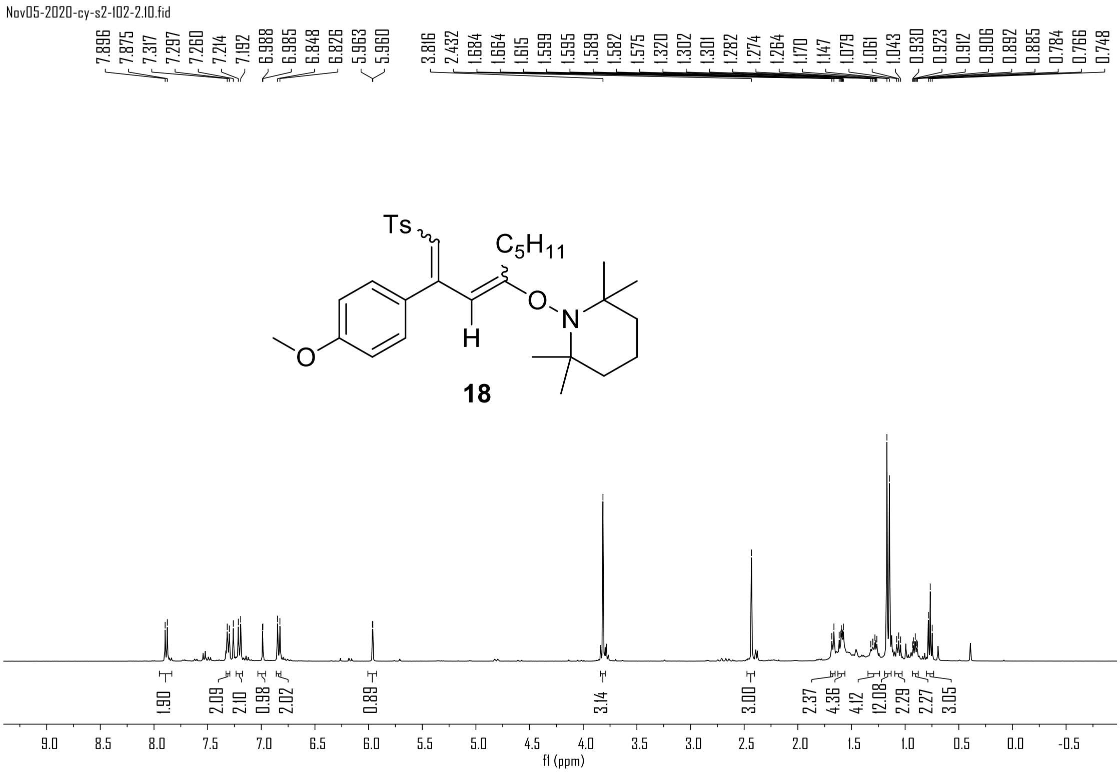
Nov05-2020-cy-s2-102-2.10.fid
7.896 7.875 7.317 7.297 7.260 7.214 7.192 6.988 6.985 6.848 6.826 5.963 5.960
3.816 2.432 1.684 1.664 1.615 1.599 1.595 1.589 1.582 1.575 1.320 1.302 1.301 1.282 1.274 1.264 1.170 1.147 1.079 1.061 1.043 0.930 0.923 0.912 0.906 0.892 0.885 0.784 0.766 0.748
Ts
C5H11
O
N
H
18
1.90 2.09 2.10 0.98 2.02 0.89 3.14 3.00 2.37 4.36 4.12 12.08 2.29 2.27 3.05
9.0 8.5 8.0 7.5 7.0 6.5 6.0 5.5 5.0 4.5 4.0 3.5 3.0 2.5 2.0 1.5 1.0 0.5 0.0 -0.5
f1 (ppm)

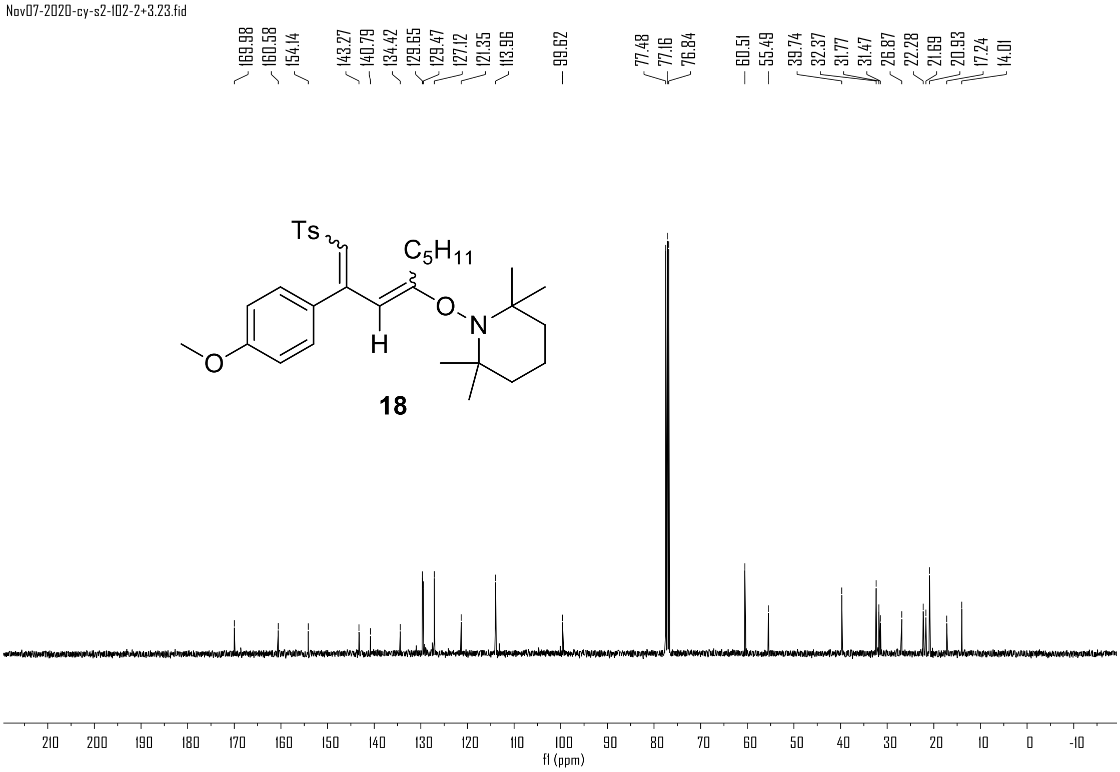
Nov07-2020-cy-s2-102-2+3.23.fid
169.98 160.58 154.14 143.27 140.79 134.42 129.65 129.47 127.12 121.35 113.96 99.62 77.48 77.16 76.84 60.51 55.49 39.74 32.37 31.77 31.47 26.87 22.28 21.69 20.93 17.24 14.01
Ts
C5H11
O
N
H
18
210 200 190 180 170 160 150 140 130 120 110 100 90 80 70 60 50 40 30 20 10 0 -10
f1 (ppm)
